# Virus versus Host Plant MicroRNAs: Who Determines the Outcome of the Interaction?

**DOI:** 10.1371/journal.pone.0098263

**Published:** 2014-06-04

**Authors:** Fatemeh Maghuly, Rose C. Ramkat, Margit Laimer

**Affiliations:** 1 Plant Biotechnology Unit (PBU), Department Biotechnology, University of Natural Resources and Life Sciences, BOKU-VIBT, Vienna, Austria; 2 Department of Biological Sciences, Egerton University, Nakuru, Kenya; Virginia Tech, United States of America

## Abstract

Considering the importance of microRNAs (miRNAs) in the regulation of essential processes in plant pathogen interactions, it is not surprising that, while plant miRNA sequences counteract viral attack via antiviral RNA silencing, viruses in turn have developed antihost defense mechanisms blocking these RNA silencing pathways and establish a counter-defense. In the current study, computational and stem-loop Reverse Transcription – Polymerase Chain Reaction (RT-PCR) approaches were employed to a) predict and validate virus encoded mature miRNAs (miRs) in 39 DNA-A sequences of the bipartite genomes of *African cassava mosaic virus* (ACMV) and *East African cassava mosaic virus*-Uganda (EACMV-UG) isolates, b) determine whether virus encoded miRs/miRs* generated from the 5′/3′ harpin arms have the capacity to bind to genomic sequences of the host plants *Jatropha* or cassava and c) investigate whether plant encoded miR/miR* sequences have the potential to bind to the viral genomes. Different viral pre-miRNA hairpin sequences and viral miR/miR* length variants occurring as isomiRs were predicted in both viruses. These miRNAs were located in three Open Reading Frames (ORFs) and in the Intergenic Region (IR). Moreover, various target genes for miRNAs from both viruses were predicted and annotated in the host plant genomes indicating that they are involved in biotic response, metabolic pathways and transcription factors. Plant miRs/miRs* from conserved and highly expressed families were identified, which were shown to have potential targets in the genome of both begomoviruses, representing potential plant miRNAs mediating antiviral defense. This is the first assessment of predicted viral miRs/miRs* of ACMV and EACMV-UG and host plant miRNAs, providing a reference point for miRNA identification in pathogens and their hosts. These findings will improve the understanding of host- pathogen interaction pathways and the function of viral miRNAs in *Euphorbiaceous* crop plants.

## Introduction

*Jatropha curcas* (*Euphorbiaceae*) is a drought resistant plant, native to tropical America, now widely cultivated in tropical and subtropical regions for harvesting a unique oil contained in its seeds, which can be used as raw material for the production of biofuel [1]–[2]. In addition, different parts of *Jatropha* containing a range of interesting metabolites and medicinal components [3]–[4] have long been used as raw material for lamp oil, soap production, paints, lubricating oils, and for medical applications [1], [5]. Its seeds contain 30–45% toxic oil, with a high percentage of monounsaturated oleic and polyunsaturated linoleic acid [6]–[8]. The press cake from seeds is also rich in proteins (60–63%) compared to soybean (45%) [9]. On the one hand, cassava (*Manihot esculenta* Crantz) is a key staple food in sub-Saharan Africa. Cassava is a potential source of biomass for bioethanol production and its root is a good source of carbohydrates, but a poor source of proteins. However, these economically important crops can be infected by several geminiviruses, like ACMV and EACMV-UG [10], causing severe losses.

The bipartite genomes of ACMV and EACMV-UG consist of two components, DNA-A and DNA-B, each of 2.7–3 kb [11], which encodes 6 and 2 ORFs, respectively. The ORFs AV1 and AV2 are located on the sense strand of DNA-A, while AC1 to AC4 reside on the complementary strand. AV1 (Coat Protein, CP) is essential for viral transmission by whiteflies, *Bemisia tabaci*, while AV2 (pre-CP) is involved in virus movement [12]. AC1 (Replication associated-protein, Rep) is required for replication [13], while AC2 (Transcriptional Activator Protein, TrAP), required for the transcription – activation of plus strand genes, is also involved in suppression of Post Transcriptional Gene Silencing (PTGS) [14]. AC3 (Replication Enhancer protein, REn) enhances viral DNA accumulation [15]. The pathogenicity enhancer protein AC4 plays a crucial role in pathogenicity and PTGS [16]. The IR contains the invariant TAATAT/AC motif responsible for the initiation of rolling circle DNA replication [17].

RNA silencing is a conserved defense mechanism that plants and other eukaryotes use to protect their genomes against aberrant nucleic acids, like viruses. This process uses short RNAs (20–30 nt) to recognize and manipulate complementary nucleic acids [18]. Any pathogen able to establish a successful infection must evade this line of defense [19]. As a result, several viruses encode ORFs termed suppressors of PTGS that compromise the RNA silencing pathways of the host plant, including both miRNAs and small interfering RNAs (siRNAs) [18], [20]. In this way viruses are able to control viral and host gene expression [21]–[22].

MiRNAs constitute a class of small RNAs of 21–24 nucleotides that regulate gene expression at the post-transcriptional level by targeting specific messenger RNAs (mRNAs) for cleavage or translational repression [23]. They are expressed in plants and animals, as well as in several viruses [22]–[26]. Primary miRNA (pri-miRNA) transcripts are first cleaved by the nuclear based Dicer like enzyme, resulting in the release of short stem loop precursor miRNA (pre-miRNA) [7]. miRNA genes are transcribed from pre-miRNAs, in which mature miRNAs reside either on the 5′ or the 3′ arm [28]–[29]. MiRs are processed from stem-loop regions by a Dicer like enzyme and loaded into the RNA-Induced Silencing Complex (RISC), where they directly cleave messenger RNAs (mRNAs), while the other strand, also named miR*, is released and degraded [19], [27].

**Figure 1:**
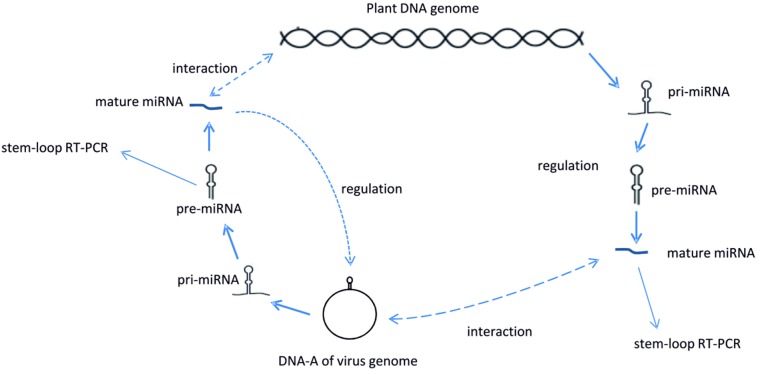
Schematic overview of virus host interactions. The potential interactions between *Euphorbiaceae* and begomoviruses.

There are different methods for identifying miRNAs including cloning, NextGen sequencing and computational approaches. Due to the difficulty to systematically detect miRNAs from a host or pathogen genome by available experimental techniques, especially for those with low expression [30], the computational approach has been applied to identify miRNAs [31]–[33]. At present, computational approaches can be divided into three types: a) the integrated approach based on algorithms, b) comparative genomic approach based on evolutionary conservation and c) *ab initio* prediction based on sequence and structure features [33]. An integrated approach uses two or more computational approaches to improve the sensitivity or specificity of predictions [34]. The comparative genomic approach is not suitable for virus miRNAs prediction, since for many viruses, only very distant evolutionary orthologs are known [35]. Thus *ab initio* prediction methods appear the method of choice [36]. In the current study a combination of different *ab initio* computational and stem-loop Reverse Transcription - Polymerase Chain Reaction (RT-PCR) approaches [37]–[40] were performed to investigate if a) virus miRNAs have the capacity to interact with host ORFs or b) host miRNAs have any possibility to suppress virus ORFs (Figure 1). The stem-loop reverse transcription is based on a miRNA-specific stem-loop RT primer that hybridizes to the 3′ end of the mature miRNA, which increases the sensitivity and provides higher specificity than linear primers because of base stacking and spatial constraints of the stem-loop structure [39], [41]–[42].

Due to the devastating impact by begomoviruses on *Jatropha* and cassava, the situation calls for the provision of stable virus resistant plants to offer a long term solution. Based on the knowledge that members of the *Euphorbiaceae* family share the same pathogens [11], identifying miRNAs both of pathogens and hosts will improve the understanding of host pathogen interaction and in turn benefit *Euphorbiaceae* health. The obtained results reveal important implications for miRNAs encoded by begomoviruses, which are located in genes acting as suppressor of PTGS, and their targets in pathways related to plant pathogenesis. They also show how plants can utilize cooperative regulation by employing multiple miRNAs to target the ORFs of ACMV and EACMV-UG to strengthen their defense mechanisms against viruses.

## Materials and Methods

### Datasets

Thirty three complete DNA-A sequences (9 from ACMV and 24 from EACMV-UG) isolated from infected *Jatropha* and six complete DNA-A sequences (2 from ACMV and 4 from EACMV-UG) isolated from infected cassava [11] were used to predict virus miRNAs (Table 1). *Jatropha* and cassava Expressed Sequence Tags (ESTs) from the GenBank (http://www.ncbi.nlm.nih.gov/genba- nk) were used to predict virus miRNA targets.

**Table 1:** Sequences of DNA A of 11 ACMV and 28 EACMV-UG deposited in the Genbank were compared [4].

ACMV	EACMV-UG
JN053421	JN053432
JN053422	JN053433
JN053423	JN053434
JN053424	JN053435
JN053425	JN053436
JN053426	JN053437
JN053427	JN053438
JN053428	JN053439
JN053429	JN053440
**JN053430**	JN053441
**JN053431**	JN053442
	JN053443
	JN053444
	JN053445
	JN053446
	JN053447
	JN053448
	JN053449
	JN053450
	JN053451
	JN053452
	JN053453
	JN053454
	JN053455
	**JN053456**
	**JN053457**
	**JN053458**
	**JN053459**

The sequences were isolated from *Jatropha* and cassava (in bold).

### Plant Material

Cuttings of 6 symptomatic *Jatropha* (K5J5, K7J3, K7J8, K7J9, K8J5, K8J6) and 5 cassava (B4C3, K5C6, S2C6, S4C4, S4C6) plants as well as one symptomless plant of both species S4J12 and B2C15, respectively, from three districts in Kenya (Kakamega, Siaya, Busia) were collected in private fields (no specific permission was required to sample plant material from farmers crops) by Rose Ramkat from Egerton University (contact person for future permissions), and planted in the glasshouse of the Plant Biotechnology Unit (PBU) for gene expression analysis [11].

### Potential ACMV and EACMV-UG miRNA Hairpins

The VMir Analyzer [35], [43] was used to identify novel miRNA hairpins encoded by ACMV and EACMV-UG. Each virus sequence (Table 1) was processed individually. Both viruses have a circular genome; therefore the options “circular” and “any” were chosen to display all hairpins in direct or reverse orientation, for conformation and orientation. To obtain the main hairpins, the results from each sequence were further filtered using VMir Viewer [43]. The filter values for “minimal score” and “window counts” were set to the most stringent parameters of 115 and 35 respectively [43]. After the completion of the prediction, the recorded hairpins were compared to one another and categorized to retain only the largest, i.e. the main hairpin [35]. Also the web interface Vir-Mir db (http://alk.ibms.sinica.edu.tw) [44] was searched for predicted viral hairpins.

### Classification of Hairpins and Prediction of Secondary Structures

The viral hairpins, obtained after filtering, were further screened with MiPred (http://www.bioinf.seu.edu.cn/miRNA) to distinguish real from pseudo pre-miRNA, using a hybrid feature, including local contiguous structure sequence composition, minimum of Minimum of Free Energy (MFE) of the secondary structure and *P*-value of randomization test [36]. For any pre-miRNA like hairpin the random forest-based classifier predicts whether it is a real pre-miRNA (MFE <−20 kcal/mol, *P*-value <0.05 and continuously paired nucleotides at high frequencies) or pseudo pre-miRNA (MFE >−20 kcal/mol, *P*-value >0.05 and continuously unpaired nucleotides at high frequencies [36], [45].

The secondary structures of hairpins grouped as real pre-miRNA were predicted using the RNAshapes [46].

### Prediction of Virus miRs/miRs* with Capacity to Bind *Jatropha* and Cassava ESTs as Targets

The ACMV and EACMV-UG real pre-miRNAs sequences with MFE <−20 kcal/mol, were compared to ESTs of *Jatropha* and cassava using BlastN with a sensitive setting of word length 7 [47]. Sequences of 17–24 bp with <5 mismatches located directly on the 5′ and 3′ arms of the hairpin structures were retained and used in RNAhybrid (http://bibiserv.techfak.unibielefeld.de/rnahybrid/submission.html) [48] and psRNATarget (http://plantgrn.noble.org/psRNATarget) [49] to identify complementary regions of predicted virus miRNAs in *Jatropha* and cassava.

The selection of targets of miRNAs by RNAhybrid considered the following parameters: a) at least 17 of 21 nucleotides should exhibit complementarity with their target sequence, b) the nucleotides 2–8 at the 5′ end should exhibit high sequence complementarity (only 1 mismatch allowed), c) any mismatch in the nucleotides 2–8 at the 5′ end should be compensated by strong binding beyond this region [19], d) end overhangs should not be more than 2 nucleotides, e) G:U base pairs should not be treated as mismatches [21], [50], f) the miRNA: target pair should have a low free energy of binding (maximum –20 kcal/mol). The latter criterion was used for miRNA target prediction in various plants [51]–[52]. To score the complementarity between miRNA and their target transcript, the default cut-off threshold 0–2.0 for lower false positive and 4.0–5.0 for higher prediction coverage were used in psRNATarget analyses [49]. miRNA sequences from the 5′ and 3′ arm were represented as miR and miR*, respectively.

### Plant miRs/miRs* with Capacity to Bind the ACMV and EACMV-UG Genome as a Target

The possible existence of plant miRNAs having the potential of binding to ACMV [Genbank: JN053423; JN053421] and EACMV-UG ORFs [Genbank: JN053454; JN053447] was investigated (Table 1). All currently known plant mature miRNAs as well as all available conserved and non-conserved *Euphorbiaceae* miRNAs were obtained from the miRBase, release 18 (http://www.mirbase.org). To avoid redundant or overlapping miRNAs, the non-redundant miRNA sequences were extracted as query sequences for the Blast search. A total of 1552 plant miR/miR* sequences were used to predict viral targets on ACMV and EACMV-UG by employing RNAhybrid and the psRNATarget [48]. The stringency parameters for RNAhybrid were set as follows: 3 hits per target, −25 kcal/mol energy cut-off and maximum 1 bulge or loop size per side. The same parameters as described above were used to select putative miR/miR* with the best hybridization sites on the genome for both viruses. To score the complementarity between miRNA and their target transcript, for psRNATarget, the default parameters were used as described above.

psRNATarget was further used to predict targets for the plant miRs/miRs* found to target the ACMV and EACMV-UG genome on the *Jatropha* and cassava ESTs.

### Virus Detection and RNA Extraction

Total RNA was extracted from young leaves of *Jatropha* and cassava using plant RNA purification reagents (Invitrogen) according to the supplier’s instructions. The quality and concentrations of total RNAs were determined using NanoVue Spectrophotometer (GE Healthcare Life Sciences) and gel electrophoresis.

Triple Antibody Sandwich Enzyme-Linked Immunosorbent Assay (ELISA) using commercially available kits (DSMZ GmbH, Germany) and PCR with primers JC6F and JC2R [11] were applied to detect the presence of ACMV and EACMV.

### Primer Design, Stem-loop RT-PCR and Sequencing

The sequences of all predicted virus and plant miRs were used to design specific forward and stem-loop RT primers according to criteria mentioned by [37]–[38].

MiRNA cDNA synthesis was performed on 2.5 µg of total RNA treated with DNase I (Roche) using SuperScript III Reverse Transcriptase (Invitrogen) in combination with the stem-loop RT primers (Table S1–S2 in File S1) according to [38]. No template reactions were included for each primer as -RT control. The plants B2C15 and S4J12 which were not virus infected based on ELISA and PCR tests were included as negative controls.

An end point PCR was performed in a total volume of 25 µl using 2.5 µl 10×PCR buffer (QIAGEN), 1 µl MgCl_2_ (25 mM), 0.5 µl dNTP (20 mM), 0.5 µl of each primer (10 pmol), 0.15 µl HotStarTaq Polymerase (QIAGEN HotStar Plus TM PCR) and 2.5 µl of reverse transcription product. The following primers were used for end point PCR: miRNA-specific forward primers (Table S1–S2 in File S1) and the universal miRNA reverse primer (GTGCAGGGTCCGAGGT) [38] and Actin primers (TGGTTCCACTATGTTCCCTGGTA and CTTCATGCTGCTTGGAGCAA) as an internal control [53]. The PCR cycling conditions were according to [38]. The PCR products were separated by electrophoresis on a 4% agarose gel.

To validate the sequence and the size of miRNAs, amplified PCR products were purified by QIAquick PCR Purification Kit (QIAGEN) and directly sequenced.

## Results

### Prediction of Novel pre-miRNA Hairpins from ACMV and EACMV-UG Genomes

**Figure 2:**
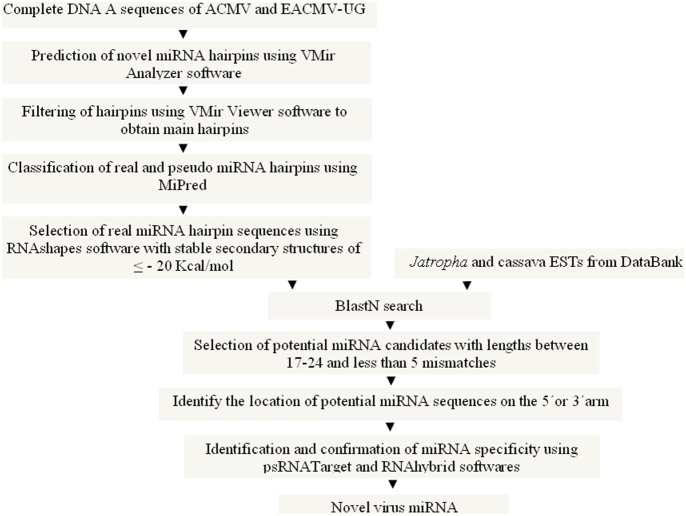
Outline of the strategy to identify miRNAs in viral DNA-A of ACMV and EACMV-UG and their potential targets in the host plants *Jatropha* and cassava. Computational approaches were used to scan and filter the DNA-A genomes of 11 ACMV and 28 EACMV-UG isolates to identify novel virus miRNAs and their targets in *Jatropha* and cassava.

Computational approaches were used for the first time to scan and filter the DNA-A genomes of 11 ACMV and 28 EACMV-UG isolates to identify novel pre-miRNA hairpins encoding miRNAs, which could target the *Jatropha* and cassava genome (Table 1). Figure 2 gives an overview of the strategies adopted for the search and prediction of novel virus (ACMV and EACMV-UG) miRNAs matching targets in their host plants *Jatropha* and cassava. As a result, a total of 14 different predicted pre-miRNA hairpin sequences (9 from ACMV and 5 from EACMV-UG) were obtained (Table 2).

**Table 2:** Fourteen viral pre-miRNAhairpins from 11 ACMV and 28 EACMV-UG isolates from *Jatropha*and cassava were classified as real or pseudo.

Pre-miRNA	No. of virus sequences used	Location	Position*	Real/pseudo	Hairpin sequence
**ACMV 1**	7/11	AC2	1350–1433	Real	AGCAATGAATGGCGTGTATACCTGGGAAATAAACAATCCCCTGTATTTCACAATCACCAGGCACCAACAACGACCATTCCTGCT
**ACMV 2**	4/11	AC4	2549–2614	Real	TTTGGGTATGTGAGAAAGACATTCTTGGCTTGAATTCAAAACGAGGAGTTCTCATGTTGACCAAG
**ACMV 3**	8/11	AC4	2442–2521	Real	GTTCTCCATTCTGATGCAGCTCTCTACAGATTTTAATGAACTTAGGGTTTGATGGGAGAGAGAGTGTTTGAAGGAAGGAC
**ACMV 4**	8/11	AV2	160–246	Real	TCCAGACTCGGTACATGGGCTTAGGTGTATGCTTGCAATTAAATATTTGCAGGCCTTAGAGGATACATACGAGCCCAGTACTTTGGG
**ACMV 5**	8/11	AC1	1645–1718	Real	TTCTTGCTTTTCCTCGTCTAGGAACTCTTTATAGGACGAGGTA xGGTCCTGGATTGCAGAGGAAGATAGTGGGAA
**ACMV 6**	6/11	AC1	1839–1941	Real	ACCAGGCAGCAATATGAGACCTTTGGACTAGGTCCAGGTGTCCACATAGGTAATTGTGTGGGCCTAAAGATCTGGCCCATATCGTCTTCCCTGTTCTGCT
**ACMV 7**	4/11	IR	2622–2704	Real	AGAATGCCATTTAGAGACACCTATATAATGTCTCCAATTAACAGGAGGACTTGCTCAAGAGTGTCTCTAGTTGAGTGTCTC
**ACMV 8**	3/11	IR	44–105	Real	GTATGTCGGCCAATCATGTTGTAGCTGTAAAAGTTATGTATTAGTGGTGGGCCACTATATAC
**ACMV 9**	5/11	AC2	1234–1310	Pseudo	CAGTCTGAGGCTGTAAGGTCGTCCAGATCTCGAAGTTGAGAAAACATTTGTGAATCCCCAGCGCCTTCCTCAGGTTG
**EACMV-UG 1**	25/28	AC2	1416–1500	Real	TTTCGAAATAGAGGGGATTTGTTATGTCCCAGGTAAAAACGCCATTCCTTGCTTGAGGCGCAGTGATGAGTTCCCCTGTGCGAGA
**EACMV-UG 2**	10/28	AC1	2208–2268	Real	GGAGGGCCAGCATTTAGCTCAGGTATATGCAGACGCGTTAAATGCTTCGTCTAAATCGAGGCTCTTC
**EACMV-UG 3**	24/28	AC2	1535–1618	Pseudo	CGAGCAGCCGCATTCGAGGTCGACCCGCCTACGTCGGACGGCCCTGGTCTTCGCTGTGCGGTGTTGGACTTTGATGGGCACTTG
**EACMV-UG 4**	9/28	IR	2675–2751	Pseudo	AAGTCTATAGCAATCGGTGGAATGGGGGGCAATATATATGATGTCCCCCAATGGCATATGTGTAAATAGGTAGACTT
**EACMV-UG 5**	9/28	IR	2628–2692	Pseudo	ACCGGCTCTTGGCATATTGGCTGTCGTTTTGGATCGGGGGACACTCAAAACTCCAGGGGAACGGT

The number of virus sequences, their locationin the virus genome and their hairpin sequences are shown.

*position based on [Genbank:JN053428 and JN053454].

One of the important features that distinguish miRNAs from other endogenous small RNAs is the ability of the pre-miRNA sequence to form a stem loop hairpin structure [29], [33]. It is also known that the secondary structure of pre-miRNAs is an essential feature for the computational identification of miRNAs [54]–[55]. Furthermore, the presence of potential pseudo hairpins makes the filtering of hairpins in the computational analysis necessary. For this purpose, the MiPred was used to show that 10 of the 14 different predicted pre-miRNAs were real, while 4 were pseudo miRNAs (Table 2).

The length of the real pre-miRNA hairpin sequences ranged from 62 bp (ACMV 8) to 100 bp for the longest hairpin (ACMV 6). Pseudo pre-miRNA harpins varied from 65 bp (EACMV-UG 5) to 84 bp (EACMV-UG 3) (Table S3 in File S1). The MFE ranged from −19 kcal/mol (ACMV 8) to −38.7 kcal/mol (ACMV 6) for real pre-miRNA hairpins, and from −20.5 kcal/mol (ACMV 9) to −34.4 kcal/mol (hairpin EACMV-UG 3) in the pseudo pre-miRNA harpins. Prediction confidence values ranged from 52.10% (ACMV 1) to 75.40% (ACMV 4) for real and from 50% (EACMV-UG 3) to 77% (ACMV 9) for pseudo pre-miRNA hairpins (Table S3 in File S1). Results clearly showed that most *P*-values were very low and all real pre-miRNA hairpins had *P*-values <0.03, although the real and pseudo pre-miRNAs had similar ranges of MFE, prediction confidence and sequence length. Within the pseudo pre-miRNAs only EACMV-UG 4 had a low *P*-value (<0.02). Comparing each of the basic MiPred structure units between real and pseudo pre-miRNA hairpins showed that the real pre-miRNA structures had continuously more paired nucleotides [“)))” or “(((“], than the pseudo pre-miRNAs with more unpaired structures [“…”] (Table S3 in File S1), as described by [38], [41]. Since the real hairpin ACMV 8 had a MFE >−20 kcal/mol, only 9 out of 10 real pre-miRNA hairpin sequences (ACMV 1–7 and EACMV-UG 1–2) were retained for further analyses.

In addition, a stable secondary structure is a functional prerequisite critical for early stages of the mature miRNA biogenesis by avoiding early degradation [27]. Nucleotides G and C contribute to the stabilization of the secondary structure of stem-loop hairpins [55], meaning that the ideal GC content of pre-miRNA should be between 24 and 71% [56]. The nucleotide composition of the real viral pre-miRNA hairpins showed that hairpins ACMV 7 and EACMV-UG 1 had the highest content of A+T (59.26 and 51.76%, respectively) and ACMV 5 and EACMV-UG 2 had the highest C+G content (45.95 and 49.25%, respectively) (Table 3).

**Table 3:** Nucleotide content of 7 real ACMV and 2 real EACMV-UG miRNA hairpins.

Virus	Pre-miRNA	A%	C%	G%	T%	A+T%	C+G%
ACMV	ACMV 1	33.33	28.57	16.67	21.43	54.76	45.24
	ACMV 2	29.23	13.85	26.15	30.77	60	40
	ACMV 3	26.25	13.75	28.75	31.25	57.5	42.5
	ACMV 4	25.29	19.54	26.44	28.74	47	40
	ACMV 5	22.97	16.22	29.73	31.08	54.05	45.95
	ACMV 6	22	25	25	28	50	50
	ACMV 7	29.63	19.75	20.99	29.63	59.26	40.74
EACMV-UG	EACMV-UG 1	23.53	20	28.24	28.24	51.76	48.24
	EACMV-UG 2	23.88	22.39	26.87	26.87	50.75	49.25

**Figure 3:**
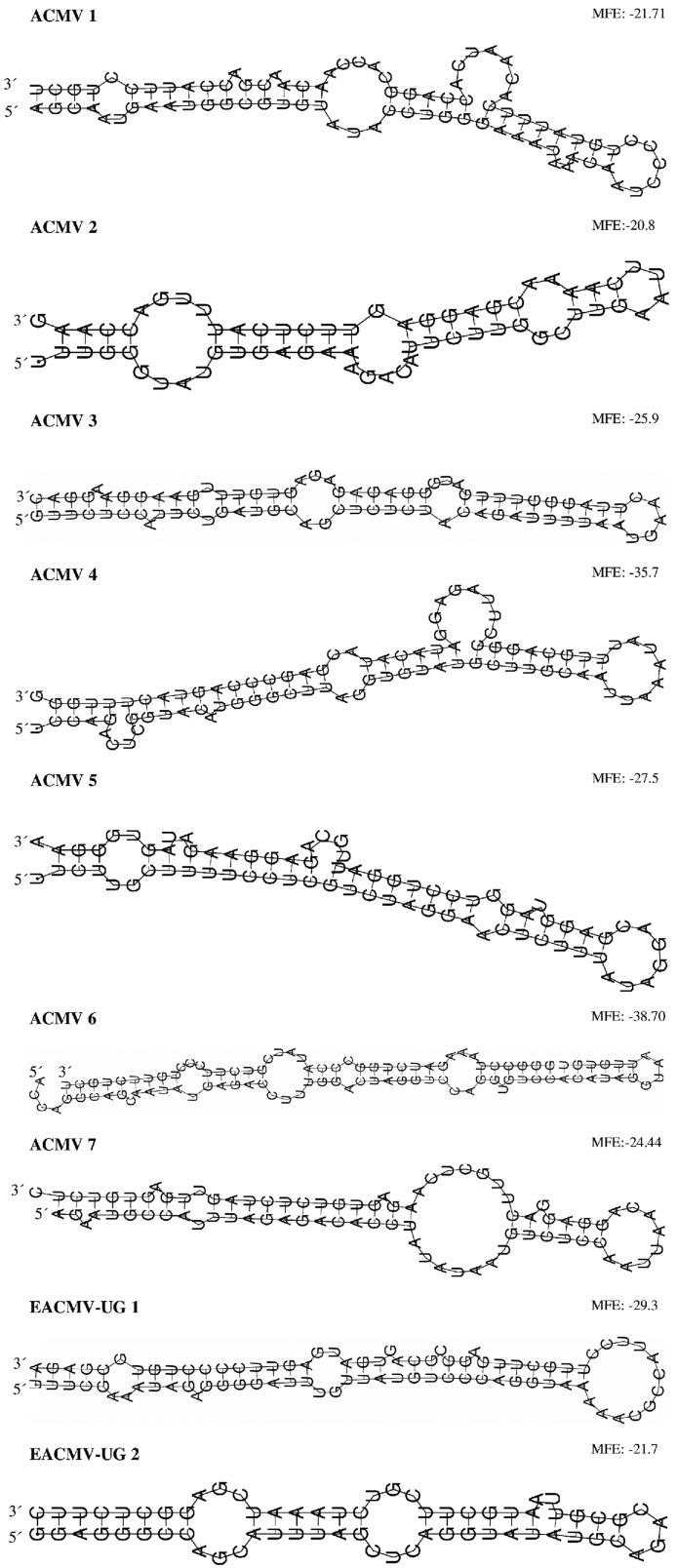
Secondary structures of 9 predicted real viral pre-miRNA hairpins. The secondary structures of the real pre-miRNAs were folded using the RNAshapes.

The localization of pre-miRNA hairpins in the ACMV ORFs showed that AC1, AC2, AC4 and IR encoded 2 pre-miRNA hairpins each, while AV2 encoded 1 pre-miRNA hairpin (Table 2). The pre-miRNA hairpins ACMV 3–5 were encoded by the highest number of ACMV sequences (8 out of 11, 72%, Table 2). In EACMV-UG, 1 pre-miRNA hairpin was encoded by AC1, while 2 were encoded by AC2 and the IR (Table 2). The pre-miRNA EACMV-UG 1 was predicted by the highest number of EACMV-UG sequences (25 out of 28, 89%, Table 2). For both viruses no pre-miRNA hairpin could be located in AC3 and AV1 (Table 2) and interestingly no similar hairpins could be found. The secondary structures of 9 real pre-miRNA hairpin sequences predicted by RNAShapes (Figure 3) confirmed the data obtained by MiPred (Table S3 in File S1).

Searching the Vir-Mir db [44] for virus hairpins, only 3 deposited EACMV sequences were retrieved. One of the EACMV sequences (ID 18042) predicted from [Genbank: NC-004674.1] [44] was similar to the hairpin EACMV-UG 3 found in this study (Table 2).

### Virus miRs/miRs* Location on Secondary Structures

The lack of information on miRNAs encoded by plant viruses and incomplete genome information from *Jatropha* and cassava, made it a challenge to locate candidate miRNAs on the hairpin sequences. Nine real pre-miRNA hairpin sequences were compared with the *Jatropha* and cassava ESTs using BlastN. A total of 980 *Jatropha* and 1240 cassava sequence hits between 12–36 bp were obtained and carefully allocated on the secondary structures of predicted hairpins using RNAShapes. Excluding those that fell directly on the stem loop, 111 *Jatropha* and cassava sequences of 18–24 bp located on the 5′ or 3′ arms of the secondary pre-miRNA harpins were classified as putative virus miRNA/miRNA* candidates (Table 4). 49 out of 111 predicted mature miRNA sequences were located on the 5′ arm, and 62 on the 3′ arm of pre-miRNA hairpins (Table 4). 84 out of 111 miRs/miRs* corresponded to ACMV and 27 to EACMV-UG (Table 4).

**Table 4:** The novel virus miR/miR* sequences predicted from ACMV and EACMV-UG real pre-miRNA hairpins.

Pre-miRNA hairpins	miR/miR*	miR/miR*sequences	miR/miR* length	Location of in 5′ or 3′ arm	Nucleotides 2–8 at the 5′ end
**ACMV 1**	ACMV-mir-1–1	AGCAAUGAAUGGCGUGUAUACCUG	24	5′	**GCAAUGA**
	ACMV-mir-1–2	AGCAAUGAAUGGCGUGUAUA	20	5′	**GCAAUGA**
	ACMV-mir-1–3	CAAUGAAUGGCGUGUAUACCUG	22	5′	AAUGAAU
	ACMV-mir-1–4	AUGAAUGGCGUGUAUACCUGGG	22	5′	UGAAUGG
	ACMV-mir-1–5	UGAAUGGCGUGUAUACCUGGGAA	23	5′	GAAUGGC
	ACMV-mir-1–6	AUGGCGUGUAUACCUGGGAAAUA	23	5′	UGGCGUG
	ACMV-mir-1–7	UGUAUACCUGGGAAAUAAACA	21	5′	**GUAUACC**
	ACMV-mir-1–8	UGUAUACCUGGGAAAUAAAC	20	5′	**GUAUACC**
	ACMV-mir-1–9*	CCAGGCACCAACAACGACCAU	21	3′	**CAGGCAC**
	ACMV-mir-1–10*	CCAGGCACCAACAACGACCAUUC	23	3′	**CAGGCAC**
	ACMV-mir-1–11*	CCAGGCACCAACAACGACCAUUCC	24	3′	**CAGGCAC**
	ACMV-mir-1–12*	CAGGCACCAACAACGACCAUUCCU	24	3′	AGGCACC
	ACMV-mir-1–13*	GGCACCAACAACGACCAUUCCUGC	24	3′	GCACCAA
	ACMV-mir-1–14*	CCAACAACGACCAUUCCUGC	20	3′	CAACAAC
**ACMV 2**	ACMV-mir-2–1	UUUGGGUAUGUGAGAAAGAC	20	5′	UUGGGUA
	ACMV-mir-2–2	UUGGGUAUGUGAGAAAGACAUU	22	5′	UGGGUAU
	ACMV-mir-2–3	UGGGUAUGUGAGAAAGACAUUCUU	24	5′	GGGUAUG
	ACMV-mir-2–4	GGUAUGUGAGAAAGACAUUCUUGG	24	5′	GUAUGUG
	ACMV-mir-2–5	GUAUGUGAGAAAGACAUUCUUGG	23	5′	UAUGUGA
	ACMV-mir-2–6	AUGUGAGAAAGACAUUCUUGGCUU	24	5′	UGUGAGA
	ACMV-mir-2–7	UGUGAGAAAGACAUUCUUGGCUUG	24	5′	GUGAGAA
	ACMV-mir-2–8*	CAAAACGAGGAGUUCUCAUUUGA	23	3′	AAAACGA
**ACMV 3**	ACMV-mir-3–1	GAUGCAGCUCUCUACAGAUUU	21	5′	AUGCAGC
	ACMV-mir-3–2	UUCUCCAUUCUGAUGCAGCUCU	22	5′	UCUCCAU
	ACMV-mir-3–3	UCUCCAUUCUGAUGCAGCUCUA	22	5′	CUCCAUU
	ACMV-mir-3–4*	UUAGGGUUUGAUGGGAGAGAGAG	23	3′	UAGGGUU
	ACMV-mir-3–5*	UAGGGUUUGAUGGGAGAGAGAGUG	24	3′	AGGGUUU
	ACMV-mir-3–6*	AGGGUUUGAUGGGAGAGAGAGUGU	24	3′	GGGUUUG
	ACMV-mir-3–7*	GGGUUUGAUGGGAGAGAGAGUGUU	24	3′	GGUUUGA
	ACMV-mir-3–8*	GGUUUGAUGGGAGAGAGAGUGUUU	24	3′	GUUUGAU
	ACMV-mir-3–9*	GUUUGAUGGGAGAGAGAGUGUUUG	24	3′	UUUGAUG
	ACMV-mir-3–10*	UUUGAUGGGAGAGAGAGUGUUUG	23	3′	**UUGAUGG**
	ACMV-mir-3–11*	UUUGAUGGGAGAGAGAGUGUUUGA	24	3′	**UUGAUGG**
	ACMV-mir-3–12*	UUGAUGGGAGAGAGAGUGUUUGAA	24	3′	UGAUGGG
	ACMV-mir-3–13*	UGAUGGGAGAGAGAGUGUUUGAAG	24	3′	GAUGGGA
	ACMV-mir-3–14*	GAUGGGAGAGAGAGUGUUUGA	21	3′	AUGGGAG
	ACMV-mir-3–15*	UGGGAGAGAGAGUGUUUGAAGGAA	24	3′	GGGAGAG
	ACMV-mir-3–16*	GGGAGAGAGAGUGUUUGAAGGAAG	24	3′	GGAGAGA
	ACMV-mir-3–17*	GGAGAGAGAGUGUUUGAAGGAAGG	24	3′	**GAGAGAG**
	ACMV-mir-3–18*	AGAGAGAGUGUUUGAAGGAAGGA	23	3′	**GAGAGAG**
	ACMV-mir-3–19*	AGAGAGAGUGUUUGAAGGAAGGAC	24	3′	**GAGAGAG**
**ACMV 4**	ACMV-mir-4–1	GGUACAUGGGCUUAGGUGUAUGCU	24	5′	GUACAUG
	ACMV-mir-4–2	ACAUGGGCUUAGGUGUAUGCUUGC	24	5′	CAUGGGC
	ACMV-mir-4–3	CAUGGGCUUAGGUGUAUGCUUGCA	24	5′	AUGGGCU
	ACMV-mir-4–4	UGGGCUUAGGUGUAUGCUUGCAA	23	5′	GGGCUUA
	ACMV-mir-4–5	GCUUAGGUGUAUGCUUGCAA	20	5′	CUUAGGU
	ACMV-mir-4–6*	UACAUACGAGCCCAGUACUUUGG	23	3′	ACAUACG
	ACMV-mir-4–7*	AUACGAGCCCAGUACUUUGG	20	3′	UACGAGC
					
**ACMV 5**	ACMV-mir-5–1	UCUUGCUUUUCCUCGUCUAGGAA	23	5′	**CUUGCUU**
	ACMV-mir-5–2	UCUUGCUUUUCCUCGUCUAGGAAC	24	5′	**CUUGCUU**
	ACMV-mir-5–3	CUUGCUUUUCCUCGUCUAGGAACU	24	5′	UUGCUUU
	ACMV-mir-5–4	UUGCUUUUCCUCGUCUAGGAACUC	24	5′	UGCUUUU
	ACMV-mir-5–5	CUUUUCCUCGUCUAGGAACUCU	22	5′	**UUUUCCU**
	ACMV-mir-5–6	CUUUUCCUCGUCUAGGAACUCUU	23	5′	**UUUUCCU**
	ACMV-mir-5–7*	GAGGUAGGUCCUGGAUUGCAGAGG	24	3′	AGGUAGG
	ACMV-mir-5–8*	GUAGGUCCUGGAUUGCAGAGGAA	23	3′	UAGGUCC
	ACMV-mir-5–9*	AGGUCCUGGAUUGCAGAGGAAGA	23	3′	GGUCCUG
	ACMV-mir-5–10*	GGUCCUGGAUUGCAGAGGAAGAU	23	3′	GUCCUGG
	ACMV-mir-5–11*	CUGGAUUGCAGAGGAAGAUAGUG	23	3′	UGGAUUG
	ACMV-mir-5–12*	UGGAUUGCAGAGGAAGAUAGUGGG	24	3′	GGAUUGC
	ACMV-mir-5–13*	GGAUUGCAGAGGAAGAUAGUGGGA	24	3′	GAUUGCA
	ACMV-mir-5–14*	GAUUGCAGAGGAAGAUAGUGGGA	23	3′	**AUUGCAG**
	ACMV-mir-5–15*	GAUUGCAGAGGAAGAUAGUGGGAA	24	3′	**AUUGCAG**
**ACMV 6**	ACMV-mir-6–1	AGGCAGCAAUAUGAGACCUUU	21	5′	GGCAGCA
	ACMV-mir-6–2	GGCAGCAAUAUGAGACCUUUGGAC	24	5′	GCAGCAA
	ACMV-mir-6–3	AGCAAUAUGAGACCUUUGGACUAG	24	5′	GCAAUAU
	ACMV-mir-6–4	AUGAGACCUUUGGACUAGGUCCA	23	5′	UGAGACC
	ACMV-mir-6–5	CUUUGGACUAGGUCCAGGUGUCCA	24	5′	UUUGGAC
	ACMV-mir-6–6	GACUAGGUCCAGGUGUCCACAUAG	24	5′	ACUAGGU
	ACMV-mir-6–7*	UUGUGUGGGCCUAAAGAUCU	20	3′	UGUGUGG
	ACMV-mir-6–8*	UGUGGGCCUAAAGAUCUGGCCCAU	24	3′	GUGGGCC
	ACMV-mir-6–9*	CCUAAAGAUCUGGCCCAUAUCGUC	24	3′	CUAAAGA
	ACMV-mir- 6–10*	AAGAUCUGGCCCAUAUCGUCUUCC	24	3′	AGAUCUG
	ACMV-mir-6–11*	AGAUCUGGCCCAUAUCGUCUUC	22	3′	GAUCUGG
	ACMV-mir-6–12*	GAUCUGGCCCAUAUCGUCU	19	3′	AUCUGGC
	ACMV-mir-6–13*	UCUGGCCCAUAUCGUCUUCCCU	22	3′	CUGGCCC
	ACMV-mir-6–14*	UGGCCCAUAUCGUCUUCCCUG	21	3′	GGCCCAU
	ACMV-mir-6–15*	GCCCAUAUCGUCUUCCCUGUUCUG	24	3′	CCCAUAU
	ACMV-mir-6–16*	CCAUAUCGUCUUCCCUGUUCUGCU	24	3′	CAUAUCG
	ACMV-mir-6–17*	CAUAUCGUCUUCCCUGUUCUG	21	3′	AUAUCGU
	ACMV-mir-6–18*	UCGUCUUCCCUGUUCUGCU	19	3′	CGUCUUC
**ACMV 7**	ACMV-mir-7–1	AGAAUGCCAUUUAGAGACACCU	22	5′	GAAUGCC
	ACMV-mir-7–2*	AGAGUGUCUCUAGUUGAGUGUCU	23	3′	GAGUGUC
	ACMV-mir-7–3*	AGUGUCUCUAGUUGAGUGUCU	21	3′	GUGUCUC
**EACMV-UG 1**	EACMV-UG-mir-1–1	UUUCGAAAUAGAGGGGAUUUGUUA	24	5′	UUCGAAA
	EACMV-UG-mir-1–2	UCGAAAUAGAGGGGAUUUGUUAUG	24	5′	**CGAAAUA**
	EACMV-UG-mir-1–3	UCGAAAUAGAGGGGAUUUGUUAU	23	5′	**CGAAAUA**
	EACMV-UG-mir-1–4	CGAAAUAGAGGGGAUUUGUUAU	22	5′	GAAAUAG
	EACMV-UG-mir-1–5	GAAAUAGAGGGGAUUUGUUAUGU	23	5′	AAAUAGA
	EACMV-UG-mir-1–6	AAAUAGAGGGGAUUUGUUAUGUC	23	5′	AAUAGAG
	EACMV-UG-mir-1–7	AAUAGAGGGGAUUUGUUAUGUC	22	5′	AUAGAGG
	EACMV-UG-mir-1–8	AUAGAGGGGAUUUGUUAUGUCCCA	24	5′	UAGAGGG
	EACMV-UG-mir-1–9	AGAGGGGAUUUGUUAUGUCC	20	5′	GAGGGGA
	EACMV-UG-mir-1–10	GGGAUUUGUUAUGUCCCAGGUAA	23	5′	GGAUUUG
	EACMV-UG-mir-1–11	AUUUGUUAUGUCCCAGGUAA	20	5′	UUUGUUA
	EACMV-UG-mir-1-l2*	UUGCUUGAGGCGCAGUGAUGAGUU	24	3′	UGCUUGA
	EACMV-UG-mir-1–13*	UGCUUGAGGCGCAGUGAUGAGUUC	24	3′	GCUUGAG
	EACMV-UG-mir-1–14*	GCUUGAGGCGCAGUGAUGAGUUCC	24	3′	**CUUGAGG**
	EACMV-UG-mir-1–15*	GCUUGAGGCGCAGUGAUGAG	20	3′	**CUUGAGG**
	EACMV-UG-mir-1–16*	CUUGAGGCGCAGUGAUGAGUUCCC	24	3′	UUGAGGC
	EACMV-UG-mir-1–17*	GAGGCGCAGUGAUGAGUUCCCCUG	24	3′	AGGCGCA
	EACMV-UG-mir-1–18*	AGGCGCAGUGAUGAGUUCCCCU	22	3′	GGCGCAG
	EACMV-UG-mir-1–19*	AGUGAUGAGUUCCCCUGUGCGAGA	24	3′	GUGAUGA
**EACMV-UG 2**	EACMV-UG-mir-2–1	CAGCAUUUAGCUCAGGUAUAU	21	5′	AGCAUUU
	EACMV-UG-mir-2–2	AGGGCCAGCAUUUAGCUCAGGU	22	5′	GGGCCAG
	EACMV-UG-mir-2–3*	UAAUGCUUCGUCUAAAUCGAGG	22	3′	GAGCUAA
	EACMV-UG-mir-2–4*	UGCUUCGUCUAAAUCGAGGCU	21	3′	GCUUCGU
	EACMV-UG-mir-2–5*	GCUUCGUCUAAAUCGAGGCUC	21	3′	CUUCGUC
	EACMV-UG-mir-2–6*	UUCGUCUAAAUCGAGGCUCUUC	22	3′	UCGUCUA
	EACMV-UG-mir-2–7*	UCGGAGCUAAAUCUGCUUCGU	20	3′	CGGAGCU
	EACMV-UG-mir-2–8*	GCGUUAAUGCUUCGUCUA	18	3′	CGUUAAU

The miR/miRs* were predicted using *Jatropha* and cassava sequence hits from BlastN. The length, location on 5′ or 3′ arms of hairpins and sequence of the nucleotides 2–8 at the 5′ end (highlighted in bold) using RNAShape are shown.

Some miRNA sequences exhibited variations in a few nucleotides at the 3′ or 5′ end, leading to the production of multiple mature variants, which therefore were referred to as isomiRs (Table 4). In some case, the predicted isomiRs even shared a common region (nucleotides 2–8 at the 5′ end) and could bind to the same targets, for instance ACMV-mir-3–17* to 3–19*, located on AC4, all targeted the ring finger protein family [Genbank: GW876074]. Cloonan et al. [57] reported that isomiRs are biologically relevant and target pathways of functionally related genes. ACMV-mir-5–1 and 5–2, located on AC2, targeted an AT-rich interactive domain [Genbank: GW878601]. ACMV-mir-5–5 and 5–6 targeted phospholipase D [Genbank: GW613466], ACMV-mir-5–14 and 5–15 targeted a cysteine protease inhibitor [Genbank: GT976828]. ECMV-UG-mir 1–2 and 1–3, located on AC2 targeted serine/threonine protein kinase (Table 4, Table S4–S5 in File S1). All isomiR groups with the same region at the 5′ end (nucleotides 2–8) shared the same targets, only for ACMV mir-1–1 and 1–2, ACMV mir-3–10* and 3–11* and EACMV-UG 1–14* and 1–15* no common target was found.

### Virus miRs/miRs* with Putative Targets in the *Jatropha* Genome

Putative target genes of *Jatropha* were predicted for the 111 miR/miR* by RNAhybrid and psRNATarget. Although psRNATarget is important for providing scores for target selection [49], it could not predict targets for all the virus miRNA, when compared to RNAhybrid. In fact, RNAhybrid provides the MFE, based on the use of free energy of mRNA:miRNA hybridization, as alternative feature for target selection [50]. This is particularly important for virus target prediction, since viruses evolve very fast and are typically highly adapted to specific hosts [58] and not all miRNA target sites adhere to nucleotides 2–8 at the 5′ end complementarity [59]. In addition, to gain better understanding of the functional role of predicted miRNAs, the obtained targets were annotated by BlastX and UniProt (www.uniprot.org) Gene Ontology (GO) using the domain “molecular function” describing the elemental activities of a gene product at the molecular level, such as binding or catalysis (Table S4–S5 in File S1).

Based on the RNAhybrid analyses, a total of 234 targets were predicted for 78 ACMV- and 27 EACMV-UG miRs/miRs*. For ACMV-miR-1–11*, 3–17*, 5–7*, 4–2, 5–9*, 6–4 and 6–15* no target was predicted. The different miRNA targets were grouped into 9 molecular functions, where 83 (35.5%) of targets possess binding functions, 68 (29%) showed catalytic activity, while 43 (18.4%) were proteins with unknown molecular functions. In addition, 9 (3.9%) predicted targets were involved in enzyme regulator activity, 13 (5.6%) had structural molecule activity, 5 (2.1%) electron carrier activity, 5 (2.1%) transport activity, 5 (2.1%) nucleic acid binding transcription factor activity, 2 (0.85%) nutrient reservoir activity and 1 (0.43%) molecular transducer activity (Table S4 in File S1).

Using psRNATarget, 621 targets were predicted for 79 ACMV- and 26 EACMV-UG-miRs/miRs* (Table S5 in File S1). For ACMV-miR-2–3, 42, 5–7*, 6–12*, 6–18* and EACMV-UG miR 6–3 no targets were predicted. 260 targets (41.9%) showed binding activity and 163 (26.2%) catalytic activity, while 94 (15.1%) were proteins with unknown molecular functions. 17 targets (2.7%) possess nucleic acid binding transcription factor activity and 9 (1.5%) showed electron carrier activity. 21 targets (3.4%) showed enzyme regulator activity, 27 (4.4%) structural molecule activity, 20 (3.2%) transporter activity, 2 (0.03%) molecular transducer activity and 8 (1.3%) nutrient reservoir activity (Table S4 in File S1).

Out of the 621 predicted targets, 4 targets (2 binding and two with unknown molecular functions) had a score of 1 (0.64%). A score of 1.5 was obtained for 2 targets (one enzyme with regulatory and one with binding function) (0.32%). Further, a score of 2 for 10 targets (6 with binding, 3 with catalytic activity and one with unknown function (1.6%), a score of 2.5 for 41 targets (6.6%), while a score of 3 was predicted for 56 targets (9.01%) and a score of 3.5 was obtained in 102 targets (16.42%). The highest number of 162 targets (26.1%) had a score of 4. Further scores of 4.5 and 5 were obtained for 129 targets (20.8%) and 115 targets (18.5%) respectively (Table S5 in File S1).

Both programs produced 107 identical *Jatropha* ESTs targets for ACMV miRNAs and 30 for EACMV-UG miRNAs.

### Virus miRs/miRs* with Putative Targets in the Cassava Genome

Putative target genes for the 111 miRs/miRs* were predicted and annotated in the cassava ESTs from the GenBank as described for *Jatropha*.

Based on the RNAhybrid analyses, 84 ACMV- and 27 EACMV-UG-miRs/miRs* targeted 370 cassava ESTs. The different miRNA targets were assigned to 8 groups of molecular functions, of which 172 targets (46.5%) with molecular functions as binding, 67 (18.1%) with catalytic activity, 35 targets (9.4%) represent structural proteins, while 60 targets (16.2%) were proteins with unknown functions. Further, 5 targets (1.3%) showed nucleic acid binding/transcription factor activity, 4 targets (1.08%) belong to enzymes with regulator activity, 15 (4.05%) transporter activity and 11 (2.97%) electron carrier activity. The nutrient reservoir activity protein was targeted only once (0.27%) by ACMV-mir-6–3 (Table S6 in File S1).

Analyses with psRNATarget revealed that 81 ACMV- and 26 EACMV-UG-miRs/miRs*, targeted 688 cassava ESTs (Table S7 in File S1). For ACMV-mir-1–10*, 3–10*, 6–12* and 6–18* no target was found. The highest number of 361 targets (52.5%) showed molecular functions as binding, 125 (18.2%) with catalytic activity, 54 targets (7.9%) had structural molecule activity and 89 (12.9%) were proteins with unknown functions. Two proteins (0.29%) with nutrient reservoir activity were targeted by ACMV-mir-3–16* and ACMV-mir-6–3, while ACMV-mir-4–1 targeted a molecular transducer activity protein. In addition, 7 targets (1.01%) were predicted with enzyme regulator activity, 27 (3.9%) with transporter activity, 12 (1.7%) with electron carrier activity and 10 (1.45%) with nucleic acid binding transcription factor activity (Table S7 in File S1).

In addition, data showed that reticulum-3 protein was targeted by ACMV-mir-5–14* and 5–15*) with a score of 1 (0.29%), while 7 targets with binding function (1.02%) had a score of 1.5. A score of 2 was obtained by 12 targets (1.7%), a score of 2.5 by 36 targets (5.2%), and a score of 3 by 84 targets (12.2%). For 128 targets (18.63%) a score of 3.5 was obtained. The highest number of 158 targets (22.99%) reached a score of 4, while 143 had a score of 4.5 (20.8%) followed by 118 (17.17%) with a score of 5 (Table S7 in File S1).

Both programs produced 222 identical cassava ESTs targets for ACMV miRNAs and 54 for EACMV-UG miRNAs.

### Plant miRs/miRs* with Putative Targets on DNA-A of ACMV and EACMV-UG

**Figure 4:**
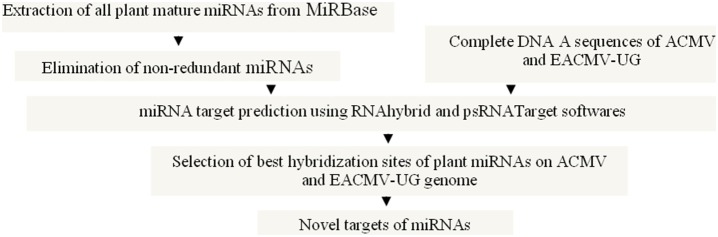
Outline of the strategy to identify plant miRNAs from miRBase that potentially target regions of DNA-A in ACMV and EACMV-UG. Computational approaches were identified plant miRs/miRs* with potential to target viral genomic regions.

The approach used to search plant miRNA targets in DNA A of ACMV and EACMV-UG is shown in Figure 4.

#### Plant miRs/miRs* with putative targets on DNA-A of ACMV

RNAhybrid analyses of all plant miRNAs from the miRBase Database (release18) revealed 24 miR/miR* sequences of 20 miR/miR* families, having putative targets in the DNA-A of ACMV (Table S8 in File S1). 18 of the miR/miR* sequences (14 miRs and 4 miRs*) had binding sites in AC1. The family miR164 (a, c, d) was found to be targeting the same position, in a single nucleotide. This also occurred in miR169aa and miR169* as well as miR1107 and miR1117, which were located in an overlapping region of AC1 and AC4. Furthermore, miR2094-3p and miR2668 shared binding sites in AC1 and AC4. AC3 was targeted by 3 miR sequences (miR397a, miR397b and miR841c), AV1 by 2 miR sequences (miR160a and miR1864), and AV2 only by miR2640a, while no miR/miR* sequence was found to have a significant complementarity with AC2 and IR (Table S8 in File S1).

psRNATarget analyses showed a total of 14 miR families containing 15 miR sequences, with targets in the DNA-A of ACMV (Table S9 in File S1). Both miR159a and miR159b shared overlapping binding sites in AC1 and AC4 at position 2242. Our data also showed that AC1 was targeted by miR395b, miR868 and miR4243 at positions 1786, 1687 and 2008, respectively. The overlapping ORFs AC2 and AC3 were targeted by miR397b at position 1095. The miR4246 also targeted the AC3 at position 1065. AV1 was targeted by 6 miRs and AV2 by two miRs. However, no miR sequence was found to target the IR (Table S9 in File S1). Our analyses also revealed that each program detected different targets at different positions; however miR397b and miR841 were located on similar targets by both programs.

#### Plant miRs/miRs* with putative targets on DNA-A of EACMV UG

RNAhybrid identified 23 different miR/miR* families containing 27 miR/miR* sequences, with putative targets in DNA-A of EACMV-UG. 22 miR/miR* sequences were miRs and 6 miRs* (Table S8 in File S1). AC1 was targeted by 11 miR/miR* sequences (3 conserved and 8 non-conserved miRNAs (miR156e*, miR156g, miR166h* and miR472*, miR868*, miR1118, miR1311, miR1510b, miR2119, miR4390, miR4409, respectively; Table S8 in File S1), while AC4 only by non-conserved miR1118. The miR171 family was present as 4 members (miR171a, b, d, and f) with differences in nucleotide composition, all targeting AC2 and AC3, and overlapping at positions 1364 or 1366. In addition, AC2 and AC3 were targeted by miR859, miR1111, miR1520j and miR2104. AV1, AV2 and IR were targeted by 3 (miR160f*, miR1082b, miR1446a), 2 (miR399c*, miR2927) and 4 (miR478a, miR482, miR2119, miR3633b) miR/miR* sequences, respectively (Table S8 in File S1). The miR2119 targeted two different positions (1791 and 65) in AC1 and IR respectively, indicating that multiplicity of miRNA targets within the viral genome is possible.

psRNATarget showed that 25 different miRs containing 22 miR families targeted DNA-A of EACMV-UG (Table S9 in File S1). AC1 and AC2 were targeted at position 1580 by miR2588a and b. AC1 was further targeted by 7 miRs at different positions. AC2 and AC3 were targeted by 4 sequences of conserved miR171 (a, b, d, f) family and non-conserved miR859. AC4 was targeted by miR2668 at position 236. AV1 was targeted by 6 miRs, while AV2 and IR were each targeted by 2 non-conserved miRs (miR859, miR4232 and miR4238, miR4379, respectively) (Table S9 in File S1).

Both programs produced identical results for miR171 (a, b, d and f) and miR859, which targeted both AC2 and AC3, and for two miRs (miR472, miR4390) and miR1446a that targeted AC1 and AV1 respectively (Table S8–S9 in File S1).

#### Plant miR/miR* location on Jatropha and cassava ESTs

Analyses by psRNATarget revealed the location of 72 plant miR/miR* sequences in *Jatropha* ESTs (Table S10 in File S1). MiR164a, miR164c and miR164d (Table S10 in File S1) with a score of 1.5, all located in the NAC domain [Genbank: GT978826], a validated target for miR164 [60]. The four miR 171 (a, b, d and f) had different targets (Table S10 in File S1). However, miR 159 (a, b, c), with a score of 2.5 and miR 319c targeted the same position on CSD (Copper/Zinc Superoxide Dismutase 2) responsible for abiotic stress. miR164a, c and d located in *Jatropha* ESTs targeted the NAC domain, which controls leaf development and determines the pattern of leaves [61].

69 different plant miR/miR* sequences were successfully localized in cassava ESTs (Table S11 in File S1). MiR156g located in the squamosa promoter-binding protein [Genbank: DV456109] while miR164d targeted the WRKY transcription factor [Genbank: DR085222]. Both miR169aa and miR169* targeted a nuclear transcription factor Y subunit A-1 [Genbank: DV443290, Genbank: DV455197, Genbank: DV445967] while miR397a and miR397b targeted laccase [Genbank: DR087678] (Table S11 in File S1).

### Detection of Virus and Plant miRNA

To validate the predicted miRNAs (Table 4, Table S8–S9 in File S1), stem-loop RT-PCR was performed, using infected and non-infected *Jatropha* and cassava plant, which were selected based on ELISA and PCR. Based on ELISA results, both plants S2C6 and S4C6 were highly co-infected with ACMV and EACMV (data not shown). In addition, PCR detected even low infections with selected primers [57], which showed that all selected plants except S4J12 and B2C15 were infected by geminiviruses, using as negative controls for *Jatropha* and cassava respectively (data not shown). The isolate from plant S4C6 previously sequenced [Genbank: JN053458] was determined to correspond to EACMV-UG [11].

**Figure 5:**
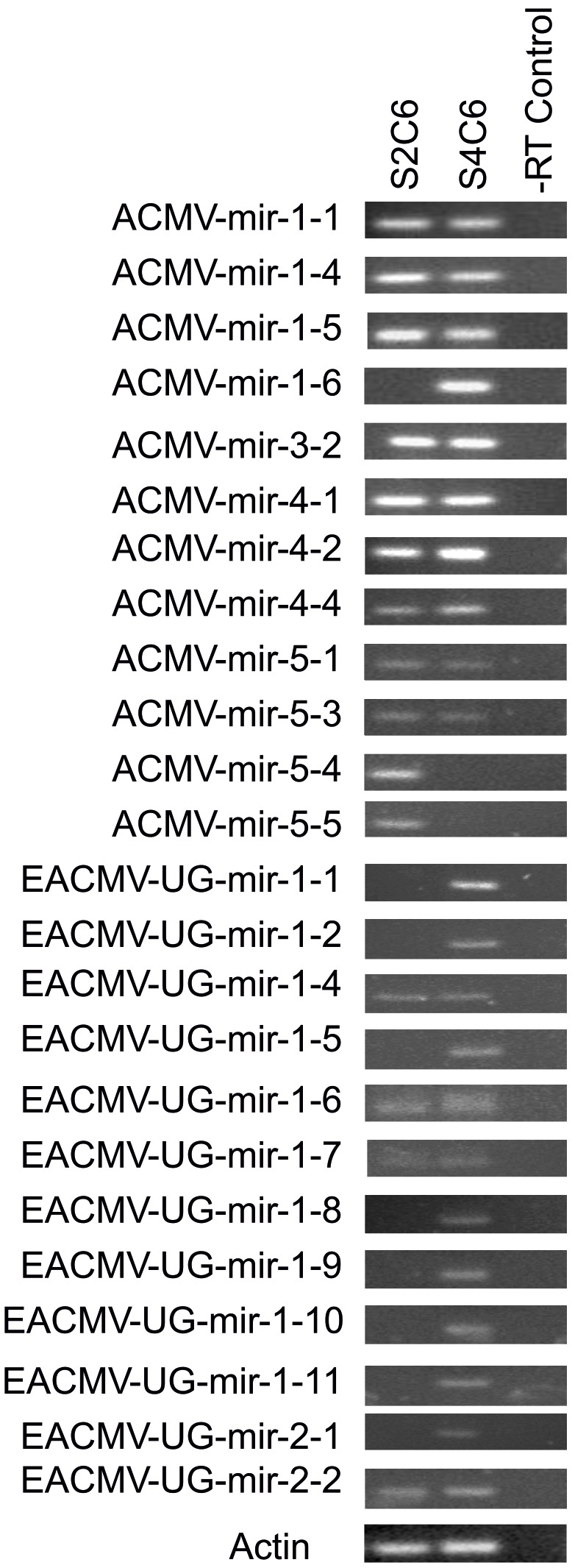
End point PCR amplification of ACMV and EACMV-UG virus miRNAs. PCR products of 60-infected with ACMV and EACMV: S2C6, S4C6, –RT control. Actin (76 bp) was used as internal control.

End point PCR showed that 12/27 ACMV miRs and all EACMV-UG miRs could be amplified from at least one of the infected plants with different expression levels (Figure 5, Table S12 in File S1). Some virus miRNA could not be detected in both infected S2C6 and S4C6 plants. It is possible that some miRNA may accumulate at lower levels, which make their detection difficult [62]–[63]. On the other hand, due to the restricted availability of infected plant material, it was not possible to detect all predicted miRNAs.

**Figure 6:**
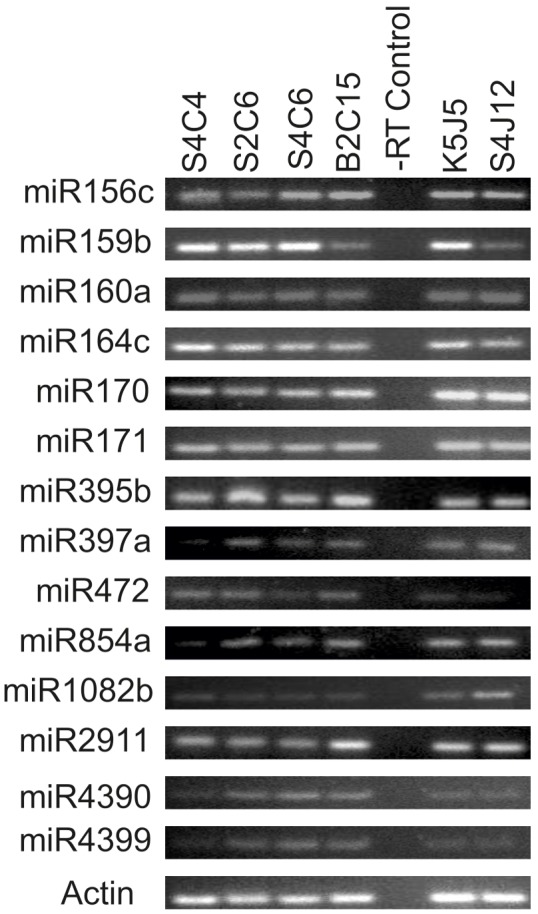
End point PCR amplification of plant miRNAs on cassava and *Jatropha*. PCR products of 60-infected cassava plant samples, respectively: S4C4, S2C6, S4C6, B2C15, –RT control. Lanes 6–7 are one infected and one non-infected *Jatropha* plant samples, respectively: K5J5, S4J12. *Actin* (76 bp) was used as internal control.

14 out of 55 plant miRs were detected with different expression levels using the stem-loop RT primers in all virus-infected plants and the non**-**infected controls (Figure 6, Table S13 in File S1). Sequencing of PCR products confirmed size and sequence identity of miRNAs.

## Discussion

Granted that miRNA mediated gene silencing serves as general defense mechanism against viruses, it would not be surprising that viruses also employ miRNAs to circumvent the host plant’s defense system [25]. Although DNA viruses were shown to encode miRNAs, it is generally believed that this pathway is not available to viruses that replicate in the cytoplasm, because miRNA biogenesis is initiated in the nucleus [64]. Interestingly, ACMV and EACMV-UG analyzed in the current study, replicate inside the nucleus [65]. However, even cytoplasmic RNA viruses were shown to encode and produce functional miRNAs during viral infection without impairing viral RNA replication [66]. In addition, viral miRNAs are derived not only from non-coding regions and introns, but also from coding mRNAs [22], [25], [58], which may be a fitness disadvantage for viruses with RNA genomes, if they were to encode regions that are prone to endonucleolytic cleavage by either Dicer or Drosha, thus resulting in reductions of genome/antigenome copy numbers, or mRNA production, which destabilizes miRNA processing of RNA genomes [35]. On the other hand, one important benefit, viruses could gain from employing miRNAs is the ability to regulate host and/or viral gene expression [35]. The combination of protein-mediated and miRNA-mediated regulations forms an intricate strategy for viruses to resist host defense systems and thus increase the opportunities of their survival [67]. This may be the strategy for viruses to extend the life of the infected cells and to establish a favorable environment for their replication.

The discovery of virus encoded miRNAs playing a crucial role in pathogenesis, sheds new light on host-pathogen interactions [68]. In addition to regulating endogenous gene expression, a host can use miRNA pathways as defense against viruses [51]. Furthermore, miRNAs are produced both by the begomoviruses and their two host plants, which can benefit either the virus or the host depending on particular interactions. Such interactions are likely to occur in viral pathogenesis determining the degree to which hosts restrict viral infection [58].

### Virus miR/miR* with Putative Targets in the *Jatropha* and Cassava Genome

Current data support the hypothesis that virus encoded miRNAs can target critical proteins associated with biotic responses in *Jatropha* and cassava. The predicted viral miR/miRs* showed complementarity to several regions in the *Jatropha* and cassava genome including proteins with molecular functions such as binding, catalysis, enzyme regulatory activity, electron carrier activity, nucleic acid/transcription factor activity, nutrient reservoir activity, structural molecule activity, transporter activity and signal transducer activity (Table S4–S7 in File S1). ACMV-mir-1–7, 7–3 and EACMV-UG-mir-2–4* and 2–5* targeted a protein similar to heat shock proteins [Genbank: GT972247]. Production of heat shock proteins can be triggered by exposure to different stress conditions, such as pathogen infection, exposure to toxins, nitrogen deficiency, or water deprivation [69]. ACMV-mir-1–4 and ACMV-mir-6–9* targeted an ADK (Adenosine kinase) protein, predicted from hairpins located on AC2 and AC1, respectively, while ACMV-mir-3–15* targeted a leucine-rich repeat family protein (Table S4–S5 in File S1). Both proteins are typical representatives of proteins involved in biotic stress response. The leucine rich repeat is present in the majority of immune receptors that form the innate immune system in plants [70]. An increase in ADK activity as factor of the host response to virus challenge was reported [71]; therefore the AC2 of geminivirus is a premeditated counter-response to inhibit ADK activity. Furthermore, ACMV-mir-5–12*, 6–9*, 7–3* and EACMV-UG-mir-2–4*, 2–5*, 2–8* -all miR*- targeted a zinc finger protein (Table S5 in File S1) correlated with the loss of biological function by inducing necrosis and suppressing PTGS in plants [72]. Further ACMV-miR 5–12* and ACMV-miR 6–9* were located on AC1, which is a replication associated protein. Although the predicted targets of miRNAs were not validated experimentally, there is a high probability of host miRs* to bind virus or plant ORFs as displayed by sequence complementarity, however further study need to be done in the future. High throughput sequencing data set demonstrated that pre-miRNAs can produce mature functional miRNAs from each of the two arms [73]. Furthermore, the regulatory role of miRs* was confirmed, especially due to their dynamic expression and evolutionary pattern [73]. MiR* sequences have been demonstrated to accumulate in response to pathogen invasion and thus their role in basal defense was hypothesized [74]. In order to avoid the successful establishment of pathogen, a host might activate silencing of some transcripts by using miR* sequences [19].

The serine/threonine protein kinase was targeted by EACMV-UG-mir-1–4 and EACMV-UG-mir-1–5, both located on AC2 and known to be involved in suppression of PTGS. In eukaryotic cells, regulation of signal transduction pathways through enzymatic protein phosphorylation by serine/threonine kinase is a widely distributed mechanism [75], suggesting that a serine/threonine protein kinase plays either a direct role in AC2 mediated pathogenesis or in PTGS suppression [72].

Although the host encoded miRs/miRs* have the capacity to bind crucial ORFs of ACMV and EACMV-UG, as revealed in this study, the analysed viruses evolve rapidly by undergoing mutations and recombinations, which could in turn lead to a loss of target for plant miRNAs. Since miRs/miRs* are short, minute changes in the viral genome provide them with opportunity to escape miRNAs related defense pathways [19]. However, in the current study, the regions in the virus genome targeted by the host miRNAs were rather conserved, which reduces the probability of escape from plant miRNA attack.

### Plant miRs/miRs* with Putative Targets on DNA-A of ACMV and EACMV-UG

The bipartite genome of ACMV and EACMV consists of DNA-A and DNA-B. While the A component encodes 6 ORFs, the B component only encodes the movement protein (BC1) and a nuclear shuttle protein (BV1). In the context of host pathogen interaction, DNA-A appears most attractive for further analyses, since it carries three viral suppressor of RNA silencing (AC2, AC4 and AV2) and, further, AC1-3 play key roles in replication and transcriptional regulation [76]. On the other hand, AC4 of ACMV and AC2 of EACMV are unique virus encoded PTGS proteins that bind to target mRNAs and presumably inactivate mature host miRNAs [77]. Recombination occurred during mixed infections in the field giving rise to novel virus species, e.g. EACMV-UG, with increased virulence and adaptation to new host species [78]. In fact, the symptom severity because of synergism between ACMV and EACMV is due to the action of both PTGS suppressors, AC4 and AC2, respectively, with differential roles targeting different steps in RNA silencing in a temporal and spatial manner [11]. Therefore any host miRNA sequence targeting ORFs AC4 and AC2 encoded by these viruses represents a potential candidate to develop a resistance strategy and possible achieve immunity against viral diseases. The current study revealed a number of plant miRs/miRs* potentially targeting viral genomic regions of ACMV and EACMV-UG. Host-encoded miRNAs are involved in modulating plant disease symptoms observed as developmental abnormalities by binding to virus – encoded PTGS suppressor proteins [79].

*In* *vitro* binding assays [77] revealed the ability of AC4 of ACMV to bind single stranded forms of miR159 and presumably inactivate the mature miRNAs, thus blocking the normal miRNA-mediated regulation of target mRNAs and thus resulting in developmental disturbances. Furthermore, it is known that IR is indispensable for viral replication and such a miRNA interaction could be effectively utilized by the host plant to attenuate viral replication at an early stage [19]. The predicted data also show that plants employ a cooperative regulation mode [19], [80] by using multiple different miRNAs to target ACMV and EACMV-UG in AC1 to disrupt viral replication. However such strategies are counteracted by the infecting virus carrying AC2 and AC4, known to be efficient suppressors of gene silencing [16], [61], [79], [81].

In the current study, plant miRNAs from conserved and highly expressed families (e.g miR156, miR160, miR164, miR166, miR169 and miR171) were shown to have potential targets in the genomes of two begomoviruses. This suggests that highly expressed plant miRNAs have multiple functions as well as multiple targets [50]. Furthermore, these miRNA families have multiple targets within the genome of the host plants, and some were shown to be expressed differentially in response to viral infections, playing a role in pathogen defense [20], [51], [77]. Interestingly, in this study the miR156, miR159, miR160, miR164, miR169, miR170, miR171, miR395 and miR395 were predicted and their expression was detected, which also were found in *Jatropha*, cassava, *Ricinus communis* and *Hevea brasiliensis* in response to abiotic stress, development, transcription factors and metabolism [82]–[87].

### Detection of Virus and Plant miRNA

Several methods allow the detection of mature miRNAs, which include among others Northern blotting with radiolabelled probes, semi quantitative reverse transcription-polymerase chain reaction (RT-PCR), quantitative RT-PCR, microarrays and massive parallel sequencing [37]–[38], [42]. Although qRT-PCR miRNA primer sets and detection kits are available commercially, their high costs make them not suitable for high throughput miRNA analyses [38]. Furthermore, the cost of massive parallel sequencing is still considerable, limiting the number of samples to be tested [88]. Therefore, in the current study RT-PCR using stem loop primers were applied, which, being more specific and sensitive than linear primers, enable a selective detection of only mature processed miRNA from small amounts of total RNA [37], [39]–[40], [89]. In addition, poor sensitivity of RT-PCR can be overcome by performing RT reactions with stem loop primers [37]–[38], [42]. Stem-loop primers are designed to include a short part that is complementary to the 3′ end of miRNA, a double-stranded part (the stem) and the loop containing the universal primer-binding sequence [37]–[38], [42]. This method appeals for its specificity and increased sensitivity compared to classical methods such as Northern blot, and could be easily used to identify and validate mature miRNAs [40], [90]. The structure of stem loop primers increases the ability to distinguish between a mature miRNA and its precursor, by reducing the annealing of the primers to the pre-miRNAs and pri-miRNAs [40], [42], [90]–[91]. Therefore, stem-loop RT-PCR was applied to explore expressed miRNAs in infected plants. Interestingly, the predicted virus miRNAs were clearly detected and validated from the infected plants carrying the isolate S4C6, which was previously sequenced and identified as an EACMV-UG strain.

Additionally, the current data confirmed that the expression of miRs is significantly higher than of pre-miRNA, which is in agreement with previous studies [91]. In fact, if mature miRNA and precursor were present at equal concentrations (if the precursor was not degraded), all isomiRs, which are heterogenous in length and predicted from the same precursor on both arms (Table 4) should be amplified. However, results showed that the expression patterns of isomiRs are not randomly distributed, which implies that the stem loop RT-PCR assay is specific to mature miRNAs (Figure 5). The current data indicate that multiple isomiRs with various sequences and expression levels might vary in diseased samples and also might have potential roles in multiple biological processes [73], [92]. The observed differences in isomiR expression could be influenced by recognition of the target site or/and binding capacity by the AGO complex [73], [91]. However, miRNA length variation could also reflect various downstream effects and might have important functional implications [91], [93], which were demonstrated in *Arabidopsis thaliana* with different activities on AGO1 and for individual loci in *Prunus persica* [93]–[95].

Plant miRs were detected also in infected and non-virus infected controls, which could be explained as cross-talk between the pathways of abiotic and biotic stresses [96]. In this case, miRNAs that play a role in response to abiotic stress might be the same miRs that are induced in response to biotic stresses [97]. Begomovirus infections have been found to increase the accumulation of plant miRNAs [98]. miRNAs 159, 164 and 319 accumulate to high levels and have been identified to regulate leaf deformations linked to geminivirus infections [99]. Induction of disease symptoms after infection of plants with ACMV has been attributed to the accumulation of miR159 and miR164 [98]. Furthermore, in cassava, infection by *Xanthomonas axonopodis* induces the miR160, miR170, miR171 and miR2911 [100].

## Conclusions

Using computational approaches allowed to show for the first time that ACMV and EACMV-UG encode possible pre-miRNA hairpins, and the expression of a number of predicted miRNAs could be detected using stem loop-RT PCR. This study is the first computational prediction of viral miRs/miRs* (both ACMV and EACMV-UG) and their targets in *Jatropha* and cassava ESTs and provides further support for the regulatory potential of viral as well as plant miRs/miRs*. The method led to the identification of virus miRNAs encoded in ORFs including AC2 and AC4, which are suppressors of RNA silencing and pathogenesis related proteins targeting pathways of functionally important genes for plant defense. In addition, several plant miRNAs were identified that could target the ORFs of both ACMV and EACMV-UG, representing potential plant molecules mediating antiviral defense. Different miRNAs could target single ORFs showing that the plant employs a cooperative regulation mode to enhance defense. In the end, the winner of this battle will be, who first manages to establish its attack or counter-defense strategy, and this might be additionally influenced by factors as time of infection, virus titer, physiological and developmental stage of the plant, etc.

These findings will be useful for the further identification of miRNAs in viruses and plants, and will speed up progress in *Eurphorbiaceae* genome research. They can be further used to engineer virus resistance via RNA based strategies, which could offer a long term solution by providing resistance to the important begomoviruses, ACMV and EACMV-UG [51].

## Supporting Information

File S1 **Contains the files: Table S1:** Forward and stem loop RT primers used in the detection of virus miRNAs from ACMV and EACMV-UG. **Table S2:** Forward and stem loop RT primers used in the detection of plant miRNAs. **Table S3:** Distinction and classification of real and pseudo miRNA precursors of 14 (9 ACMV and 5 EACMV-UG) hairpin sequences using MiPred. **Table S4:** Predicted putative targets of miRs/miRs* from ACMV and EACMV-UG in *Jatropha* ESTs using RNAhybrid. **Table S5:** Predicted putative targets of miRs/miRs* from ACMV and EACMV-UG in *Jatropha* ESTs using psRNATarget. **Table S6:** Predicted putative targets of miRs/miRs* from ACMV and EACMV-UG in cassava ESTs using RNAhybrid. **Table S7:** Predicted putative targets of miRs/miRs* from ACMV and EACMV-UG in cassava ESTs using psRNATarget. **Table S8:** Plant miRs/miRs* from the miRBase sequence DataBase, release 18, with putative targets in DNA-A of ACMV [Genbank: JN053423, JN053421] and EACMV-UG [Genbank: JN053454, JN053447] using RNAhybrid. **Table S9:** Plant miRs/miRs* from the miRBase sequence DataBase, release 18, with putative targets in DNA-A of ACMV [Genbank: JN053423, JN053421] and EACMV-UG [Genbank: JN053454, JN053447] using psRNATarget. **Table S10:** Predicted putative target location of plant miRs/miRs* in *Jatropha* ESTs using psRNATarget. **Table S11:** Predicted putative target location of plant miRs/miRs* in cassava ESTs using psRNATarget. **Table S12:** Summary of results for ACMV and EACMV-UG virus miRNA detection from cassava and *Jatropha* plant samples. **Table S13:** Summary of results for plant miRNA detection using on cassava and *Jatropha* plant samples.
(PDF)
