# Correction: Virus versus Host Plant MicroRNAs: Who Determines the Outcome of the Interaction?

**DOI:** 10.1371/journal.pone.0215588

**Published:** 2019-04-12

**Authors:** Fatemeh Maghuly, Rose C. Ramkat, Margit Laimer

After publication of this article [1], concerns were noted in the following figures:

1. Fig 5, the ACMV-mir-5-1 panel appears to be the same as the ACMV-mir-5-3 panel.
2. Fig 6, the miR4390 appears to be the same as the miR4399 panel.

**Fig 5:**
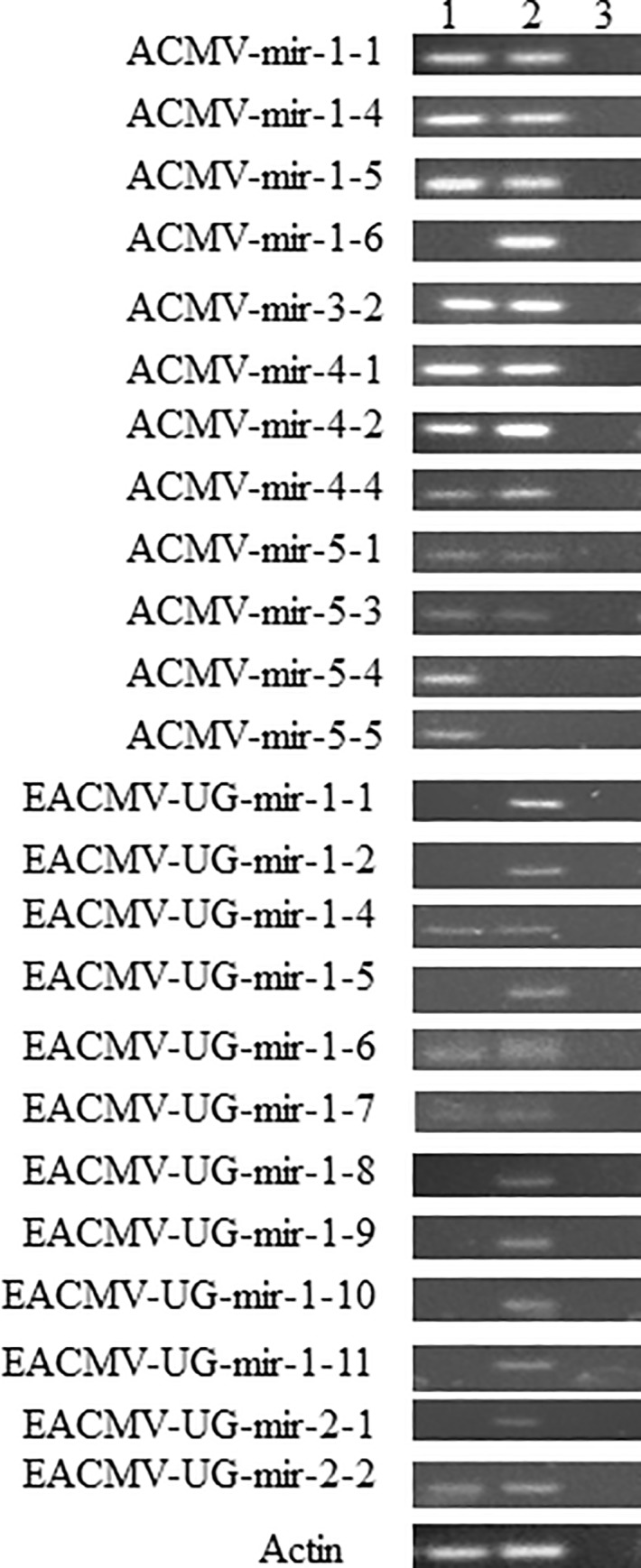
End point PCR amplification of ACMV and EACMV-UG virus miRNAs. PCR products of 60 bp were amplified in two plants co-infected with ACMV and EACMV: S2C6, S4C6,–RT control. Actin (76 bp) was used as internal control.

**Fig 6:**
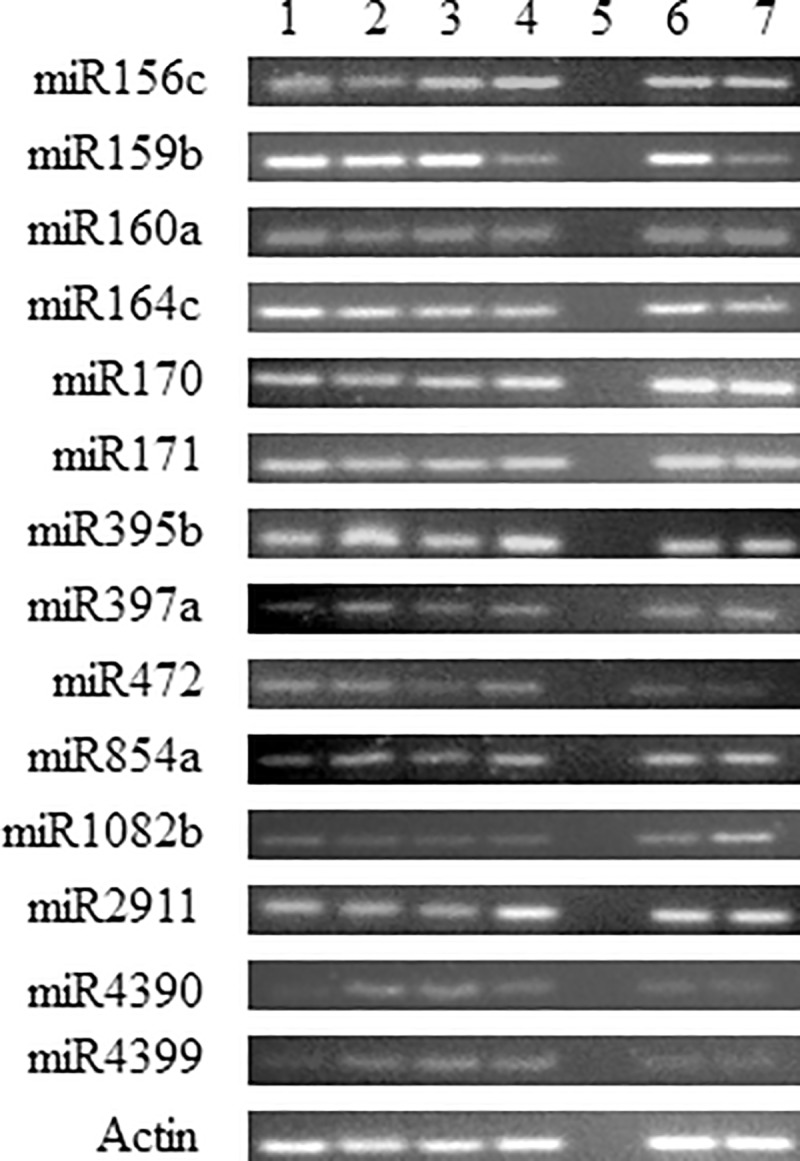
End point PCR amplification of plant miRNAs on cassava and Jatropha. PCR products of 60 bp were amplified. Lanes 1 to 4 are three infected and one non—infected cassava plant samples, respectively: S4C4, S2C6, S4C6, B2C15,–RT control. Lanes 6–7 are one infected and one non-infected Jatropha plant samples, respectively: K5J5, S4J12. Actin (76 bp) was used as internal control.

The authors acknowledge that an error was made during figure preparation for Fig 5 image of ACMV-mir 5–3 and Fig 6 image of miR430 due to the similarity in the images. The authors have provided replacement images for Fig 5 ACMV-mir-5-3 and Fig 6 miR4390 panels

The primary data underlying Figs 5 and 6 can be obtained via Figshare at DOI: 10.6084/m9.figshare.7804301 for Fig 5 and DOI: 10.6084/m9.figshare.7804409 for Fig 6.

The authors apologize for the errors in the published article.
