# Supplementary material for: Virus versus Host Plant MicroRNAs: Who Determines the Outcome of the Interaction?
Source: PLoS One. 2014 Jun 4;9(6):e98263. doi: 10.1371/journal.pone.0098263 (PMC4045720; doi:10.1371/journal.pone.0098263)
Supplement: File S1 — Contains the files: Table S1: Forward and stem loop RT primers used in the detection of virus miRNAs from ACMV and EACMV-UG. Table S2: Forward and stem loop RT primers used in the detection of plant miRNAs. Table S3: Distinction and classification of real and pseudo miRNA precursors of 14 (9 ACMV and 5 EACMV-UG) hairpin sequences using MiPred. Table S4: Predicted putative targets of miRs/miRs* from ACMV and EACMV-UG in Jatropha ESTs using RNAhybrid. Table S5: Predicted putative targets of miRs/miRs* from ACMV and EACMV-UG in Jatropha ESTs using psRNATarget. Table S6: Predicted putative targets of miRs/miRs* from ACMV and EACMV-UG in cassava ESTs using RNAhybrid. Table S7: Predicted putative targets of miRs/miRs* from ACMV and EACMV-UG in cassava ESTs using psRNATarget. Table S8: Plant miRs/miRs* from the miRBase sequence DataBase, release 18, with putative targets in DNA-A of ACMV [Genbank: JN053423, JN053421] and EACMV-UG [Genbank: JN053454, JN053447] using RNAhybrid. Table S9: Plant miRs/miRs* from the miRBase sequence DataBase, release 18, with putative targets in DNA-A of ACMV [Genbank: JN053423, JN053421] and EACMV-UG [Genbank: JN053454, JN053447] using psRNATarget. Table S10: Predicted putative target location of plant miRs/miRs* in Jatropha ESTs using psRNATarget. Table S11: Predicted putative target location of plant miRs/miRs* in cassava ESTs using psRNATarget. Table S12: Summary of results for ACMV and EACMV-UG virus miRNA detection from cassava and Jatropha plant samples. Table S13: Summary of results for plant miRNA detection using on cassava and Jatropha plant samples. (PDF) [file pone.0098263.s001.pdf]

Table S1 Forward and stem loop RT primers used in the detection of virus miRNAs from ACMV and EACMV-UG

miR	primer type	sequences of miR and primers
ACMV-mir-1-1	miR-sequence	AGCAAUGAAUGGCGUGUAUA
	forward primer	ACGCAGCAATGAATGGCGTGTA
	RT primer	GTCGTATCCAGTGCAGGGTCCGAGGTATTCGCACTGGATACGACAGGTA
ACMV-mir-1-3	miR-sequence	CAAUGAAUGGCGUGUAUACCUG
	forward primer	TCGCGCAATGAATGGCGTGTA
	RT primer	GTCGTATCCAGTGCAGGGTCCGAGGTATTCGCACTGGATACGACAGGTA
ACMV-mir-1-4	miR-sequence	AUGAAUGGCGUGUAUACCUGGG
	forward primer	TCGCGCATGAATGGCGTGTATA
	RT primer	GTCGTATCCAGTGCAGGGTCCGAGGTATTCGCACTGGATACGACCCAGG
ACMV-mir-1-5	miR-sequence	UGAAUGGCGUGUAUACCUGGGAA
	forward primer	CGCGCTGAATGGCGTGTATACC
	RT primer	GTCGTATCCAGTGCAGGGTCCGAGGTATTCGCACTGGATACGATTCCCA
ACMV-mir-1-6	miR-sequence	AUGGCGUGUAUACCUGGGAAAUA
	forward primer	ACGCGATGGCGTGTATACCTGG
	RT primer	GTCGTATCCAGTGCAGGGTCCGAGGTATTCGCACTGGATACGATATTTC
ACMV-mir-1-8	miR-sequence	UGUAUACCUGGGAAAUAAAC
	forward primer	TCGCGCGTGTATACCTGGGAA
	RT primer	GTCGTATCCAGTGCAGGGTCCGAGGTATTCGCACTGGATACGAGTTTAT
ACMV-mir-2-1	miR-sequence	UUUGGGUAUGUGAGAAAGAC
	forward primer	TCGCGCGTTTGGGTATGTGAG
	RT primer	GTCGTATCCAGTGCAGGGTCCGAGGTATTCGCACTGGATACGAGTCTTT
ACMV-mir-2-3	miR-sequence	UGGGUAUGUGAGAAAGACAUUCUU
	forward primer	ACGCGCTGGGTATGTGAGAAAGAC
	RT primer	GTCGTATCCAGTGCAGGGTCCGAGGTATTCGCACTGGATACGAAAGAAT
ACMV-mir-2-4	miR-sequence	GGUAUGUGAGAAAGACAUUCUUGG
	forward primer	CGCGCGGTATGTGAGAAAGACAT
	RT primer	GTCGTATCCAGTGCAGGGTCCGAGGTATTCGCACTGGATACGACCAAGA
ACMV-mir-2-7	miR-sequence	UGUGAGAAAGACAUUCUUGGCUUG
	forward primer	CGCGCGTGTGAGAAAGACATTCTT
	RT primer	GTCGTATCCAGTGCAGGGTCCGAGGTATTCGCACTGGATACGACAAGCC
ACMV-mir-3-1	miR-sequence	GAUGCAGCUCUCUACAGAUUU

	forward primer	CGCGCGGATGCAGCTCTCTAC
	RT primer	GTCGTATCCAGTGCAGGGTCCGAGGTATTCGCACTGGATACGAAAATCT
ACMV-mir-3-3	miR-sequence	UCUCCAUUCUGAUGCAGCUCUA
	forward primer	CGCGCTCTCCATTCTGATGCA
	RT primer	GTCGTATCCAGTGCAGGGTCCGAGGTATTCGCACTGGATACGATAGAGC
ACMV-mir-4-1	miR-sequence	GGUACAUGGGCUUAGGUGUAUGCU
	forward primer	TCGCGGTACATGGGCTTAGGTG
	RT primer	GTCGTATCCAGTGCAGGGTCCGAGGTATTCGCACTGGATACGAAGCATA
ACMV-mir-4-2	miR-sequence	ACAUGGGCUUAGGUGUAUGCUUGC
	forward primer	CGCGCACATGGGCTTAGGTGTAT
	RT primer	GTCGTATCCAGTGCAGGGTCCGAGGTATTCGCACTGGATACGAGCAAGC
ACMV-mir-4-3	miR-sequence	CAUGGGCUUAGGUGUAUGCUUGCA
	forward primer	CGCGCATGGGCTTAGGTGTATG
	RT primer	GTCGTATCCAGTGCAGGGTCCGAGGTATTCGCACTGGATACGATGCAAG
ACMV-mir-4-4	miR-sequence	UGGGCUUAGGUGUAUGCUUGCAA
	forward primer	TCGCGTGGGCTTAGGTGTATGC
	RT primer	GTCGTATCCAGTGCAGGGTCCGAGGTATTCGCACTGGATACGATTGCAA
ACMV-mir-4-5	miR-sequence	GCUUAGGUGUAUGCUUGCAA
	forward primer	CGCGCGGCTTAGGTGTATGC
	RT primer	GTCGTATCCAGTGCAGGGTCCGAGGTATTCGCACTGGATACGATTGCAA
ACMV-mir-5-1	miR-sequence	UCUUGCUUUUCCUCGUCUAGGAA
	forward primer	TCGCGTCTTGCTTTTCCTCGTC
	RT primer	GTCGTATCCAGTGCAGGGTCCGAGGTATTCGCACTGGATACGATTCCTA
ACMV-mir-5-3	miR-sequence	CUUGCUUUUCCUCGUCUAGGAACU
	forward primer	ACGCGCTTGCTTTTCCTCGTCTA
	RT primer	GTCGTATCCAGTGCAGGGTCCGAGGTATTCGCACTGGATACGAAGTTCC
ACMV-mir-5-4	miR-sequence	UUGCUUUUCCUCGUCUAGGAACUC
	forward primer	CGCGCTTGCTTTTCCTCGTCTAG
	RT primer	GTCGTATCCAGTGCAGGGTCCGAGGTATTCGCACTGGATACGAGAGTTC
ACMV-mir-5-5	miR-sequence	CUUUUCCUCGUCUAGGAACUCU
	forward primer	ACGCGCCTTTTCCTCGTCTAGG
	RT primer	GTCGTATCCAGTGCAGGGTCCGAGGTATTCGCACTGGATACGAAGAGTT

ACMV-mir-6-1	miR-sequence	AGGCAGCAAUAUGAGACCUUU
	forward primer	TCGCGCAGGCAGCAATATGAG
	RT primer	GTCGTATCCAGTGCAGGGTCCGAGGTATTCGCACTGGATACGAAAAGGT
ACMV-mir-6-3	miR-sequence	AGCAAUAUGAGACCUUUGGACUAG
	forward primer	ACGCGCAGCAATATGAGACCTTTG
	RT primer	GTCGTATCCAGTGCAGGGTCCGAGGTATTCGCACTGGATACGACTAGTC
ACMV-mir-6-4	miR-sequence	AUGAGACCUUUGGACUAGGUCCA
	forward primer	TCGCGCATGAGACCTTTGGACTA
	RT primer	GTCGTATCCAGTGCAGGGTCCGAGGTATTCGCACTGGATACGATGGACC
ACMV-mir-6-6	miR-sequence	GACUAGGUCCAGGUGUCCACAUAG
	forward primer	CGCGGACTAGGTCCAGGTGTCC
	RT primer	GTCGTATCCAGTGCAGGGTCCGAGGTATTCGCACTGGATACGACTATGT
ACMV-mir-7-1	miR-sequence	AGAAUGCCAUUUAGAGACACCU
	forward primer	CGCGCGAGAATGCCATTTAGAG
	RT primer	GTCGTATCCAGTGCAGGGTCCGAGGTATTCGCACTGGATACGAAGGTGT
EACMV-UG-mir-1-1	miR-sequence	UUUCGAAAUAGAGGGGAUUUGUUA
	forward primer	CGCGCTTTCGAAATAGAGGGGAT
	RT primer	GTCGTATCCAGTGCAGGGTCCGAGGTATTCGCACTGGATACGATAACAA
EACMV-UG-mir-1-2	miR-sequence	UCGAAAUAGAGGGGAUUUGUUAUG
	forward primer	TCGCGTCGAAATAGAGGGGATTT
	RT primer	GTCGTATCCAGTGCAGGGTCCGAGGTATTCGCACTGGATACGACATAAC
EACMV-UG-mir-1-4	miR-sequence	CGAAAUAGAGGGGAUUUGUUAU
	forward primer	CGCGCCGAAATAGAGGGGATT
	RT primer	GTCGTATCCAGTGCAGGGTCCGAGGTATTCGCACTGGATACGAATAACA
EACMV-UG-mir-1-5	miR-sequence	GAAAUAGAGGGGAUUUGUUAUGU
	forward primer	CGCGCGAAATAGAGGGGATTTG
	RT primer	GTCGTATCCAGTGCAGGGTCCGAGGTATTCGCACTGGATACGAACATAA
EACMV-UG-mir-1-6	miR-sequence	AAAUAGAGGGGAUUUGUUAUGUC
	forward primer	TCGCGCAAATAGAGGGGATTTGT
	RT primer	GTCGTATCCAGTGCAGGGTCCGAGGTATTCGCACTGGATACGAGACATA
EACMV-UG-mir-1-8	miR-sequence	AUAGAGGGGAUUUGUUAUGUCCCA
	forward primer	CGCGCGATAGAGGGGATTTGTTAT

	RT primer	GTCGTATCCAGTGCAGGGTCCGAGGTATTCGCACTGGATACGATGGGAC
EACMV-UG-mir-1-9	miR-sequence	AGAGGGGAUUUGUUAUGUCC
	forward primer	CGCGCGAGAGGGGATTTGTT
	RT primer	GTCGTATCCAGTGCAGGGTCCGAGGTATTCGCACTGGATACGAGGACAT
EACMV-UG-mir-1-10	miR-sequence	GGGAUUUGUUAUGUCCCAGGUAA
	forward primer	CGCGGGGATTTGTTATGTCCC
	RT primer	GTCGTATCCAGTGCAGGGTCCGAGGTATTCGCACTGGATACGATTACCT
EACMV-UG-mir-1-11	miR-sequence	AUUUGUUAUGUCCCAGGUAA
	forward primer	CGCGCGCATTTGTTATGTCCC
	RT primer	GTCGTATCCAGTGCAGGGTCCGAGGTATTCGCACTGGATACGATTACCT
EACMV-UG-mir-2-1	miR-sequence	CAGCAUUUAGCUCAGGUAUAU
	forward primer	TCGCGCCAGCATTTAGCTCAG
	RT primer	GTCGTATCCAGTGCAGGGTCCGAGGTATTCGCACTGGATACGAATATAC
EACMV-UG-mir-2-2	miR-sequence	AGGGCCAGCAUUUAGCUCAGGU
	forward primer	CGCGAGGGCCAGCATTTAGC
	RT primer	GTCGTATCCAGTGCAGGGTCCGAGGTATTCGCACTGGATACGAACCTGA

Last six nucleotides of miR sequence

Nucleotides added to increase primer melting temperature

Reverse complement of the last 6 nucleotides of the miR sequence

Universal reverse primer

Table S2 Forward and stem loop RT primers used in the detection of plant miRNAs

miR	primer type	sequences of miR and primers
miR156c	miR-sequence	UGACAGAAGAGAGUGAGCAC
	forward primer	CGCGCGCTGACAGAAGAGAGT
	RT primer	GTCGTATCCAGTGCAGGGTCCGAGGTATTCGCACTGGATACGAGTGCTC
miR159b	miR-sequence	UUUGGAUUGAAGGGAGCUCUU
	forward primer	ACGCGCTTTGGATTGAAGGGA
	RT primer	GTCGTATCCAGTGCAGGGTCCGAGGTATTCGCACTGGATACGAAAGAGC
miR160a	miR-sequence	UGCCUGGCUCCCUGUAUGCCA
	forward primer	TCGCTGCCTGGCTCCCTGT
	RT primer	GTCGTATCCAGTGCAGGGTCCGAGGTATTCGCACTGGATACGATGGCAT
miR164c	miRNA sequence	UGGAGAAGCAGGGCACGUGCG
	forward primer	GCGCTGGAGAAGCAGGGCA
	RT primer	GTCGTATCCAGTGCAGGGTCCGAGGTATTCGCACTGGATACGACGCACG
miR169aa	miR-sequence	GAGCCAAGAAUGACUUGUCGG
	forward primer	ACGCGCGAGCCAAGAATGACT
	RT primer	GTCGTATCCAGTGCAGGGTCCGAGGTATTCGCACTGGATACGACCGACA
miR170	miR-sequence	UGAUUGAGCCGUGUCAAUAUC
	forward primer	CGCGCTGATTGAGCCGTGTC
	RT primer	GTCGTATCCAGTGCAGGGTCCGAGGTATTCGCACTGGATACGAGATATT
miR171b	miRNA sequence	UUGAGCCGUGCCAAUAUCACG
	forward primer	AGCGCTTGAGCCGTGCCAATA
	RT primer	GTCGTATCCAGTGCAGGGTCCGAGGTATTCGCACTGGATACGACGTGAT
miR395b	miR-sequence	CUGAAGUGUUUGGGGGGACUC
	forward primer	TCGCGCTGAAGTGTTTGGGG
	RT primer	GTCGTATCCAGTGCAGGGTCCGAGGTATTCGCACTGGATACGAGAGTCC
miR397a	miR-sequence	UCAUUGAGUGCAGCGUUGAUG
	forward primer	TCGCGTCATTGAGTGCAGCG
	RT primer	GTCGTATCCAGTGCAGGGTCCGAGGTATTCGCACTGGATACGACATCAA
miR446	miR-sequence	CAUCAAUAUGAAUAUGGGAAAUGG
	forward primer	TCGCGCCATCAATATGAATATGGG
	RT primer	GTCGTATCCAGTGCAGGGTCCGAGGTATTCGCACTGGATACGACCATTT
miR477g	miR-sequence	CUCUCCCUCAAGUUCUUCUA

	forward primer	CGCGCGCTCTCCCTCAAGTT
	RT primer	GTCGTATCCAGTGCAGGGTCCGAGGTATTCGCACTGGATACGATAGAAG
miR472	miR-sequence	UUUUUCCUACUCCGCCCAUACC
	forward primer	CGCGCTTTTTCCTACTCCGCC
	RT primer	GTCGTATCCAGTGCAGGGTCCGAGGTATTCGCACTGGATACGAGGTATG
miR478a	miR-sequence	UGACGUGUCUUCUAUUUUUAGGGA
	forward primer	CGCGCGTGACGTGTCTTCTATTTT
	RT primer	GTCGTATCCAGTGCAGGGTCCGAGGTATTCGCACTGGATACGATCCCTA
miR482	miR-sequence	CCUACUCCUCCCAUUCC
	forward primer	CGCGCGCCCTACTCCTCC
	RT primer	GTCGTATCCAGTGCAGGGTCCGAGGTATTCGCACTGGATACGAGGAATG
miR771	miR-sequence	UGAGCCUCUGUGGUAGCCCUCA
	forward primer	ACGCGCTGAGCCTCTGTGGTAG
	RT primer	GTCGTATCCAGTGCAGGGTCCGAGGTATTCGCACTGGATACGATGAGGG
miR841	miR-sequence	UACGAGCCACUUGAAACUGAA
	forward primer	TCGCGCTACGAGCCACTTGAA
	RT primer	GTCGTATCCAGTGCAGGGTCCGAGGTATTCGCACTGGATACGATTCAGT
miR854a	miR-sequence	GAUGAGGAUAGGGAGGAGGAG
	forward primer	ACGCGCGATGAGGATAGGGAG
	RT primer	GTCGTATCCAGTGCAGGGTCCGAGGTATTCGCACTGGATACGACTCCTC
miR857	miR-sequence	UUUUGUAUGUUGAAGGUGUAU
	forward primer	CGCGCGCTTTTGTATGTTGAAG
	RT primer	GTCGTATCCAGTGCAGGGTCCGAGGTATTCGCACTGGATACGAATACAC
miR859	miR-sequence	UCUCUCUGUUGUGAAGUCAAA
	forward primer	TCGCGCGTCTCTCTGTTGTGAA
	RT primer	GTCGTATCCAGTGCAGGGTCCGAGGTATTCGCACTGGATACGATTTGAC
miR868	miR-sequence	UCAUGUCGUAAUAGUAGUCAC
	forward primer	CGCGCGCTCATGTCGTAATAGT
	RT primer	GTCGTATCCAGTGCAGGGTCCGAGGTATTCGCACTGGATACGAGTGACT
miR948	miR-sequence	UCAGGCUGUGUGGGAUCCGG
	forward primer	TCGCGTCAGGCTGTGTGGG
	RT primer	GTCGTATCCAGTGCAGGGTCCGAGGTATTCGCACTGGATACGACCGGAT

miR1082b	miR-sequence	GUGUUGGCCUGCUGGCCGGCG
	forward primer	CGCGTGTTGGCCTGCTGG
	RT primer	GTCGTATCCAGTGCAGGGTCCGAGGTATTCGCACTGGATACGACGCCGG
miR1084	miR-sequence	UAACUCAGGUGGUAUGUUCCCA
	forward primer	TCGCGCGTAACTCAGGTGGTATG
	RT primer	GTCGTATCCAGTGCAGGGTCCGAGGTATTCGCACTGGATACGATGGGAA
miR1107	miR-sequence	GGUGCUGGUUCCAAUUCAGGU
	forward primer	CGCGGGTGCTGGTTCCAAT
	RT primer	GTCGTATCCAGTGCAGGGTCCGAGGTATTCGCACTGGATACGAACCTGA
miR1111	miR-sequence	GCCAUGACUAAGCAGGACCAG
	forward primer	CGCGCGCCATGACTAAGCAG
	RT primer	GTCGTATCCAGTGCAGGGTCCGAGGTATTCGCACTGGATACGACTGGTC
miR1117	miR-sequence	UAGUACCGGUUCGUGGCACGAACC
	forward primer	ACGCTAGTACCGGTTCGTGGCA
	RT primer	GTCGTATCCAGTGCAGGGTCCGAGGTATTCGCACTGGATACGAGGTTCG
miR1118	miR-sequence	CACUACAUUAUGGAAUGGAGGGA
	forward primer	CGCGCGCACTACATTATGGAATG
	RT primer	GTCGTATCCAGTGCAGGGTCCGAGGTATTCGCACTGGATACGATCCCTC
miR1311	miR-sequence	UCAGAGUUUUGCCAGUUCCGCC
	forward primer	ACGCGCTCAGAGTTTTGCCAGT
	RT primer	GTCGTATCCAGTGCAGGGTCCGAGGTATTCGCACTGGATACGAGGCGGA
miR1446a	miR-sequence	UUCUGAACUCUCUCCCUCAA
	forward primer	CGCGCGCTTCTGAACTCTCTC
	RT primer	GTCGTATCCAGTGCAGGGTCCGAGGTATTCGCACTGGATACGATTGAGG
miR1510b	miR-sequence	UGUUGUUUUACCUAUUCCACC
	forward primer	TCGCGCGCTGTTGTTTTACCTAT
	RT primer	GTCGTATCCAGTGCAGGGTCCGAGGTATTCGCACTGGATACGAGGTGGA
miR1520j	miR-sequence	AAGAACGUGACACAUGACAAUCAA
	forward primer	TCGCGAAGAACGTGACACATGAC
	RT primer	GTCGTATCCAGTGCAGGGTCCGAGGTATTCGCACTGGATACGATTGATT
miR1864	miR-sequence	UUGUAGUAACGUGAUGGUCAAUGU
	forward primer	CGCGCGTTGTAGTAACGTGATGGT
	RT primer	GTCGTATCCAGTGCAGGGTCCGAGGTATTCGCACTGGATACGAACATTG

miR1887	miR-sequence	UACUAAGUAGAGUCUAAGAGA
	forward primer	CGCGCGCGTACTAAGTAGAGTCT
	RT primer	GTCGTATCCAGTGCAGGGTCCGAGGTATTCGCACTGGATACGATCTCTT
miR2094-3p	miR-sequence	CAGAGCUGUGGCAUCCACGUCG
	forward primer	ACGCCAGAGCTGTGGCATCC
	RT primer	GTCGTATCCAGTGCAGGGTCCGAGGTATTCGCACTGGATACGACGACGT
miR2104	miR-sequence	GCGGCGAGGGGAUGCGAGCGUG
	forward primer	GCGGCGAGGGGATGCG
	RT primer	GTCGTATCCAGTGCAGGGTCCGAGGTATTCGCACTGGATACGACACGCT
miR2119	miR-sequence	UCAAAGGGAGUUGUAGGGGAA
	forward primer	CGCGCGTCAAAGGGAGTTGTA
	RT primer	GTCGTATCCAGTGCAGGGTCCGAGGTATTCGCACTGGATACGATTCCCC
miR2588a	miR-sequence	UAACACUGUGCAACUAAGUCC
	forward primer	TCGCGCGTAACACTGTGCAACT
	RT primer	GTCGTATCCAGTGCAGGGTCCGAGGTATTCGCACTGGATACGAGGACTT
miR2630a	miR-sequence	UGGUUUUGGUCCUUGGUAUUU
	forward primer	CGCGCTGGTTTTGGTCCTTG
	RT primer	GTCGTATCCAGTGCAGGGTCCGAGGTATTCGCACTGGATACGAAAATAC
miR2640a	miR-sequence	UUCCUUGCCGGAGCUGGACUAC
	forward primer	CGCTTCCTTGCCGGAGCTG
	RT primer	GTCGTATCCAGTGCAGGGTCCGAGGTATTCGCACTGGATACGAGTAGTC
miR2668	miR-sequence	UUCAUCCUUGCAAUUAGGGGUC
	forward primer	CGCGCGTTCATCCTTGCAATTA
	RT primer	GTCGTATCCAGTGCAGGGTCCGAGGTATTCGCACTGGATACGAGACCCC
miR2669	miR-sequence	AAAGUUCAGUCUUCAUAGUAUC
	forward primer	TCGCGCGAAAGTTCAGTCTTCAT
	RT primer	GTCGTATCCAGTGCAGGGTCCGAGGTATTCGCACTGGATACGAGATACT
miR2905	miR-sequence	UACAUGUCAGUGACAAAGGCA
	forward primer	CGCGCGCTACATGTCAGTGACA
	RT primer	GTCGTATCCAGTGCAGGGTCCGAGGTATTCGCACTGGATACGATGCCTT
miR2911	miR-sequence	GGCCGGGGGACGGGCUGGGA
	forward primer	TGGCCGGGGGACGGG

	RT primer	GTCGTATCCAGTGCAGGGTCCGAGGTATTCGCACTGGATACGATCCCAG
miR2927	miR-sequence	UGUCGUCGUCGAUGGAGCCCAUG
	forward primer	ACGCTGTCGTCGTCGATGGAG
	RT primer	GTCGTATCCAGTGCAGGGTCCGAGGTATTCGCACTGGATACGACATGGG
miR3513-5p	miR-sequence	UUGAUAAGAUAGAAAUUGUAU
	forward primer	CGCGCGCGTTGATAAGATAGAAA
	RT primer	GTCGTATCCAGTGCAGGGTCCGAGGTATTCGCACTGGATACGAATACAA
miR3633b	miR-sequence	GGAAUGGGUGGCUGGGAUCUA
	forward primer	CGCGGAATGGGTGGCTGG
	RT primer	GTCGTATCCAGTGCAGGGTCCGAGGTATTCGCACTGGATACGATAGATC
miR4232	miR-sequence	UCACAUUUAUUAGGAUGUGUGC
	forward primer	CGCGCGCTCACATTTATTAGGAT
	RT primer	GTCGTATCCAGTGCAGGGTCCGAGGTATTCGCACTGGATACGAGCACAC
miR4238	miR-sequence	UUUUUGCAAAUUAAAACCCCAA
	forward primer	CGCGCGCTTTTTGCAAATTAAAA
	RT primer	GTCGTATCCAGTGCAGGGTCCGAGGTATTCGCACTGGATACGATTGGGG
miR4243	miR-sequence	UUGAAAUUGUAGAUUUCGUAC
	forward primer	CGCGCGCGTTGAAATTGTAGATT
	RT primer	GTCGTATCCAGTGCAGGGTCCGAGGTATTCGCACTGGATACGAGTACGA
miR4246	miR-sequence	AAAUCCAAUUUUCAUUGCUUAA
	forward primer	CGCGCGAAATCCAATTTTCATT
	RT primer	GTCGTATCCAGTGCAGGGTCCGAGGTATTCGCACTGGATACGATTAAGC
miR4379	miR-sequence	UAGAGUGUAUACUGUGAGAGGCCU
	forward primer	CGCGCGCTAGAGTGTATACTGTGAG
	RT primer	GTCGTATCCAGTGCAGGGTCCGAGGTATTCGCACTGGATACGAAGGCCT
miR4390	miR-sequence	UCGUACUCGUCGGGUAUCGGGUAU
	forward primer	ACGCGTCGTACTCGTCGGGTATC
	RT primer	GTCGTATCCAGTGCAGGGTCCGAGGTATTCGCACTGGATACGAATACCC
miR4399	miR-sequence	UUAACGAAAAAGGACUAACGAC
	forward primer	TCGCGCGTTAACGAAAAAGGACT
	RT primer	GTCGTATCCAGTGCAGGGTCCGAGGTATTCGCACTGGATACGAGTCGTT
miR4409	miR-sequence	UAACAAGUGGGUUUGUUGACUG

	forward primer	CGCGCGTAACAAGTGGGTTTGT
	RT primer	GTCGTATCCAGTGCAGGGTCCGAGGTATTCGCACTGGATACGACAGTCA
miR4412	miR-sequence	UGUUGCGGGUAUCUUUGCCUC
	forward primer	TCGCGCTGTTGCGGGTATCTT
	RT primer	GTCGTATCCAGTGCAGGGTCCGAGGTATTCGCACTGGATACGAGAGGCA

Last six nucleotides of miR sequence

Nucleotides added to increase primer melting temperature

Reverse complement of the last 6 nucleotides of the miR sequence

Universal reverse primer

Table S3 Distinction and classification of real and pseudo miRNA precursors of 14 (9 ACMV and 5 EACMV-UG) hairpin sequences using MiPred.

**Hairpin name**	**ACMV 1**
Sequence	AGCAAUGAAUGGCGUGUAUACCUGGGAAAUAAACAAUCCCCUGUAUUUCACAAUCACCAGGCACCAACAACGACCAUUCCUGCU
Length	84
Pre-miRNA-like hairpin?	Yes
Secondary structure	((((..((((((((((...(((((((((..(((......)))))))).......)))))......)).))).)))))).))))
MFE	-21.71
p-value (shuffle times:1000)	0.016
Prediction result	Real microRNA precursor
Prediction confidence	52.10%

**Hairpin name**	**ACMV 2**
Sequence	UUUGGGUAUGUGAGAAAGACAUUCUUGGCUUGAAUUCAAAACGAGGAGUUCUCAUUUGACCAAG
Length	64
Pre-miRNA-like hairpin?	Yes
Secondary structure	.((((....(((((((.....((((((..(((....)))..)))))).)))))))....)))).
MFE	-20.8
p-value (shuffle times:1000)	0.007
Prediction result	Real microRNA precursor
Prediction confidence	56.10%

**Hairpin name**	**ACMV 3**
Sequence	GUUCUCCAUUCUGAUGCAGCUCUCUACAGAUUUUAAUGAACUUAGGGUUUGAUGGGAGAGAGAGUGUUUGAAGGAAGGAC
Length	80
Pre-miRNA-like hairpin?	Yes
Secondary structure	((((((.(((.(((((..((((((.((((((((((.....))))))))))...))))))...))))).))))).)))))
MFE	-25.9
p-value (shuffle times:1000)	0.015
Prediction result	Real microRNA precursor
Prediction confidence	65.50%

**Hairpin name**	**ACMV 4**
Sequence	UCCAGACUCGGUACAUGGGCUUAGGUGUAUGCUUGCAAUUAAAUAUUUGCAGGCCUUAGAGGAUACAUACGAGCCCAGUACUUUGGG
Length	87
Pre-miRNA-like hairpin?	Yes
Secondary structure	.((((....(((((.(((((((..((((((((((((((........))))))))........))))))..)))))))))))))))).
MFE	-35.7
p-value (shuffle times:1000)	0.001
Prediction result	Real microRNA precursor
Prediction confidence	75.40%

**Hairpin name**	**ACMV 5**

Sequence	UUCUUGCUUUUCCUCGUCUAGGAACUCUUUAUAGGACGAGGUAGGUCCUGGAUUGCAGAGGAAGAUAGUGGGAA
Length	74
Pre-miRNA-like hairpin?	Yes
Secondary structure	.(((..((((((((((((((((.((((((.......)))).)).))))))))....)))))))..))..))).
MFE	-27.5
p-value (shuffle times:1000)	0.018
Prediction result	Real microRNA precursor
Prediction confidence	60.70%

**Hairpin name**	**ACMV 6**
Sequence	ACCAGGCAGCAAUAUGAGACCUUUGGACUAGGUCCAGGUGUCCACAUAGGUAAUUGUGUGGGCCUAAAGAUCUGGC CCAUAUCGUCUUCCCUGUUCUGCU
Length	100
Pre-miRNA-like hairpin?	Yes
Secondary structure	....(((((.((((.(((((...(((.(((((((..((.((((((((((....)))))))))))...))))))).)))....)))))...)))))))))
MFE	-38.7
p-value (shuffle times:1000)	0.005
Prediction result	Real microRNA precursor
Prediction confidence	73%

**Hairpin name**	**ACMV 7**
Sequence	AGAAUGCCAUUUAGAGACACCUAUAUAAUGUCUCCAAUUAACAGGAGGACUUGCUCAAGAGUGUCUCUAGUUGAGU GUCUC
Length	81
Pre-miRNA-like hairpin?	Yes
Secondary structure	(((.(((((.((((((((((((.......((((((........))).)))........)).))))))))))).)).)))))).
MFE	-24.44
p-value (shuffle times:1000)	0.019
Prediction result	Real microRNA precursor
Prediction confidence	53.30%

**Hairpin name**	**ACMV 8**
Sequence	GUAUGUCGGCCAAUCAUGUUGUAGCUGUAAAAGUUAUGUAUUAGUGGUGGGCCACUAUAUAC
Length	62
Pre-miRNA-like hairpin?	Yes
Secondary structure	((((((.((((.(((((..(((((((.....))))))).....))))).))))...))))))
MFE	-19
p-value (shuffle times:1000)	0.022
Prediction result	Real microRNA precursor
Prediction confidence	53.30%

**Hairpin name**	**ACMV 9**
Sequence	CAGUCUGAGGCUGUAAGGUCGUCCAGAUCUCGAAGUUGAGAAAACAUUUGUGAAUCCCCAGCCCUUCCUCAGGUUG
Length	76
Pre-miRNA-like hairpin?	Yes
Secondary structure	((..(((((((((...((...(((((((((((....)))))).....)))).))..)).)))))).....))))..))

MFE	-20.5
p-value (shuffle times:1000)	0.074
Prediction result	Pseudo microRNA precursor
Prediction confidence	77%

**Hairpin name**	**EACMV-UG 1**
Sequence	UUUCGAAAUAGAGGGGAUUUGUUAUGUCCCAGGUAAAAACGCCAUUCCUUGCUUGAGGCGCAGUGAUGAGUUCCCCUGUGCGAGA
Length	85
Pre-miRNA-like hairpin?	Yes
Secondary structure	(((((..((((.(((((((..((((((((((((((............))))))).)).))).)))..))))))))))).)))))
MFE	-29.3
p-value (shuffle times:1000)	0.006
Prediction result	Real microRNA precursor
Prediction confidence	74.80%

**Hairpin name**	**EACMV-UG 2**
Sequence	GGAGGGCCAGCAUUUAGCUCAGGUAUAUGCAGACGCGUUAAUGCUUCGUCUAAAUCGAGGCUCUUC
Length	66
Pre-miRNA-like hairpin?	Yes
Secondary structure	((((((((.(.((((((...((((((((((....))))..))))))...))))))))..))))))))
MFE	-21.7
p-value (shuffle times:1000)	0.025
Prediction result	Real microRNA precursor
Prediction confidence	56.30%

**Hairpin name**	**EACMV-UG 3**
Sequence	CGAGCAGCCGCAUUCGAGGUCGACCCGCCUACGUCGGACGGCCCUGGUCUUCGCUGUGCGGUGUUGGACUUUGAUGGGCACUUG
Length	84
Pre-miRNA-like hairpin?	Yes
Secondary structure	((((..(((.(((.((((((((((((((..(((.((((.(((....))))))).))))))).))).))))))))))))).))))
MFE	-34.4
p-value (shuffle times:1000)	0.098
**Prediction result**	Pseudo microRNA precursor
Prediction confidence	50%

**Hairpin name**	**EACMV-UG 4**
Sequence	AAGUCUAUAGCAAUCGGUGGAAUGGGGGGCAAUAUAUAUGAUGUCCCCCAAUGGCAUAUGUGUAAAUAGGUAGACUU
Length	77
Pre-miRNA-like hairpin?	Yes
Secondary structure	((((((((.(((....(((..((((((((((.((....)).)))))))))).))..)))...)))......))))))))
MFE	-25.1
p-value (shuffle times:1000)	0.018
Prediction result	Pseudo microRNA precursor
Prediction confidence	51%

**Hairpin name**	**EACMV-UG 5**
Sequence	ACCGGCUCUUGGCAUAUUGGCUGUCGUUUUGGAUCGGGGGACACUCAAAACUCCAGGGGAACGGU
Length	65
Pre-miRNA-like hairpin?	Yes
Secondary structure	((((.(((((((....(((..((((.(((......))).))))..)))....)))))))..))))
MFE	-20.7
p-value (shuffle times:1000)	0.106
Prediction result	Pseudo microRNA precursor
Prediction confidence	66.60%

Table S4 Predicted putative targets of miRs/miRs* from ACMV and EACMV-UG in *Jatropha* ESTs using RNAhybrid

miR/miR*	predicted miRNA and target contig	MFE	Start position	Genbank accession	Target description	Target Function
**ACMV-mir-1-1**	target 5' U C G 3'<br>GGUG ACAC CUAUUUAUUGCU<br>CCAU UGUG GGUAAGUAACGA<br>miRNA 3' GU A C 5'	-29	480	GW878812	similar to hydroxyacylglutathione hydrolase [Ricinus communis, EEF41604.1]	catalytic activity
	target 5' G C U G A 3'<br>AGG AUGUGC CCAUU AUUGCU<br>UCC UAUGUG GGUAA UAACGA<br>miRNA 3' G A C G 5'	-26.1	311	GT973521	similar to ATP-citrate lyase [Ricinus communis, EEF50019.1]	binding
**ACMV-mir-1-2**	target 5' A U A C 3'<br>AUAC C UCAUUUAUUGCU<br>UAUG G GGUAAGUAACGA<br>miRNA 3' A U C 5'	-21.7	244	GW879878	similar to xyloglucan endotransglucosylase / hydrolase protein A [Ricinus communis, EEF43915.1]	catalytic activity
**ACMV-mir-1-3**	target 5' U C G A 3'<br>GGUA AUA GCCAUUCAUU<br>CCAU UGU CGGUAAGUAA<br>miRNA 3' GU A G C 5'	-28	316	FM894433	similar to protein phosphatase 2a, regulatory subunit [Ricinus communis, EEF37048.1]	enzyme regulator activity
**ACMV-mir-1-4**	target 5' C G G A A 3'<br>CCU AGGUG ACAUG CAUUCAU<br>GGG UCCAU UGUGC GUAAGUA<br>miRNA 3' A G 5'	-27.4	263	GW614613	similar to glutathione-s-transferase theta, gst [Ricinus communis, EEF51173.1]	catalytic activity
	target 5' A C G 3'<br>CAGGUA AUGCGCUGUUUGU<br>GUCCAU UGUGCGGUAAGUA<br>miRNA 3' GG A 5'	-27.7	304	GT974021	similar to adenosine kinase [Ricinus communis, EEF30697.1]	catalytic activity

	target 5' G U A C 3'<br>CCC GGUGUGUAUG CAUUCA<br>GGG CCAUAUGUGC GUAAGU<br>miRNA 3' U G A 5'	-28	650	GT981187	similar to 5-methyltetrahydropteroyltriglutamate--homocysteine methyltransferase [Ricinus communis, EEF46521.1]	catalytic activity
**ACMV-mir-1-5**	target 5' A U G U 3'<br>UCUU GGGUG UG AUGCCAUUCA<br>AGGG UCCAU AU UGCGGUAAGU<br>miRNA 3' A G 5'	-25.4	206	GW612329	similar to serine/threonine protein kinase [Ricinus communis, EEF43433.1]	binding
	target 5' G U C 3'<br>UCUAGGUG GC UGCUAUUUA<br>GGGUCCAU UG GCGGUAAGU<br>miRNA 3' AA A U 5'	-25	555	GW616170	similar to ankyrin repeat-containing protein [Ricinus communis, EEF33285.1]	binding
	target 5' A C A 3'<br>UUCCUGGGU G AUAUGUUGUUCA<br>AAGGGUCCA U UGUGCGGUAAGU<br>miRNA 3' A 5'	-26.5	267	FM888971	similar to NADH-ubiquinone oxidoreductase 39 kD subunit [Ricinus communis, EEF35812.1]	binding
	target 5' U U 3'<br>UUUUCAGG AUG GCGUCAUUC<br>AAGGGUCC UAU UGCGGUAAG<br>miRNA 3' A G U 5'	-25.9	194	GW616682	similar to proteasome subunit beta type 7,10 [Ricinus communis, EEF36326.1]	catalytic activity
**ACMV-mir-1-6**	target 5' C G G G C 3'<br>UUU C GGGUGUACAUGU GU<br>AAA G UCCAUAUGUGCG UA<br>miRNA 3' AU G G G 5'	-27.3	192	FM888455	similar to short-chain dehydrogenase [Ricinus communis, EEF51203.1]	binding
**ACMV-mir-1-7**	target 5' C C 3'<br>UUUAUUUCUUAGGUAUGC<br>AAAUAAAGGGUCCAUAUG<br>miRNA 3' AC U 5'	-26.9	440	FM895812	similar to profilin-like protein [Jatropha curcas, ACV50427.1]	

**ACMV-mir-1-8**	target 5' C C 3'<br>UUUAUUUCUUAGGUAUGC<br>AAAUAAAGGGUCCAUAUG<br>miRNA 3' C U 5'	-26.9	440	FM895812	similar to profilin-like protein [Jatropha curcas, ACV50427.1]	
**ACMV-mir-1-9***	target 5' C C 3'<br>GGUU UUGUUGGUGCCUGG<br>CCAG AACAACCACGGACC<br>miRNA 3' UA C 5'	-36	112	GW618656	similar to phosphatidylcholine-sterol O-acyltransferase [Ricinus communis, EEF38239.1]	catalytic activity
**ACMV-mir-1-10***	target 5' A C A C 3'<br>GGUGG UUG U UUGGUGCCUGG<br>UUACC AGC A AACCACGGACC<br>miRNA 3' C A C 5'	-30.7	305	FM896626	similar to 60S ribosomal protein L3 [Ricinus communis, EEF38042.1]	structural molecule activity
**ACMV-mir-1-12***	target 5' A A A C 3'<br>AG GAAU G CGUUGUUGG GUUUG<br>UC CUUA C GCAACAACC CGGAC<br>miRNA 3' C A A 5	-25.6	245	GT977039	similar to cysteine protease inhibitor [Ricinus communis, EEF36811.1]	enzyme regulator activity
**ACMV-mir-1-13***	target 5' U U C U C 3'<br>GCAG G U GGUU UUGUUGGUGCU<br>CGUC U A CCAG AACAACCACGG<br>miRNA 3' C U C 5'	-28.1	659	GT977124	similar to uroporphyrinogen decarboxylase [Ricinus communis, EEF41551.1]	catalytic activity
**ACMV-mir-1-14***	target 5' U U A 3'<br>GUAGGA UGGUUGUUGUUGG<br>CGUCCU ACCAGCAACAACC<br>miRNA 3' U 5'	-34.9	298	GT980390	similar to phosphoribosylamine-glycine ligase [Ricinus communis, EEF35937.1]	binding
	target 5' C C C C 3'<br>GCAG G UGGUCGUUGUUG<br>CGUC U ACCAGCAACAAC<br>miRNA 3' C U C 5'	-28.6	121	GW879285	similar to carboxypeptidase B2 precursor [Ricinus communis, EEF52851.1]	catalytic activity

**ACMV-mir-2-1**	target 5' A U 3'<br>UUUUCUCACAUACCCAGA<br>GAAAGAGUGUAUGGGUUU<br>miRNA 3' CA 5'	-32.6	99	GT977225	similar to myo inositol monophosphatase [Ricinus communis, EEF38236.1]	catalytic activity
	target 5' C A C 3'<br>UCUUUCUCACA AUCCAAA<br>AGAAAGAGUGU UGGGUUU<br>miRNA 3' C A 5'	-29.1	52	FM887240	similar to protein CYPRO4 [Ricinus communis, EEF43694.1]	
**ACMV-mir-2-2**	target 5' U C G G 3'<br>UGUCUU UCU ACAUACUUGA<br>ACAGAA AGA UGUAUGGGUU<br>miRNA 3' UU G 5'	-25.8	248	GW611858	similar to aminopeptidase [Ricinus communis, EEF51211.1]	catalytic activity
**ACMV-mir-2-3**	target 5' A C A C 3'<br>AAGAA G U UUUCUUACAUACCCA<br>UUCUU C A AAAGAGUGUAUGGGU<br>miRNA 3' A G 5'	-28.3	468	GT975742	similar to kif4 [Ricinus communis, EEF45542.1]	binding
**ACMV-mir-2-4**	target 5' A C C C 3'<br>UAGGGA UGUUUUUCUU CAUGCC<br>GUUCUU ACAGAAAGAG GUAUGG<br>miRNA 3' G U 5'	-28.4	608	GW875806	similar to predicted protein [Populus trichocarpa, XP_002332240.1	
**ACMV-mir-2-5**	target 5' U G G U 3'<br>CCAAGGAUG U UUCUUAUAUGC<br>GGUUCUUAC G AAGAGUGUAUG<br>miRNA 3' A A 5'	-27.6	335	GW876781	similar to glycerophosphoryl diester phosphodiesterase [Ricinus communis, EEF47756.1]	catalytic activity
**ACMV-mir-2-6**	target 5' G U C C 3'<br>GAGCUAA AGUGUCUUUU UGCAU<br>UUCGGUU UUACAGAAAG GUGUA<br>miRNA 3' C A 5'	-27.2	508	GW879600	Translation initiation factor IF-2 protein	binding
**ACMV-mir-2-7**	target 5' G U C C C 3'	-29.5	150	GW619363	similar to LOB domain-containing protein [Ricinus communis, EEF30755.1]	

	CAAGCUAA G GA UGUCUUUCUCGC<br>GUUCGGUU C UU ACAGAAAGAGUG<br>miRNA 3' U 5'					
	target 5' C U 3'<br>CAAGUUAGGGG GUUUUUCU GCA<br>GUUCGGUUCUU CAGAAAGA UGU<br>miRNA 3' A G 5'	-25.2	231	FM890400	similar to cytochrome P450 [Ricinus communis, EEF40189.1]	electron carrier activity
**ACMV-mir-2-8***	target 5' U A G C 3'<br>UCAGAUGGGAA U CUCGUUUU<br>AGUUUACUCUU A GAGCAAAA<br>miRNA 3' G G C 5'	-28.2	207	FM895375	similar to nucleolysin tia-1 [Ricinus communis, EEF33213.1]	binding
	target 5' C C C C 3'<br>UCAGAUGA AGCUCU CGUUUU<br>AGUUUACU UUGAGG GCAAAA<br>miRNA 3' C A C 5'	-26.7	95	GW614586	similar to neutral alpha-glucosidase ab precursor [Ricinus communis, EEF33007.1]	binding
	target 5' U C 3'<br>UUUUGCUCUUCAGGA UGAAC<br>AAAACGAGGAGUUCU AUUUG<br>miRNA 3' C C A 5'	-29.4	377	GW611630	similar to protein phosphatase-2c [Ricinus communis, EEF47391.1]	binding
**ACMV-mir-3-1**	target 5' C U 3'<br>AAGUUUGUGGA AGCUGCAUC<br>UUUAGACAUCU UCGACGUAG<br>miRNA 3' C 5'	-31.9	115	GW878887	similar to protein KTI12 [Ricinus communis, EEF50764.1]	binding
**ACMV-mir-3-2**	target 5' U C A U 3'<br>GGA CUGCAUCAGAA GGGGAA<br>UCU GACGUAGUCUU CCUCUU<br>miRNA 3' C A 5'	-34	575	GW877851	similar to monothiol glutaredoxin-4 [Ricinus communis, EEF44744.1]	electron carrier activity
	target 5' U U A 3'<br>GAGCUGUAU G GAAUGGAGAG<br>CUCGACGUA U CUUACCUCUU	-31.5	612	GW878121	similar to DAG protein, chloroplast precursor [Ricinus communis, EEF29510.1]	binding

	miRNA 3' U G 5'					
**ACMV-mir-3-3**	target 5' U U G 3'<br>GAGCUGUAU G GAAUGGAGA<br>CUCGACGUA U CUUACCUCU<br>miRNA 3' AU G 5'	-30.9	612	GW878121	similar to DAG protein, chloroplast precursor [Ricinus communis, EEF29510.1]	binding
	target 5' A A U C 3'<br>UAG AGCUGU GUCGGAAUGGAG<br>AUC UCGACG UAGUCUUACCUC<br>miRNA 3' U 5'	-32.5	456	GW613728	similar to cytochrome P450 [Ricinus communis, EEF52326.1]	electron carrier activity
	target 5' A U A 3'<br>UAG GUUGC UU GGAUGGAGG<br>AUC CGACG AG CUUACCUCU<br>miRNA 3' U U U 5'	-26.3	145	FM890413	similar to vacuolar-processing enzyme precursor [Ricinus communis, EEF50964.1]	catalytic activity
**ACMV-mir-3-4***	target 5' U A U 3'<br>CUCUCUCUCCCA CAAACCCU<br>GAGAGAGAGGGU GUUUGGGA<br>miRNA 3' A UU 5'	-40.8	225	GW613001	similar to conserved hypothetical protein [Ricinus communis, EEF36969.1]	
**ACMV-mir-3-5***	target 5' U U G 3'<br>CUCUCUCUCUC UC AAACCCUA<br>GAGAGAGAGGG AG UUUGGGAU<br>miRNA 3' GU U 5'	-36.1	57	GW613747	similar to ubiquitin-protein ligase [Ricinus communis, EEF51716.1]	catalytic activity
**ACMV-mir-3-6***	target 5' C C U 3'<br>UAUUCUCUUU CCCAUCAAACCCU<br>GUGAGAGAGA GGGUAGUUUGGGA<br>miRNA 3' U 5'	-40.3	244	GT973174	similar to conserved hypothetical protein [Ricinus communis, EEF31424.1]	
	target 5' U G U U 3'<br>CAC C UCUCUCCC UCAAACCCU<br>GUG G AGAGAGGG AGUUUGGGA<br>miRNA 3' U A U 5'	-37	51	GT970166	similar to casein kinase II beta chain [Ricinus communis, EEF35876.1]	catalytic activity

<table>
<tr><th></th><th></th><th></th><th></th><th></th><th></th><th></th></tr>
<tr><td>ACMV-mir-3-7*</td><td><pre>target 5' C   G 3'
           ACGC C CUCUCCCAUCAAGCCC
           UGUG G GAGAGGGUAGUUUGGG
miRNA 3' U   A A   5'</pre></td><td>-41.1</td><td>127</td><td>GW610943</td><td>similar to DNA binding protein [Ricinus communis, EEF33368.1]</td><td>binding</td></tr>
<tr><td>ACMV-mir-3-8*</td><td><pre>target 5' C   C 3'
            ACGC C CUCUCCCAUCAAGCC
            UGUG G GAGAGGGUAGUUUGG
miRNA 3' UU   A A   5'</pre></td><td>-36.9</td><td>127</td><td>GW610943</td><td>similar to DNA binding protein [Ricinus communis, EEF33368.1]</td><td>binding</td></tr>
<tr><td>ACMV-mir-3-9*</td><td><pre>target 5' U   A A   A 3'
           CAAGCAUUUUC CU CCAUUAAAC
           GUUUGUGAGAG GA GGUAGUUUG
miRNA 3'   A G   5'</pre></td><td>-29</td><td>120</td><td>GT976741</td><td>similar to ATP synthase alpha subunit mitochondrial [Ricinus communis, EEF42989.1]</td><td>binding</td></tr>
<tr><td>ACMV-mir-3-10*</td><td><pre>target 5' C   U   C 3'
           UAAGC UUCUUUUUUUCAUCAA
           GUUUG GAGAGAGAGGGUAGUU
miRNA 3'   U   U 5'</pre></td><td>-29</td><td>13</td><td>FM893865</td><td>similar to metallothionein-like MT-3 [Jatropha curcas, ADB02892.1]</td><td>binding</td></tr>
<tr><td>ACMV-mir-3-11*</td><td><pre>target 5' A   C   U 3'
            AAACAUUUUCUCUCCCG CAA
            UUUGUGAGAGAGAGGGU GUU
miRNA 3' AG   A   U 5'</pre></td><td>-33.1</td><td>67</td><td>GW879286</td><td>similar to structural maintenance of chromosome protein [Ricinus communis, EEF30346.1]</td><td>binding</td></tr>
<tr><td></td><td><pre>target 5' C   A A   C 3'
           UCAAGCAUUUUC CU CCAUUAAA
           AGUUUGUGAGAG GA GGUAGUUU
miRNA 3'   A G   5'</pre></td><td>-28</td><td>119</td><td>GT976741</td><td>similar to ATP synthase alpha subunit mitochondrial [Ricinus communis, EEF42989.1]</td><td>binding</td></tr>
<tr><td></td><td><pre>target 5' U   C   U   U 3'
           UCAAAUACUC CUCUCCC UUGA
           AGUUUGUGAG GAGAGGG AGUU
miRNA 3'   A   U   U 5'</pre></td><td>-32.8</td><td>56</td><td>GT976385</td><td>similar to protein phosphatase 2a, regulatory subunit [Ricinus communis, EEF37048.1]</td><td>enzyme regulator activity</td></tr>
</table>

**ACMV-mir-3-12***	target 5' C A A A 3'<br>UCAAGCAUUUUC CU CCAUUAA<br>AGUUUGUGAGAG GA GGUAGUU<br>miRNA 3' A A G 5'	-28.2	119	GT976741	similar to ATP synthase alpha subunit mitochondrial [Ricinus communis, EEF42989.1]	binding
	target 5' C A A U 3'<br>UUCAAA A UUUCUUUCUCAUCG<br>AAGUUU U AGAGAGAGGGUAGU<br>miRNA 3' G G U 5'	-26	62	GT982476	similar to aspartic proteinase precursor [Ricinus communis, EEF32480.1]	catalytic activity
**ACMV-mir-3-13***	target 5' G A A U 3'<br>CUUCAAA A UUUCUUUCUCAUCG<br>GAAGUUU U AGAGAGAGGGUAGU<br>miRNA 3' G G 5'	-28	37	GW614166	similar to aspartic proteinase precursor [Ricinus communis, EEF32480.1]	catalytic activity
**ACMV-mir-3-14***	target 5' G U C 3'<br>AAAC CUCUCUCUCUCAUC<br>UUUG GAGAGAGAGGGUAG<br>miRNA 3' AG U 5'	-32.7	15	GT972003	similar to DELLA protein GAI [Ricinus communis, EEF49067.1]	binding
	target 5' A C G 3'<br>UCAAGCACUCUC UUCCCA<br>AGUUUGUGAGAG GAGGGU<br>miRNA 3' A AG 5'	-33.8	270	GW616565	similar to polygalacturonase [Ricinus communis, EEF46246.1]	catalytic activity
**ACMV-mir-3-15***	target 5' U U 3'<br>UUCUUUCAGACACUCUCUCUUUC<br>AAGGAAGUUUGUGAGAGAGAGGG<br>miRNA 3' U 5'	-40.1	95	GW876074	similar to RING-H2 finger protein ATL51 [Arabidopsis thaliana, Q9SRQ8.2]	binding
	target 5' G C U 3'<br>UCUUCAAAUACUC CUCUCCC<br>GGAAGUUUGUGAG GAGAGGG<br>miRNA 3' AA A U 5'	-37.3	53	GT976385	similar to protein phosphatase 2a, regulatory subunit [Ricinus communis, EEF37048.1]	enzyme regulator activity
	target 5' U U U 3'	-34	69	GW878002	similar to serine-threonine protein kinase [Ricinus communis, EEF43635.1]	binding

	UUCUUUC A UACUCUCUUUCCCA<br>AAGGAAG U GUGAGAGAGAGGGU<br>miRNA 3' U U 5'					
	target 5' U U A 3'<br>UUCUUUC A UACUCUCUUUCCCA<br>AAGGAAG U GUGAGAGAGAGGGU<br>miRNA 3' U U 5'	-34.2	61	GW874613	similar to leucine-rich repeat (LRR) family protein [Zea mays, ACG34018.1]	binding
	target 5' U C U U 3'<br>UUCUUUC A CACUCUCUCUUCU<br>AAGGAAG U GUGAGAGAGAGGG<br>miRNA 3' U U U 5'	-32.4	6	GW620161	similar to cullin-1 [Ricinus communis, EEF45513.1]	catalytic activity
	target 5' C G C 3'<br>UUC UUUAGGUACUCUUUCUCUC<br>AAG AAGUUUGUGAGAGAGAGGG<br>miRNA 3' G U 5'	-30.4	50	GW880193	similar to group II plp decarboxylase [Ricinus communis, EEF29371.1]	catalytic activity
	target 5' C C A 3'<br>UCC UCAAGCAUUCUCUCUCC<br>AGG AGUUUGUGAGAGAGAGG<br>miRNA 3' A A GU 5'	-39	139	GW620165	similar to lactoylglutathione lyase [Ricinus communis, EEF45290]	catalytic activity
**ACMV-mir-3-16***	target 5' U U 3'<br>UUCUUUCAGACACUCUCUCUUUC<br>AAGGAAGUUUGUGAGAGAGAGGG<br>miRNA 3' G 5'	-40.9	95	GW876074	similar to RING-H2 finger protein ATL51 [Arabidopsis thaliana, Q9SRQ8.2]	binding
	target 5' C A 3'<br>UUCUUCAAACAUUUUUUUUCC<br>AGGAAGUUUGUGAGAGAGAGG<br>miRNA 3' GA G 5'	-32.4	30	GW613144	similar to serine/threonine protein phosphatase [Ricinus communis, EEF49903.1]	binding
	target 5' C C A 3'<br>UCC UCAAGCAUUCUCUCUCC	-39	132	GW615921	similar to lactoylglutathione lyase [Ricinus communis, EEF45290]	catalytic activity

	AGG AGUUUGUGAGAGAGAGG<br>miRNA 3' GA A G 5'					
	target 5' C G C 3'<br>CUUC UUUAGGUACUCUUUCUCUC<br>GAAG AAGUUUGUGAGAGAGAGGG<br>miRNA 3' G 5'	-32.7	49	GW880193	similar to group II plp decarboxylase [Ricinus communis, EEF29371.1]	catalytic activity
**ACMV-mir-3-18***	target 5' C U 3'<br>UCUUUCUUUCAGACACUCUCUCU<br>AGGAAGGAAGUUUGUGAGAGAGA<br>miRNA 3' 5'	-40.7	92	GW876074	similar to RING-H2 finger protein ATL51 [Arabidopsis thaliana, Q9SRQ8.2]	binding
	target 5' C G A A 3'<br>UCCUUCCUUUAGA A UCUCUC<br>AGGAAGGAAGUUU U AGAGAG<br>miRNA 3' G G A 5'	-33.7	251	GW878471	similar to protein binding protein [Ricinus communis, EEF47573.1]	binding
**ACMV-mir-3-19***	target 5' C U 3'<br>UCUUUCUUUCAGACACUCUCUCU<br>AGGAAGGAAGUUUGUGAGAGAGA<br>miRNA 3' C 5'	-41.2	92	GW876074	similar to RING-H2 finger protein ATL51 [Arabidopsis thaliana, Q9SRQ8.2]	binding
	target 5' U U G G 3'<br>UC CUUCCUUCAAAUGCUU CUCU<br>AG GAAGGAAGUUUGUGAG GAGA<br>miRNA 3' C A 5'	-33.8	44	GW877934	similar to nuclear transcription factor Y subunit A-1 [Ricinus communis, EEF48902.1]	nucleic acid binding transcription factor activity
	target 5' U A C A 3'<br>GUCCU CCUUCGAGC UUUUCUC<br>CAGGA GGAAGUUUG GAGAGAG<br>miRNA 3' A U A 5'	-33.6	273	GW881391	similar to cationic amino acid transporter [Populus trichocarpa, EEE88357.1]	transporter activity
**ACMV-mir-4-1**	target 5' G C G C U 3'<br>AGCG A AUC AAGCCUAUGUAUU	-29.3	569	GW875401	similar to hypersensitive-induced response protein 1 [Vitis vinifera]	

	`UCGU U UGG UUCGGGUACAUGG`<br>`miRNA  3'      A G   A               5'`					
	`target 5' G  U        G             A 3'`<br>`AG CAUAUGCU GAGUUUAUGUGCC`<br>`UC GUAUGUGG UUCGGGUACAUGG`<br>`miRNA  3'             A               5'`	-32.4	274	GW618449	similar to serine carboxypeptidase [Ricinus communis, EEF35107.1]	catalytic activity
**ACMV-mir-4-3**	`target 5'  A      U             C  U 3'`<br>`UAAGUG UG ACCUAAGCCC UG`<br>`GUUCGU AU UGGAUUCGGG AC`<br>`miRNA  3' AC         G          U    5'`	-26.6	458	GT981264	similar to ribosomal protein L2 (mitochondrion) [Phoenix dactylifera, AEM43912.1]	structural molecule activity
**ACMV-mir-4-4**	`target 5' C     C    A           C 3'`<br>`UGUAA UGUA GCCUAAGCCCA`<br>`ACGUU GUAU UGGAUUCGGGU`<br>`miRNA  3' A     C    G             5'`	-31.2	35	GH296295	similar to 14 kDa proline-rich protein DC2.15 precursor [Ricinus communis, EEF46942.1]	
	`target 5' C  G                  G 3'`<br>`UG AAGCAUGUGUCUAAGCUU`<br>`AC UUCGUAUGUGGAUUCGGG`<br>`miRNA  3' A  G                  U 5'`	-26.5	44	FM887259	similar to sucrose synthase [Ricinus communis, EEF45577.1]	catalytic activity
**ACMV-mir-4-5**	`target 5' G                C   A 3'`<br>`UUGCAAGCAUGUGUCU AGC`<br>`AACGUUCGUAUGUGGA UCG`<br>`miRNA  3'                  U     5'`	-25.5	261	GW615281	similar to 60S ribosomal protein L44 [Ricinus communis, EEF46746.1]	structural molecule activity
	`target 5' G             G     A 3'`<br>`UGCAGGUAUAUAU UAGGC`<br>`ACGUUCGUAUGUG AUUCG`<br>`miRNA  3' A             G       5'`	-30.5	422	GW614133	similar to acyl-CoA:diacylglycerol acyltransferase 2 [Jatropha curcas, AEZ56254.1]	catalytic activity
**ACMV-mir-4-6***	`target 5'  A     U      U         U 3'`<br>`AAGGU UUGGGU UUGUGUGUA`<br>`UUUCA GACCCG AGCAUACAU`	-27.2	378	GW611728	similar to lysine-specific histone demethylase 1 homolog 1-like [Glycine max, XP_003533370.1]	binding

	miRNA 3' GG U 5'					
	target 5' G G 3'<br>AAAGUGCUGGGCUCGUGUG<br>UUUCAUGACCCGAGCAUAC<br>miRNA 3' GG AU 5'	-40.3	388	GT980294	similar to trytophan synthase alpha subunit [Ricinus communis, EEF41392.1]	catalytic activity
	target 5' G A G 3'<br>AAAG UACUGGGCUUGUG GUG<br>UUUC AUGACCCGAGCAU CAU<br>miRNA 3' GG A 5'	-31.4	436	GT975026	similar to Aminotransferase ybdL [Ricinus communis, EEF48365.1]	catalytic activity
**ACMV-mir-4-7***	target 5' G G 3'<br>AAAGUGCUGGGCUCGUGU<br>UUUCAUGACCCGAGCAUA<br>miRNA 3' GG 5'	-37.1	388	GT980294	similar to trytophan synthase alpha subunit, [Ricinus communis, EEF41392.1]	catalytic activity
	target 5' U A G 3'<br>CUAAAGUACUG GUUUGUA<br>GGUUUCAUGAC CGAGCAU<br>miRNA 3' C A 5'	-27	303	GW879408	similar to ribosome biogenesis protein nop10, [Ricinus communis, EEF32551.1]	
	target 5' G A G 3'<br>AAAGUGCUGG CUCGUGU<br>UUUCAUGACC GAGCAUA<br>miRNA 3' GG C 5'	-30.1	388	GT970365	similar to trytophan synthase alpha subunit, [Ricinus communis, EEF41392.1]	catalytic activity
	target 5' A U U A 3'<br>UC AAGUACU GGCUUGUG<br>GG UUCAUGA CCGAGCAU<br>miRNA 3' U C A 5'	-26.2	525	GW879628	similar to glutathione-s-transferase theta protein	catalytic activity
**ACMV-mir-5-1**	target 5' A C 3'<br>UUUAGACGAGGAAGGGCGGG<br>GGAUCUGCUCCUUUUCGUUC<br>miRNA 3' AA U 5'	-34.8	545	GW620145	similar to AT-rich interactive domain-containing protein 2 [Vitis vinifera]	binding

**ACMV-mir-5-2**	target 5' C A A A 3'<br>UUCC AGGC GAGGAAGAGCGAGA<br>AAGG UCUG CUCCUUUUCGUUCU<br>miRNA 3' C A 5'	-37.3	177	GT973192	similar to conserved hypothetical protein [Ricinus communis, XP_002519153.1]	
**ACMV-mir-5-3**	target 5' A G U U 3'<br>AGUU CUA GACGAGGGAAAGCGAG<br>UCAA GAU CUGCUCCUUUUCGUUC<br>miRNA 3' G 5'	-38.5	625	GW611228	similar to dtdp-glucose 4-6-dehydratase [Ricinus communis, EEF33919.1]	catalytic activity
	target 5' A A C 3'<br>UUUUUGGG GAGGAAAAGCAG<br>AAGGAUCU CUCCUUUUCGUU<br>miRNA 3' UC G C 5'	-29.3	307	GW880768	similar to palmitoyltransferase ZDHHC9 [Ricinus communis, EEF51726.1]	catalytic activity
	target 5' U A C C 3'<br>GG UCC AGAUGAGGGAGAGUGA<br>UC AGG UCUGCUCCUUUUCGUU<br>miRNA 3' A A C 5'	-31.8	162	GT972305	similar to serine/threonine protein phosphatase 2a regulatory subunit A [Ricinus communis, EEF33251.1]	binding
**ACMV-mir-5-4**	target 5' U A C C 3'<br>GG UCC AGAUGAGGGAGAGUGA<br>UC AGG UCUGCUCCUUUUCGUU<br>miRNA 3' C A A 5'	-31.8	365	GW614802	similar to serine/threonine protein phosphatase 2a regulatory subunit A [Ricinus communis, EEF33251.1]	binding
	target 5' A A C C 3'<br>GGG UCCU GAUGGGGAAGAGC<br>CUC AGGA CUGCUCCUUUUCG<br>miRNA 3' A U UU 5'	-33	561	GW615568	similar to Beta-glucosidase [Ricinus communis, EEF42691.1]	binding
	target 5' A G U G 3'<br>AGUU CUA GACGAGGGAAAGCGA<br>UCAA GAU CUGCUCCUUUUCGUU<br>miRNA 3' C G 5'	-36.7	625	GW611228	similar to dtdp-glucose 4-6-dehydratase [Ricinus communis, EEF33919.1]	catalytic activity
	target 5' C A C 3'	-29.8	256	GW611675	similar to copine [Ricinus communis, EEF49756.1]	binding

	AGUUUCUGGA G AGGAAGAGUA<br>UCAAGGAUCU C UCCUUUUCGU<br>miRNA 3' C G U 5'					
	target 5' G U A U 3'<br>GAGUUUUUGG A GAG AAGAGCAA<br>CUCAAGGAUC U CUC UUUUCGUU<br>miRNA 3' G C 5'	-25.3	159	GT979942	similar to importin alpha [Ricinus communis, lEEF49980.1]	transporter activity
**ACMV-mir-5-5**	target 5' A A A 3'<br>AGAGUU CUAGAUGGGGAAGAG<br>UCUCAA GAUCUGCUCCUUUUC<br>miRNA 3' G 5'	-34.9	44	GW612818	similar to phospholipase D [Jatropha curcas, ADA72022.1]	binding
**ACMV-mir-5-6**	target 5' U A A G 3'<br>GAG U UUAGAUGAGGAAAAG<br>CUC A GAUCUGCUCCUUUUC<br>miRNA 3' UU A G 5'	-28.4	81	GW612818	similar to phospholipase D [Jatropha curcas, ADA72022.1]	binding
	target 5' C A G G 3'<br>AGGGUUUC AGA GGGGAAGAG<br>UCUCAAGG UCU CUCCUUUUC<br>miRNA 3' U A G 5'	-31.9	528	GT971019	similar to 50S ribosomal protein L15 [Ricinus communis, EEF30749.1]	structural molecule activity
**ACMV-mir-5-7***	target 5' A A G 3'<br>CCUCUGUAAUUCG GAUCUGCC<br>GGAGACGUUAGGU CUGGAUGG<br>miRNA 3' C AG 5'	-37.2	555	GW611710	similar to auxin-repressed protein-like protein ARP1 [Jatropha curcas, ADB02903.1]	
	target 5' A U G 3'<br>CUUU UG UCCAGGACUUAUU<br>GAGA GU AGGUCCUGGAUGG<br>miRNA 3' G C U AG 5'	-26	302	GW613935	similar to 1L-myo-inositol 1-phosphate synthase [Jatropha curcas, ABO93456.1]	catalytic activity
**ACMV-mir-5-8***	target 5' A A C 3'<br>CCUCUGUAAUUCG GAUCUGC	-33.9	408	GT979423	similar to auxin-repressed protein-like protein ARP1 [Jatropha curcas, ADB02903.1]	

	GGAGACGUUAGGU CUGGAUG<br>miRNA 3' AA C 5'					
**ACMV-mir-5-10***	target 5' G G G A 3'<br>GUU UUUUCUGCGA CCAGGGCC<br>UAG AGGAGACGUU GGUCCUGG<br>miRNA 3' A A 5'	-35.2	364	GW611682	similar to inorganic pyrophosphatase [Ricinus communis, EEF47080.1]	catalytic activity
**ACMV-mir-5-11***	target 5' U C A 3'<br>UUAUCU UCUCUGCAAUCUA<br>GAUAGA GGAGACGUUAGGU<br>miRNA 3' GU A C 5'	-30.7	165	GW877380	similar to guanine nucleotide-binding protein beta [Ricinus communis, EEF50865.1]	catalytic activity
	target 5' C A A 3'<br>CUAUCUUCCUU GCAAUCUA<br>GAUAGAAGGAG CGUUAGGU<br>miRNA 3' GU A C 5'	-32.7	99	GT976156	similar to ubiquitin ligase SINAT2 [Ricinus communis, EEF28013.1]	catalytic activity
**ACMV-mir-5-12***	target 5' A A A 3'<br>UC CUAUCUUCCUU GCAAUCUA<br>GG GAUAGAAGGAG CGUUAGGU<br>miRNA 3' G U A 5'	-33.8	97	GT976156	similar to ubiquitin ligase SINAT2 [Ricinus communis, EEF28013.1]	catalytic activity
	target 5' U U C A 3'<br>CCU UUAUCU UCUCUGCAAUCUA<br>GGG GAUAGA GGAGACGUUAGGU<br>miRNA 3' U A 5'	-33.6	136	GW876402	similar to guanine nucleotide-binding protein beta [Ricinus communis, EEF50865.1]	catalytic activity
	target 5' U U G A 3'<br>UC CUGUCUUCCUUUGUGAUC G<br>GG GAUAGAAGGAGACGUUAG U<br>miRNA 3' G U G 5'	-32.6	105	GT976828	similar to cysteine protease inhibitor [Ricinus communis, EEF36811.1]	catalytic activity
**ACMV-mir-5-13***	target 5' C U C A 3'<br>UCCU UUAUCU UCUCUGCAAUCU<br>AGGG GAUAGA GGAGACGUUAGG	-34.9	160	GW877380	similar to guanine nucleotide-binding protein beta [Ricinus communis, EEF50865.1]	catalytic activity

	miRNA 3' U A 5'					
	target 5' U U G 3'<br>UUUC CUGUCUUCCUUUGUGAUC<br>AGGG GAUAGAAGGAGACGUUAG<br>miRNA 3' U G 5'	-33.5	103	GT979824	similar to cysteine protease inhibitor [Ricinus communis, EEF36811.1]	enzyme regulator activity
**ACMV-mir-5-14***	target 5' C U C U 3'<br>UCCU UUAUCU UCUCUGCAAUC<br>AGGG GAUAGA GGAGACGUUAG<br>miRNA 3' U A 5'	-33.3	135	GW876402	similar to guanine nucleotide-binding protein beta [Ricinus communis, EEF50865.1]	catalytic activity
	target 5' U U G 3'<br>UUUC CUGUCUUCUUUUGUGAUC<br>AGGG GAUAGAAGGAGACGUUAG<br>miRNA 3' U 5'	-30.8	103	GT976828	similar to cysteine protease inhibitor [Ricinus communis, EEF36811.1]	enzyme regulator activity
**ACMV-mir-5-15***	target 5' C U G 3'<br>UUUUC CUGUCUUCCUUUGUGAUC<br>AAGGG GAUAGAAGGAGACGUUAG<br>miRNA 3' U 5'	-31.6	102	GT977661	similar to cysteine protease inhibitor [Ricinus communis, EEF36811.1]	enzyme regulator activity
	target 5' U G 3'<br>UUUCCAUUGUUUUCUUCUGCAG<br>AAGGGUGAUAGAAGGAGACGUU<br>miRNA 3' AG 5'	-35.4	471	GT228564	similar to ethylene-responsive transcription factor [Jatropha curcas, AEJ87198.1]	nucleic acid binding transcription factor activity
	target 5' C U U C 3'<br>UC CUACU UCUUC CUUUGUGGUU<br>AG GGUGA AGAAG GAGACGUUAG<br>miRNA 3' A U 5'	-26.2	12	GW619393	similar to translationally controlled tumor protein [Jatropha curcas, ABO25950.1]	binding
**ACMV-mir-6-1**	target 5' C U C 3'<br>AAG UUUCGUAUUGCUGCCU<br>UUC AGAGUAUAACGACGGA<br>miRNA 3' U C 5'	-31.3	88	GT972735	similar to DNA binding protein [Ricinus communis, EEF38786.1]	binding

	target 5' C U C 3'<br>AAGGUCUC UAUUGCUGCU<br>UUCCAGAG AUAACGACGG<br>miRNA 3' U U A 5'	-33.6	115	FM890468	similar to triose phosphate/phosphate translocator, non-green plastid, chloroplast [Ricinus communis, EEF51988.1]	transporter activity
	target 5' G C 3'<br>AAAGG CUUAUAUUGCUGCCU<br>UUUCC GAGUAUAACGACGGA<br>miRNA 3' A 5'	-34.6	156	GT972267	similar to WD-repeat protein [Ricinus communis, EEF30258.1]	
	target 5' C U A 3'<br>AAGGUUUUAUGUUGUU CC<br>UUCCAGAGUAUAACGA GG<br>miRNA 3' U C A 5'	-26.8	474	GW618981	similar to nutrient reservoir [Ricinus communis, EEF31271.1]	nutrient reservoir activity
**ACMV-mir-6-2**	target 5' C C U U 3'<br>CU AAG UUUCGUAUUGCUGCC<br>GG UUC AGAGUAUAACGACGG<br>miRNA 3' CA U C 5'	-31.7	85	GT972735	similar to DNA binding protein [Ricinus communis, EEF38786.1]	binding
**ACMV-mir-6-3**	target 5' G U A C G 3'<br>CUGG UUUGGAGG CUC UGUUGCU<br>GAUC AGGUUUCC GAG AUAACGA<br>miRNA 3' A U 5'	-27.1	474	GW877257	similar to conserved hypothetical protein [Ricinus communis, EEF40706.1]	
**ACMV-mir-6-5**	target 5' U A C 3'<br>GA ACCUGGACCUGGUUCAGAG<br>CU UGGACCUGGAUCAGGUUUC<br>miRNA 3' AC G 5'	-39.1	176	FM890649	similar to glucan endo-1,3-beta-glucosidase precursor [Ricinus communis, EEF52560.1]	binding
	target 5' G U A G U 3'<br>UGG ACACC GGA CUGGUCCAAAG<br>ACC UGUGG CCU GAUCAGGUUUC<br>miRNA 3' A G 5'	-40	241	FM892996	similar to 60S ribosomal protein L18 [Ricinus communis, EEF39291.1]	structural molecule activity

	target 5' G U 3'<br>UGGAUACUUGGGUCUA UCCAAA<br>ACCUGUGGACCUGGAU AGGUUU<br>miRNA 3' C C 5'	-33.4	205	GW875881	similar to 4-alpha-glucanotransferase [Ricinus communis, EEF38704.1]	catalytic activity
	target 5' U A C 3'<br>GAUACC GGAUUUGGUUCAAG<br>CUGUGG CCUGGAUCAGGUUU<br>miRNA 3' AC A C 5'	-31.2	299	GW611572	similar to auxilin [Ricinus communis, EEF42106.1]	
**ACMV-mir-6-6**	target 5' A U A U 3'<br>AUGU GACAU UGGACUUGGUU<br>UACA CUGUG ACCUGGAUCAG<br>miRNA 3' GA C G 5'	-31.1	351	GT971958	similar to pectinesterase-2 precursor [Ricinus communis, EEF51542.1]	enzyme regulator activity
**ACMV-mir-6-7***	target 5' C C 3'<br>AGAUCUUUAGGCCUG AUAA<br>UCUAGAAAUCCGGGU UGUU<br>miRNA 3' G 5'	-28.7	316	GW616902	similar to tubulin beta chain [Ricinus communis, EEF39168.1]	structural molecule activity
	target 5' C A 3'<br>GAUCUUUAGGCCC C CAA<br>CUAGAAAUCCGGG G GUU<br>miRNA 3' U U U 5'	-30.8	357	GW879298	similar to aldose 1-epimerase [Ricinus communis, EEF50625.1]	binding
**ACMV-mir-6-8***	target 5' U U A C U 3'<br>UGG GU UG GUCUUUGGGUCUAU<br>ACC CG GU UAGAAAUCCGGGUG<br>miRNA 3' U C U 5'<br>miRNA 3' C U C 5'	-25.8	431	FM889880	similar to nonspecific lipid-transfer protein precursor [Ricinus communis, EEF29523.1]	binding
**ACMV-mir-6-9***	target 5' C U A U A 3'<br>CG AUAUGG CUA GUCUUUGGG<br>GC UAUACC GGU UAGAAAUCC<br>miRNA 3' CU C C 5'	-27.1	394	GT978642	similar to adenosine kinase [Ricinus communis, EEF30697.1]	catalytic activity
**ACMV-mir- 6-10***	target 5' C U C G 3'	-27.4	323	GT974250	similar to conserved hypothetical protein [Ricinus communis, EEF43404.1]	

	AAGAUGAUGUGG CU GAUUUU<br>UUCUGCUAUACC GG CUAGAA<br>miRNA 3' CC C U 5'					
**ACMV-mir-6-11***	target 5' A A A 3'<br>GAAGACGAU AU GGCUGGAUC<br>CUUCUGCUA UA CCGGUCUAG<br>miRNA 3' C A 5'	-31.4	231	GW875047	similar to cyclin B [Ricinus communis, EEF52862.1]	
	target 5' U U G 3'<br>GGGUGAU UGGGUCAGAUC<br>UCUGCUA ACCCGGUCUAG<br>miRNA 3' CU U A 5'	-30.4	326	GW613225	similar to NADH-ubiquinone oxidoreductase 39 kD subunit [Ricinus communis, EEF35812.1]	binding
**ACMV-mir-6-12***	target 5' A U 3'<br>AGGCGG GUGGGUCGGGUU<br>UCUGCU UACCCGGUCUAG<br>miRNA 3' A 5'	-28.9	382	GT972304	similar to transferase [Ricinus communis, EEF33042.1]	catalytic activity
	target 5' G U G 3'<br>GGA UGGUG GGGUCGGGUU<br>UCU GCUAU CCCGGUCUAG<br>miRNA 3' A 5'	-25.1	190	GT974377	similar to mitogen activated protein kinase kinase kinase 3 [Ricinus communis, EEF32660.1]	binding
	target 5' U U A 3'<br>GGAUGAUGUGGGC UGGAUU<br>UCUGCUAUACCCG GUCUAG<br>miRNA 3' 5'	-31.1	319	FM894132	similar to cell differentiation protein rcd1 [Ricinus communis, EEF32925.1]	
**ACMV-mir-6-13***	target 5' U A G 3'<br>GGGAAG C AUGUGGGCCAGA<br>CCCUUC G UAUACCCGGUCU<br>miRNA 3' U U C 5'	-38	313	GT976507	similar to dihydropteroate synthase [Ricinus communis, EEF41573.1]	catalytic activity
	target 5' U U U U 3'<br>GGG GAAGAUGA UAUGGGUCAG<br>UCC CUUCUGCU AUACCCGGUC	-35.4	475	GW881211	similar to protein kinase 1b [Solanum lycopersicum, ACD77110.1]	binding

	miRNA 3' U 5'					
**ACMV-mir-6-14***	target 5' C U A A 3'<br>GGGGA A GAUAUGGGCCA<br>CCCUU U CUAUACCCGGU<br>miRNA 3' GU C G 5'	-32.3	541	GT976507	similar to dihydropteroate synthase [Ricinus communis, EEF41573.1]	catalytic activity
	target 5' U A G 3'<br>GGGAAG C AUGUGGGUCA<br>CCCUUC G UAUACCCGGU<br>miRNA 3' GU U C 5'	-30.8	113	GT975582	similar to dihydropteroate synthase [Ricinus communis, EEF41573.1]	catalytic activity
**ACMV-mir-6-16***	target 5' U A U A 3'<br>CAGGAUA GGAGGACGAU UG<br>GUCUUGU CCUUCUGCUA AC<br>miRNA 3' UC C U C 5'	-29.4	460	FM888386	similar to 4-hydroxybenzoate octaprenyltransferase [Ricinus communis, EEF40466.1]	catalytic activity
**ACMV-mir-6-17***	target 5' A G U U 3'<br>AGAA A GGAAGAUGAUAUG<br>UCUU U CCUUCUGCUAUAC<br>miRNA 3' G G C 5'	-28.2	106	FM894086	similar to eukaryotic initiation factor iso-4F subunit p82-34 [Ricinus communis, EEF28321.1]	binding
	target 5' A U C U 3'<br>CAGAACA GGG AGACGAUAUG<br>GUCUUGU CCC UCUGCUAUAC<br>miRNA 3' U 5'	-33.8	464	GT981069	similar to heterogeneous nuclear ribonucleoprotein [Ricinus communis, EEF48904.1]	binding
	target 5' G A U C 3'<br>AGAA AGGGGA GCGGUAUG<br>UCUU UCCCUU UGCUAUAC<br>miRNA 3' G G C 5'	-27.3	602	GW616227	similar to ATP-dependent Clp protease proteolytic subunit [Ricinus communis, EEF43288.1]	binding
**ACMV-mir-6-18***	target 5' C A G U 3'<br>AGUAG A GCGGGGGAGGC<br>UCGUC U UGUCCCUUCUG	-26.4	17	FM887314	similar to chlorophyll A/B binding protein [Ricinus communis, EEF34284.1]	

	miRNA 3' CU 5'					
**ACMV-mir-7-1**	target 5' U G G 3'<br>GGU GUCUCUGGA GGUGUU<br>CCA CAGAGAUUU CCGUAA<br>miRNA 3' U A GA 5'	-26.2	423	GW611021	similar to O-sialoglycoprotein endopeptidase [Ricinus communis, EEF50357.1]	catalytic activity
	target 5' G A A A 3'<br>AGG GUU UUUAGAUGGCAUUUU<br>UCC CAG AGAUUUACCGUAAGA<br>miRNA 3' A 5'	-27	316	FM893119	similar to ribosomal protein L39e [Ricinus communis, EEF38862.1]	structural molecule activity
**ACMV-mir-7-2***	target 5' A C U G 3'<br>AG AUAUUC GCUGGAGAUAUU<br>UC UGUGAG UGAUCUCUGUGA<br>miRNA 3' U GA 5'	-26.8	572	GW616486	similar to protein phosphatase 2c [Ricinus communis, EEF46865]	binding
	target 5' G G A 3'<br>AGGCAUUCA GCUGGAG CAUUU<br>UCUGUGAGU UGAUCUC GUGAG<br>miRNA 3' U A 5'	-30.1	588	GW612007	similar to ATP-citrate synthase [Ricinus communis, EEF43093.1]	binding
	target 5' U C U 3'<br>GGCAU CAACUA AGACAUUUU<br>CUGUG GUUGAU UCUGUGAGA<br>miRNA 3' U A C 5'	-27.2	551	GT977103	similar to WD-repeat protein [Ricinus communis, EEF33655.1]	
	target 5' A G A G 3'<br>AGG ACUCAAUUAGAGA ACUU<br>UCU UGAGUUGAUCUCU UGAG<br>miRNA 3' G G A 5'	-31.2	456	GT972310	similar to Beta-1,3-galactosyltransferase sqv-2 [Ricinus communis, EEF43468.1]	catalytic activity
	target 5' G U U 3'<br>GGAU CUU GCUGGAGGUGCUCU<br>UCUG GAG UGAUCUCUGUGAGA<br>miRNA 3' U U 5'	-25.8	400	GW611965	similar to chloroplast photosystem II 10 kDa polypeptide [Jatropha curcas, ADB93062.1]	

	target 5' A A G 3'<br>GCAUUUAACUG AGACAUU<br>UGUGAGUUGAU UCUGUGA<br>miRNA 3' UC C GA 5'	-25.7	34	GW611883	similar to DNA repair helicase rad5,16 [Ricinus communis, EEF37546.1]	binding
	target 5' G G 3'<br>GGAUGCUU GCUGGAGGUACU<br>UCUGUGAG UGAUCUCUGUGA<br>miRNA 3' U GA 5'	-26.6	455	GW611811	similar to NADH-cytochrome B5 reductase [Ricinus communis, EEF34822.1]	catalytic activity
	target 5' G A A 3'<br>AGAUAC CAGCUGGA AUGUUCU<br>UCUGUG GUUGAUCU UGUGAGA<br>miRNA 3' A C 5'	-25.7	97	GW611781	similar to mitochondrial carrier protein [Ricinus communis, EEF32670.1]	binding
	target 5' A A 3'<br>AGAU CUUGGCU GAGGCAUUC<br>UCUG GAGUUGA CUCUGUGAG<br>miRNA 3' U U A 5'	-27.3	81	GW612998	similar to mom(plant) [Ricinus communis, EEF32941.1]	binding
**ACMV-mir-7-3***	target 5' C A 3'<br>AUAUUCAACUAGGGACA<br>UGUGAGUUGAUCUCUGU<br>miRNA 3' UC GA 5'	-29.2	435	GW611798	similar to lipoxygenase [Ricinus communis, EEF49219.1]	binding
	target 5' U A A 3'<br>GACAUU AAUUGGGGACAC<br>CUGUGA UUGAUCUCUGUG<br>miRNA 3' U G A 5'	-28.6	84	GO246773	similar to ubiquitin-conjugating enzyme E2 [Ricinus communis, EEF51304.1]	binding
	target 5' A A C 3'<br>AGGC C CGGCUGGAGACAC<br>UCUG G GUUGAUCUCUGUG<br>miRNA 3' U A A 5'	-28.8	53	GW611924	similar to heat shock protein [Ricinus communis, EEF34649.1]	binding
	target 5' G G 3'	-26.2	455	GW611811	similar to NADH-cytochrome B5 reductase [Ricinus communis, EEF34822.1]	catalytic activity

	GGAUGCUUG CUGGAGGUACU<br>UCUGUGAGU GAUCUCUGUGA<br>miRNA 3' U 5'					
	target 5' U U C 3'<br>AGA CAUUCAGCUGGAGAUAU<br>UCU GUGAGUUGAUCUCUGUG<br>miRNA 3' A 5'	-31.1	273	GW612528	similar to afc [Ricinus communis, EEF51903.1]	binding
	target 5' G A A 3'<br>AGA ACUCAACU AGAGGCGC<br>UCU UGAGUUGA UCUCUGUG<br>miRNA 3' G A 5'	-29.9	1	FM887683	similar to legumin B precursor [Ricinus communis, AAF73007.1]	nutrient reservoir activity
	target 5' A U A 3'<br>AGGC UUCAA UGGAGAUACU<br>UCUG GAGUU AUCUCUGUGA<br>miRNA 3' U G 5'	-26	378	FM888640	similar to zinc finger protein [Ricinus communis, EEF35988.1]	binding
**EACMV-UG-mir-1-1**	target 5' C A U 3'<br>ACAGAUCCC UUUGUUUCGAGA<br>UGUUUAGGG AGAUAAAGCUUU<br>miRNA 3' AU G 5'	-31.4	2	GT970645	similar to vesicle-associated membrane protein [Ricinus communis, EEF33458.1]	structural molecule activity
	target 5' G A G 3'<br>UAAUGGAUCCUUUCUAUU CGAAG<br>AUUGUUUAGGGGAGAUAA GCUUU<br>miRNA 3' A 5'	-29.1	404	GT969638	similar to JHL23C09.2 [Jatropha curcas, AJ53210.1]	binding
	target 5' U C C G 3'<br>UAACGGGUCCUC CU UUUUGAGA<br>AUUGUUUAGGGG GA AAAGCUUU<br>miRNA 3' A U 5'	-27.3	644	GT969497	similar to 14.3 kDa OLEO1 [Jatropha curcas]	
**EACMV-UG-mir-1-2**	target 5' U G U 3'<br>GUAACAAAUCU CUUUGUUUCGA<br>UAUUGUUUAGG GAGAUAAAGCU	-33.2	195	GW881131	similar to serine/threonine protein kinase [Ricinus communis, EEF39636.1]	binding

	miRNA 3' G G 5'					
	target 5' A A G C 3'<br>AUGAUGAGUCC U UCUGUUUUG<br>UAUUGUUUAGG G AGAUAAAGC<br>miRNA 3' G G U 5'	-25.1	569	GW611093	similar to DNA photolyase [Ricinus communis, EEF39277.1]	catalytic activity
	target 5' A G G A 3'<br>AUGA A GAUUCUCUCUGUUUUGG<br>UAUU U UUAGGGGAGAUAAAGCU<br>miRNA 3' G G 5'	-26.2	249	GW613407	similar to tetratricopeptide repeat protein, tpr [Ricinus communis, EEF52668.1]	binding
	target 5' C U G G 3'<br>CAUGAC G UCCU UCUGUUUUGA<br>GUAUUG U AGGG AGAUAAAGCU<br>miRNA 3' U U G 5'	-27.3	288	GW613541	similar to zinc finger protein [Ricinus communis, EEF42871.1]	binding
**EACMV-UG-mir-1-3**	target 5' U G U 3'<br>GUAACAAAUCU CUUUGUUUCGA<br>UAUUGUUUAGG GAGAUAAAGCU<br>miRNA 3' G 5'	-32.5	195	GW881131	similar to serine/threonine protein kinase [Ricinus communis, EEF39636.1]	binding
	target 5' A U U 3'<br>AACA AUCUCUUUUGUUUUGA<br>UUGU UAGGGGAGAUAAAGCU<br>miRNA 3' UA U 5'	-26.6	446	GT981649	similar to 60S acidic ribosomal protein P2 (RPP2D)	structural molecule activity
	target 5' A A A 3'<br>GUAAUGGAUCCUUUCUAUU CGA<br>UAUUGUUUAGGGGAGAUAA GCU<br>miRNA 3' A 5'	-29.1	85	GT969463	similar to JHL23C09.2 [Jatropha curcas, AJ53210.1]	
**EACMV-UG-mir-1-4**	target 5' U G A 3'<br>GUAACAAAUCU CUUUGUUUCG<br>UAUUGUUUAGG GAGAUAAAGC<br>miRNA 3' G 5'	-30.6	195	GW881131	similar to serine/threonine protein kinase [Ricinus communis, EEF39636.1]	binding

**EACMV-UG-mir-1-5**	target 5' U G G 3'<br>AUGUAACAAAUCU CUUUGUUUC<br>UGUAUUGUUUAGG GAGAUAAAG<br>miRNA 3' G 5'	-29.9	193	GW881131	similar to serine/threonine protein kinase [Ricinus communis, EEF39636.1]	binding
**EACMV-UG-mir-1-6**	target 5' U G C 3'<br>AUGUAACAAAUCU CUUUGUUU<br>UGUAUUGUUUAGG GAGAUAAA<br>miRNA 3' C G 5'	-26	193	GW881131	similar to serine/threonine protein kinase [Ricinus communis, EEF39636.1]	binding
	target 5' A U G 3'<br>AUAU ACAGAUUCCUUUUGUUU<br>UGUA UGUUUAGGGGAGAUAAA<br>miRNA 3' C U 5'	-25.3	538	FM890970	similar to LEC14B protein [Ricinus communis, EEF45451.1]	
**EACMV-UG-mir-1-7**	target 5' U G U 3'<br>AUGUAACAAAUCU CUUUGUU<br>UGUAUUGUUUAGG GAGAUAA<br>miRNA 3' C G 5'	-25.1	193	GW881131	similar to serine/threonine protein kinase [Ricinus communis, EEF39636.1]	binding
	target 5' A G 3'<br>ACAUAAUGGAUUUCUUCUAUU<br>UGUAUUGUUUAGGGGAGAUAA<br>miRNA 3' C 5'	-26.5	4	FM893965	similar to dimethyladenosine transferase [Ricinus communis, EEF33247.1]	catalytic activity
**EACMV-UG-mir-1-8**	target 5' A U C 3'<br>GGGC UGAUGAAUUCUCUCUA<br>CCUG AUUGUUUAGGGGAGAU<br>miRNA 3' AC U A 5'	-29	211	GW880718	similar to nonsense-mediated mRNA decay protein [Ricinus communis, EEF33616.1]	binding
	target 5' U U C 3'<br>UGGGGC AUAAC AG CUCCUUUGU<br>ACCCUG UAUUG UU GGGGAGAUA	-27.7	396	FM888943	similar to biotin carboxyl carrier protein of acetyl-CoA carboxylase [Ricinus communis, EEF41512.1]	catalytic activity

	miRNA 3' U A 5'					
**EACMV-UG-mir-1-9**	target 5' A G A C 3'<br>GGA A GACAGAUCCUCUCU<br>CCU U UUGUUUAGGGGAGA<br>miRNA 3' G A 5'	-29.2	100	GT969953	similar to sphingolipid delta 4 desaturase/C-4 hydroxylase protein des2 [Ricinus communis, EEF43254.1]	catalytic activity
	target 5' A U A 3'<br>GGGC UGAUGAAUUCUCUCU<br>CCUG AUUGUUUAGGGGAGA<br>miRNA 3' U 5'	-26.8	211	GW880718	similar to nonsense-mediated mRNA decay protein [Ricinus communis, EEF33616.1]	binding
	target 5' U A G 3'<br>GGACAUAGC AGAUUCUUUUU<br>CCUGUAUUG UUUAGGGGAGA<br>miRNA 3' 5'	-25.4	611	GT980733	similar to nibrin [Ricinus communis, EEF36478.1]	
	target 5' U U A 3'<br>GG UAUAGCAAAUCCUUUC<br>CC GUAUUGUUUAGGGGAG<br>miRNA 3' U A 5'	-26.9	168	GT971758	similar to alpha-galactosidase-like isoform 1 [Glycine max, XP_003526810.1]	catalytic activity
**EACMV-UG-mir-1-10**	target 5' A G C 3'<br>UAUC GGGA CGUGGCGGAUUUC<br>AUGG CCCU GUAUUGUUUAGGG<br>miRNA 3' A A 5'	-26.5	83	FM896764	similar to cytidine deaminase 1, 2, 7 [Ricinus communis, EEF48094.1]	catalytic activity
	target 5' C C C G 3'<br>UUGCCUGGG CG A GCGGGUCCU<br>AAUGGACCC GU U UGUUUAGGG<br>miRNA 3' U A 5'	-29.1	102	FM894216	similar to cop9 complex subunit 7a [Ricinus communis, EEF28842.1]	
	target 5' A A U G 3'<br>UGCUUG GGAUGUAGUG AUCCC<br>AUGGAC CCUGUAUUGU UAGGG<br>miRNA 3' A U 5'	-27.5	443	FM894211	similar to 40S ribosomal protein S7 [Ricinus communis, EEF30900.1]	structural molecule activity

-26.5 | 102 | FM890057 | similar to type 2A phosphatase activator TIP41 [Ricinus communis, EEF34869.1]

```
target 5'  A       A              U 3'
            ACUUGGG ACAU AUAAAUCUU
            UGGACCC UGUA UGUUUAGGG
miRNA  3' AA            U           5'
```

EACMV-UG-mir-1-11

-28.3 | 482 | GW616967 | similar to hydrolase, hydrolyzing O-glycosyl compounds [Ricinus communis, EEF29960.1] | binding

```
target 5' A                    G 3'
           UUGCUUGGGAUGUAAUGAAU
           AAUGGACCCUGUAUUGUUUA
miRNA  3'                        5'
```

-25.3 | 156 | GW611801 | similar to wound-induced protein WIN1 precursor [Ricinus communis, EEF31099.1]

```
target 5' C           C       C 3'
           UACUUGGGAUG UGGCAAG
           AUGGACCCUGU AUUGUUU
miRNA  3' A                   A 5'
```

-27.6 | 547 | GW611716 | similar to WD-repeat protein [Ricinus communis, EEF31490.1]

```
target 5' A            A      G 3'
           UUGCUUGGGGCG AGCAAG
           AAUGGACCCUGU UUGUUU
miRNA  3'              A      A 5'
```

EACMV-UG-mir-1-12*

-32.1 | 142 | GW881300 | similar to defective in cullin neddylation protein [Ricinus communis, EEF35070.1] | binding

```
target 5'  C   U     C            A 3'
            UUU UCGCU CGCCUCAAGUAA
            GAG AGUGA GCGGAGUUCGUU
miRNA  3' UU   U     C              5'
```

EACMV-UG-mir-1-13*

-31.2 | 240 | GW612570 | similar to conserved hypothetical protein [Ricinus communis, EEF44682.1]

```
target 5' A           A  G        C 3'
           GGCUCAUCGUU UG CUCAAGCA
           UUGAGUAGUGA GC GAGUUCGU
miRNA  3' C           C  G          5'
```

-34.8 | 505 | GW613376 | similar to conserved hypothetical protein [Ricinus communis, EEF52573.1]

```
target 5' G       U                 A 3'
           GAAUUCG UCGUUGCGUUUCAGGCA
           CUUGAGU AGUGACGCGGAGUUCGU
```

	miRNA 3' 5'					
**EACMV-UG-mir-1-14***	target 5' A A G A 3'<br>GGCUCAUCGUU UG CUCAAGC<br>UUGAGUAGUGA GC GAGUUCG<br>miRNA 3' CC C G 5'	-30.3	240	GW612570	similar to conserved hypothetical protein [Ricinus communis, EEF44682.1]	
	target 5' A U A 3'<br>GGAAUUCG UCGUUGCGUUUCAGGC<br>CCUUGAGU AGUGACGCGGAGUUCG<br>miRNA 3' 5'	-37.1	504	GW613376	similar to conserved hypothetical protein [Ricinus communis, EEF52573.1]	
**EACMV-UG-mir-1-15***	target 5' G U G 3'<br>UUAUUACUGC GCCUCAAGU<br>AGUAGUGACG CGGAGUUCG<br>miRNA 3' G 5'	-34.6	19	FM889529	similar to gc-rich DNA-binding factor [Ricinus communis, EEF49657.1]	nucleic acid binding transcription factor activity
	target 5' U A C A 3'<br>UCAU AUUGC UUUCAAGC<br>AGUA UGACG GGAGUUCG<br>miRNA 3' G G C 5'	-25	129	GT228485	similar to Brain protein [Ricinus communis, EEF43789.1]	
	target 5' C A A 3'<br>CUCG UGCU UGCCUUGAGC<br>GAGU GUGA GCGGAGUUCG<br>miRNA 3' A C 5'	-26	165	GW613167	similar to s-adenosylmethionine synthetase [Ricinus communis, EEF50022.1]	catalytic activity
	target 5' A G C U 3'<br>CU GUCG UG CGUUUCAGGC<br>GA UAGU AC GCGGAGUUCG<br>miRNA 3' G G 5'	-25.3	491	GW613086	similar to GMP synthase [Ricinus communis, EEF29614.1]	binding
	target 5' U G G 3'<br>UUGUUGCUG GUUUCAGG<br>AGUAGUGAC CGGAGUUC	-25.5	260	GW612680	similar to LEC14B homolog [Vitis vinifera]	

	miRNA 3' G G G 5'					
	target 5' U C G A 3'<br>UUA CA UGUGUCUUGAGC<br>AGU GU ACGCGGAGUUCG<br>miRNA 3' G A G 5'	-26.3	515	FM890781	similar to pentatricopeptide repeat-containing protein [Ricinus communis, EEF35459.1]	binding
**EACMV-UG-mir-1-16***	target 5' U A A U 3'<br>GGAGCU GUUACUGUG CUUGA<br>CCUUGA UAGUGACGC GAGUU<br>miRNA 3' C G G C 5'	-29.2	563	GW612887	similar to ATP-dependent Clp protease proteolytic subunit [Ricinus communis, EEF34901.1]	binding
	target 5' U A A 3'<br>GAACUCAUUGC GC GCUUCAAG<br>CUUGAGUAGUG CG CGGAGUUC<br>miRNA 3' CC A 5'	-32.8	99	GW613879	similar to DNA binding protein [Ricinus communis, EEF33421.1]	binding
	target 5' C A C G A 3'<br>GG GGGCUUG UAU GUGUCUUGAG<br>CC CUUGAGU GUG CGCGGAGUUC<br>miRNA 3' A A 5'	-25.8	407	GW611465	similar to 40S ribosomal protein S3 [Ricinus communis, EEF46125.1]	structural molecule activity
	target 5' U A U C 3'<br>GGAGC CA CGUUG UGCUUCAGG<br>CCUUG GU GUGAC GCGGAGUUC<br>miRNA 3' C A A 5'	-26.7	308	FM895820	similar to aquaporin PIP1.3 [Ricinus communis, EEF51202.1]	transporter activity
**EACMV-UG-mir-1-17***	target 5' U U G G 3'<br>G GGAGCUU AUUACUGCGUUU<br>C CCUUGAG UAGUGACGCGGA<br>miRNA 3' GU C G 5'	-30.5	232	FM893702	similar to conserved hypothetical protein [Ricinus communis, EEF49979.1]	
**EACMV-UG-mir-1-18***	target 5' U U G G 3'<br>G GGAGCUU AUUACUGCGUUU<br>C CCUUGAG UAGUGACGCGGA<br>miRNA 3' U C 5	-30.1	143	GT228752	similar to conserved hypothetical protein [Ricinus communis, lEEF49979.1]	

**EACMV-UG-mir-1-19***	target 5' C A C G 3'<br>CUC UGUAG GGAACUCGUUAUU<br>GAG GUGUC CCUUGAGUAGUGA<br>miRNA 3' A C C 5'	-27	353	GW618384	similar to conserved hypothetical protein [Ricinus communis, EEF48602.1]	
**EACMV-UG-mir-2-1**	target 5' C G A 3'<br>AUGCUUGAG UAAAUGCUG<br>UAUGGACUC AUUUACGAC<br>miRNA 3' UA G 5'	-30.9	244	FM891923	similar to nucleic acid binding protein [Ricinus communis, EEF43170.1]	binding
	target 5' U G C U 3'<br>GUA ACCU GGCUAAAUGCU<br>UAU UGGA UCGAUUUACGA<br>miRNA 3' A C C 5'	-26.9	246	FM891901	similar to transferase, transferring glycosyl groups [Ricinus communis, EEF34254.1]	catalytic activity
	target 5' G U C 3'<br>AUAUCUGAGUUGA UGUUG<br>UAUGGACUCGAUU ACGAC<br>miRNA 3' UA U 5'	-26.7	158	FM888931	similar to cytochrome P450 [Ricinus communis, EEF48632.1]	electron carrier activity
	target 5' G U 3'<br>GUAUUUGAGUUAAGUGUU<br>UAUGGACUCGAUUUACGA<br>miRNA 3' UA C 5'	-25.8	19	GW612386	similar to 40S ribosomal protein S21e [Jatropha curcas, ADB93070.1]	structural molecule activity
	target 5' G U G 3'<br>GUGUGCUUGGGUUGGAU UUG<br>UAUAUGGACUCGAUUUA GAC<br>miRNA 3' C 5'	-25.4	210	FM895795	similar to PLE [Ricinus communis, EEF40549.1]	
	target 5' C G 3'<br>AUAUGCC GGGCU GGUGCUG<br>UAUAUGG CUCGA UUACGAC<br>miRNA 3' A U 5'	-28.4	175	GT975479	similar to enolase [Ricinus communis, EEF50680.1]	binding
**EACMV-UG-mir-2-2**	target 5' G U G 3'	-31.5	159	FM887573	similar to esterase precursor [Ricinus communis, EEF27799.1]	catalytic activity

	CUGAGCUAA UGUUGGUUU<br>GACUCGAUU ACGACCGGG<br>miRNA 3' UG U A 5'					
	target 5' A C C C A 3'<br>ACCUGA CU A AUGUUGGCCCU<br>UGGACU GA U UACGACCGGGA<br>miRNA 3' C U 5'	-33.7	269	GT977483	similar to lyase [Ricinus communis, EEF35024.1]	binding
	target 5' A U 3'<br>ACUUGGGUU AGUGCU GCCCU<br>UGGACUCGA UUACGA CGGGA<br>miRNA 3' U C 5'	-31.6	499	GT981938	similar to 21 kDa protein precursor [Ricinus communis, EEF32721.1]	enzyme regulator activity
	target 5' A U G 3'<br>GCUUGA UUA GUGUUGGCUUU<br>UGGACU GAU UACGACCGGGA<br>miRNA 3' C U 5'	-27.1	339	GT978828	similar to 40S ribosomal protein S9 [Ricinus communis, EEF45032.1]	structural molecule activity
	target 5' U G 3'<br>CCUGAGUUAGAUGCU GCU<br>GGACUCGAUUUACGA CGG<br>miRNA 3' U C GA 5'	-32.6	199	FM893635	similar to protein disulfide isomerase [Ricinus communis, EEF28509.1]	electron carrier activity
	target 5' G G C 3'<br>GCC GGGUU G UGCUGGCUCU<br>UGG CUCGA U ACGACCGGGA<br>miRNA 3' A U U 5'	-30	311	FM893511	similar to DNA-directed RNA polymerase II subunit [Ricinus communis, EEF43271.1]	binding
**EACMV-UG-mir-2-3***	target 5' A U G 3'<br>CUUGAUUUA AUGAAGCGU<br>GAGCUAAAU UGCUUCGUA<br>miRNA 3' G C AU 5'	-26.9	653	GT982597	similar to ADP,ATP carrier protein [Ricinus communis, EEF30484.1]	transporter activity
	target 5' U A C U C 3'<br>CCUCGG U AGG CGGAGCAUU<br>GGAGCU A UCU GCUUCGUAA<br>miRNA 3' A A U 5	-25.8	87	GW613444	similar to translation factor sui1 [Ricinus communis, EEF39555.1]	binding

**EACMV-UG-mir-2-4***	target 5' U U G 3'<br>UCUU AUUUGGACGAAGCA<br>GGAG UAAAUCUGCUUCGU<br>miRNA 3' UC C 5'	-28.6	353	GT975480	similar to signal transducer [Ricinus communis, EEF44718.1]	molecular trasducer activity
	target 5' U G G 3'<br>GGCCUUGAUU GGAUGAAG<br>UCGGAGCUAA UCUGCUUC<br>miRNA 3' A GU 5'	-30.7	92	GT973048	similar to glutamyl-tRNA synthetase 1, 2 [Ricinus communis, EEF29910.1]	binding
	target 5' C A A U 3'<br>AG CCUUGGU UAGGUGGAGC<br>UC GGAGCUA AUCUGCUUCG<br>miRNA 3' A U 5'	-25.8	249	GW612565	similar to alpha-1,3-mannosyltransferase [Ricinus communis, EEF36738.1]	catalytic activity
	target 5' G G U C 3'<br>AGUUUCGAUU GGGC AAGCG<br>UCGGAGCUAA UCUG UUCGU<br>miRNA 3' A C 5'	-26.9	267	GT981519	similar to heat shock factor protein [Ricinus communis, EEF51130.1]	nucleic acid binding transcription factor activity
**EACMV-UG-mir-2-5***	target 5' U G G 3'<br>GGCCUUGAUU GGAUGAAG<br>UCGGAGCUAA UCUGCUUC<br>miRNA 3' C A G 5'	-30.8	92	GT973048	similar to glutamyl-tRNA synthetase 1, 2 [Ricinus communis, EEF29910.1]	binding
	target 5' C A A U 3'<br>AG CCUUGGU UAGGUGGAGC<br>UC GGAGCUA AUCUGCUUCG<br>miRNA 3' C A 5'	-25.9	249	GW612565	similar to alpha-1,3-mannosyltransferase [Ricinus communis, EEF36738.1]	catalytic activity
	target 5' G G U G 3'<br>GAGUUUCGAUU GGGC AAGC<br>CUCGGAGCUAA UCUG UUCG	-28.7	266	GT981519	similar to heat shock factor protein [Ricinus communis, EEF51130.1]	nucleic acid binding transcription factor activity

	miRNA 3' A C 5'					
**EACMV-UG-mir-2-6***	target 5' A G U A 3'<br>GAGGAGCU UU GUUUGGAUGGA<br>CUUCUCGG AG UAAAUCUGCUU<br>miRNA 3' C 5'	-26.8	118	GW611852	similar to pectin acetylesterase [Ricinus communis, EEF42516.1]	catalytic activity
	target 5' G A A U 3'<br>GAAGAGCC U GUUUGGAUGG<br>CUUCUCGG G UAAAUCUGCU<br>miRNA 3' A C U	-27.6	6	GW612741	similar to 12-oxophytodienoate reductase opr [Ricinus communis, EEF35996.1]	catalytic activity
**EACMV-UG-mir-2-7***	target 5' A G G 3'<br>GAUUUGGG GAAGCGUUAA<br>CUAAAUCU CUUCGUAAUU<br>miRNA 3' G G 5'	-26.8	25	FM891122	similar to pyridoxin biosynthesis protein PDX1 [Ricinus communis, EEF46478.1]	catalytic activity
	target 5' A C 3'<br>GAUUUAGAUGAAGUGUUA<br>CUAAAUCUGCUUCGUAAU<br>miRNA 3' UG 5'	-26	230	GW619538	similar to shwachman-Bodian-Diamond syndrome protein [Ricinus communis, EEF42033.1]	
**EACMV-UG-mir-2-8***	target 5' G G A 3'<br>AGGCGAGGUA UUGAUGC<br>UCUGCUUCGU AAUUGCG<br>miRNA 3' A 5'	-26.6	599	GW613133	similar to hypothetical protein VITISV_036712 [Vitis vinifera]	

Table S5 Predicted putative targets of miRs/miRs* from ACMV and EACMV-UG in *Jatropha* ESTs using psRNATarget

miR/miR*	Expectation Score	Target start	Target end	Genbank accession	Target desccription	Target function
**ACMV-mir-1-1**	4.5	474	501	GW878812	similar to hydroxyacylglutathione hydrolase [Ricinus communis, EEF41604.1]	catalytic activity
	5	537	563	GT973023	similar to ATP-citrate lyase [Ricinus communis, EEF50019.1]	binding
	5	625	653	GW618705	similar to xyloglucan endotransglucosylase/hydrolase protein 2 precursor [Ricinus communis, EEF44209.1]	catalytic activity
**ACMV-mir-1-2**	4	148	167	GW877743	similar to beclin-1 [Ricinus communis, EEF39074.1]	
	4	244	263	GW879878	similar to xyloglucan endotransglucosylase / hydrolase protein A [Ricinus communis, EEF43915.1]	catalytic activity
	4.5	192	211	GT980870	similar to enolase [Ricinus communis, EEF51513.1]	binding
	4.5	173	192	GT972969	similar to lyase [Ricinus communis, EEF35024.1]	catalytic activity
	5	459	478	GW612383	similar to hydroxyacylglutathione hydrolase [Ricinus communis, EEF41604.1]	catalytic activity
**ACMV-mir-1-3**	3.5	47	66	GT974964	similar to protein phosphatase 2a, regulatory subunit [Ricinus communis, EEF37048.1]	enzyme regulator activity
	4	38	59	GT229219	similar to aspartic proteinase nepenthesin-1 precursor [Ricinus communis, EEF48609.1]	catalytic activity
	4	159	178	GW618769	similar to ctp synthase [Ricinus communis, EEF47967.1]	catalytic activity
	4.5	50	69	GW613454	similar to beclin-1 [Ricinus communis, EEF39074.1]	
	5	335	356	GW611248	similar to flavin-containing amine oxidase domain-containing protein [Ricinus communis, EEF28674.1]	binding
	5	282	303	FM891486	similar to pyruvate kinase [Ricinus communis, EEF45207.1]	binding
**ACMV-mir-1-4**	3.5	79	98	FM890127	similar to polyprotein [Arabidopsis thaliana, BAH30336.1]	binding
	4	28	47	GT978633	similar to ccr4-not transcription complex [Ricinus communis, EEF50313.1]	binding
	4	265	286	GW614613	similar to glutathione-s-transferase theta [Ricinus communis, EEF51173.1]	catalytic activity
	4	305	324	GT974021	similar to adenosine kinase [Ricinus communis, EEF30697.1]	catalytic activity
	4.5	335	356	GT979966	similar to protein transporter [Ricinus communis, EEF45900.1]	transporter activity
	5	651	672	GT981187	similar to 5-methyltetrahydropteroyltriglutamate-homocysteine methyltransferase [Ricinus communis, EEF46521.1]	catalytic activity
	5	133	154	GT980054	similar to big map kinase/bmk [Ricinus communis, EEF33894.1]	binding
	5	145	165	GT974552	similar to hydroxyacylglutathione hydrolase [Ricinus communis, EEF32884.1]	catalytic activity
**ACMV-mir-1-5**	4	385	404	GW614374	similar to ccr4-not transcription complex [Ricinus communis, EEF50313.1]	binding
	4	242	261	GT977179	similar to rubber particle protein [Ricinus communis, EEF30521.1]	
	4.5	670	691	GW877652	similar to sugar transporter [Ricinus communis, EEF49773.1]	transporter activity
	4.5	651	671	GT981187	similar to 5-methyltetrahydropteroyltriglutamate-homocysteine methyltransferase [Ricinus communis, EEF46521.1]	catalytic activity

	4.5	37	58	FM890843	similar to thioredoxin family protein [Medicago truncatula, AES64091.1]	electron carrier activity
	4.5	376	397	GW611332	similar to expressed protein [Ricinus communis, EEF45809.1]	binding
	5	518	540	GO246958	similar to profilin-like protein [Jatropha curcas, ACV50427.1]	
	5	131	153	GT980054	similar to big map kinase/bmk [Ricinus communis, EEF33894.1]	binding
**ACMV-mir-1-6**	4	29	48	FM890893	similar to catechol o-methyltransferase [Populus trichocarpa, EEE99217.1]	catalytic activity
	5	129	151	GT971194	similar to ATP synthase subunit a chloroplastic [Medicago truncatula, AES88228.1]	transporter activity
	5	614	636	GW612494	similar to GTP-binding protein (p) alpha subunit, gpa1 [Ricinus communis, EEF46154.1]	binding
	5	379	401	GW613522	similar to receptor protein kinase CLAVATA1 precursor [Ricinus communis, EEF52194.1]	binding
**ACMV-mir-1-7**	3.5	138	158	GW878128	similar to phosphatidylinositol-4-phosphate 5-kinase [Ricinus communis, EEF33121.1]	binding
	4	383	403	FM889095	similar to ATP binding protein [Ricinus communis, EEF48786.1]	binding
	4	151	170	GT972247	similar to heat shock protein binding protein [Arabidopsis lyrata subsp. Lyrata, EFH66709.1]	binding
	4.5	419	439	GW877743	similar to beclin-1 [Ricinus communis, EEF39074.1]	
	4.5	459	478	GW618812	similar to nucleoredoxin [Ricinus communis, EEF52780.1]	binding
	5	561	581	GW612274	similar to Alpha-N-arabinofuranosidase 1 precursor [Ricinus communis, EEF38707.1]	catalytic activity
	5	467	486	GW616837	similar to cinnamoyl-CoA reductase [Ricinus communis, EEF36026.1]	binding
**ACMV-mir-1-8**	2.5	22	41	GW880326	similar to DELLA protein GAI1 [Ricinus communis, EEF38923.1]	binding
	3.5	440	459	FM895812	similar to profilin-like protein [Jatropha curcas, ACV50427.1]	
	4	225	244	GW875314	similar to preprotein translocase secy subunit [Ricinus communis, EEF40118.1]	
	4	124	143	GW876194	similar to AP-2 complex subunit beta-1 [Ricinus communis, EEF45918.1]	transporter activity
	4	557	576	GW879599	similar to WD-repeat protein [Ricinus communis, EEF43070.1]	
	4.5	56	75	FM894823	similar to monothiol glutaredoxin-4 [Ricinus communis, EEF44744.1]	electron carrier activity
	5	538	557	GO246796	similar to trypsin-alpha amylase inhibitor [Jatropha curcas, ACM90158.1]	enzyme regulator activity
**ACMV-mir-1-9***	3.5	320	338	GW877361	similar to Phosphatidylcholine-sterol O-acyltransferase	catalytic activity
	4.5	566	586	GT972607	similar to signal recognition particle receptor subunit beta [Ricinus communis, EEF37035.1	binding
**ACMV-mir-1-10***	4.5	565	586	GT972607	similar to signal recognition particle receptor subunit beta [Ricinus communis, EEF37035.1	binding

**ACMV-mir-1-11***	4.5	565	586	GT972607	similar to signal recognition particle receptor subunit beta [Ricinus communis, EEF37035.1	binding
**ACMV-mir-1-12***	4.5	117	136	GT977148	similar to glyceraldehyde-3-phosphate dehydrogenase [Daucus carota, AEM24408.1]	binding
	4.5	565	585	GT972607	similar to signal recognition particle receptor subunit beta [Ricinus communis, EEF37035.1	binding
	5	325	346	GT976476	similar to myosin XI [Ricinus communis, EEF41316.1]	binding
**ACMV-mir-1-13***	4	115	134	GT977148	similar to glyceraldehyde 3-phosphate dehydrogenase [Ricinus communis, EEF51837.1]	binding
	5	167	190	GT977979	similar to ABC transporter-like protein [Glycine max, AAL66714.1]	binding
	5	309	332	FM893493	similar to vacuolar protein sorting-associated protein VPS9 [Ricinus communis, EEF48424.1]	binding
**ACMV-mir-1-14***	2	172	191	GT973147	similar to phosphoribosylamine-glycine ligase [Ricinus communis, EEF35937.1]	binding
	3.5	122	141	GW879285	similar to carboxypeptidase B2 precursor [Ricinus communis, EEF52851.1]	catalytic activity
	3.5	404	423	GW875007	similar to 4-hydroxy-3-methylbut-2-En-1-Yl diphosphate synthase [Ricinus communis, EEF32093.1]	binding
	3.5	216	235	GW612979	similar to type II inositol 5-phosphatase [Ricinus communis, EEF47324.1]	catalytic activity
	4	477	496	GT976492	similar to RNA-directed DNA polymerase (Reverse transcriptase) [Medicago truncatula, ABN08038.1]	binding
	4.5	103	122	GW877064	similar to actin depolymerizing factor [Ricinus communis, EEF37828.1]	
	4.5	324	343	FM895530	similar to aquaporin PIP1.3 [Ricinus communis, EEF51202.1]	transporter activity
	5	309	328	FM893493	similar to vacuolar protein sorting-associated protein VPS9 [Ricinus communis, EEF48424.1]	binding
**ACMV-mir-2-1**	2	49	68	GW880821	similar to myo inositol monophosphatase [Ricinus communis, EEF38236.1]	catalytic activity
	2.5	52	71	FM887240	similar to CYPRO4 [Ricinus communis, EEF43694.1]	
	2.5	30	49	GT972796	similar to epidermis-specific secreted glycoprotein EP1 precursor [Ricinus communis, EEF36763.1]	
	3	336	355	GW614445	similar to reticuline oxidase precursor [Ricinus communis, EEF32494.1]	catalytic activity
	3.5	27	46	GW618356	similar to vesicle transport V-snare protein vti1a [Ricinus communis, EEF39976.1]	
	4	378	397	GT976957	similar to CA2 (carbonic anhydrase) [Ricinus communis, EEF37015.1]	catalytic activity
	4	138	157	FM896552	similar to reticuline oxidase precursor [Ricinus communis, EEF32494.1]	catalytic activity
	4	462	481	FM890723	similar to pyruvate dehydrogenase [Ricinus communis, EEF42253.1]	catalytic activity

**ACMV-mir-2-2**	2.5	614	635	GW875806	similar to predicted protein [Populus trichocarpa, XP_002332240.1]	
	3	48	67	GW880821	similar to myo inositol monophosphatase [Ricinus communis, EEF38236.1]	catalytic activity
	3	377	396	GT976957	similar to CA2 (carbonic anhydrase) [Ricinus communis, EEF37015.1]	catalytic activity
	3	51	70	FM887240	similar to CYPRO4 [Ricinus communis, EEF43694.1]	
	3.5	29	48	GT972796	similar to epidermis-specific secreted glycoprotein EP1 precursor [Ricinus communis, EEF36763.1]	
	4	526	547	GW879086	similar to phosphoenolpyruvate carboxylase [Jatropha curcas, ABU41519.1]	catalytic activity
	4.5	363	384	GW875423	similar to alternative oxidase 4 [Ricinus communis, EEF43798.1] |	binding
	4.5	333	354	GW611582	similar to reticuline oxidase precursor [Ricinus communis, EEF32494.1]	catalytic activity
	4.5	519	540	GW615898	imilar to D-alanyl-D-alanine endopeptidase [Ricinus communis, EEF49094.1]	catalytic activity
**ACMV-mir-2-4**	3.5	469	490	GT975742	similar to kif4 [Ricinus communis, EEF45542.1]	binding
	4	477	501	FM889963	similar to yth domain-containing protein [Ricinus communis, EEF35947.1]	
	4.5	119	141	GW613467	similar to long-chain-fatty-acid CoA ligase [Ricinus communis, EEF48390.1]	catalytic activity
	4.5	168	189	GT980868	similar to tRNA modification GTPase [Ricinus communis, EEF48461.1]	binding
	5	336	359	GW876781	similar to glycerophosphoryl diester phosphodiesterase [Ricinus communis, EEF47756.1]	catalytic activity
	5	55	78	GW616510	imilar to shikimate kinase, chloroplast precursor [Ricinus communis, EEF30113.1]	binding
	5	564	587	GR716986	similar to protease inhibitor/seed storage/lipid transfer protein family protein [Jatropha curcas, ADU56178.1]	catalytic activity
**ACMV-mir-2-5**	3.5	469	489	GT975742	similar to kif4 [Ricinus communis, EEF45542.1]	binding
	4	168	188	GT980868	similar to tRNA modification GTPase [Ricinus communis, EEF48461.1]	binding
	4.5	336	358	GW876781	similar to glycerophosphoryl diester phosphodiesterase [Ricinus communis, EEF47756.1]	catalytic activity
	4.5	559	582	GT229152	similar to yth domain-containing protein [Ricinus communis, EEF35947.1]	
	5	55	77	GW616510	similar to shikimate kinase, chloroplast precursor [Ricinus communis, EEF30113.1]	binding
	5	495	514	GT970238	similar to cysteine protease inhibitor [Ricinus communis, EEF36811.1]	enzyme regulator activity
	5	119	140	GW613467	similar to long-chain-fatty-acid CoA ligase[Ricinus communis, EEF48390.1]	catalytic activity
**ACMV-mir-2-6**	4	56	79	FM893246	similar to 60S ribosomal protein L38 [Ricinus communis, EEF47361.1]	structural molecule activity
	4	489	512	GT970238	similar to cysteine protease inhibitor [Ricinus communis, EEF36811.1]	enzyme regulator activity

	5	509	532	GW879600	translation initiation factor IF-2 protein	binding
	5	52	75	GW616510	similar to shikimate kinase, chloroplast precursor [Ricinus communis, EEF30113.1]	binding
**ACMV-mir-2-7**	3.5	516	535	GW615203	similar to cytochrome P450 [Ricinus communis, EEF48632.1]	electron carrier activity
	4	389	412	FM894305	similar to cinnamoyl-CoA reductase [Ricinus communis, EEF44583.1]	binding
	4.5	124	146	FM892236	similar to NADPH:quinone oxidoreductase [Ricinus communis, EEF49550.1]	catalytic activity
	4.5	244	267	GW616972	similar to acyltransferase [Ricinus communis, EEF30758.1]	catalytic activity
	4.5	351	373	GW879881	similar to srpk [Ricinus communis, EEF34919.1]	binding
	4.5	356	379	GW617671	similar to non-symbiotic hemoglobin [Ricinus communis, EEF43318.1]	binding
	5	553	576	GW612164	similar to kinesin heavy chain [Ricinus communis, EEF52364.1]	binding
	5	544	566	FM894176	similar to GTP-binding protein-plant [Ricinus communis, EEF41261.1]	binding
	5	136	159	GW618350	similar to 40S ribosomal protein S1 [Ricinus communis, EEF50364.1]	structural molecule activity
**ACMV-mir-2-8***	3.5	560	581	GT969460	similar to DNA binding protein [Ricinus communis, EEF52479.1]	binding
	3.5	110	129	GW614884	similar to minor histocompatibility antigen H13 [Ricinus communis, EEF50539.1]	catalytic activity
	4	573	595	GW611952	similar to nucleolysin tia-1 [Ricinus communis, EEF33213.1]	binding
	4.5	379	401	GW619728	similar to conserved hypothetical protein [Ricinus communis, EEF29383.1]	
	4.5	90	112	GW875193	similar to neutral alpha-glucosidase ab precursor [Ricinus communis, EEF33007.1]	binding
	4.5	159	180	GW876854	similar to lysosomal pro-X carboxypeptidase [Ricinus communis, EEF31852.1]	catalytic activity
	5	575	596	GW878084	similar to rubisco subunit binding-protein beta subunit [Ricinus communis, EEF47654.1]	binding
**ACMV-mir-3-1**	3.5	116	135	GW878887	similar to protein KTI12 [Ricinus communis, EEF50764.1]	binding
	4	521	541	GW878793	similar to endoplasmic reticulum-Golgi intermediate compartment protein [Ricinus communis, EEF48479.1]	
	4	232	251	GW614590	similar to annexin [Ricinus communis, EEF48493.1]	binding
	4.5	342	362	FM896486	similar to transmembrane protein 14 [Ricinus communis, EEF46177.1]	
	4.5	338	358	FM893297	similar to malic enzyme [Ricinus communis, EEF48184.1]	binding
**ACMV-mir-3-2**	3	576	597	GW877851	similar to monothiol glutaredoxin-4 [Ricinus communis, EEF44744.1]	electron carrier activity
	3.5	248	267	GO246528	similar to ubiquitin-conjugating enzyme m [Ricinus communis, EEF37225.1]	binding
	3.5	376	397	GW614640	similar to ATP binding protein [Ricinus communis, EEF48054.1]	binding
	3.5	384	403	GT969533	similar to histone-lysine N-methyltransferase ASHR1-like [Vitis vinifera]	catalytic activity

	4	146	167	GW613233	similar to autoinhibited H+ ATPase [Populus trichocarpa, EEF09737.1]	binding
	4	109	129	GW879514	similar to transcription factor [Ricinus communis, EEF32013.1]	binding
**ACMV-mir-3-3**	2.5	613	632	GW878121	similar to DAG protein, chloroplast precursor [Ricinus communis, EEF29510.1]	
	3.5	411	432	GW614352	similar to ubiquitin-conjugating enzyme E2 G [Ricinus communis, EEF31013.1]	binding
	4	336	356	GW612177	similar to protein with unknown function [Ricinus communis, EEF28701.1]	
**ACMV-mir-3-4***	2.5	226	248	GW613001	similar to conserved hypothetical protein [Ricinus communis, EEF38004.1]	
	3	29	51	GW875900	similar to mitochondrial processing peptidase beta subunit [Ricinus communis, EEF36292.1]	binding
	3.5	51	73	GT977918	similar to ATP/ADP-transporter [Ricinus communis, EEF44719.1]	transporter activity
	3.5	58	80	GW613747	similar to ubiquitin-protein ligase [Ricinus communis, EEF51716.1]	catalytic activity
	4	66	86	GW879731	similar to oligopeptide transporter [Ricinus communis, EEF37920.1]	transporter activity
	4	438	460	GO247287	similar to annexin-like protein [Jatropha curcas, ACV50434.1]	binding
**ACMV-mir-3-5***	2	224	247	GW613001	similar to conserved hypothetical protein [Ricinus communis, EEF38004.1]	
	2.5	66	85	GW879731	similar to oligopeptide transporter [Ricinus communis, EEF37920.1]	transporter activity
	3	127	150	GW610943	similar to DNA binding protein [Ricinus communis, EEF33368.1]	binding
	3	52	75	GT970166	similar to casein kinase II beta chain [Ricinus communis, EEF35876.1]	catalytic activity
	3	27	50	GW875900	similar to mitochondrial processing peptidase beta subunit [Ricinus communis, EEF36292.1]	binding
	4	437	459	GO247285	similar to annexin-like protein [Jatropha curcas, ACV50434.1]	binding
	4.5	372	394	GW616393	similar to sarcosine oxidase [Ricinus communis, EEF27281.1]	catalytic activity
	5	52	75	GW880879	similar to WD-repeat protein [Ricinus communis, EEF28134.1]	
	5	526	549	GT980245	similar to gag-pol polyprotein [Medicago truncatula, ACL97386.1]	binding
**ACMV-mir-3-6***	1	223	246	GW613001	similar to conserved hypothetical protein [Ricinus communis, EEF38004.1]	
	2.5	245	268	GT973174	similar to conserved hypothetical protein [Ricinus communis, EEF31424.1]	
	3	66	88	GW881224	similar to casein kinase II beta chain [Ricinus communis, EEF35876.1]	catalytic activity
	3	51	74	GW620075	similar to ATP binding protein [Ricinus communis, EEF40223.1]	binding
	3	437	458	GO247285	similar to annexin-like protein [Jatropha curcas, ACV50434.1]	binding
	3.5	127	149	GW610943	similar to DNA binding protein [Ricinus communis, EEF33368.1]	binding
	3.5	115	138	GT975620	similar to nucleic acid binding protein [Ricinus communis, EEF40542.1]	binding

	4	525	548	GT980245	similar to gag-pol polyprotein [Medicago truncatula, ACL97386.1]	binding
**ACMV-mir-3-7***	1	223	245	GW613001	similar to conserved hypothetical protein [Ricinus communis, EEF38004.1]	
	2.5	125	148	GW610943	similar to DNA binding protein [Ricinus communis, EEF33368.1]	binding
	2.5	51	73	GW620075	similar to ATP binding protein [Ricinus communis, EEF40223.1]	binding
	2.5	52	73	GW880879	similar to WD-repeat protein [Ricinus communis, EEF28134.1]	
	4	524	547	GT980245	similar to gag-pol polyprotein [Medicago truncatula, ACL97386.1]	binding
	5	69	92	GW879286	similar to structural maintenance of chromosome protein [Ricinus communis, EEF30346.1]	binding
**ACMV-mir-3-8***	2.5	124	151	GW880532	DNA binding protein [Ricinus communis, XP_002528998.1]	binding
	2.5	258	279	GW875347	conserved hypothetical protein [Ricinus communis, XP_002525479.1]	
	4.5	55	82	GT976385	similar to protein phosphatase 2a, regulatory subunit [Ricinus communis, EEF37048.1]	enzyme regulator activity
	4.5	187	212	GT975672	similar to ATP synthase alpha subunit mitochondrial [Ricinus communis, EEF42989.1]	binding
**ACMV-mir-3-9***	3.5	188	211	GT975672	similar to ATP synthase alpha subunit mitochondrial [Ricinus communis, EEF42989.1]	binding
	4.5	45	68	FM896623	similar to 60S ribosomal protein L7ae [Ricinus communis, EEF36457.1]	structural molecule activity
	4.5	272	295	GW616006	similar to cyclophilin [Ricinus communis, EEF41372.1]	catalytic activity
	5	355	378	FM891174	similar to beta-mannosidase [Ricinus communis, EEF49833.1]	binding
**ACMV-mir-3-10***	2.5	223	243	GW613001	similar to conserved hypothetical protein [Ricinus communis, EEF38004.1]	
	2.5	258	278	GW875347	similar to conserved hypothetical protein [Ricinus communis, EEF36969.1]	
	3.5	121	144	GT974772	similar to ATP synthase alpha subunit mitochondrial [Ricinus communis, EEF42989.1]	binding
	4.5	88	111	GO247088	similar to 60S ribosomal protein L7a [Ricinus communis, EEF36457.1]	structural molecule activity
	4.5	272	295	GW616006	similar to cyclophilin [Ricinus communis, EEF41372.1]	catalytic activity
	5	355	378	FM891174	similar to beta-mannosidase [Ricinus communis, EEF49833.1]	binding
**ACMV-mir-3-11***	3	118	139	GT229245	similar to conserved hypothetical protein [Ricinus communis, EEF22889.1	
	3.5	105	127	GT973828	similar to hypothetical protein VITISV_029441 [Vitis vinifera]	
	4	187	210	GT975672	similar to ATP synthase alpha subunit mitochondrial [Ricinus communis, EEF42989.1]	binding
	4	68	89	GW879286	similar to structural maintenance of chromosome protein [Ricinus communis, EEF30346.1]	binding
	4	88	110	GO247088	similar to 60S ribosomal protein L7a [Ricinus communis, EEF36457.1]	structural molecule activity

	4	651	674	GW612153	similar to S-adenosylmethionine-dependent methyltransferase [Ricinus communis, EEF42470.1]	catalytic activity
	5	57	80	GT976385	similar to protein phosphatase 2a, regulatory subunit [Ricinus communis, EEF37048.1]	enzyme regulator activity
**ACMV-mir-3-12***	3	68	88	GW879286	similar to structural maintenance of chromosome protein [Ricinus communis, EEF30346.1]	binding
	4	56	79	GT976385	similar to protein phosphatase 2a, regulatory subunit [Ricinus communis, EEF37048.1]	enzyme regulator activity
	4	42	65	GT979674	similar to ATP synthase beta chain 2, mitochondrial [Ricinus communis, EEF30158.1]	binding
	4	650	673	GW612153	similar to S-adenosylmethionine-dependent methyltransferase [Ricinus communis, EEF42470.1]	catalytic activity
	4	120	142	GT974772	similar to ATP synthase alpha subunit mitochondrial [Ricinus communis, EEF42989.1]	binding
	5	61	84	GT979105	similar to aspartic proteinase precursor [Ricinus communis, EEF32480.1]	catalytic activity
**ACMV-mir-3-13***	2.5	118	137	GT229245	similar to conserved hypothetical protein [Ricinus communis, EEF22889.1]	
	3	41	64	GT979985	similar to ATP synthase beta chain 2, mitochondrial [Ricinus communis, EEF30158.1]	binding
	4	55	78	GT976385	similar to protein phosphatase 2a, regulatory subunit [Ricinus communis, EEF37048.1]	enzyme regulator activity
	4	60	83	GT979105	similar to aspartic proteinase precursor [Ricinus communis, EEF32480.1]	catalytic activity
	4	38	61	GW614166	similar to aspartic proteinase precursor [Ricinus communis, EEF32480.1]	catalytic activity
**ACMV-mir-3-14***	2.5	15	34	GT972003	similar to DELLA protein GAI [Ricinus communis, EEF49067.1]	binding
	2.5	36	55	GW615196	similar to flavonol 4'-sulfotransferase [Ricinus communis, EEF24263.1]	catalytic activity
	3	81	100	GW878538	similar to vacuolar ATP synthase subunit ac39 [Ricinus communis, EEF40830.1]	transporter activity
	3	355	374	FM891174	similar to beta-mannosidase [Ricinus communis, EEF49833.1]	binding
	3.5	57	77	GT976385	similar to protein phosphatase 2a, regulatory subunit [Ricinus communis, EEF37048.1]	enzyme regulator activity
	3.5	62	82	GT979105	similar to aspartic proteinase precursor [Ricinus communis, EEF32480.1]	catalytic activity
**ACMV-mir-3-15***	2.5	96	119	GW876074	similar to RING-H2 finger protein ATL51 [Arabidopsis thaliana, Q9SRQ8.2]	binding
	2.5	52	75	GT976385	similar to protein phosphatase 2a, regulatory subunit [Ricinus communis, EEF37048.1]	enzyme regulator activity
	3.5	69	92	GW878002	similar to serine-threonine protein kinase, plant-type [Ricinus communis, EEF43635.1]	binding
	3.5	61	84	GW874613	similar to leucine-rich repeat (LRR) family protein [Zea mays, ACG34018.1]	binding
	4	7	30	GW620161	similar to cullin-1 [Ricinus communis, EEF45513.1]	catalytic activity

	4	258	280	FM894039	similar to eukaryotic translation elongation factor [Ricinus communis, EEF48807.1]	binding
	4	52	75	GW880157	similar to pyridoxal-dependent decarboxylase	catalytic activity
	4	41	64	FM889919	similar to group II plp decarboxylase [Ricinus communis, EEF29371.1]	catalytic activity
	4.5	135	157	FM893864	similar to lactoylglutathione lyase [Ricinus communis, EEF45290]	catalytic activity
**ACMV-mir-3-16***	1.5	51	74	GT976385	similar to protein phosphatase 2a, regulatory subunit [Ricinus communis, EEF37048.1]	enzyme regulator activity
	1.5	114	137	GO247031	similar to DNA binding protein [Ricinus communis, EEF41393.1]	binding
	2	95	118	GW876074	similar to RING-H2 finger protein ATL51 [Arabidopsis thaliana, Q9SRQ8.2]	binding
	3	31	52	GW613144	similar to serine/threonine protein phosphatase [Ricinus communis, EEF49903.1]	binding
	3	133	154	GW615921	similar to lactoylglutathione lyase [Ricinus communis, EEF45290]	catalytic activity
	3.5	33	56	GW878318	similar to Transport Inhibitor Response 1 protein [Ricinus communis, EEF41643.1]	binding
	4	51	74	GW880157	similar to pyridoxal-dependent decarboxylase	catalytic activity
	4	40	63	FM889919	similar to group II plp decarboxylase [Ricinus communis, EEF29371.1]	catalytic activity
	5	48	71	GW877934	similar to nuclear transcription factor Y subunit A-1 [Ricinus communis, lEEF48902.1]	nucleic acid binding transcription factor activity
**ACMV-mir-3-17***	2	94	117	GW876074	similar to RING-H2 finger protein ATL51 [Arabidopsis thaliana, Q9SRQ8.2]	binding
	2	133	153	GW615921	similar to lactoylglutathione lyase [Ricinus communis, EEF45290]	catalytic activity
	2.5	31	51	GW613144	similar to serine/threonine protein phosphatase [Ricinus communis, EEF49903.1]	binding
	3	5	28	GW620161	similar to cullin-1[Ricinus communis, EEF45513.1]	catalytic activity
	3.5	33	55	GW878318	similar to transport Inhibitor Response 1 protein	binding
	4	49	72	GW880193	similar to group II plp decarboxylase [Ricinus communis, EEF29371.1]	catalytic activity
	4.5	47	70	GW877934	similar to nuclear transcription factor Y subunit A-1 [Ricinus communis, EEF48902.1]	nucleic acid binding transcription factor activity
**ACMV-mir-3-18***	1	93	115	GW876074	similar to RING-H2 finger protein ATL51 [Arabidopsis thaliana, Q9SRQ8.2]	binding
	2.5	4	26	GW620161	similar to cullin-1 [Ricinus communis, EEF45513.1]	catalytic activity
	3	208	230	GW874786	similar to cyclin dependent kinase [Ricinus communis, EEF42298.1]	catalytic activity
	3	47	68	GW877934	similar to nuclear transcription factor Y subunit A-1 [Ricinus communis, EEF48902.1]	nucleic acid binding transcription factor activity
	3	102	124	GT978628	similar to NADH dehydrogenase [Ricinus communis, EEF40884.1]	catalytic activity
	3.5	50	71	GW880157	similar to group II plp decarboxylase [Ricinus communis, EEF29371.1]	catalytic activity

	3.5	27	49	GW613144	similar to serine/threonine protein phosphatase [Ricinus communis, EEF49903.1]	binding
	4.5	121	143	GW612956	similar to peptidyl-tRNA hydrolase 2, mitochondrial isoform 1 [Vitis vinifera, CBI26515.3]	catalytic activity
**ACMV-mir-3-19***	1	93	115	GW876074	similar to RING-H2 finger protein ATL51 [Arabidopsis thaliana, Q9SRQ8.2]	binding
	2.5	4	26	GW620161	similar to cullin-1 [Ricinus communis, EEF45513.1]	catalytic activity
	3	208	230	GW874786	similar to cyclin dependent kinase [Ricinus communis, EEF42298.1]	catalytic activity
	3	47	68	GW877934	similar to nuclear transcription factor Y subunit A-1 [Ricinus communis, EEF48902.1]	nucleic acid binding transcription factor activity
	3	102	124	GT978628	similar to NADH dehydrogenase [Ricinus communis, EEF40884.1]	catalytic activity
	3.5	50	71	GW880157	similar to group II plp decarboxylase [Ricinus communis, EEF29371.1]	catalytic activity
	4	252	274	GW878471	similar to protein binding protein [Ricinus communis, EEF47573.1]	binding
	4.5	121	143	GW612956	similar to peptidyl-tRNA hydrolase 2, mitochondrial isoform 1 [Vitis vinifera, CBI26515.3]	catalytic activity
	5	274	297	GW881391	similar to cationic amino acid transporter [Populus trichocarpa, EEE88357.1]	transporter activity
**ACMV-mir-4-1**	3	118	138	GT975015	similar to WD-repeat protein [Ricinus communis, EEF30503.1]	
	4.5	461	484	GT981264	similar to ribosomal protein L2 (mitochondrion) [Phoenix dactylifera, AEM43912.1]	structural molecule activity
	4.5	453	475	GT975925	similar to serine/thronine protein kinase [Ricinus communis, EEF34412.1]	binding
	5	570	593	GW875401	similar to hypersensitive-induced response protein 1[Vitis vinifera]	
	5	592	615	GT228545	similar to protein PPLZ12 [Ricinus communis, EEF42191.1]	
	5	556	577	GW614403	similar to serine carboxypeptidase [Ricinus communis, EEF35107.1]	catalytic activity
**ACMV-mir-4-3**	4.5	496	519	GT974662	similar to chaperonin [Ricinus communis, EEF32749.1]	binding
	5	457	482	GT981264	similar to ribosomal protein L2 (mitochondrion) [Phoenix dactylifera, AEM43912.1]	structural molecule activity
**ACMV-mir-4-4**	4	170	190	GT979085	similar to 14 kDa proline-rich protein DC2.15 precursor, [Ricinus communis, EEF46942.1]	
	4.5	45	66	FM887259	similar to sucrose synthase [Ricinus communis, EEF45577.1]	catalytic activity
	4.5	90	112	GT980812	similar to tubby-like F-box protein 7-like [Glycine max, XP_003540491.1]	nucleic acid binding transcription factor activity
	5	478	500	FM896610	similar to 5-methyltetrahydropteroyltriglutamate-homocysteine methyltransferase [Ricinus communis, EEF36692.1]	catalytic activity
**ACMV-mir-4-5**	3.5	262	281	GW615281	similar to 60S ribosomal protein L44 [Ricinus communis, EEF46746.1]	structural molecule activity

	3.5	254	273	GW878805	similar to G-box-binding factor [Medicago truncatula, AET04216.1]	nucleic acid binding transcription factor activity
	4	44	63	FM887259	similar to sucrose synthase [Ricinus communis, EEF45577.1]	catalytic activity
	4	525	544	GT975233	similar to ATP binding protein [Ricinus communis, EEF29630.1]	binding
	4	67	86	GT971115	similar to aminotransferase class IV family protein [Zea mays, ACG38278.1]	catalytic activity
	4.5	13	32	GT974840	similar to elongation factor 1-alpha [Arabidopsis thaliana, AEE28214.1]	binding
	5	348	367	GT978930	similar to brassinosteroid insensitive 1-associated receptor kinase 1 precursor [Ricinus communis, EEF44972.1]	binding
	5	422	441	GW614133	similar to acyl-CoA:diacylglycerol acyltransferase 2 [Jatropha curcas, AEZ56254.1]	catalytic activity
**ACMV-mir-4-6***	2.5	389	409	GT980294	similar to trytophan synthase alpha subunit [Ricinus communis, EEF41392.1]	catalytic activity
	4	261	281	GW877652	similar to sugar transporter [Ricinus communis, EEF49773.1]	transporter activity
	4	287	307	GW617980	similar to cell elongation protein diminuto [Ricinus communis, EEF46584.1]	catalytic activity
	5	304	327	GW879408	similar to ribosome biogenesis protein nop10 [Ricinus communis, EEF32551.1]	
	5	542	564	GW618533	similar to alcohol dehydrogenase [Ricinus communis, EEF37925.1]	binding
**ACMV-mir-4-7***	3	387	406	GT980294	similar to trytophan synthase alpha subunit [Ricinus communis, EEF41392.1]	catalytic activity
	3.5	304	323	GW879408	similar to ribosome biogenesis protein nop10 [Ricinus communis, EEF32551.1]	
	3.5	318	337	FM894994	similar to 14-3-3 protein [Ricinus communis, EEF48539.1]	binding
	4	295	314	GW876278	similar to trytophan synthase alpha subunit [Ricinus communis, EEF41392.1]	catalytic activity
	4	6	25	FM895179	similar to serine/threonine kinase [Manihot esculenta, ABK58142.1]	binding
	4	265	284	GW611892	similar to UDP-glucuronosyltransferase [Ricinus communis, EEF47788.1]	catalytic activity
	4	329	348	GT974095	similar rotenone-insensitive NADH-ubiquinone oxidoreductase, mitochondrial precursor [Ricinus communis, EEF36776.1]	catalytic activity
	4.5	553	572	GW618709	similar to glutathione-s-transferase theta [Jatropha curcas, ADB93065.1]	catalytic activity
	5	417	436	GW878919	similar to 1-aminocyclopropane-1-carboxylate synthase [Populus trichocarpa, EEE81653.1]	catalytic activity
**ACMV-mir-5-1**	3	617	637	GW878601	similar to AT-rich interactive domain-containing protein 2 [Vitis vinifera, XP_002283908.1]	binding
	3.5	67	86	FM888834	similar to translationally controlled tumor protein [Jatropha curcas, ABO25950.1]	binding
	4	475	497	GO246779	similar to o-methyltransferase [Ricinus communis, EEF38813.1]	catalytic activity
**ACMV-mir-5-2**	3	617	637	GW878601	similar to AT-rich interactive domain-containing protein 2 [Vitis vinifera, XP_002283908.1]	binding

	3.5	67	86	FM888834	similar to translationally controlled tumor protein [Jatropha curcas, ABO25950.1]	binding
	4	627	651	GW611228	similar to dtdp-glucose 4-6-dehydratase [Ricinus communis, EEF33919.1]	catalytic activity
	4	475	497	GO246779	similar to o-methyltransferase [Ricinus communis, EEF38813.1]	catalytic activity
	4.5	502	525	GW613466	similar to phospholipase D [Jatropha curcas, ADA72022.1]	binding
	5	46	70	GT979291	similar to DNA-binding protein MNB1B [Ricinus communis, EEF34283.1]	binding
**ACMV-mir-5-3**	3	617	637	GW878601	similar to AT-rich interactive domain-containing protein 2 [Vitis vinifera, XP_002283908.1]	binding
	3	626	650	GW611228	similar to dtdp-glucose 4-6-dehydratase [Ricinus communis, EEF33919.1]	catalytic activity
	4	66	85	FM888834	similar to translationally controlled tumor protein [Jatropha curcas, ABO25950.1]	binding
	4.5	308	329	GW880768	similar to Palmitoyltransferase ZDHHC9 [Ricinus communis, EEF51726.1]	catalytic activity
	4.5	366	389	GW614802	similar to serine/threonine protein phosphatase 2a regulatory subunit A [Ricinus communis, EEF33251.1]	
**ACMV-mir-5-4**	3.5	308	328	GW880768	similar to Palmitoyltransferase ZDHHC9 [Ricinus communis, EEF51726.1]	catalytic activity
	3.5	366	388	GW614802	similar to serine/threonine protein phosphatase 2a regulatory subunit A [Ricinus communis, EEF33251.1]	
	4	626	649	GW611228	similar to dtdp-glucose 4-6-dehydratase [Ricinus communis, EEF33919.1]	catalytic activity
	4	562	585	GW615568	similar to Beta-glucosidase [Ricinus communis, EEF42691.1]	binding
	4.5	61	84	FM892758	similar to stromal membrane-associated protein [Ricinus communis, EEF31337.1]	enzyme regulator activity
	5	104	127	GW616955	similar to importin alpha [Ricinus communis, EEF49980.1]	transporter activity
	5	257	279	GW611675	similar to copine [Ricinus communis, EEF49756.1]	binding
**ACMV-mir-5-5**	2.5	499	520	GW613466	similar to phospholipase D [Ricinus communis, AAB37305.1]	binding
	3.5	503	522	GW612536	similar to U1 small nuclear ribonucleoprotein C [Ricinus communis, EEF41660.1]	binding
	4	22	43	GW614054	similar to DNA binding protein [Ricinus communis, EEF33593.1]	binding
	4	529	550	GT971019	similar to 50S ribosomal protein L15 [Ricinus communis, EEF30749.1]	structural molecule activity
	4	561	582	GW616484	similar to Beta-glucosidase [Ricinus communis, EEF42691.1]	binding
**ACMV-mir-5-6**	2.5	498	520	GW613466	similar to phospholipase D [Ricinus communis, AAB37305.1]	binding
	4	560	582	GW616484	similar to Beta-glucosidase [Ricinus communis, EEF42691.1]	binding
	4	6	27	GW615413	similar to DNA binding protein [Ricinus communis, EEF33593.1]	binding
	4	529	550	GT971019	similar to 50S ribosomal protein L15 [Ricinus communis, EEF30749.1]	structural molecule activity
**ACMV-mir-5-8***	3.5	403	423	GT979455	similar to auxin-repressed protein-like protein ARP1 [Jatropha curcas, ADB02903.1]	

	5	228	249	GW880777	similar to AFG1-like [Arabidopsis thaliana, AAU95415.1]	binding
**ACMV-mir-5-9***	2	228	247	GW880777	similar to AFG1-like [Arabidopsis thaliana, AAU95415.1]	binding
	4	366	388	GW611682	similar to inorganic pyrophosphatase [Ricinus communis, EEF47080.1]	catalytic activity
	4.5	236	257	GT979071	similar to prefoldin [Ricinus communis, EEF41462.1]	binding
	5	141	163	GT978329	similar to inosine triphosphate pyrophosphatase [Ricinus communis, EEF44280.1]	catalytic activity
	5	39	61	GW880240	similar to vacuole membrane protein [Ricinus communis, EEF45384.1]	
	5	467	489	GW875930	similar to Poly(rC)-binding protein [Ricinus communis, EEF52201.1]	binding
	5	186	207	GW881443	similar to calcium-dependent protein kinase [Ricinus communis, EEF40200.1]	binding
**ACMV-mir-5-10***	2	227	246	GW880777	similar to AFG1-like [Arabidopsis thaliana, AAU95415.1]	binding
	3.5	141	162	GT978329	similar to inosine triphosphate pyrophosphatase [Ricinus communis, EEF44280.1]	catalytic activity
	4	365	387	GW611682	similar to inorganic pyrophosphatase [Ricinus communis, EEF47080.1]	catalytic activity
	4	467	488	GW875930	similar to Poly(rC)-binding protein [Ricinus communis, EEF52201.1]	binding
	4.5	574	596	GW881211	similar to protein kinase 1b [Solanum lycopersicum, ACD77110.1]	binding
	4.5	153	174	FM893428	similar to 40S ribosomal protein S5B [Hevea brasiliensis, ADR71287.1]	structural molecule actvity
	5	124	145	GT975799	similar to methionine aminopeptidase[Ricinus communis, EEF38754.1]	catalytic activity
	5	332	352	GT228587	similar to pectinesterase inhibitor [Ricinus communis, EEF49909.1]	enzyme regulator activity
**ACMV-mir-5-11***	3	100	120	GT976156	similar to ubiquitin ligase SINAT2 [Ricinus communis, EEF28013.1]	catalytic activity
	3	136	158	GT978329	similar to inosine triphosphate pyrophosphatase [Ricinus communis, EEF44280.1]	catalytic activity
	3	166	186	GW877380	similar to guanine nucleotide-binding protein beta [Ricinus communis, EEF50865.1]	catalytic activity
	3.5	103	124	GT971284	similar to porphobilinogen deaminase [Ricinus communis, EEF42025.1]	catalytic activity
	4	77	99	GW875325	similar to muconate cycloisomerase [Ricinus communis, EEF37519.1]	catalytic activity
	4	64	86	GT971328	similar to amino acid binding protein [Ricinus communis, EEF42045.1]	binding
**ACMV-mir-5-12***	2	100	119	GT976156	similar to ubiquitin ligase SINAT2 [Ricinus communis, EEF28013.1]	catalytic activity
	2.5	162	185	GW877380	similar to guanine nucleotide-binding protein beta [Ricinus communis, EEF50865.1]	catalytic activity

	3	76	98	GW875325	similar to muconate cycloisomerase [Ricinus communis, EEF37519.1]	catalytic activity
	4.5	105	128	GT976828	similar to cysteine protease inhibitor [Ricinus communis, EEF36811.1]	enzyme regulator activity
	5	134	157	GO247609	similar to ethylene-responsive transcription factor [Ricinus communis, EEF44389.1]	nucleic acid binding transcription factor activity
	5	140	163	GW876523	similar to pentatricopeptide repeat-containing protein [Ricinus communis, EEF38551.1]	binding
	5	65	88	GW875268	similar to zinc finger family protein [Arabidopsis lyrata subsp. Lyrata, EFH70377.1]	binding
	5	84	107	GW877801	similar to flowering time control protein [Ricinus communis]	binding
**ACMV-mir-5-13***	3	74	97	GW875325	similar to muconate cycloisomerase [Ricinus communis, EEF37519.1]	catalytic activity
	3.5	161	184	GW877380	similar to guanine nucleotide-binding protein beta [Ricinus communis, EEF50865.1]	catalytic activity
	4	104	127	GT976828	similar to cysteine protease inhibitor [Ricinus communis, EEF36811.1]	enzyme regulator activity
	4	59	82	GO247642	similar to ubiquinol-cytochrome C reductase [Jatropha curcas, ADB02893.1]	enzyme regulator activity
	5	477	500	GO247112	similar to ethylene-responsive transcription factor [Jatropha curcas, AEJ87198.1]	nucleic acid binding transcription factor activity
**ACMV-mir-5-14***	3	104	126	GT976828	similar to cysteine protease inhibitor [Ricinus communis, EEF36811.1]	enzyme regulator activity
	3.5	136	158	GW876402	similar to guanine nucleotide-binding protein beta [Ricinus communis, EEF50865.1]	catalytic activity
	4	477	499	GO247112	similar to ethylene-responsive transcription factor [Jatropha curcas, AEJ87198.1]	nucleic acid binding transcription factor activity
**ACMV-mir-5-15***	3	103	126	GT976828	similar to cysteine protease inhibitor [Ricinus communis, EEF36811.1]	enzyme regulator activity
	4	476	499	GO247112	similar to ethylene-responsive transcription factor [Jatropha curcas, AEJ87198.1]	nucleic acid binding transcription factor activity
**ACMV-mir-6-1**	2	89	108	GT972735	similar to DNA binding protein [Ricinus communis, EEF38786.1]	binding
	2.5	116	135	FM890468	similar to triose phosphate/phosphate translocator, non-green plastid, chloroplast precursor [Ricinus communis, EEF51988.1]	transporter activity
	2.5	157	176	GT972267	similar to WD-repeat protein [Ricinus communis, EEF30258.1]	
	4	56	76	GT974438	similar to ubiquitin carboxyl-terminal hydrolase [Ricinus communis, EEF32116.1]	catalytic activity
	4.5	475	494	GW618981	similar to nutrient reservoir [Ricinus communis, EEF31271.1]	nutrient reservoir activity
	4.5	327	347	GW614359	similar to WD-repeat protein [Ricinus communis, EEF41096.1]	
	4.5	278	297	GT978886	similar to aquaporin PIP1.3 [Ricinus communis, EEF51202.1]	transporter activity
	4.5	190	210	FM895181	similar to mitogen activated protein kinase kinase, mapkk2 [Ricinus communis, EEF48158.1]	binding
	5	250	270	GW615830	similar to multiple inositol polyphosphate phosphatase 1 precursor [Ricinus communis, EEF41487.1]	catalytic activity

	5	450	470	GT978333	similar to short-chain dehydrogenase [Ricinus communis, EEF31844]	binding
**ACMV-mir-6-2**	2.5	115	134	FM890468	similar to triose phosphate/phosphate translocator, non-green plastid, chloroplast precursor [Ricinus communis, EEF51988.1]	transporter activity
	3	86	107	GT972735	similar to DNA binding protein [Ricinus communis, EEF38786.1]	binding
	3.5	242	266	GW611674	similar to heterogeneous nuclear ribonucleoprotein 27C [Ricinus communis, EEF34155.1]	binding
	3.5	199	218	GT973762	similar to multiple inositol polyphosphate phosphatase 1 precursor [Ricinus communis, EEF41487.1]	catalytic activity
	4	134	155	GW617175	similar to protein phosphatase 2c [Ricinus communis, EEF43151]	binding
	4.5	470	493	GW618981	similar to nutrient reservoir [Ricinus communis, EEF31271.1]	nutrient reservoir activity
	4.5	276	298	GW618640	similar to 30S ribosomal protein S14 [Ricinus communis, EEF29890.1]	structural molecule activity
	4.5	582	604	GT977004	similar to ubiquitin-protein ligase [Ricinus communis, EEF36838.1]	catalytic activity
	5	232	255	GW614732	similar to rer1 protein [Ricinus communis, EEF52597.1]	
	5	324	346	GW614359	similar to WD-repeat protein [Ricinus communis, EEF41096.1]	
	5	101	123	GW880575	similar to RNA binding protein [Ricinus communis, EEF34725.1]	binding
**ACMV-mir-6-3**	2.5	115	134	FM890468	similar to triose phosphate/phosphate translocator, non-green plastid, chloroplast precursor [Ricinus communis, EEF51988.1]	transporter activity
	3	86	107	GT972735	similar to DNA binding protein [Ricinus communis, EEF38786.1]	binding
	3.5	242	266	GW611674	similar to heterogeneous nuclear ribonucleoprotein 27C [Ricinus communis, EEF34155.1]	binding
	3.5	199	218	GT973762	similar to multiple inositol polyphosphate phosphatase 1 precursor [Ricinus communis, EEF41487.1]	catalytic activity
	4	134	155	GW617175	similar to protein phosphatase 2c [Ricinus communis, EEF43151]	binding
	4.5	275	298	GW618410	similar to nutrient reservoir [Ricinus communis, EEF31271.1]	nutrient reservoir activity
	4.5	276	298	GW618640	similar to 30S ribosomal protein S14 [Ricinus communis, EEF29890.1]	structural molecule activity
	4.5	582	604	GT977004	similar to ubiquitin-protein ligase [Ricinus communis, EEF36838.1]	catalytic activity
	5	232	255	GW614732	similar to rer1 protein [Ricinus communis, EEF52597.1]	
	5	324	346	GW614359	similar to WD-repeat protein [Ricinus communis, EEF41096.1]	
	5	101	123	GW880575	similar to RNA binding protein [Ricinus communis, EEF34725.1]	binding
**ACMV-mir-6-4**	3	640	659	GW615426	similar to conserved hypothetical protein [Ricinus communis, EEF36862.1]	
	3.5	462	481	GT976955	similar to protein kinase family protein	catalytic activity
	4	85	105	GT978410	similar to GTP binding / GTPase	binding
	4.5	10	30	GW876253	similar to truncated hemoglobin	binding
	4.5	486	505	GT979493	similar to cytochrome B [Ricinus communis, XP_002535342.1]	electron carrier activity
	4.5	13	34	GW874915	similar to ubiquitin specific protease3	catalytic activity
	4.5	258	277	FM894440	similar to peptidase M1 family protein [Arabidopsis thaliana, AEE34145.1]	catalytic activity
	4.5	97	119	GT979312	similar to heavy metal cation transport atpase [Ricinus communis, EEF37424.1]	binding

	5	328	350	GT970219	similar to rer1 protein [Ricinus communis, EEF37411.1]	
**ACMV-mir-6-5**	2.5	175	198	FM890649	similar to glucan endo-1,3-beta-glucosidase precursor [Ricinus communis, EEF52560.1]	binding
	2.5	244	266	FM892996	similar to 60S ribosomal protein L18 [Ricinus communis, EEF39291.1]	structural molecule activity
	4	232	251	GT971843	similar to serine/threonine protein phosphatase 7 long form homolog	binding
	4	466	487	GT979240	similar to 28S ribosomal protein S29, mitochondrial [Vitis vinifera, CBI15861.3]	structural molecule activity
	4	151	173	FM889134	similar to pyruvate kinase [Ricinus communis, EEF33220.1]	binding
	4.5	180	201	GW614575	similar to auxin-responsive protein IAA1 [Ricinus communis, EEF44693.1]	nucleic acid binding transcription factor activity
	4.5	501	522	GW615091	similar to protein with unknown function [Ricinus communis, EEF40990.1]	
	5	206	228	GW875881	similar to 4-alpha-glucanotransferase [Ricinus communis, EEF38704.1]	catalytic activity
**ACMV-mir-6-6**	4	352	373	GT971958	similar to pectinesterase family protein [Ricinus communis, EEF51542.1]	enzyme regulator activity
	4.5	445	468	GW610934	similar to annexin-like protein [Jatropha curcas, ACV50434.1]	binding
**ACMV-mir-6-7***	4	226	245	GW878229	similar to aldose 1-epimerase [Ricinus communis, EEF50625.1]	binding
	4	130	149	GT979168	similar to pentatricopeptide repeat-containing protein [Ricinus communis, EEF47332.1]	binding
	4	317	335	GW616902	similar to tubulin beta chain [Ricinus communis, EEF39168.1]	structural molecule activity
	4.5	14	33	GW616012	similar to E3 ubiquitin-protein ligase MARCH6-like [Glycine max, XP_003518705.1]	catalytic activity
	4.5	224	243	GW876142	similar to pumilio protein [Ricinus communis, EEF48717.1]	binding
	4.5	303	322	GW880504	similar to shikimate kinase, chloroplast precursor [Ricinus communis, EEF30113.1]	binding
	4.5	427	446	GW881184	similar to arginine/serine-rich splicing factor [Ricinus communis, EEF39264.1]	binding
**ACMV-mir-6-8***	3.5	258	277	GW881107	similar to cinnamoyl-CoA reductase [Ricinus communis, EEF36026.1]	binding
	4	313	333	GW616902	similar to tubulin beta chain [Ricinus communis, EEF39168.1]	structural molecule activity
	4	126	146	GT979168	similar to pentatricopeptide repeat-containing protein [Ricinus communis, EEF47332.1]	binding
	4	423	443	GW881184	similar to arginine/serine-rich splicing factor [Ricinus communis, EEF39264.1]	binding
	5	607	630	GT971709	similar to transcription factor [Ricinus communis, EEF27845.1]	binding
	5	493	516	GT976215	similar to NADH-ubiquinone oxidoreductase I subunit j [Ricinus communis, EEF38455.1]	binding
**ACMV-mir-6-9***	2.5	305	327	GW616902	similar to tubulin beta chain [Ricinus communis, EEF39168.1]	structural molecule activity
	3.5	510	529	GT974037	similar to adenosine kinase [Ricinus communis, EEF30697.1]	catalytic activity
	4	642	661	GT972764	similar to zinc finger protein [Ricinus communis, EEF52759.1]	binding

	5	82	104	GW879191	similar to nicastrin precursor [Ricinus communis, EEF30446.1]	catalytic activity
**ACMV-mir- 6-10***	4	369	389	FM888971	similar to NADH-ubiquinone oxidoreductase 39 kD subunit, [Ricinus communis, EEF35812.1]	binding
	5	483	505	GW618952	similar to exopolygalacturonase clone GBGE184 precursor [Ricinus communis, EEF45581.1]	catalytic activity
	5	210	232	GW619957	similar to glucose-6-phosphate 1-dehydrogenase [Ricinus communis, EEF32168.1]	binding
	5	185	207	GW612101	similar to fad NAD binding oxidoreductases [Ricinus communis, EEF50830.1]	catalytic activity
**ACMV-mir-6-11***	4	232	253	GW875047	similar to cyclin B [Ricinus communis, EEF52862.1]	
	4	458	478	GT981318	similar to hydrolase [Ricinus communis, EEF28272.1]	catalytic activity
	4	369	388	FM888971	similar to NADH-ubiquinone oxidoreductase 39 kD subunit [Ricinus communis, EEF35812.1]	binding
	5	480	502	GW881211	similar to protein kinase 1b [Solanum lycopersicum, ACD77110.1]	binding
**ACMV-mir-6-13***	3.5	145	167	GT976985	similar to tRNA (cytosine-5-)-methyltransferase NSUN2 [Medicago truncatula, AES72179.1]	catalytic activity
	3.5	306	325	GT973352	similar to dihydropteroate synthase [Ricinus communis, EEF41573.1]	catalytic activity
	4	78	97	GW614193	similar to cucumber peeling cupredoxin [Ricinus communis, EEF35204.1]	binding
	4	231	250	GW875047	similar to cyclin B [Ricinus communis, EEF52862.1]	
	4	154	175	GO246643	similar to gibberellin-regulated protein 1 precursor [Ricinus communis, EEF52055.1]	
	4	477	499	GW881211	similar to protein kinase 1b [Solanum lycopersicum, ACD77110.1]	binding
	4	114	133	GT975582	similar to dihydropteroate synthase [Ricinus communis, EEF41573.1]	catalytic activity
	4.5	70	89	GT970708	similar to 2,3-biphosphoglycerate-independent phosphoglycerate mutase [Ricinus communis, EEF42299.1]	binding
	4.5	248	269	GW619610	similar to protein phosphatase 2c [Ricinus communis, EEF48180]	binding
	4.5	655	674	GW877570	similar to transcription factor [Ricinus communis, EEF33838.1]	binding
	5	487	507	GT981698	similar to kiwellin [Ricinus communis, EEF32953.1]	
**ACMV-mir-6-14***	3.5	541	560	GT976507	similar to dihydropteroate synthase [Ricinus communis, EEF41573.1]	catalytic activity
	4.5	353	374	GW877369	similar to uncharacterized protein LOC100265434 [Vitis vinifera]	
	4.5	136	155	GT974081	similar to dihydropteroate synthase [Ricinus communis, EEF41573.1]	catalytic activity
	5	68	87	GT970708	similar to 2,3-biphosphoglycerate-independent phosphoglycerate mutase [Ricinus communis, EEF42299.1]	binding
**ACMV-mir-6-15***	3.5	576	596	GW617139	similar to isoflavone reductase related protein [Pyrus communis]	binding
	4.5	332	355	GW615118	similar to flavonoid 3'-hydroxylase [Populus trichocarpa, EEE95684.1]	electron carrier activity

**ACMV-mir-6-16***	3	528	549	GT981524	similar to cytochrome P450 [Ricinus communis, EEF33750.1]	electron carrier activity
	3.5	119	139	FM894086	similar to eukaryotic initiation factor iso-4F subunit p82-34 [Ricinus communis, EEF28321.1]	binding
	4	465	487	GT981069	similar to heterogeneous nuclear ribonucleoprotein [Ricinus communis, EEF48904.1]	binding
	4	465	487	GT972684	similar to heterogeneous nuclear ribonucleoprotein [Ricinus communis, EEF48904.1]	binding
	4.5	293	316	GW615112	similar to polygalacturonase non-catalytic subunit AroGP2 precursor [Ricinus communis, EEF39174.1]	
	5	459	482	FM888386	similar to 4-hydroxybenzoate octaprenyltransferase [Ricinus communis, EEF40466.1]	catalytic activity
**ACMV-mir-6-17***	2.5	119	138	FM894086	similar to eukaryotic initiation factor iso-4F subunit p82-34 [Ricinus communis, EEF28321.1]	binding
	3	465	486	GT981069	similar to ATP-dependent Clp protease proteolytic subunit, [Ricinus communis, EEF43288.1]	binding
	3	465	486	GT972684	similar to heterogeneous nuclear ribonucleoprotein [Ricinus communis, EEF48904.1]	binding
	3.5	528	548	GT981524	similar to cytochrome P450 [Ricinus communis, EEF33750.1]	electron carrier activity
	4	461	481	FM888386	similar to 4-hydroxybenzoate octaprenyltransferase [Ricinus communis, EEF40466.1]	catalytic activity
	4	233	252	GT981153	similar to pyruvate dehydrogenase [Ricinus communis, EEF42253.1]	catalytic activity
	4.5	633	653	GT977443	similar to protein phosphatase 2C [Ricinus communis, EEF44394.1]	binding
**ACMV-mir-7-1**	3.5	186	205	GW880834	similar to aldehyde dehydrogenase [Ricinus communis, EEF43083.1]	catalytic activity
	3.5	297	316	GW618732	similar to lupus la ribonucleoprotein [Ricinus communis, EEF40478.1]	binding
	4	281	301	GT971733	similar to aminoadipic semialdehyde synthase [Ricinus communis, EEF46187.1]	binding
	4	466	487	GT977783	similar to ADP-ribosylation factor [Ricinus communis, EEF29965.1]	binding
	4.5	309	328	FM895096	similar to ATP binding protein [Ricinus communis, EEF31681.1]	binding
	5	317	339	FM893119	similar to ribosomal protein L39e [Ricinus communis, EEF38862.1]	structural molecule activity
**ACMV-mir-7-2***	3.5	552	573	GT977103	similar to WD-repeat protein [Ricinus communis, EEF33655.1]	
	3.5	324	346	GW613305	similar to fyve finger-containing phosphoinositide kinase, fyv1[Ricinus communis, EEF36348.1]	catalytic activity
	4.5	457	479	GT972310	similar to Beta-1,3-galactosyltransferase sqv-2 [Ricinus communis, EEF43468.1]	catalytic activity
	5	85	106	GO246773	similar to ubiquitin-conjugating enzyme E2 [Ricinus communis, EEF51304.1]	catalytic activity
	5	106	127	FM889497	similar to calcium ion binding protein [Ricinus communis, EEF38369.1]	binding

**ACMV-mir-7-3***	3.5	457	477	GT972310	similar to Beta-1,3-galactosyltransferase sqv-2 [Ricinus communis, EEF43468.1]	catalytic activity
	3.5	552	571	GT977103	similar to WD-repeat protein [Ricinus communis, EEF33655.1]	
	4	328	348	GW880609	similar to zinc finger protein [Ricinus communis, EEF42871.1]	binding
	4	52	71	GT229130	similar to tubulin-specific chaperone A [Ricinus communis, EEF38347.1]	binding
	4	85	104	GO246773	similar to ubiquitin-conjugating enzyme E2 [Ricinus communis, EEF51304.1]	binding
	4.5	229	249	GW614642	similar to zinc finger protein [Ricinus communis, EEF42871.1]	binding
	5	126	146	GT982234	similar to lipoxygenase [Ricinus communis, EEF49219.1]	binding
**EACMV-UG-mir-1-1**	3	3	24	GT970645	similar to vesicle-associated membrane protein [Ricinus communis, EEF33458.1]	structural molecule activity
	4	87	110	GT969463	similar to JHL23C09.2 [Jatropha curcas, AJ53210.1]	
	5	197	220	GW881131	similar to Serine/threonine protein kinase [Ricinus communis, EEF39636.1]	binding
	5	645	668	GT969497	similar to 14.3 kDa OLEO1 [Jatropha curcas]	
**EACMV-UG-mir-1-2**	2.5	195	218	GW881131	similar to serine/threonine protein kinase [Ricinus communis, EEF39636.1]	binding
	2.5	3	22	GT970645	similar to vesicle-associated membrane protein [Ricinus communis, EEF33458.1]	structural molecule activity
	3.5	443	463	GT981493	similar to 60S acidic ribosomal protein P2 [Ricinus communis, EEF37217.1]	structural molecule activity
	4	86	108	GT969463	similar to JHL23C09.2 [Jatropha curcas, AJ53210.1]	
**EACMV-UG-mir-1-3**	2.5	196	218	GW881131	similar to Serine/threonine protein kinase [Ricinus communis, EEF39636.1]	binding
	2.5	3	22	GT970645	similar to vesicle-associated membrane protein [Ricinus communis, EEF33458.1]	structural molecule activity
	3.5	443	463	GT981493	similar to 60S acidic ribosomal protein P2 [Ricinus communis, EEF37217.1]	structural molecule activity
	4	86	108	GT969463	similar to JHL23C09.2 [Jatropha curcas, AJ53210.1]	
**EACMV-UG-mir-1-4**	2.5	196	217	GW881131	similar to serine/threonine protein kinase [Ricinus communis, EEF39636.1]	binding
	4	86	107	GT969463	similar to JHL23C09.2 [Jatropha curcas, AJ53210.1]	
	4.5	448	469	GT972521	similar to casein kinase [Ricinus communis, EEF30734.1]	binding
**EACMV-UG-mir-1-5**	2.5	194	216	GW881131	similar to serine/threonine protein kinase [Ricinus communis, EEF39636.1]	binding
	4	3	25	GW876282	similar to serpentine Receptor, class Z family member (srz-94)	
	4.5	539	561	FM890970	similar to LEC14B protein [Ricinus communis, EEF45451.1]	
	4.5	260	282	GW613019	similar to casein kinase [Ricinus communis, EEF36922.1]	binding
	5	130	152	GT970837	similar to hAT family dimerisation domain containing protein [Oryza sativa, ABA98161.2]	binding

**EACMV-UG-mir-1-6**	3	194	215	GW881131	similar to serine/threonine protein kinase [Ricinus communis, EEF39636.1]	binding
	3.5	539	560	FM890970	similar to LEC14B protein [Ricinus communis, EEF45451.1]	
	3.5	141	161	GT972943	similar to growth-regulating factor 2 [Arabidopsis thaliana, NP_195488.2]	binding
	4	5	26	FM893965	similar to dimethyladenosine transferase [Ricinus communis, EEF33247.1]	catalytic activity
	4	474	494	GT976424	similar to prenylcysteine oxidase [Arabidopsis thaliana, NP_201196.1]	catalytic activity
	4.5	441	462	GW879570	similar to lecithine cholesterol acyltransferase-like protein	catalytic activity
	4.5	157	178	GW876220	similar to protein MEI2-like 2-like [Glycine max, XP_003533847.1]	binding
	5	129	151	GT970837	similar to hAT family dimerisation domain containing protein [Oryza sativa, ABA98161.2]	binding
**EACMV-UG-mir-1-7**	3	5	25	FM893965	similar to dimethyladenosine transferase [Ricinus communis, EEF33247.1]	catalytic activity
	3.5	194	214	GW881131	similar to Serine/threonine protein kinase [Ricinus communis, EEF39636.1]	binding
	3.5	157	177	GW876220	similar to RNA-binding protein [Ricinus communis, EEF28317.1]	binding
	4	441	461	GW879570	similar to lecithine cholesterol acyltransferase-like protein [Medicago truncatula, AAN77002.1]	catalytic activity
	4	539	559	FM890970	similar to LEC14B protein [Ricinus communis, EEF45451.1]	
	4	173	192	GT969517	similar to JHL23C09.2 [Jatropha curcas, AJ53210.1]	
**EACMV-UG-mir-1-8**	3	5	24	FM893965	similar to dimethyladenosine transferase [Ricinus communis, EEF33247.1]	catalytic activity
	3	603	622	GW878950	similar to ycf2 gene product (chloroplast) [Ricinus communis, AEJ82556.1]	binding
	3.5	473	492	GT976424	similar to prenylcysteine oxidase [Arabidopsis thaliana, NP_201196.1]	catalytic activity
	3.5	155	176	GW876220	similar to RNA-binding protein [Ricinus communis, EEF28317.1]	binding
	4.5	276	297	FM894157	similar to alpha-amylase/subtilisin inhibitor precursor [Ricinus communis, EEF36475.1]	enzyme regulator activity
	5	212	233	GW880718	similar to nonsense-mediated mRNA decay protein [Ricinus communis, EEF33616.1]	binding
**EACMV-UG-mir-1-9**	3	206	225	GT978456	similar to selenium-binding protein [Ricinus communis, EEF41749.1]	binding
	3	155	174	GW876220	similar to RNA-binding protein [Ricinus communis, EEF28317.1]	binding
	3.5	101	120	GT969953	similar to sphingolipid delta 4 desaturase/C-4 hydroxylase protein des2 [Ricinus communis, EEF43254.1]	catalytic activity
	3.5	494	513	FM892999	similar to seed storage protein [Ricinus communis, AF262999_1]	nutrient reservoir activity
	4	180	199	FM896713	similar to annexin [Ricinus communis, EEF36703.1]	binding
	4	411	430	GW614680	similar to alpha-galactosidase/alpha-n-acetylgalactosaminidase [Ricinus communis, EEF49661.1]	catalytic activity
	4	98	117	FM889814	similar to alpha-galactosidase [Vitis vinifera, CBI25379.3]	catalytic activity
	4	212	231	GW880718	similar to nonsense-mediated mRNA decay protein [Ricinus communis, EEF33616.1]	binding

	4	612	631	GT980733	similar to nibrin [Ricinus communis, EEF36478.1]	
	4.5	439	458	GW879570	similar to phosphatidylcholine acyltransferase [Ricinus communis, EEF35850.1]	catalytic activity
	4.5	86	105	FM896303	similar to transcription factor ICE1 [Ricinus communis, EEF51703.1]	binding
	4.5	454	473	FM895444	similar to nucleic acid binding protein [Ricinus communis, EEF35929.1]	binding
**EACMV-UG-mir-1-10**	4	442	463	GW877638	similar to orf49 gene product (mitochondrion) [Daucus carota subsp. sativus, YP_006291838.1]	
	4.5	219	240	GT976657	similar to ATP-dependent Clp protease proteolytic subunit [Ricinus communis, EEF29744.1]	binding
**EACMV-UG-mir-1-11**	3	483	502	GW616967	similar to hydrolase, hydrolyzing O-glycosyl compounds [Ricinus communis, EEF29960.1]	binding
	4	266	285	GW880649	similar to RNA binding protein [Ricinus communis, EEF34976.1]	binding
	4	218	237	GT976657	similar to ATP-dependent Clp protease proteolytic subunit [Ricinus communis, EEF29744.1]	binding
	4	340	359	FM891821	similar to HVA22 [Ricinus communis, EEF52162.1]	
**EACMV-UG-mir-1-12***	3	507	531	GW613377	similar to endomembrane protein emp70 [Ricinus communis, EEF31705.1]	
	3.5	241	264	GW612571	similar to annexin [Ricinus communis, EEF49584.1]	binding
	3.5	235	258	GW614450	similar to GATA transcription factor [Ricinus communis, EEF35041.1]	nucleic acid binding transcription factor activity
	3.5	144	165	GW881306	similar to U2 small nuclear ribonucleoprotein A [Ricinus communis, EEF50164.1]	binding
	3.5	143	164	GW881301	similar to 40S ribosomal protein S2 [Ricinus communis, EEF48884.1]	structural molecule activity
	3.5	230	252	FM893546	similar to 2S albumin precursor [Ricinus communis, EEF39545.1]	nutrient reservoir activity
	3.5	514	536	FM890782	similar to elongation factor 1 gamma [Ricinus communis, EEF35927.1]	binding
	4	125	146	FM891294	similar to protein binding protein [Ricinus communis, EEF50870.1]	binding
	4	72	94	GT975838	similar to JHL23C09.1 [Jatropha curcas, BAJ53209.1]	
	4.5	184	207	GW618554	similar to ring finger protein [Ricinus communis, EEF45119.1]	binding
	4.5	256	276	GW617297	similar to DNA-binding protein [Ricinus communis, EEF52876.1]	binding
	4.5	604	627	GW876940	similar to calmodulin [Ricinus communis, EEF29019.1]	binding
**EACMV-UG-mir-1-13***	3.5	127	149	GT228485	similar to similar to brain protein [Ricinus communis, EEF43789.1]	
	3.5	144	164	GW881305	similar to defective in cullin neddylation protein [Ricinus communis, EEF35070.1]	binding
	4.5	280	303	FM888790	similar to ATP synthase [Ricinus communis, EEF26828.1]	binding
**EACMV-UG-mir-1-14***	3.5	241	262	GW612570	similar to conserved hypothetical protein [Ricinus communis, EEF44682.1]	

	3.5	235	256	GW614449	similar to conserved hypothetical protein [Ricinus communis, EEF52573.1]	
	4.5	469	490	FM889758	similar to legumin B precursor [Ricinus communis, AAF73007.1]	nutrient reservoir activity
	5	400	423	FM890608	similar to cell differentiation protein rcd1 [Ricinus communis, EEF32925.1]	
	5	146	166	FM887456	similar to plasminogen activator inhibitor 1 RNA-binding protein [Ricinus communis, EEF30592.1]	binding
**EACMV-UG-mir-1-15***	3.5	20	39	FM889529	similar to gc-rich sequence DNA-binding factor [Ricinus communis, EEF49657.1]	nucleic acid binding transcription factor activity
	4	143	162	GW881300	similar to defective in cullin neddylation protein [Ricinus communis, EEF35070.1]	binding
	4	334	353	GO246967	similar to dfg10 protein [Ricinus communis, EEF30870.1]	catalytic activity
	4.5	471	490	FM889758	similar to legumin B precursor [Ricinus communis, AAF73007.1]	nutrient reservoir activity
	5	25	44	GT979981	similar to nuclear RNA-binding protein [Ricinus communis, EEF50247.1]	binding
	5	246	265	FM891048	similar to calcyclin-binding protein [Ricinus communis, EEF42655.1]	
**EACMV-UG-mir-1-16***	3.5	469	489	FM889758	similar to legumin B precursor [Ricinus communis, AAF73007.1]	nutrient reservoir activity
	5	568	591	FM894604	similar to small glutamine-rich tetratricopeptide repeat-containing protein A [Ricinus communis, EEF38119.1]	binding
	5	399	422	FM890608	similar to cell differentiation protein rcd1 [Ricinus communis, EEF32925.1]	
**EACMV-UG-mir-1-17***	3.5	678	698	GW875572	similar to hydrolase, hydrolyzing O-glycosyl compounds [Ricinus communis, EEF41796.1]	binding
	3.5	566	587	FM894604	similar to small glutamine-rich tetratricopeptide repeat-containing protein A [Ricinus communis, EEF38119.1]	binding
	4	301	324	GT978124	similar to acyl-CoA oxidase [Ricinus communis, EEF37074.1]	catalytic activity
	4	381	403	GW618350	similar to serine/threonine protein phosphatase [Ricinus communis, EEF40372.1]	binding
**EACMV-UG-mir-1-18***	3.5	678	697	GW875572	similar to hydrolase, hydrolyzing O-glycosyl compounds [Ricinus communis, EEF41796.1]	binding
	4	381	402	GW618350	similar to serine/threonine protein phosphatase [Ricinus communis, EEF40372.1]	binding
	4	566	587	FM894604	similar to small glutamine-rich tetratricopeptide repeat-containing protein A [Ricinus communis, EEF38119.1]	binding
	4	185	206	GT979021	similar to acyl-CoA oxidase [Ricinus communis, EEF37074.1]	catalytic activity
	5	241	260	GW880794	similar to nucleoredoxin [Ricinus communis, EEF52780.1]	binding
**EACMV-UG-mir-1-19***	3.5	130	150	GT980901	similar to phosphoglucomutase [Ricinus communis, EEF34593.1]	binding
	4	368	388	GT974660	similar to calcineurin B [Ricinus communis, EEF30215.1]	binding
	4.5	231	253	GT973837	similar to cytosolic malate dehydrogenase [Malus x domestica]	catalytic activity
	5	295	317	GT978124	similar to acyl-CoA oxidase [Ricinus communis, EEF37074.1]	catalytic activity
**EACMV-UG-mir-2-1**	3	244	263	FM891923	nucleic acid binding proteine [Ricinus communis, EEF43170.1]	binding

	3.5	227	247	GT976941	similar to 60S acidic ribosomal protein P2 [Ricinus communis, EEF37217.1]	structural molecule activity
	3.5	247	267	FM891901	similar to transferase, transferring glycosyl groups [Ricinus communis, EEF34254.1]	catalytic activity
	3.5	325	344	GT980765	similar to nuclear acid binding protein [Ricinus communis, EEF43723.1]	binding
	4	159	178	GW880840	similar to lipoxygenase [Ricinus communis, EEF49838.1]	binding
	4.5	158	177	FM888931	similar to cytochrome P450 [Ricinus communis, EEF48632.1]	electron carrier activity
	5	539	559	GO246873	similar to ubiquitin-conjugating enzyme E2 [Ricinus communis, EEF51304.1]	binding
	5	143	162	GT976141	similar to protein disulfide isomerase [Ricinus communis, EEF28509.1]	electron carrier activity
**EACMV-UG-mir-2-2**	4	329	349	GT980765	similar to nuclear acid binding protein [Ricinus communis, EEF43723.1]	binding
	4	158	179	FM887573	similar to esterase precursor [Ricinus communis, EEF27799.1]	catalytic activity
	4.5	270	292	GT977483	similar to lyase [Ricinus communis, EEF35024.1]	binding
	4.5	287	306	GT978064	similar to nuclear acid binding protein [Ricinus communis, EEF43723.1]	binding
**EACMV-UG-mir-2-4***	3.5	353	372	GT975480	similar to signal transducer [Ricinus communis, EEF44718.1]	molecular transducer activity
	4	290	310	GT229327	similar to hydrolase [Ricinus communis, EEF51791.1]	catalytic activity
	4.5	198	217	GW877310	similar to respiratory burst oxidase [Ricinus communis, EEF51661.1	catalytic activity
	4.5	225	245	GW876696	similar to glycosyltransferase [Ricinus communis, EEF41406.1	catalytic activity
	4.5	652	671	GT982597	similar to ADP,ATP carrier protein [Ricinus communis, EEF30484.1]	transporter activity
	4.5	363	384	GT981516	similar to zinc finger protein [Ricinus communis, EEF30066.1]	binding
	5	93	113	GT973048	similar to glutamyl-tRNA synthetase 1, 2 [Ricinus communis, EEF29910.1]	binding
	5	268	288	GT981519	similar to heat shock factor protein [Ricinus communis, EEF51130.1]	nucleic acid binding transcription factor activity
	5	360	379	GW875411	similar to ATP binding protein [Ricinus communis, EEF40511.1	binding
	5	29	48	GW879662	similar to respiratory burst oxidase [Ricinus communis, EEF51661]	catalytic activity
	5	115	135	FM891335	similar to skp1 [Ricinus communis, EEF52763.1]	
	5	429	448	FM889857	similar to elongation factor 1-gamma-like [Vitis vinifera, XP_002278756.2]	binding
	5	326	345	GT981122	similar to mitochondrial oxoglutarate/malate carrier protein [Ricinus communis, EEF30324.1]	transporter activity
**EACMV-UG-mir-2-5***	4	114	134	FM891335	similar to skp1 [Ricinus communis, EEF52763.1]	
	4	93	112	GT973048	similar to glutamyl-tRNA synthetase 1, 2 [Ricinus communis, EEF29910.1]	binding
	4	103	123	GW614653	similar to skp1 [Ricinus communis, EEF52763.1]	
	4	650	670	GT982597	similar to ADP,ATP carrier protein [Ricinus communis, EEF30484.1]	transporter activity
	4	226	245	GT976508	similar to glycosyltransferase [Ricinus communis, EEF41406.1	catalytic activity

	4.5	267	287	GT981519	similar to heat shock factor protein [Ricinus communis, EEF51130.1]	nucleic acid binding transcription factor activity
	4.5	352	371	GT975480	similar to signal transducer [Ricinus communis, EEF44718.1]	molecular transducer activity
	4.5	362	383	GT981516	similar to zinc finger protein [Ricinus communis, EEF30066.1]	binding
	5	13	33	GT981795	similar to H/ACA ribonucleoprotein complex subunit 4-like [Glycine max]	binding
**EACMV-UG-mir-2-6***	3.5	112	132	FM891335	similar to skp1 [Ricinus communis, EEF52763.1]	
	4.5	84	104	GT979293	similar to cysteine protease inhibitor [Ricinus communis, EEF36811.1]	enzyme regulator activity
	4.5	6	26	GW613333	similar to ADP-ribosylation factor [Medicago truncatula, AES74425.1]	binding
**EACMV-UG-mir-2-7***	3	378	397	GW874836	similar to conserved oligomeric golgi complex component [Ricinus communis, EEF51806.1]	
	3.5	26	45	FM891122	similar to pyridoxin biosynthesis protein PDX1 [Ricinus communis, EEF46478.1]	catalytic activity
	4	231	250	GW619538	similar to shwachman-Bodian-Diamond syndrome protein [Ricinus communis, EEF42033.1]	
	4	135	154	GT981577	similar to transcription factor VIP1-like [Vitis vinifera]	nucleic acid binding transcription factor activity
	4	609	628	GW615796	similar to r2r3-myb transcription factor [Ricinus communis, EEF31422.1]	binding
	4.5	370	389	FM896654	similar to 30S ribosomal protein S5 [Ricinus communis, EEF38399.1]	structural molecule activity
**EACMV-UG-mir-2-8***	3.5	229	248	GW619538	similar to shwachman-Bodian-Diamond syndrome protein [Ricinus communis, EEF42033.1]	
	4	10	29	GT972153	similar to conserved oligomeric golgi complex component [Ricinus communis, EEF51806.1]	
	4.5	294	315	GT979800	similar to acetyl-CoA C-acyltransferase	catalytic activity
	4.5	24	43	FM891122	similar to pyridoxin biosynthesis protein PDX1 [Ricinus communis, EEF46478.1]	catalytic activity
	4.5	614	634	GT228513	similar to zinc finger protein [Ricinus communis, EEF28461.1]	binding
	4.5	373	394	GO247186	similar to PPLZ12 [Ricinus communis, EEF51517.1]	
	4.5	207	228	GT229035	similar to auxin-induced protein AUX22 [Ricinus communis, EEF44692.1]	nucleic acid binding transcription factor activity
	5	654	674	GT982597	similar to ADP, ATP carrier protein [Ricinus communis, EEF30484.1]	transporter activity
	5	379	400	GW615654	similar to acid phosphatase 1 precursor [Ricinus communis, EEF48149.1]	catalytic activity

Table S6 Predicted putative targets of miRs/miRs* from ACMV and EACMV-UG in cassava ESTs using RNAhybrid

miR/miR*	predicted miRNA and target contig	MFE	Start position	Genbank accession	Target description	Target function
**ACMV-mir-1-1**	target 5' A G U 3'<br>AGG AUG ACGCUAUUCGUUGCU<br>UCC UAU UGCGGUAAGUAACGA<br>miRNA 3' G A G 5'	-31.8	53	DB932220	similar to predicted protein [Populus trichocarpa, EEE91112.1]	
**ACMV-mir-1-2**	target 5' U C A G 3'<br>UAUAU CGCCAU UCAUUGU<br>AUAUG GCGGUA AGUAACG<br>miRNA 3' U A 5'	-25.7	400	FF535400	similar to gamma-interferon-inducible lysosomal thiol reductase precursor [Ricinus communis, EEF34301.1]	catalytic activity
	target 5' G A C 3'<br>GUACACGCCAUUUG UGC<br>UAUGUGCGGUAAGU ACG<br>miRNA 3' A A A 5'	-29.3	453	DB949248	similar to glyceraldehyde 3-phosphate dehydrogenase A subunit [Arabidopsis thaliana, BAD93961.1]	binding
**ACMV-mir-1-3**	target 5' G A 3'<br>AGGUAUAUAUGUCAUUUAU<br>UCCAUAUGUGCGGUAAGUA<br>miRNA 3' G AC 5'	-30.1	457	DV448856	similar to ankyrin-repeat containing protein [Ricinus communis, EEF49533.1]	binding
	target 5' G A A 3'<br>AGGUG GC ACGUUGUUCAUUG<br>UCCAU UG UGCGGUAAGUAAC<br>miRNA 3' G A 5'	-26.5	300	DB938699	similar to cytochrome P450 [Ricinus communis, EEF27920.1]	electron carrier activity
**ACMV-mir-1-4**	target 5' U U C G 3'<br>UCUGGG UAUGC UGUCAUUUAU<br>GGGUCC AUAUG GCGGUAAGUA<br>miRNA 3' U 5'	-25.7	411	FF534650	similar to casein kinase [Ricinus communis, EEF46395.1]	binding
	target 5' U G A 3'<br>U AGGUAUAUAUGUCAUUUAU	-30.1	455	DV448856	similar to ankyrin-repeat containing protein [Ricinus communis, EEF49533.1]	binding

	G UCCAUAUGUGCGGUAAGUA<br>miRNA 3' G G 5'					
	target 5' U U G A 3'<br>CCCGG UAUG AUGCCGUUUAU<br>GGGUC AUAU UGCGGUAAGUA<br>miRNA 3' C G 5'	-30.2	500	DB923039	similar to nucleoside transporter [Ricinus communis, EEF36986.1]	transporter activity
	target 5' G A G 3'<br>CCAG UAU UACGCUAUUUGU<br>GGUC AUA GUGCGGUAAGUA<br>miRNA 3' G C U 5'	-25	622	FF380083	similar to sigma factor sigb regulation protein rsbq [Ricinus communis, EEF52288.1]	catalytic activity
**ACMV-mir-1-5**	target 5' U G U 3'<br>UUUU AGGUAUAUAUGUCAUUUA<br>AAGG UCCAUAUGUGCGGUAAGU<br>miRNA 3' G 5'	-30.8	452	DV448856	similar to ankyrin-repeat containing protein [Ricinus communis, EEF49533.1]	binding
	target 5' G U G U 3'<br>UCCCGG UAUG AUGCCGUUUA<br>AGGGUC AUAU UGCGGUAAGU<br>miRNA 3' A C G 5'	-32.3	499	DB923039	similar to nucleoside transporter [Ricinus communis, EEF36986.1]	transporter activity
	target 5' A C C 3'<br>UUUUCAGG AUGUGCGUUGUUCA<br>AAGGGUCC UAUGUGCGGUAAGU<br>miRNA 3' A 5'	-26.3	404	DB944787	similar to zinc finger protein [Ricinus communis, EEF34907.1]	binding
	target 5' G A A U 3'<br>UUCCCGGGUA A GUGUUGUUCA<br>AAGGGUCCAU U UGCGGUAAGU<br>miRNA 3' A G 5'	-27.2	469	FF382006	similar to big map kinase/bmk [Ricinus communis, EEF33894.1]	binding
**ACMV-mir-1-6**	target 5' C G U 3'<br>UUUUU AGGUAUAUAUGUCAU<br>AAAGG UCCAUAUGUGCGGUA<br>miRNA 3' AU G 5'	-28.5	451	DV448856	similar to ankyrin-repeat containing protein [Ricinus communis, EEF49533.1]	binding

	target 5' C U U G 3'<br>AUUUUCU GGU UGUAUGCCAU<br>UAAAGGG CCA AUGUGCGGUA<br>miRNA 3' A U U 5'	-25.5	335	DV443989	similar to translation initiation factor if-3 [Ricinus communis, EEF29148.1]	binding
**ACMV-mir-1-7**	target 5' C U U 3'<br>UGUUUAUUUCCCAG GUAUAU<br>ACAAAUAAAGGGUC CAUAUG<br>miRNA 3' U 5'	-30.7	210	DB940121	similar to methylglutaconyl-CoA hydratase, mitochondrial precursor [Ricinus communis, EEF30028.1]	binding
**ACMV-mir-1-8**	target 5' U U U 3'<br>GUUUAUUUCCCAG GUAUAU<br>CAAAUAAAGGGUC CAUAUG<br>miRNA 3' U 5'	-28.5	211	DB940121	similar to methylglutaconyl-CoA hydratase, mitochondrial precursor [Ricinus communis, EEF30028.1]	binding
**ACMV-mir-1-9***	target 5' U A U 3'<br>UGGU U UUGUUGGUGCUUG<br>ACCA G AACAACCACGGAC<br>miRNA 3' U C C 5'	-27.6	306	FF535752	similar to histone h2a [Ricinus communis, EEF44725.1]	binding
	target 5' C A G 3'<br>AUG UC UUGUUGGUGCUUGG<br>UAC AG AACAACCACGGACC<br>miRNA 3' C C 5'	-31.7	368	DB923743	similar to zinc finger protein [Ricinus communis, EEF50383.1]	binding
**ACMV-mir-1-11***	target 5' A U A 3'<br>GAAUG UCGUUGUU GUGCCUGG<br>CUUAC AGCAACAA CACGGACC<br>miRNA 3' C C C 5'	-37.5	261	DB928811	similar to unnamed protein product [Vitis vinifera, CBI36534.3]	
**ACMV-mir-1-10***	target 5' G A A C 3'<br>GGGUG UUGUU UUGGUG CUGG<br>CUUAC AGCAA AACCAC GACC<br>miRNA 3' C C G 5'	-25.9	527	FF535089	similar to RNA binding protein [Ricinus communis, EEF50738.1]	binding

	target 5' A U A C 3'<br>GAGUG GU GUUG UGGUGC UUGG<br>CUUAC CA CAAC ACCACG GACC<br>miRNA 3' G A 5'	-28.1	346	FF381629	similar to mitochondrial carrier protein [Ricinus communis, EEF36204.1]	binding
**ACMV-mir-1-12***	target 5' U U A 3'<br>AGGGAU GUUGUUGUUG UGUUUG<br>UCCUUA CAGCAACAAC ACGGAC<br>miRNA 3' C C 5'	-30.9	126	DB945207	similar to protein binding protein [Ricinus communis, EEF44516.1]	binding
**ACMV-mir-1-13***	target 5' A A U G 3'<br>GG GGAUGGUUGUUGUUG UGU<br>UC CUUACCAGCAACAAC ACG<br>miRNA 3' CG C G 5'	-31.8	167	DB943003	similar to hydrolase [Ricinus communis, EEF33634.1]	binding
**ACMV-mir-1-14***	target 5' U U A 3'<br>GUGGGA UGGUUGUUGUUGG<br>CGUCCU ACCAGCAACAACC<br>miRNA 3' U 5'	-33.6	5	DV458694	similar to phosphoribosylamine-glycine ligase [Ricinus communis, EEF35937.1]	binding
**ACMV-mir-2-1**	target 5' G A A 3'<br>GUCUUUUUCA GUACCCAAA<br>CAGAAAGAGU UAUGGGUUU<br>miRNA 3' G 5'	-29.4	15	DB942360	similar to ribosomal protein S28 [Ricinus communis, XP_002531592.1]	structural molecule activity
	target 5' A G C 3'<br>GUCU UUC CACAUACCCAAA<br>CAGA AAG GUGUAUGGGUUU<br>miRNA 3' A 5'	-28.8	48	DB948720	similar to auxin-induced in root cultures protein 12 precursor [Ricinus communis, EEF30377.1]	
	target 5' C U 3'<br>UUUUUUUCACAUACCCAA<br>AGAAAGAGUGUAUGGGUU<br>miRNA 3' C U 5'	-30	483	DR084721	similar to hexokinase [Ricinus communis, EEF48175.1]	binding
	target 5' U U A 3'<br>UCUUUCUC UGUACCCAAA	-28.7	22	CK643553	similar to serine/threonine protein phosphatase [Ricinus communis]	binding

	AGAAAGAG GUAUGGGUUU<br>miRNA 3' C U 5'					
	target 5' C U 3'<br>UUUUCUUGCGUGCCCAAA<br>GAAAGAGUGUAUGGGUUU<br>miRNA 3' CA 5'	-27.9	25	DV456975	similar to E3 ubiquitin-protein ligase UPL5-like [Glycine max, XP_003528704.1]	catalytic activity
	target 5' G G U 3'<br>CUUUUUUA GUGCCCAGA<br>GAAAGAGU UAUGGGUUU<br>miRNA 3' CA G 5'	-25.9	32	DB944295	similar to fructose-bisphosphate aldolase [Ricinus communis, EEF49496.1]	catalytic activity
	target 5' C G 3'<br>UUUUUC CACAUACCCAAG<br>AGAAAG GUGUAUGGGUUU<br>miRNA 3' C A 5'	-28.2	180	FF534499	similar to ATP binding protein [Ricinus communis, EEF39917.1]	binding
**ACMV-mir-2-2**	target 5' A A 3'<br>GUCUUUC CAUAUACUCAG<br>CAGAAAG GUGUAUGGGUU<br>miRNA 3' UUA A 5'	-28.5	366	DB952841	similar to kinase [Ricinus communis, EEF32437.1]	binding
	target 5' C A G U 3'<br>GUCUU UCUCG AUACCCAG<br>CAGAA AGAGU UAUGGGUU<br>miRNA 3' UUA G 5'	-29.9	58	DB922743	similar to n-rich protein [Ricinus communis, EEF43380.1]	
	target 5' C C A 3'<br>UG UUUUCUUGCGUGCCCAA<br>AC GAAAGAGUGUAUGGGUU<br>miRNA 3' UU A 5'	-28.8	22	DV456975	similar to E3 ubiquitin-protein ligase UPL5-like [Glycine max, XP_003528704.1]	catalytic activity
	target 5' U A A 3'<br>AAUGUC UUUCUCACGUACUUAA<br>UUACAG AAAGAGUGUAUGGGUU<br>miRNA 3' 5'	-31.8	506	DV442563	similar to sulfite reductase [Ricinus communis, EEF50910.1]	electron carrier activity

	target 5' U A A 3'<br>AAUGUC UUUCUUACGUACUUAA<br>UUACAG AAAGAGUGUAUGGGUU<br>miRNA 3' 5'	-29.6	659	DV450054	similar to metal transporter [Ricinus communis, EEF42120.1]	transporter activity
	target 5' C G U 3'<br>AAUGUUUUUCUCACG AUUCAG<br>UUACAGAAAGAGUGU UGGGUU<br>miRNA 3' A 5'	-27.9	401	DR085317	similar to alternative oxidase 4, chloroplast precursor [Ricinus communis, EEF43798.1]	binding
**ACMV-mir-2-3**	target 5' U A U 3'<br>GAAU UUUUUCUCACAUACUUA<br>CUUA AGAAAGAGUGUAUGGGU<br>miRNA 3' UU C 5'	-29.7	528	DB935433	similar to aspartic proteinase precursor [Ricinus communis, EEF32480.1]	catalytic activity
	target 5' U A A 3'<br>AAUGUC UUUCUCACGUACUUA<br>UUACAG AAAGAGUGUAUGGGU<br>miRNA 3' UUC 5'	-31	506	DV442563	similar to sulfite reductase [Ricinus communis, EEF50910.1]	electron carrier activity
	target 5' C A A U 3'<br>GAG GUCUUU UUGCAUGCCCA<br>CUU CAGAAA AGUGUAUGGGU<br>miRNA 3' UU A G 5'	-29.7	469	FF379968	similar to vesicle transport v-snare protein vti1a [Ricinus communis, EEF32373.1]	
**ACMV-mir-2-4**	target 5' A U A 3'<br>CAAGAA GU CUUUCUCAUAUAUC<br>GUUCUU CA GAAAGAGUGUAUGG<br>miRNA 3' G A 5'	-31.6	273	DV448413	similar to sec15 [Ricinus communis, EEF39290.1]	
	target 5' G A 3'<br>AAGAGUGUUUUUUUCACAUA<br>UUCUUACAGAAAGAGUGUAU<br>miRNA 3' GG GG 5'	-30.6	20	DB925685	similar to heparanase-2 [Ricinus communis, EEF47802.1]	catalytic activity
	target 5' G A U U 3'<br>AAGAG GUUUUUCUCACAU UC	-27.3	369	DV449788	similar to fructokinase [Ricinus communis, EEF29025.1]	catalytic activity

	UUCUU CAGAAAGAGUGUA GG<br>miRNA 3' GG A U 5'					
**ACMV-mir-2-5**	target 5' G A 3'<br>AAGAGUGUUUUUUUCACAUA<br>UUCUUACAGAAAGAGUGUAU<br>miRNA 3' GG G 5'	-30.6	20	DB925685	similar to heparanase-2 [Ricinus communis, EEF47802.1]	catalytic activity
	target 5' G U A A 3'<br>AAGAAU UCUU CUCGCAUAU<br>UUCUUA AGAA GAGUGUAUG<br>miRNA 3' GG C A 5'	-26.1	215	DB934108	similar to receptor protein kinase [Ricinus communis, EEF34847.1]	binding
	target 5' A U C 3'<br>CAAGAA G UCUUUCUCAUAUAU<br>GUUCUU C AGAAAGAGUGUAUG<br>miRNA 3' G A 5'	-28.5	273	DV448413	similar to sec15 [Ricinus communis, EEF39290.1]	
	target 5' U A G C G 3'<br>UCAAGAA G CU UCUCACAUGC<br>GGUUCUU C GA AGAGUGUAUG<br>miRNA 3' A A A 5'	-28.6	400	DB948734	similar to porphobilinogen deaminase [Ricinus communis, EEF42025.1]	binding
	target 5' U C G 3'<br>GGGGAUGUU UUCUUACGUAU<br>UUCUUACAG AAGAGUGUAUG<br>miRNA 3' GG A 5'	-27.4	98	FF379904	similar to translation initiation factor 2b, delta subunit [Ricinus communis, EEF50656.1]	binding
**ACMV-mir-2-6**	target 5' C C G G 3'<br>AAGCUAGG AAUG CUUUCUUA CA<br>UUCGGUUC UUAC GAAAGAGU GU<br>miRNA 3' A A 5'	-27.1	752	FG807088	similar to 2-deoxyglucose-6-phosphate phosphatase, [Ricinus communis, EEF49975.1]	binding
**ACMV-mir-2-7**	target 5' A A C A 3'<br>UAGGCUAA AAU UCUUUCUCA<br>GUUCGGUU UUA AGAAAGAGU<br>miRNA 3' C C GU 5'	-26.3	601	DV451262	similar to fructose-1,6-bisphosphatase, cytosolic, [Ricinus communis, EEF29949.1]	binding

**ACMV-mir-2-8***	target 5' A A A 3'<br>AAAUGAGGA UUCUCGUUUU<br>UUUACUCUU AGGAGCAAAA<br>miRNA 3' AG G C 5'	-27.2	219	DV450633	similar to beta-adaptin-like protein A-like [Glycine max, XP_003522688.1]	transporter activity
	target 5' C U C G 3'<br>UCAGAU GGAACUCU UGUUUUG<br>AGUUUA UCUUGAGG GCAAAAC<br>miRNA 3' C A 5'	-28.1	392	FF380166	similar to chalcone synthase [Ricinus communis, EEF44265.1]	catalytic activity
	target 5' A A A 3'<br>CAGAUGGGAA UCUUUGUUU<br>GUUUACUCUU AGGAGCAAA<br>miRNA 3' A G AC 5'	-26.8	154	DB946837	similar to purine permease [Ricinus communis, EEF52048.1]	transporter activity
**ACMV-mir-3-1**	target 5' C A G 3'<br>AGUUUGUGGA AGCUGCGUC<br>UUAGACAUCU UCGACGUAG<br>miRNA 3' U C 5'	-32.4	246	DV457211	similar to tRNA-pseudouridine synthase [Ricinus communis, EEF40978.1]	binding
	target 5' U U U A 3'<br>GAAUCU U GGGAGCUGUAU<br>UUUAGA A CUCUCGACGUA<br>miRNA 3' C U G 5'	-26.6	248	CK650220	similar to ATP-binding cassette transporter [Ricinus communis, EEF28273.1]	binding
	target 5' U A A 3'<br>GGAUUUGUAGG AGCUGCGU<br>UUUAGACAUCU UCGACGUA<br>miRNA 3' C G 5'	-29.5	459	DB949701	similar to transmembrane protein 14 [Ricinus communis, EEF46177.1]	
	target 5' A U A 3'<br>AAAUCUGUA AGGGUUGUA<br>UUUAGACAU UCUCGACGU<br>miRNA 3' C AG 5'	-27.1	199	DB928794	similar to zinc finger CCCH domain-containing protein [Medicago truncatula, AET03818.1]	binding
**ACMV-mir-3-2**	target 5' U U G 3'	-38.4	47	DB935122	similar to calcineurin B [Ricinus communis, EEF45699.1]	binding

	GAGCUGCAUCAGAGU GAGAA<br>CUCGACGUAGUCUUA CUCUU<br>miRNA 3' U C 5'					
	target 5' A G U A 3'<br>GGA GUUGU UUAGAAUGGAGAA<br>UCU CGACG AGUCUUACCUCUU<br>miRNA 3' U 5'	-31.9	115	DB921700	similar to ATP-binding cassette transporter [Ricinus communis, EEF38310.1]	binding
	target 5' U A A 3'<br>AGCUGUAUUG GAUGGGGAA<br>UCGACGUAGU UUACCUCUU<br>miRNA 3' UC C 5'	-29.4	444	DV458768	similar to transmembrane protein 14 [Ricinus communis, EEF39398.1]	
	target 5' C G C 3'<br>GAGCUGCAUCA GAGUGGAGG<br>CUCGACGUAGU CUUACCUCU<br>miRNA 3' U U 5'	-39.5	131	CK901238	similar to flavonoid 3',5'-hydroxylase [Citrus clementina]	electron carrier activity
	target 5' U U U 3'<br>AGA CUGUGUUGGAAUGGAGA<br>UCU GACGUAGUCUUACCUCU<br>miRNA 3' C U 5'	-32	297	DB944450	similar to ATP-dependent clp protease [Ricinus communis, EEF51704.1]	binding
	target 5' U U U 3'<br>AGA CUGUGUUGGAAUGGAGA<br>UCU GACGUAGUCUUACCUCU<br>miRNA 3' C U 5'	-32	348	DB952898	similar to methionine-tRNA synthetase [Ricinus communis, EEF44189.1]	binding
**ACMV-mir-3-3**	target 5' A U A 3'<br>GGAG GUUGU UUAGAAUGGAGA<br>UCUC CGACG AGUCUUACCUCU<br>miRNA 3' A U U 5'	-35	115	DB921275	similar to ATP-binding cassette transporter [Ricinus communis, EEF38310.1]	binding
	target 5' A A U A 3'<br>UGG AGA UUGCAUUA AAUGGAGA<br>AUC UCU GACGUAGU UUACCUCU<br>miRNA 3' C C 5'	-29.1	194	DV444797	similar to metalloendoproteinase 1 precursor [Ricinus communis, EEF28446.1]	binding

	target 5' U U U 3'<br>AGA CUGUGUUGGAAUGGAGA<br>UCU GACGUAGUCUUACCUCU<br>miRNA 3' AUC C 5'	-31.9	348	DB952898	similar to methionine-tRNA synthetase [Ricinus communis, EEF44189.1]	binding
**ACMV-mir-3-4***	target 5' U C 3'<br>UCUCUCUCUC UCAAACCCUAA<br>AGAGAGAGGG AGUUUGGGAUU<br>miRNA 3' G U 5'	-38.7	109	DB925174	similar to DNA-binding protein MNB1B [Ricinus communis, EEF52876.1]	binding
	target 5' U C C 3'<br>CUCUUUCUCCC UUAAACCCU<br>GAGAGAGAGGG AGUUUGGGA<br>miRNA 3' U UU 5'	-36.3	41	DV455184	similar to DAG protein, chloroplast precursor [Ricinus communis, EEF43977.1]	
	target 5' U C 3'<br>UCUCUCUCUC UCAAACCCUAA<br>AGAGAGAGGG AGUUUGGGAUU<br>miRNA 3' G U 5'	-38.7	38	DV458538	similar to cold-inducible RNA-binding protein [Ricinus communis, EEF38570.1]	binding
	target 5' U U A 3'<br>CUCUCUCUCCU UCGAGCCCUAG<br>GAGAGAGAGGG AGUUUGGGAUU<br>miRNA 3' U 5'	-41.4	46	DV449925	similar to actin depolymerizing factor 4 [Hevea brasiliensis, ADV04049.1]	
	target 5' C A C U 3'<br>CUCUCUC CUU UCAAAUCCUAA<br>GAGAGAG GGG AGUUUGGGAUU<br>miRNA 3' A U 5'	-31	106	DR085604	similar to 60S ribosomal protein L18aA [Hevea brasiliensi, ADR71269.1s]	structural molecule activity
**ACMV-mir-3-5***	target 5' U G 3'<br>UAUUCUCUCUCCC UCAAACCCUA<br>GUGAGAGAGAGGG AGUUUGGGAU<br>miRNA 3' U 5'	-43.3	64	DV458538	similar to cold-inducible RNA-binding protein [Ricinus communis, EEF38570.1]	binding
	target 5' U U G 3'	-41.4	46	DV449925	similar to actin depolymerizing factor 4 [Hevea brasiliensis, ADV04049.1]	

	CUCUCUCUCCU UCGAGCCCUA<br>GAGAGAGAGGG AGUUUGGGAU<br>miRNA 3' GU U 5'					
	target 5' G C U A 3'<br>AUUCUUUU CCU UCAAAUCCUA<br>UGAGAGAG GGG AGUUUGGGAU<br>miRNA 3' G A U 5'	-30.1	129	DB920502	similar to protein binding protein [Ricinus communis, EEF45740.1]	binding
	target 5' U C C 3'<br>CUCUUUCUCCC UUAAACCCU<br>GAGAGAGAGGG AGUUUGGGA<br>miRNA 3' GU U U 5'	-36.9	57	DV446437	similar to DAG protein, chloroplast precursor [Ricinus communis, EEF43977.1]	
	target 5' C C G U 3'<br>CAC CUCUU CUCAUCAAACUUUA<br>GUG GAGAG GGGUAGUUUGGGAU<br>miRNA 3' A A 5'	-31.4	725	DV445323	similar to arginine/serine-rich-splicing factor [Ricinus communis, EEF51221.1]	binding
	target 5' G A C 3'<br>UACU UUC CUCCUAUCAAACUCUG<br>GUGA GAG GAGGGUAGUUUGGGAU<br>miRNA 3' A 5'	-33.2	159	DB949483	similar to ATP synthase [Ricinus communis, EEF30158.1]	binding
	target 5' G C C 3'<br>ACUUUCUCUCCUG CAAACCC<br>UGAGAGAGAGGGU GUUUGGG<br>miRNA 3' G A AU 5'	-36.4	2	FF535121	similar to allene oxide cyclase 4, chloroplast precursor [Ricinus communis, EEF31990.1]	catalytic activity
	target 5' C C C 3'<br>CACUUUUUCUUCC UUAAACCUU<br>GUGAGAGAGAGGG AGUUUGGGA<br>miRNA 3' U U 5'	-32.6	310	DB954030	similar to receptor serine-threonine protein kinase [Ricinus communis, EEF43165.1]	binding
**ACMV-mir-3-6***	target 5' U A 3'<br>UAUUCUCUCUCCC UCAAACCCU<br>GUGAGAGAGAGGG AGUUUGGGA<br>miRNA 3' U U 5'	-42.5	64	DV451648	similar to cold-inducible RNA-binding protein [Ricinus communis, EEF38570.1]	binding

	target 5' U U A 3'<br>CUCUCUCUCCU UCGAGCCCU<br>GAGAGAGAGGG AGUUUGGGA<br>miRNA 3' UGU U 5'	-40	46	DV449925	similar to actin depolymerizing factor 4 [Hevea brasiliensis, ADV04049.1]	
	target 5' G C C 3'<br>AUUCUCUCUCCCAUCAA CCC<br>UGAGAGAGAGGGUAGUU GGG<br>miRNA 3' UG U A 5'	-40.7	117	FF534285	similar to transferase, transferring glycosyl groups [Ricinus communis, EEF41222.1]	catalytic activity
	target 5' G U U C 3'<br>GCG C CUCUCUCCC UCAAAUUCU<br>UGU G GAGAGAGGG AGUUUGGGA<br>miRNA 3' A U 5'	-33.4	116	DB937863	similar to alcohol dehydrogenase [Ricinus communis, EEF28016.1]	binding
	target 5' C C C 3'<br>CACUUUUUCUUCC UUAAACCUU<br>GUGAGAGAGAGGG AGUUUGGGA<br>miRNA 3' U U 5'	-33	310	DB954030	similar to receptor serine-threonine protein kinase, [Ricinus communis, EEF43165.1]	binding
	target 5' G C C 3'<br>ACUUUCUCUCCUG CAAACCC<br>UGAGAGAGAGGGU GUUUGGG<br>miRNA 3' UG A A 5'	-36.4	2	FF535121	similar to allene oxide cyclase 4, chloroplast precursor [Ricinus communis, EEF31990.1]	catalytic activity
	target 5' G A G 3'<br>UACU UUC CUCCUAUCAAACUCU<br>GUGA GAG GAGGGUAGUUUGGGA<br>miRNA 3' U A 5'	-33	159	DB949483	similar to ATP synthase [Ricinus communis, EEF30158.1]	binding
	target 5' A U A 3'<br>AC UUCUCUUUUUCAUCAGACUC<br>UG GAGAGAGAGGGUAGUUUGGG<br>miRNA 3' U A 5'	-33.5	427	DB950434	similar to heat shock protein [Ricinus communis, EEF43398.1]	binding
**ACMV-mir-3-7***	target 5' C U A 3'	-34.2	426	DB950434	similar to heat shock protein [Ricinus communis, EEF43398.1]	binding

	AAC UUCUCUUUUUCAUCAGACUC<br>UUG GAGAGAGAGGGUAGUUUGGG<br>miRNA 3' U 5'					
	target 5' C C U 3'<br>CACUUUUUCUUCC UUAAACCU<br>GUGAGAGAGAGGG AGUUUGGG<br>miRNA 3' UU U 5'	-32.4	310	DB954030	similar to receptor serine-threonine protein kinase, [Ricinus communis, EEF43165.1]	binding
	target 5' G A U 3'<br>UACU UUC CUCCUAUCAAACUC<br>GUGA GAG GAGGGUAGUUUGGG<br>miRNA 3' UU A 5'	-31.4	159	DB949483	similar to ATP synthase [Ricinus communis, EEF30158.1]	binding
	target 5' C C A 3'<br>AAC UUUUCUUUUCCAUCAAAU<br>UUG GAGAGAGAGGGUAGUUUG<br>miRNA 3' U GG 5'	-29.3	236	DB954572	similar to SWIB/MDM2 domain-containing protein [Arabidopsis thaliana, AAF03473.1]	binding
**ACMV-mir-3-8***	target 5' G G A U 3'<br>AAAUA U UUUUUCUCAUCAAACU<br>UUUGU A AGAGAGGGUAGUUUGG<br>miRNA 3' G G 5'	-27	229	DB952257	similar to calmodulin [Glycine max, NP_001236711.1]	binding
	target 5' C U C 3'<br>AAC UUCUCUUUUUCAUCAGACU<br>UUG GAGAGAGAGGGUAGUUUGG<br>miRNA 3' U U 5'	-31.2	426	DB950434	similar to heat shock protein [Ricinus communis, EEF43398.1]	binding
	target 5' U A 3'<br>ACACUCUCUUUUCCAUCAGA<br>UGUGAGAGAGAGGGUAGUUU<br>miRNA 3' UU GG 5'	-36.5	108	CK643811	similar to phenylalanyl-tRNA synthetase [Ricinus communis, EEF33425.1]	binding
	target 5' C C A 3'<br>AAC UUUUCUUUUCCAUCAAAU<br>UUG GAGAGAGAGGGUAGUUUG	-29.4	236	DB954572	similar to SWIB/MDM2 domain-containing protein [Arabidopsis thaliana, AAF03473.1]	binding

	miRNA 3' U U G 5'					
	target 5' U A A A 3'<br>AGACAUU UUUCUCC AUCAAAU<br>UUUGUGA AGAGAGG UAGUUUG<br>miRNA 3' G G G 5'	-27.6	191	DV447511	similar to 60S ribosomal protein L13aA [Hevea brasiliensis, ADR71263.1]	structural molecule activity
**ACMV-mir-3-9***	target 5' C C A 3'<br>AAC UUUUCUUUUCCAUCAAAU<br>UUG GAGAGAGAGGGUAGUUUG<br>miRNA 3' GU U 5'	-29	236	DB954572	similar to SWIB/MDM2 domain-containing protein [Arabidopsis thaliana, AAF03473.1]	binding
	target 5' C A A 3'<br>UAGACA UC UUUCUCCCAUCAAAU<br>GUUUGU AG AGAGAGGGUAGUUUG<br>miRNA 3' G 5'	-32.3	678	FF381206	similar to 60S ribosomal protein L13aA [Hevea brasiliensis, ADR71263.1]	structural molecule activity
	target 5' A G U 3'<br>AAGCACUUUCUCUCCUG CAAG<br>UUUGUGAGAGAGAGGGU GUUU<br>miRNA 3' G A G 5'	-33.1	168	DV449505	similar to lipoxygenase [Ricinus communis, EEF49219.1]	binding
**ACMV-mir-3-10***	target 5' G A 3'<br>AAAC C CUCUUUUCCAUCAAA<br>UUUG G GAGAGAGGGUAGUUU<br>miRNA 3' G U A 5'	-29	331	CK645990	similar to lysM domain GPI-anchored protein 2 precursor [Ricinus communis, EEF36585.1l]	binding
	target 5' C C U 3'<br>AAC UUUUCUUUUCCAUCAAA<br>UUG GAGAGAGAGGGUAGUUU<br>miRNA 3' GU U 5'	-27.5	236	DB954572	similar to SWIB/MDM2 domain-containing protein [Arabidopsis thaliana, AAF03473.1]	binding
**ACMV-mir-3-11***	target 5' A C C U 3'<br>UC AAC UUUUCUUUUCCAUCAAA<br>AG UUG GAGAGAGAGGGUAGUUU<br>miRNA 3' U U 5'	-28.7	233	DB954572	similar to SWIB/MDM2 domain-containing protein [Arabidopsis thaliana, AAF03473.1]	binding

	target 5' A G U 3'<br>AAGCACUUUCUCUCCUG CAAG<br>UUUGUGAGAGAGAGGGU GUUU<br>miRNA 3' AG A 5'	-32.9	168	DV449505	similar to lipoxygenase [Ricinus communis, EEF49219.1]	binding
**ACMV-mir-3-12***	target 5' A C C A 3'<br>UC AAC UUUUCUUUUCCAUCAA<br>AG UUG GAGAGAGAGGGUAGUU<br>miRNA 3' A U U 5'	-28.8	233	DB954572	similar to SWIB/MDM2 domain-containing protein [Arabidopsis thaliana, AAF03473.1]	binding
	target 5' U U G 3'<br>UUCA AC CUCUUUCUUUCAUUAA<br>AAGU UG GAGAGAGAGGGUAGUU<br>miRNA 3' U U 5'	-28	392	DB925921	similar to pentatricopeptide repeat-containing protein [Ricinus communis, EEF28596.1]	binding
	target 5' C A G C 3'<br>UUCAGA ACUCUCUCUUUCA CAG<br>AAGUUU UGAGAGAGAGGGU GUU<br>miRNA 3' G A 5'	-31	457	DB938365	similar to 60S ribosomal protein L30 [Ricinus communis, EEF33410.1]	structural molecule activity
**ACMV-mir-3-13***	target 5' G A G G 3'<br>UUCUUCAGA ACUCUCUCUUUCA CA<br>AGGAAGUUU UGAGAGAGAGGGU GU<br>miRNA 3' GA G A 5'	-36.5	454	DB938365	similar to 60S ribosomal protein L30 [Ricinus communis, EEF33410.1]	structural molecule activity
	target 5' C G A U 3'<br>CUUCU U GGGUGUUCUUUCUCCCAUC<br>GAAGG A UUUGUGAGAGAGAGGGUAG<br>miRNA 3' A G U 5'	-29.8	369	DV443985	similar to calmodulin binding protein [Ricinus communis, EEF39751.1]	binding
	target 5' U C A C 3'<br>UUCUUUC AG ACUCUCUCUCUUAUU<br>AAGGAAG UU UGAGAGAGAGGGUAG<br>miRNA 3' G U G U 5'	-34.6	39	DB936996	similar to sodium/hydrogen exchanger [Ricinus communis, EEF49734.1]	transporter activity
	target 5' C G U 3'<br>UUCUUCAGAUGUUCUU CUCCCAUU	-34.1	477	DB949316	similar to gamma-tocopherol methyltransferase [Hevea brasiliensis, BAH10645.1]	catalytic activty

	`           AGGAAGUUUGUGAGAG GAGGGUAG`<br>`miRNA  3' GA                A        U 5'`					
**ACMV-mir-3-14***	`target 5'  G                U  U 3'`<br>`           AAACACUCUCUCUCCU UC`<br>`           UUUGUGAGAGAGAGGG AG`<br>`miRNA  3' AG                U    5'`	-34.7	1	DV444886	similar to glycosyltransferase CAZy family GT8 [Medicago truncatula, AES92758.1]	catalytic activty
	`target 5' G       A            G 3'`<br>`          UCAAGUG UCUCUUUCCCAU`<br>`          AGUUUGU AGAGAGAGGGUA`<br>`miRNA  3'         G            G 5'`	-27.1	379	DB947688	similar to DNA binding protein [Ricinus communis, EEF49890.1]	binding
**ACMV-mir-3-15***	`target 5' G         A              G 3'`<br>`          UUCUUCAGA ACUCUCUCUUUCA`<br>`          AGGAAGUUU UGAGAGAGAGGGU`<br>`miRNA  3' A         G                5'`	-35.5	454	DB938365	similar to 60S ribosomal protein L30 [Ricinus communis, EEF33410.1]	structural molecule activity
	`target 5' U       C  A              U 3'`<br>`          UUCUUUC AG ACUCUCUCUCUUA`<br>`          AAGGAAG UU UGAGAGAGAGGGU`<br>`miRNA  3'         U  G                5'`	-31.7	39	DB932262	similar to sodium/hydrogen exchanger [Ricinus communis, EEF49734.1]	transporter activity
	`target 5' U       C                G 3'`<br>`          UUCCU C AACAUUCUUUCUCCUA`<br>`          AAGGA G UUGUGAGAGAGAGGGU`<br>`miRNA  3'       A U                  5'`	-34.1	387	DB946667	similar to 30S ribosomal protein S8 [Ricinus communis, EEF33949.1]	structural molecule activity
	`target 5' C                    G  3'`<br>`          UUCUUCA ACGCUCUCUCUCU`<br>`          AGGAAGU UGUGAGAGAGAGG`<br>`miRNA  3' A       U             GU 5'`	-35.6	51	FF381792	similar to 50S ribosomal protein L21 [Ricinus communis, EEF35552.1]	structural molecule activity
	`target 5' U       U  A            U 3'`<br>`          UUCCUUC AA ACUCUCUCUCUU`<br>`          AAGGAAG UU UGAGAGAGAGGG`<br>`miRNA  3'         U  G            U 5'`	-33.4	118	DV447647	similar to 50S ribosomal protein L21 [Ricinus communis, EEF35552.1]	structural molecule activity

	target 5' C G U 3'<br>UUCUUCAGAUGUUCUU CUCCCA<br>AGGAAGUUUGUGAGAG GAGGGU<br>miRNA 3' A A 5'	-32	477	DB949316	similar to gamma-tocopherol methyltransferase [Hevea brasiliensis, BAH10645.1]	catalytic activity
	target 5' U U G 3'<br>UUCUUUCAA UAUUCUUUUUCUUA<br>AAGGAAGUU GUGAGAGAGAGGGU<br>miRNA 3' U 5'	-28.9	413	DB952421	similar to photosystem II reaction center protein M [Medicago truncatula, AES87815.1]	
	target 5' C G U 3'<br>UUCUUCAGAUGUUCUU CUCCCA<br>AGGAAGUUUGUGAGAG GAGGGU<br>miRNA 3' A A 5'	-32	423	DB950660	similar to gamma-tocopherol methyltransferase [Hevea brasiliensis, BAH10645.1]	catalytic activity
**ACMV-mir-3-16***	target 5' G A A 3'<br>UUCUUCAGA ACUCUCUCUUUC<br>AGGAAGUUU UGAGAGAGAGGG<br>miRNA 3' GA G 5'	-34.3	454	DB938365	similar to 60S ribosomal protein L30 [Ricinus communis, EEF33410.1]	structural molecule activity
	target 5' C G 3'<br>UUCUUCA ACGCUCUCUCUCU<br>AGGAAGU UGUGAGAGAGAGG<br>miRNA 3' GA U G 5'	-35.6	51	FF381792	similar to 50S ribosomal protein L21 [Ricinus communis, EEF3555]	structural molecule activity
	target 5' U U A U 3'<br>UUCCUUC AA ACUCUCUCUCUU<br>AAGGAAG UU UGAGAGAGAGGG<br>miRNA 3' G U G 5'	-34	122	DB924367	similar to 50S ribosomal protein L21 [Ricinus communis, EEF3555]	structural molecule activity
	target 5' U C A 3'<br>UUCCU C AACAUUCUUUCUCCU<br>AAGGA G UUGUGAGAGAGAGGG<br>miRNA 3' G A U 5'	-33.8	387	DB946667	similar to 30S ribosomal protein S8 [Ricinus communis, EEF33949.1]	structural molecule activity
	target 5' U C G U 3'<br>UUCUUUC AACACUUUCUC CCU	-32.6	256	DB924110	similar to catalytic [Ricinus communis, EEF51611.1]	binding

	AAGGAAG UUGUGAGAGAG GGG<br>miRNA 3' G U A 5'					
	target 5' U G A 3'<br>CUUCCUUCAAACGU UUCUCUU<br>GAAGGAAGUUUGUG GAGAGAG<br>miRNA 3' A GG 5'	-30.7	450	DB927765	similar to breast carcinoma amplified sequence [Ricinus communis, EEF49767.1]	
**ACMV-mir-3-17***	target 5' G U A U 3'<br>CUUUCCUUC AA ACUCUCUCUCU<br>GGAAGGAAG UU UGAGAGAGAGG<br>miRNA 3' U G 5'	-35.4	120	FF381792	similar to 50S ribosomal protein L21 [Ricinus communis, EEF3555]	structural molecule activity
	target 5' C G 3'<br>UUCUUCA ACGCUCUCUCUCU<br>AGGAAGU UGUGAGAGAGAGG<br>miRNA 3' GGA U 5'	-35.2	51	FF381792	similar to 50S ribosomal protein L21 [Ricinus communis, EEF3555]	structural molecule activity
	target 5' U C A U 3'<br>UUUCUUUC AG ACUCUCUCUCU<br>GAAGGAAG UU UGAGAGAGAGG<br>miRNA 3' G U G 5'	-31.1	68	DB932262	similar to sodium/hydrogen exchanger [Ricinus communis, EEF49734.1]	transporter activity
	target 5' U C U 3'<br>UUUCCU C AACAUUCUUUCUCC<br>GAAGGA G UUGUGAGAGAGAGG<br>miRNA 3' G A U 5'	-32.8	386	DB946667	similar to 30S ribosomal protein S8 [Ricinus communis, EEF33949.1]	structural molecule activity
	target 5' U G A 3'<br>UUUUU UCAAACACUCUUUUUC<br>GAAGG AGUUUGUGAGAGAGAG<br>miRNA 3' G A G 5'	-29.9	474	DB950915	similar to uncharacterized protein Glycine max, XP_003556451.1]	
	target 5' A G A 3'<br>UCUUCCUUCAAACGU UUCUCUU<br>GGAAGGAAGUUUGUG GAGAGAG<br>miRNA 3' A G 5'	-32.4	449	DB927765	similar to breast carcinoma amplified sequence [Ricinus communis, EEF49767.1]	

	target 5' C C C A 3'<br>UCUUCCU C AAU UUCUCUCUCC<br>GGAAGGA G UUG GAGAGAGAGG<br>miRNA 3' A U U 5'	-30.7	126	DB928865	similar to electron transporter [Ricinus communis, EEF49572.1]	electron carrier activity
	target 5' C U A A 3'<br>UCUUUCUUCAA CGCUCUCUU CU<br>GGAAGGAAGUU GUGAGAGAG GG<br>miRNA 3' U A 5'	-32.7	551	DB928865	similar to electron transporter [Ricinus communis, EEF49572.1]	electron carrier activity
	target 5' U G A 3'<br>CUUUUCUUCAAGUACUUUUUU UC<br>GGAAGGAAGUUUGUGAGAGAG GG<br>miRNA 3' A 5'	-26.1	346	DB940623	similar to 60S ribosomal protein L22 [Ricinus communis, EEF39275.1]	structural molecule activity
	target 5' C G G 3'<br>CUUUCUUUUAAAUGCUCUC UUC<br>GGAAGGAAGUUUGUGAGAG GAG<br>miRNA 3' A G 5'	-30.2	296	DB929517	similar to ERD1 protein, chloroplast [Ricinus communis, EEF51657.1]	binding
**ACMV-mir-3-18***	target 5' G U A C 3'<br>CUUUCCUUC AA ACUCUCUCU<br>GGAAGGAAG UU UGAGAGAGA<br>miRNA 3' A U G 5'	-32	120	DB924367	similar to 50S ribosomal protein L21 [Ricinus communis, EEF3555]	structural molecule activity
	target 5' U C C 3'<br>UCUU UUCUUCA ACGCUCUCUCU<br>AGGA AGGAAGU UGUGAGAGAGA<br>miRNA 3' U 5'	-31.6	46	DB924367	similar to 50S ribosomal protein L21 [Ricinus communis, EEF3555]	structural molecule activity
	target 5' A G U 3'<br>UCUUCCUUCAAACGU UUCUCU<br>GGAAGGAAGUUUGUG GAGAGA<br>miRNA 3' A A 5'	-30.9	449	DB927765	similar to breast carcinoma amplified sequence [Ricinus communis, EEF49767.1]	
	target 5' U U C 3'<br>UUUUUUUUUU AACACUCUCUC	-28.3	577	DV457189	similar to 2-deoxyglucose-6-phosphate phosphatase [Ricinus communis, EEF49975.1]	binding

	AGGAAGGAAG UUGUGAGAGAG<br>miRNA 3' U A 5'					
	target 5' U A U G 3'<br>UCCUUCCUU GAGC CUCUCUUU<br>AGGAAGGAA UUUG GAGAGAGA<br>miRNA 3' G U 5'	-33.6	242	DB933884	similar to diphosphomevalonate decarboxylase [Ricinus communis, EEF41203.1]	binding
	target 5' U C C G 3'<br>UCCUUCU UC GACGCUCUCUCU<br>AGGAAGG AG UUGUGAGAGAGA<br>miRNA 3' A U 5'	-36.7	144	XDB929515	similar to disulfide oxidoreductase [Ricinus communis, EEF32882.1]	binding
	target 5' U G 3'<br>UCUUUUCUUCAAGUACUUUUUU<br>AGGAAGGAAGUUUGUGAGAGAG<br>miRNA 3' A 5'	-28.1	345	DB940623	similar to 60S ribosomal protein L22 [Ricinus communis, EEF39275.1]	structural molecule activity
**ACMV-mir-3-19***	target 5' C U U 3'<br>UCUUUCUUCAA CGCUCUCU<br>GGAAGGAAGUU GUGAGAGA<br>miRNA 3' CA U 5'	-30.6	551	DB928865	similar to electron transporter [Ricinus communis, EEF49572.1]	electron carrier activity
	target 5' A A 3'<br>CCUUCCUUUGAAUAUUCUC<br>GGAAGGAAGUUUGUGAGAG<br>miRNA 3' CA A 5'	-33	337	CK641680	similar to short-chain dehydrogenase [Ricinus communis, EEF512204]	binding
	target 5' U U 3'<br>UCUUUUCUUCAAGUACUUUUU<br>AGGAAGGAAGUUUGUGAGAGA<br>miRNA 3' C 5'	-26.4	345	DB940623	similar to 60S ribosomal protein L22 [Ricinus communis, EEF39275.1]	structural molecule activity
	target 5' U U C 3'<br>UUUUUUUUUU AACACUCUCU<br>AGGAAGGAAG UUGUGAGAGA<br>miRNA 3' C U 5'	-25.5	284	FG807027	similar to 2-deoxyglucose-6-phosphate phosphatase [Ricinus communis, EEF49975.1]	binding

	target 5' U G G U 3'<br>GUUU UCCUU AAACACUCUUU<br>CAGG AGGAA UUUGUGAGAGA<br>miRNA 3' A G 5'	-28.3	370	DB949918	similar to uncharacterized protein LOC100789970 [Glycine max, XP_003517375.1]	
	target 5' U A 3'<br>UUUUUUCUUCAAAUAUUUUC<br>AGGAAGGAAGUUUGUGAGAG<br>miRNA 3' C A 5'	-26.7	378	DV445050	similar to nucleic acid binding protein [Ricinus communis, EEF35632.1]	binding
	target 5' U A A 3'<br>UUCUUUCUUCAAACA UUUC<br>AGGAAGGAAGUUUGU AGAG<br>miRNA 3' C G A 5'	-27.4	482	DB934327	similar to kinase [Ricinus communis, EEF37837.1]	binding
	target 5' U U A 3'<br>GUCCUUCUUUCAAGC CUCUU<br>CAGGAAGGAAGUUUG GAGAG<br>miRNA 3' U A 5'	-33.9	289	DB926022	similar to Omega-3 fatty acid desaturase, chloroplast [Ricinus communis, P48619.1]	catalytic activity
**ACMV-mir-4-1**	target 5' G A C U 3'<br>GGUAUAUAC CU AGCUCAUGUAUU<br>UCGUAUGUG GA UCGGGUACAUGG<br>miRNA 3' U 5'	-32.8	128	FG805909	similar to big map kinase/bmk [Ricinus communis, EEF36363.1]	binding
**ACMV-mir-4-2**	target 5' G G G U 3'<br>GCGAG AUGCACCUAAG CCGUGU<br>CGUUC UAUGUGGAUUC GGUACA<br>miRNA 3' G G 5'	-40	290	DB929628	similar to actin related protein [Populus trichocarpa, EEE96159.1]	
**ACMV-mir-4-3**	target 5' C A U G G 3'<br>UG AAGC UACAUU AAGCCCAUG<br>AC UUCG AUGUGG UUCGGGUAC<br>miRNA 3' G U A 5'	-31.8	170	DB928320	similar to R2R3 MYB transcription factor [Jatropha curcas]	binding
	target 5' G G G U 3'	-40	290	DB929628	similar to actin related protein [Populus trichocarpa, EEE96159.1]	

	GCGAG AUGCACCUAAG CCGUG<br>CGUUC UAUGUGGAUUC GGUAC<br>miRNA 3' A G G 5'					
	target 5' U G C 3'<br>CAGGCAUAUGCU AAGUUUAUG<br>GUUCGUAUGUGG UUCGGGUAC<br>miRNA 3' AC A 5'	-28.3	466	DB953621	similar to map3k delta-1 protein kinase [Ricinus communis, EEF31491.1]	binding
	target 5' C A G A 3'<br>UGCAAGUAU GCA CUAAGCCCA<br>ACGUUCGUA UGU GAUUCGGGU<br>miRNA 3' G AC 5'	-37.7	460	DB944600	similar to glutamine synthetase I [Medicago truncatula, CAB63844.1]	binding
	target 5' C U G C 3'<br>UGUA GUAUAUAUU AAGUCUAUG<br>ACGU CGUAUGUGG UUCGGGUAC<br>miRNA 3' U A 5'	-26.9	770	DV457971	similar to heme-binding protein [Ricinus communis, EEF51963.1]	electron carrier activity
**ACMV-mir-4-4**	target 5' C G G A 3'<br>UGCAAGUAUA CA CUAAGCCCA<br>ACGUUCGUAU GU GAUUCGGGU<br>miRNA 3' A G 5'	-38.2	460	DB944600	similar to glutamine synthetase I [Medicago truncatula, CAB63844.1]	binding
	target 5' A A C 3'<br>UGUGAGUAUACGCCUGAG UCA<br>ACGUUCGUAUGUGGAUUC GGU<br>miRNA 3' A G 5'	-33.6	251	DB944352	similar to ATP binding protein [Ricinus communis, EEF40790.1]	binding
**ACMV-mir-4-5**	target 5' G C A 3'<br>UUGCAAGCAUGUAUCU AGC<br>AACGUUCGUAUGUGGA UCG<br>miRNA 3' U 5'	-29.5	285	DB928841	similar to 60S ribosomal protein L44 [Ricinus communis, EEF46746.1]	structural molecule activty
**ACMV-mir-4-6***	target 5' U U C U 3'<br>UA GGU UUGGGCUUGUGUGU<br>GU UCA GACCCGAGCAUACA	-29.6	31	DB934920	similar to heat shock factor protein HSF24-like [Vitis vinifera, CBI21429.3]	nucleic acid binding transcription factor activity

	miRNA 3' G U U U 5'					
**ACMV-mir-4-7***	target 5' U U U 3'<br>UCA GGUGUUGGGCUUGUGU<br>GGU UCAUGACCCGAGCAUA<br>miRNA 3' U 5'	-28.3	563	FF536420	similar to 60S ribosomal protein L21 [Ricinus communis, EEF31216.1]	structural molecule activty
**ACMV-mir-5-1**	target 5' G G 3'<br>CUGGAUGAGGAGAAGCAAGA<br>GAUCUGCUCCUUUUCGUUCU<br>miRNA 3' AAG 5'	-38.7	510	CK647841	similar to casein kinase [Ricinus communis, EEF48084.1]	binding
**ACMV-mir-5-2**	target 5' A G U C 3'<br>UC UGGGCGAGGA GAAGCAGGG<br>AG AUCUGCUCCU UUUCGUUCU<br>miRNA 3' CA G 5'	-35.5	262	DB932284	similar to sucrose synthase [Ricinus communis, EEF39300.1]	catalytic activity
	target 5' A U C 3'<br>UUCUGG GUGGGGAAGAGUAG<br>AGGAUC UGCUCCUUUUCGUU<br>miRNA 3' CA CU 5'	-25.8	240	DB927558	similar to protein phosphatase 2c [Ricinus communis, EEF42042.1]	binding
**ACMV-mir-5-3**	target 5' U G C 3'<br>UUUU AGAUGAGG GAAGCAGG<br>AAGG UCUGCUCC UUUCGUUC<br>miRNA 3' UC A U 5'	-27.4	157	DB949212	similar to conserved hypothetical protein [Ricinus communis, EEF29221.1]	
**ACMV-mir-5-4**	target 5' C U U 3'<br>AGUUCCU GAUGAGGAAAAGU<br>UCAAGGA CUGCUCCUUUUCG<br>miRNA 3' C U UU 5'	-35.3	253	DB924672	similar to WD-repeat protein [Ricinus communis, EEF44865.1]	
	target 5' C C G U 3'<br>GAGUUUCU GA GA GAAGGGUAA<br>CUCAAGGA CU CU CUUUUCGUU<br>miRNA 3' U G C 5'	-27.3	254	DB926979	similar to cytochrome P450 protein CYP71E [Manihot esculenta, AAP57704.1]	electron carrier activity

	target 5' A C U A 3'<br>AGUUUCU GAUGA GGAAAGUGA<br>UCAAGGA CUGCU CUUUUCGUU<br>miRNA 3' C U C 5'	-26.9	209	DB944307	similar to long-chain-fatty-acid CoA ligase [Ricinus communis, EEF48390.1]	catalytic activity
	target 5' C U G A C 3'<br>GA UU C AGGUGAGGAGAAGCAA<br>CU AA G UCUGCUCCUUUUCGUU<br>miRNA 3' C G A 5'	-27.5	381	DB934040	similar to FGGY carbohydrate kinase domain-containing protein [Vitis vinifera, CBI17791.3]	catalytic activity
**ACMV-mir-5-5**	target 5' A C A 3'<br>AGAG UCCUAGAUGGGGAAGAG<br>UCUC AGGAUCUGCUCCUUUUC<br>miRNA 3' A 5'	-37.5	423	DB936323	similar to phopholipase d alpha [Ricinus communis, EEF44789.1]	binding
	target 5' C C A 3'<br>AGUUCUU GACGAGGAAGA<br>UCAAGGA CUGCUCCUUUU<br>miRNA 3' UC U C 5'	-30.5	82	FF381793	similar to thioredoxin h-type [Ricinus communis, EEF42213.1]	electron carrier activity
	target 5' U G C G 3'<br>GGAGUUCCUAGA GGGGA AAG<br>UCUCAAGGAUCU CUCCU UUC<br>miRNA 3' G U 5'	-36.4	270	DR083912	similar to RNA and export factor-binding protein 2 [Vitis vinifera, CBI16171.3]	binding
	target 5' U U 3'<br>GAGUUCCUGGAUGAGGAAGG<br>CUCAAGGAUCUGCUCCUUUU<br>miRNA 3' U C 5'	-37.8	544	DV454057	similar to vacuolar cation/proton exchanger 1a, [Ricinus communis, EEF34279.1]	transporter activity
	target 5' A G A 3'<br>GGGGUUUU GGAUGAGGAAAAG<br>UCUCAAGG UCUGCUCCUUUUC<br>miRNA 3' A 5'	-32.6	293	CK649231	similar to AP2/ERF domain-containing transcription factor [Populus trichocarpa, EEE71425.1]	nucleic acid binding transcription factor activity

	target 5' A C C A 3'<br>AGAG UUCU GACGAGGAAGA<br>UCUC AGGA CUGCUCCUUUU<br>miRNA 3' A U C 5'	-30.5	420	DB934347	similar to SIT4 phosphatase-associated family protein [Arabidopsis lyrata subsp. Lyrata, EFH65936.1]	
**ACMV-mir-5-6**	target 5' G C A 3'<br>AAGAG UCCUAGAUGGGGAAGAG<br>UUCUC AGGAUCUGCUCCUUUUC<br>miRNA 3' A 5'	-38.5	422	DB936323	similar to phopholipase d alpha [Ricinus communis, EEF44789.1]	binding
	target 5' U G C G 3'<br>GGAGUUCCUAGA GGGGA AAG<br>UCUCAAGGAUCU CUCCU UUC<br>miRNA 3' U G U 5'	-36.5	302	DR088294	similar to RNA and export factor binding protein [Ricinus communis, EEF31023.1]	binding
	target 5' U U 3'<br>GAGUUCCUGGAUGAGGAAGG<br>CUCAAGGAUCUGCUCCUUUU<br>miRNA 3' UU C 5'	-37.8	544	DV454057	similar to vacuolar cation/proton exchanger 1a [Ricinus communis, EEF34279.1]	transporter activity
	target 5' C G U A 3'<br>AGGAGUU CUA GACGAGGGAAAG<br>UUCUCAA GAU CUGCUCCUUUUC<br>miRNA 3' G 5'	-35.9	219	CK644558	similar to nucleotide sugar epimerase [Cucumis sativus]	catalytic activity
	target 5' A G A 3'<br>AGGGGUUUU GGAUGAGGAAAAG<br>UUCUCAAGG UCUGCUCCUUUUC<br>miRNA 3' A 5'	-33.2	292	CK649128	similar to AP2/ERF domain-containing transcription factor [Populus trichocarpa, EEE71425.1]	nucleic acid binding transcription factor activity
	target 5' G C C A 3'<br>AAGAG UUCU GACGAGGAAGA<br>UUCUC AGGA CUGCUCCUUUU<br>miRNA 3' A U C 5'	-31.5	419	DB934347	similar to SIT4 phosphatase-associated family protein [Arabidopsis lyrata subsp. Lyrata, EFH65936.1]	

**ACMV-mir-5-7***	target 5' A U C C 3'<br>CCUUUGC GUCCAG AUUUGCCUU<br>GGAGACG UAGGUC UGGAUGGAG<br>miRNA 3' U C 5'	-35.2	310	CK643355	similar to pyruvate decarboxylase [Ricinus communis, EEF39845.1]	binding
	target 5' U G C U 3'<br>CCUCUGCAA CC AG GCUUGCUUC<br>GGAGACGUU GG UC UGGAUGGAG<br>miRNA 3' A C 5'	-34.5	457	DB942972	similar to ribosomal protein L15 [Ricinus communis, EEF51579.1]	structural molecule activity
	target 5' A U A U 3'<br>CCU UGCA UC UAGGACUUGCUUC<br>GGA ACGU AG GUCCUGGAUGGAG<br>miRNA 3' G U 5'	-33.3	3	FF534218	similar to zinc finger protein [Ricinus communis, EEF35350.1]	binding
**ACMV-mir-5-8***	target 5' U A 3'<br>UUCUUUUGCAAUCUGGGGCUUA<br>AAGGAGACGUUAGGUCCUGGAU<br>miRNA 3' G 5'	-36.3	454	DV446664	similar to 60S ribosomal protein L13aA [Hevea brasiliensis, ADR71263.1]	catalytic activity
	target 5' A U A 3'<br>UCUUCUGCGGUUCAGG UCUAU<br>AGGAGACGUUAGGUCC GGAUG<br>miRNA 3' A U 5'	-35.7	513	CK643668	similar to pyrophosphate-energized vacuolar membrane proton pump [Ricinus communis, EEF31615.1]	catalytic activity
**ACMV-mir-5-9***	target 5' A A A 3'<br>UCUU CUCUG AAUCCAGGACC<br>AGAA GAGAC UUAGGUCCUGG<br>miRNA 3' G G A 5'	-36.9	191	DB935464	similar to calcium-activated outward-rectifying potassium channel [Ricinus communis, EEF47173.1]	binding
	target 5' A A 3'<br>UUUCUUUUGCAAUCUGGGGCUU<br>GAAGGAGACGUUAGGUCCUGGA<br>miRNA 3' A 5'	-36.2	453	DV446664	similar to 60S ribosomal protein L13aA [Hevea brasiliensis, ADR71263.1]	structural molecule activity

	target 5' G C C 3'<br>UC UCCUCUGC GUCCAGGACUU<br>AG AGGAGACG UAGGUCCUGGA<br>miRNA 3' A U 5'	-38.9	378	DB950958	similar to U2 snrnp auxiliary factor, small subunit [Ricinus communis, EEF33789.1]	binding
	target 5' C G G 3'<br>UCUUC UUCUGCAAUUCAGGAUU<br>AGAAG GAGACGUUAGGUCCUGG<br>miRNA 3' A 5'	-36.5	226	CK652240	similar to receptor protein kinase [Ricinus communis, EEF50538.1]	binding
**ACMV-mir-5-10***	target 5' A A A 3'<br>AUCUU CUCUG AAUCCAGGACC<br>UAGAA GAGAC UUAGGUCCUGG<br>miRNA 3' G G 5'	-37.8	190	DB935464	similar to calcium-activated outward-rectifying potassium channel [Ricinus communis, EEF47173.1]	binding
	target 5' A U 3'<br>UUUCUUUUGCAAUCUGGGGCU<br>GAAGGAGACGUUAGGUCCUGG<br>miRNA 3' UA 5'	-35	453	DV446664	similar to 60S ribosomal protein L13aA [Hevea brasiliensis, ADR71263.1]	structural molecule activity
	target 5' C G G 3'<br>UCUUC UUCUGCAAUUCAGGAUU<br>AGAAG GAGACGUUAGGUCCUGG<br>miRNA 3' U 5'	-36.8	226	CK652240	similar to receptor protein kinase [Ricinus communis, EEF50538.1]	binding
	target 5' C A A 3'<br>AUUUUCUUUUGUGAUUCA GAC<br>UAGAAGGAGACGUUAGGU CUG<br>miRNA 3' C G 5'	-28	323	DB932653	similar to delta-12-fatty acid desaturase [Jatropha curcas, ADB93805.1]	catalytic activity
**ACMV-mir-5-11***	target 5' U C G 3'<br>CACU UCUUCCUCUGCAAUCU<br>GUGA AGAAGGAGACGUUAGG<br>miRNA 3' U UC 5'	-37.5	23	DV454708	similar to reticulon-3 [Ricinus communis, EEF43605.1]	
	target 5' G C A U 3'	-29.4	368	FF534315	similar to photosystem I P700 apoprotein A1 [Oryza australiensis, ADD62970.1]	binding

	ACU UCUUCUU UGCAGUCCA<br>UGA AGAAGGA ACGUUAGGU<br>miRNA 3' G U G C 5'					
	target 5' G A U 3'<br>CACUGUUU CC UCUGCGAUCUA<br>GUGAUAGA GG AGACGUUAGGU<br>miRNA 3' A C 5'	-28.2	110	DB937239	similar to zinc finger CCCH domain-containing protein 6 [Vitis vinifera, XP_002277913.1]	binding
**ACMV-mir-5-12***	target 5' U C G 3'<br>CUCACU UCUUCCUCUGCAAUCU<br>GGGUGA AGAAGGAGACGUUAGG<br>miRNA 3' U U 5'	-41.1	21	DB921202	similar to reticulon-3 [Ricinus communis, EEF43605.1]	
	target 5' U U U C 3'<br>CUC CUGUCUUCUUUU CAAUCC<br>GGG GAUAGAAGGAGA GUUAGG<br>miRNA 3' U C U 5'	-29.7	6	DB929039	similar to hydroxycinnamoyl-Coenzyme A shikimate/quinate hydroxycinnamoyltransferase [Vitis vinifera]	catalytic activity
	target 5' U U C C 3'<br>CUCAUU UCUUUCUCU CAAUUC<br>GGGUGA AGAAGGAGA GUUAGG<br>miRNA 3' U C U 5'	-27.4	260	DB928965	similar to RNA binding protein [Ricinus communis, EEF29218.1]	binding
**ACMV-mir-5-13***	target 5' A C G 3'<br>UCUCACU UCUUCCUCUGCAAUCU<br>AGGGUGA AGAAGGAGACGUUAGG<br>miRNA 3' U 5'	-43.5	103	DB921202	similar to reticulon-3 [Ricinus communis, EEF43605.1]	
	target 5' U U C C 3'<br>UCUCAUU UCUUUCUCU CAAUUC<br>AGGGUGA AGAAGGAGA GUUAGG<br>miRNA 3' U C 5'	29.9	207	DV458190	similar to RNA binding protein [Ricinus communis, EEF29218.1]	
	target 5' C U U C 3'<br>UCUC CUGUCUUCUUUU CAAUCC	-32.1	5	DB929039	similar to hydroxycinnamoyl-Coenzyme A shikimate/quinate hydroxycinnamoyltransferase [Vitis vinifera]	catalytic activity

	AGGG GAUAGAAGGAGA GUUAGG<br>miRNA 3' U C 5'					
	target 5' G G U C 3'<br>UCUUAUUGUCUU CUCUGUA UCU<br>AGGGUGAUAGAA GAGACGU AGG<br>miRNA 3' G U 5'	-32.3	138	GH612006	similar to calmodulin binding protein [Ricinus communis, EEF48083.1]	binding
**ACMV-mir-5-14***	target 5' A C U 3'<br>UCUCACU UCUUCCUCUGCAAUC<br>AGGGUGA AGAAGGAGACGUUAG<br>miRNA 3' U 5'	-41.9	103	DB921202	similar to reticulon-3 [Ricinus communis, EEF43605.1]	
	target 5' U U C C 3'<br>UCUCAUU UCUUUCUCU CAAUU<br>AGGGUGA AGAAGGAGA GUUAG<br>miRNA 3' U C 5'	-27.7	259	DB928965	similar to RNA binding protein [Ricinus communis, EEF29218.1]	binding
	target 5' C U U C 3'<br>UCUC CUGUCUUCUUUU CAAUC<br>AGGG GAUAGAAGGAGA GUUAG<br>miRNA 3' U C 5'	-28.8	5	DB929039	similar to hydroxycinnamoyl-Coenzyme A shikimate/quinate hydroxycinnamoyltransferase [Vitis vinifera]	catalytic activity
	target 5' U U C 3'<br>UCCC CUAUCUUUCUCUGUGA<br>AGGG GAUAGAAGGAGACGUU<br>miRNA 3' U AG 5'	-34.5	18	DB923718	similar to scarecrow-like protein 3-like [Vitis vinifera, CAN60488.1]	nucleic acid binding transcription factor activity
**ACMV-mir-5-15***	target 5' A C U 3'<br>UCUCACU UCUUCCUCUGCAAUC<br>AGGGUGA AGAAGGAGACGUUAG<br>miRNA 3' A U 5'	-42.7	103	DB921202	similar to reticulon-3 [Ricinus communis, EEF43605.1]	
	target 5' C U C C 3'<br>UUCUCAUU UCUUUCUCU CAAUU<br>AAGGGUGA AGAAGGAGA GUUAG	-28.5	206	DV458190	similar to RNA binding protein [Ricinus communis, EEF29218.1]	binding

	miRNA 3' U C 5'					
	target 5' U U C 3'<br>UUCCC CUAUCUUUCUCUGUGA<br>AAGGG GAUAGAAGGAGACGUU<br>miRNA 3' U AG 5'	-35.4	17	DB923718	similar to scarecrow-like protein 3-like [Vitis vinifera, CAN60488.1]	nucleic acid binding transcription factor activity
**ACMV-mir-6-1**	target 5' U C A 3'<br>GAAGGUCUUGUG UGCUGCUU<br>UUUCCAGAGUAU ACGACGGA<br>miRNA 3' A 5'	-32.7	40	FG805430	similar to heat shock protein hsp82 [Oryza sativa, CAA78738.1]	binding
	target 5' U A U U 3'<br>AAG GUUUUAU UUGCUGUCU<br>UUC CAGAGUA AACGACGGA<br>miRNA 3' U U 5'	-25.3	214	DB937274	similar to vesicle-associated membrane protein [Ricinus communis, EEF30603.1]	structural molecule activity
**ACMV-mir-6-2**	target 5' U U A A 3'<br>UUUAGAG UU CAUAUUGCUGC<br>AGGUUUC AG GUAUAACGACG<br>miRNA 3' C C A G 5'	-27.9	743	FF535705	similar to 40S ribosomal protein S9 [Ricinus communis, EEF52159.1]	structural molecule activity
	target 5' C U U U 3'<br>CCAA G UCUCAUGUUGUUGUU<br>GGUU C AGAGUAUAACGACGG<br>miRNA 3' CA U C 5'	-29.4	129	CK652363	similar to mitogen activated protein kinase kinase, mapkk2 [Ricinus communis, EEF48158.1]	binding
	target 5' C G 3'<br>UUCAAGGGUUUUAUGUUGUU UC<br>AGGUUUCCAGAGUAUAACGA GG<br>miRNA 3' C C 5'	-29.1	118	DV441358	similar to rer1 protein [Ricinus communis, EEF40831.1]	
**ACMV-mir-6-3**	target 5' G A A U 3'<br>GGUCCAA AGG CU AUAUUGCU<br>UCAGGUU UCC GA UAUAACGA	-28.7	314	DV442611	similar to thylakoid lumenal 16.5 kDa protein, chloroplast precursor [Ricinus communis, EEF35210.1]	

	miRNA 3' GA A G 5'					
	target 5' U C U 3'<br>GUUC AAGGUUUUAUGUUGUU<br>CAGG UUCCAGAGUAUAACGA<br>miRNA 3' GAU U 5'	-27.5	21	DV440887	similar to nutrient reservoir [Ricinus communis, EEF31271.1]	nutrient reservoir activity
	target 5' A G G 3'<br>AGUCCAAAGG CUU AUGUUGUU<br>UCAGGUUUCC GAG UAUAACGA<br>miRNA 3' GA A 5'	-30.8	157	DV444080	similar to ubiquitin-activating enzyme E1b [Ricinus communis, EEF49457.1]	binding
	target 5' A C C U 3'<br>AG UUCGGAGGUUUCAU UUGCU<br>UC AGGUUUCCAGAGUA AACGA<br>miRNA 3' GA U 5'	-29.5	242	DV446004	similar to protein binding protein [Ricinus communis, EEF34752.1]	
	target 5' U A 3'<br>CUGGUCU GGGGUCUU UGUUGUU<br>GAUCAGG UUCCAGAG AUAACGA<br>miRNA 3' U U 5'	-27.8	528	DB938159	similar to tubulin alpha chain [Ricinus communis, EEF30323.1]	binding
**ACMV-mir-6-4**	target 5' A A U 3'<br>UGG CCUGGUC GAAGGUUUC<br>ACC GGAUCAG UUUCCAGAG<br>miRNA 3' U G UA 5'	-32.6	57	FF535000	similar to phytanoyl-CoA dioxygenase domain containing [Ricinus communis, EEF36371.1]	catalytic activity
	target 5' C U 3'<br>GGACCUGGU CAAAGGUCUC<br>CCUGGAUCA GUUUCCAGAG<br>miRNA 3' A G UA 5'	-38.8	409, 636	FF536517	similar to ecotropic viral integration site [Ricinus communis, EEF36381.1]	enzyme regulator activity
	target 5' U U C 3'<br>ACUU GUUCAGGGGUCUUGU<br>UGGA CAGGUUUCCAGAGUA<br>miRNA 3' ACC U 5'	-26.9	117	DB944692	similar to copper transporter [Ricinus communis, EEF46157.1]	transporter activity

**ACMV-mir-6-5**	target 5' G A U G 3'<br>UG AUGCC GG AUCUGGUCCAAGG<br>AC UGUGG CC UGGAUCAGGUUUC<br>miRNA 3' C A 5'	-31.6	543	DB951256	similar to acetylglucosaminyltransferase [Ricinus communis, EEF35190.1]	catalytic activity
	target 5' A A C 3'<br>GACA CUGGGUUUGGUUCGAA<br>CUGU GACCUGGAUCAGGUUU<br>miRNA 3' AC G C 5'	-27.7	465	DB931371	similar to 30S ribosomal protein S5 [Ricinus communis, EEF50844.1]	structural molecule activity
	target 5' A C U C 3'<br>UGGGUACU GGACUU GUUUAGAG<br>ACCUGUGG CCUGGA CAGGUUUC<br>miRNA 3' A U 5'	-29	397	FF535408	similar to pentatricopeptide repeat-containing protein [Ricinus communis, EEF29801.1]	binding
**ACMV-mir-6-6**	target 5' C U A C 3'<br>AUGUGGGU C UGGACCUGGUU<br>UACACCUG G ACCUGGAUCAG<br>miRNA 3' GA U G 5'	-31.9	308	CK647559	similar to conserved hypothetical protein [Ricinus communis, EEF42715.1]	
**ACMV-mir-6-7***	target 5' C G U 3'<br>AGA CUUUGGGCUUACACGA<br>UCU GAAAUCCGGGUGUGUU<br>miRNA 3' A 5'	-30.8	358	DB951561	similar to ATP-dependent clp protease [Ricinus communis, EEF51704.1]	binding
	target 5' A A A A 3'<br>AGAUC U AGGCUCAUAUAG<br>UCUAG A UCCGGGUGUGUU<br>miRNA 3' A A 5'	-26.2	13	DB948941	similar to queuine tRNA-ribosyltransferase [Ricinus communis, EEF35976.1]	binding
	target 5' G A A 3'<br>GGAUCUUUGG UCUAUACAG<br>UCUAGAAAUC GGGUGUGUU<br>miRNA 3' C 5'	-27	262	DV445604	similar to ubiquitin-protein ligase [Ricinus communis, EEF36752.1]	catalytic activity
	target 5' U C G G 3'<br>AGG CUU AGGCUCAUACAG	-26.6	343	DB922543	similar to proteasome maturation protein [Ricinus communis, EEF36388.1]	

	UCU GAA UCCGGGUGUGUU<br>miRNA 3' A A 5'					
**ACMV-mir-6-8***	target 5' G A G C 3'<br>GG GCCAGA CUUUGGGCUUACA<br>CC CGGUCU GAAAUCCGGGUGU<br>miRNA 3' UA A 5'	-38.8	356	DB944372	similar to ATP-dependent clp protease [Ricinus communis, EEF51704.1]	binding
**ACMV-mir-6-9***	target 5' G C C C 3'<br>GACG UAU GG UAGAUCUUUAGG<br>CUGC AUA CC GUCUAGAAAUCC<br>miRNA 3' U C G 5'	-29.2	441	DB951887	similar to nucleic acid binding protein [Ricinus communis, EEF48069.1]	binding
	target 5' A C U U 3'<br>GA AUGGGUCGGAUUUUUG GG<br>CU UACCCGGUCUAGAAAU CC<br>miRNA 3' CUG A 5'	-30.3	22	DV450217	similar to protein yippee-like At5g53940-like [Glycine max, XP_003543228.1]	binding
**ACMV-mir-6-10***	target 5' A C U 3'<br>AGGA GA AUGGGUCGGAUUUU<br>UUCU CU UACCCGGUCUAGAA<br>miRNA 3' CC G A 5'	-28.2	103	DV448195	similar to protein yippee-like [Glycine max, XP_003543228.1]	binding
**ACMV-mir-6-11***	target 5' U C 3'<br>GGACGAUAUGGGUUGGAUU<br>UCUGCUAUACCCGGUCUAG<br>miRNA 3' CU A 5'	-34	662	DV443483	similar to chaperonin containing t-complex protein 1, alpha subunit, tcpa, [Ricinus communis, EEF37754.1]	binding
	target 5' A C U 3'<br>AGGA GA AUGGGUCGGAUUU<br>UUCU CU UACCCGGUCUAGA<br>miRNA 3' C G A 5'	-27.3	103	DV448195	similar to protein yippee-like At5g53940-like [Glycine max, XP_003543228.1]	binding
**ACMV-mir-6-12***	target 5' A G U U 3'<br>GGA GAUGUG GCCGGAUC<br>UCU CUAUAC CGGUCUAG<br>miRNA 3' G C 5'	-30.7	124	DB923220	similar to ssm4 protein [Ricinus communis, EEF43654.1]	binding

	target 5' U G A G 3'<br>GGACGAUG GG GCUAGAU<br>UCUGCUAU CC CGGUCUA<br>miRNA 3' A G 5'	-28.7	162	DB932352	similar to DNA binding protein [Ricinus communis, EEF28231.1]	binding
	target 5' U G A 3'<br>GGAUGGU AU GGCCAGAUC<br>UCUGCUA UA CCGGUCUAG<br>miRNA 3' C 5'	-29.1	492	FG805208	similar to calmodulin-related protein isoform 4 [Vitis vinifera, XP_003632230.1]	binding
	target 5' U G 3'<br>GGAUGG GUGGGUCGGGUU<br>UCUGCU UACCCGGUCUAG<br>miRNA 3' A 5'	-28.2	784	FF536606	similar to cytochrome P450 [Ricinus communis, EEF36726.1]	electron carrier activity
**ACMV-mir-6-13***	target 5' U U A 3'<br>GGGAAGAU AUAUGGGCCA<br>CCCUUCUG UAUACCCGGU<br>miRNA 3' U C CU 5'	-37.2	246	CK643935	similar to coatomer beta subunit [Ricinus communis]	structural molecule activity
	target 5' C U U C 3'<br>AGGG GGA CGG GUGGGUUGG<br>UCCC UCU GCU UACCCGGUC<br>miRNA 3' U A U 5'	-26.8	560	DB933975	similar to photosystem I reaction center subunit III [Ricinus communis, EEF45974.1]	
**ACMV-mir-6-14***	target 5' U U A 3'<br>GGGAAGAU AUAUGGGCCA<br>CCCUUCUG UAUACCCGGU<br>miRNA 3' GU C 5'	-37.1	246	CK643935	similar to coatomer beta subunit [Ricinus communis]	structural molecule activity
	target 5' G U A 3'<br>UAGG GAG C AUAUGGGCUA<br>GUCC UUC G UAUACCCGGU<br>miRNA 3' C U C 5'	-26.3	507	DB955011	similar to amino acid transporter [Ricinus communis, EEF31031.1]	transporter activity
**ACMV-mir-6-15***	target 5' C U A 3'	-30.5	75	DB938056	similar to poly-A binding protein [Ricinus communis, EEF30719.1]	binding

	GGACA GGAAGGUGAUAUGG<br>CUUGU CCUUCUGCUAUACC<br>miRNA 3' GU C CG 5'					
**ACMV-mir-6-16***	target 5' A U C U 3'<br>GGCAGAACA GGG AGGCGAUAUG<br>UCGUCUUGU CCC UCUGCUAUAC<br>miRNA 3' U C 5'	-38.8	475	DB929843	similar to heterogeneous nuclear ribonucleoprotein [Ricinus communis, EEF48904.1]	binding
	target 5' U C U A 3'<br>GCAGAAUGG GAAG UGAUGUG<br>CGUCUUGUC CUUC GCUAUAC<br>miRNA 3' U C U C 5'	-30	599	DV458828	similar to protein phosphatase 2c [Ricinus communis, EEF42042.1]	binding
	target 5' U U A U 3'<br>GCA AACAGGGGAGGC GGUAUG<br>CGU UUGUCCCUUCUG CUAUAC<br>miRNA 3' U C C 5'	-34.7	433	DB932049	similar to ssm4 protein [Ricinus communis, EEF43655.1]	binding
	target 5' A U G A A 3'<br>CA GGA GG GGAGGAUGGUGUG<br>GU CUU UC CCUUCUGCUAUAC<br>miRNA 3' UC G C 5'	-28.5	299	DB922037	similar to pectin acetylesterase [Ricinus communis, EEF42516.1]	catalytic activity
**ACMV-mir-6-17***	target 5' A A A G 3'<br>CAGAA GGGGGAGA GAUAUG<br>GUCUU UCCCUUCU CUAUAC<br>miRNA 3' G G 5'	-30.5	397	FF381012	similar to protein phosphatase 2a, regulatory subunit [Ricinus communis, EEF42877.1]	enzyme regulator activity
	target 5' C U G 3'<br>GGACA GGAAGGUGAUAUG<br>CUUGU CCUUCUGCUAUAC<br>miRNA 3' GU C 5'	-27.1	75	DB938056	similar to poly-A binding protein [Ricinus communis, EEF30719.1]	binding
**ACMV-mir-6-18***	target 5' U A 3'<br>UAGGAUAGGGAAGGUGG	-27	312	DB921988	similar to mta/sah nucleosidase [Ricinus communis, EEF45888.1]	catalytic activity

	GUCUUGUCCCUUCUGCU<br>miRNA 3' UC 5'					
	target 5' U C A 3'<br>AGCGG GGCAGGGAGGAU<br>UCGUC UUGUCCCUUCUG<br>miRNA 3' CU 5'	-30.6	177	FF536058	similar to nucleic acid binding protein [Ricinus communis,EEF35238.1]	binding
**ACMV-mir-7-1**	target 5' U C C A 3'<br>GUG UUCUG AUGGCAUUCU<br>CAC GAGAU UACCGUAAGA<br>miRNA 3' UC A U 5'	-27.6	547	DB937072	similar to catalytic [Ricinus communis, EEF34353.1]	binding
	target 5' A A C 3'<br>GGG UGUUUC GAA GGCAUUCU<br>UCC ACAGAG UUU CCGUAAGA<br>miRNA 3' A A 5'	-26.2	442	DB930190	similar to beta-mannosidase [Ricinus communis, EEF49833.1]	binding
**ACMV-mir-7-2***	target 5' A C A A 3'<br>AGACACUC AU AGGGACAUUCU<br>UCUGUGAG UG UCUCUGUGAGA<br>miRNA 3' U A 5'	-34	138	DB953761	similar to mak [Ricinus communis, EEF42474.1]	binding
	target 5' A G 3'<br>AGA ACUCAACUAGAGACAUU<br>UCU UGAGUUGAUCUCUGUGA<br>miRNA 3' G GA 5'	-33.6	29	CK648460	similar to speckle-type POZ protein [Ricinus communis, EEF45411.1]	
	target 5' G G 3'<br>GGAUGCUU GCUGGAGGUACU<br>UCUGUGAG UGAUCUCUGUGA<br>miRNA 3' U GA 5'	-26.6	158	DV452362	similar to NADH-cytochrome B5 reductase [Ricinus communis, EEF34822.1]	catalytic activity
	target 5' U G C A 3'<br>GGG ACUC ACUGGGGAUACUC<br>UCU UGAG UGAUCUCUGUGAG<br>miRNA 3' G U A 5'	-34.2	304	BM259935	similar to CDK [Ricinus communis, EEF52923.1]	binding

	target 5' G G C 3'<br>GGGCAU CAGUUGGAGAUAUUC<br>UCUGUG GUUGAUCUCUGUGAG<br>miRNA 3' A A 5'	-28.7	384	DV442317	similar to 40S ribosomal protein S23 [Ricinus communis, XP_002525181.1]	structural molecule activity
	target 5' U C A 3'<br>GG CACUUGAUUAGAGGCGCU<br>UC GUGAGUUGAUCUCUGUGA<br>miRNA 3' U GA 5'	-32	228	CK640931	similar to chalcone synthase [Populus trichocarpa]	structural molecule activity
**ACMV-mir-7-3***	target 5' A G 3'<br>AGA ACUCAACUAGAGACAUU<br>UCU UGAGUUGAUCUCUGUGA<br>miRNA 3' G 5'	-33.2	29	CK648460	similar to speckle-type POZ protein [Ricinus communis, EEF45411.1]	
	target 5' G G 3'<br>GGAUGCUUG CUGGAGGUACU<br>UCUGUGAGU GAUCUCUGUGA<br>miRNA 3' U 5'	-26.2	158	DV452362	similar to NADH-cytochrome B5 reductase [Ricinus communis, EEF34822.1]	catalytic activity
	target 5' U C A 3'<br>GG CACUUGAUUAGAGGCGCU<br>UC GUGAGUUGAUCUCUGUGA<br>miRNA 3' U 5'	-31.6	228	CK640931	similar to chalcone synthase [Populus trichocarpa]	catalytic activity
	target 5' U A A 3'<br>GGACA UCGACUGGGGAUGC<br>UCUGU AGUUGAUCUCUGUG<br>miRNA 3' G A 5'	-31.3	58	DB926338	similar to calmodulin-binding heat-shock protein [Ricinus communis, EEF30464.1]	catalytic activity
**EACMV-UG-mir-1-1**	target 5' U C 3'<br>UAAAUCUCCUUUGUUUUGGAA<br>GUUUAGGGGAGAUAAAGCUUU<br>miRNA 3' AUU 5'	-29.1	328	DV453191	similar to density-regulated protein [Ricinus communis, EEF48071.1]	binding
	target 5' U A C 3'<br>GCAGAUCCCUUC GUUUUGAAA	-31.5	108	DB947992	similar to phenazine biosynthesis protein [Ricinus communis, EEF41730.1]	catalytic activity

	UGUUUAGGGGAG UAAAGCUUU<br>miRNA 3' AU A 5'					
	target 5' A G A G 3'<br>UAGCAGAUCU CU UAUUUUGAAA<br>AUUGUUUAGG GA AUAAAGCUUU<br>miRNA 3' G G 5'	27.6	269	FF535557	similar to sentrin/sumo-specific protease [Ricinus communis, EEF45541.1]	catalytic activity
	target 5' G U C A 3'<br>AACAA UCCCUUCU AUUUCGGAA<br>UUGUU AGGGGAGA UAAAGCUUU<br>miRNA 3' A U 5'	-31.6	173	CK642230	similar to sucrose synthase [Manihot esculenta, ABD96570.1]	catalytic activity
	target 5' A C U 3'<br>UGGCAAAUCUUUUC AUUUUGG<br>AUUGUUUAGGGGAG UAAAGCUU<br>miRNA 3' A U 5'	-25.1	190	DV444267	similar to peptidyl-prolyl cis-trans isomerase [Ricinus communis, EEF28526.1]	catalytic activity
	target 5' C C G C 3'<br>AC AGUUC CCUCUGUUUCGGA<br>UG UUAGG GGAGAUAAAGCUU<br>miRNA 3' AU U U 5'	-28.2	143	DB923200	similar to abc transporter [Ricinus communis, EEF33143.1]	binding
	target 5' C C A A 3'<br>AGCAG UCCUUUC GUUUUGAAA<br>UUGUU AGGGGAG UAAAGCUUU<br>miRNA 3' A U A 5'	-26.2	70	DB923059	similar to chloroplast photosystem II 10 kDa polypeptide [Jatropha curcas, ADB93062.1]	
	target 5' U A C 3'<br>GCAGAUCCCUUC GUUUUGAAA<br>UGUUUAGGGGAG UAAAGCUUU<br>miRNA 3' AU A 5'	-31.5	82, 2x 105	DB952982	similar to phenazine biosynthesis protein [Ricinus communis, EEF41730.1]	catalytic activity
**EACMV-UG-mir-1-2**	target 5' A C C 3'<br>AU ACAGAUUUCCUCUAUUUU<br>UA UGUUUAGGGGAGAUAAAG<br>miRNA 3' G U CU 5'	-26.8	72	CK644982	similar to oxysterol-binding protein-related protein 3C [Vitis vinifer]	binding

	target 5' A A G C 3'<br>AUGAUGAGUCC U UCUGUUUUG<br>UAUUGUUUAGG G AGAUAAAGC<br>miRNA 3' G G U 5'	-25.1	544	DB921739	similar to DNA photolyase [Ricinus communis, EEF39277.1]	catalytic activity
**EACMV-UG-mir-1-3**	target 5' A C G 3'<br>AUGGCAAAUCUUUUC AUUUUGG<br>UAUUGUUUAGGGGAG UAAAGCU<br>miRNA 3' A 5'	25.7	189	DV444267	similar to peptidyl-prolyl cis-trans isomerase [Ricinus communis, EEF28526.1]	catalytic activity
	target 5' A G A A 3'<br>AUAGCAGAUCU CU UAUUUUGA<br>UAUUGUUUAGG GA AUAAAGCU<br>miRNA 3' G G 5'	-26.9	268	FF535557	similar to sentrin/sumo-specific protease [Ricinus communis, EEF45541.1]	catalytic activity
	target 5' U A A 3'<br>GUAAUGGAUUUCCUCUGU UUGA<br>UAUUGUUUAGGGGAGAUA AGCU<br>miRNA 3' A 5'	-27.6	337	DV449149	similar to 2-deoxyglucose-6-phosphate phosphatase [Ricinus communis, EEF49975.1]	binding
	target 5' A A U 3'<br>AACA AGUCUCCUCUGUUUU<br>UUGU UUAGGGGAGAUAAAG<br>miRNA 3' UA CU 5'	-27.1	234	DB933365	similar to hydrolase, hydrolyzing O-glycosyl compounds, [Ricinus communis, EEF52605.1]	binding
**EACMV-UG-mir-1-4**	target 5' A G C 3'<br>AACGAA CCUCUCUAUUUC<br>UUGUUU GGGGAGAUAAAG<br>miRNA 3' UA A C 5'	-27.8	19	DV442611	similar to thylakoid lumenal 16.5 kDa protein, chloroplast precursor [Ricinus communis, EEF35210.1]	
	target 5' A U C 3'<br>AUGGCAAAUCCUUUCU UUUU<br>UAUUGUUUAGGGGAGA AAAG<br>miRNA 3' U C 5'	-25.5	403	DB941217	similar to coproporphyrinogen III oxidase [Ricinus communis, EEF43783.1]	catalytic activity
**EACMV-UG-mir-1-5**	target 5' A C C 3'	-26.5	72	CK644982	similar to oxysterol-binding protein-related protein 3C [Vitis vinifer]	binding

	AU ACAGAUUUCCUCUAUUUU<br>UA UGUUUAGGGGAGAUAAAG<br>miRNA 3' UG U 5'					
	target 5' A A G C 3'<br>ACA AACGAA CCUCUCUAUUUC<br>UGU UUGUUU GGGGAGAUAAAG<br>miRNA 3' A A 5'	-29.4	15	DV442611	similar to thylakoid lumenal 16.5 kDa protein, chloroplast precursor [Ricinus communis, EEF35210.1]	
	target 5' A U C 3'<br>AUAACAAGUUUCCUUU UUUC<br>UAUUGUUUAGGGGAGA AAAG<br>miRNA 3' UG U 5'	-25.3	16	BM260106	similar to 50S ribosomal protein L29, chloroplast precursor, putative [Ricinus communis]	catalytic activity
	target 5' U A A 3'<br>GCAUGACGGAUCUUUUCUAU UUC<br>UGUAUUGUUUAGGGGAGAUA AAG<br>miRNA 3' 5'	-29.8	717	FF536261	similar to nuclear acid binding protein [Ricinus communis, EEF43886.1]	binding
	target 5' U G 3'<br>UAUA UGAAUCCCUUCUGUUU<br>GUAU GUUUAGGGGAGAUAAA<br>miRNA 3' U U G 5'	-26.3	256	FF534999	similar to 2-deoxyglucose-6-phosphate phosphatase [Ricinus communis, EEF49975.1]	binding
**EACMV-UG-mir-1-6**	target 5' A A G C 3'<br>ACA AACGAA CCUCUCUAUUU<br>UGU UUGUUU GGGGAGAUAAA<br>miRNA 3' C A A 5'	-26.4	15	DV442611	similar to thylakoid lumenal 16.5 kDa protein, chloroplast precursor [Ricinus communis, EEF35210.1]	
	target 5' U G 3'<br>UAUA UGAAUCCCUUCUGUUU<br>GUAU GUUUAGGGGAGAUAAA<br>miRNA 3' CU U 5'	-25.9	116	FG807027	similar to 2-deoxyglucose-6-phosphate phosphatase, [Ricinus communis, EEF49975.1]	binding

**EACMV-UG-mir-1-7**	target 5' A A G U 3'<br>ACA AACGAA CCUCUCUAUU<br>UGU UUGUUU GGGGAGAUAA<br>miRNA 3' C A A 5'	-25.5	15	DV442611	similar to thylakoid lumenal 16.5 kDa protein, chloroplast precursor [Ricinus communis, EEF35210.1]	
	target 5' A A A 3'<br>ACA AGCAGGUCCUCUCUAU<br>UGU UUGUUUAGGGGAGAUA<br>miRNA 3' C A A 5'	-30	665	DV445085	similar to carbonyl reductase [Ricinus communis, EEF50490.1]	catalytic activity
	target 5' C G 3'<br>CAUAGCAAAUCCUCUUUA<br>GUAUUGUUUAGGGGAGAU<br>miRNA 3' CU AA 5'	-29.2	346	DB946990	similar to lupus la ribonucleoprotein, [Ricinus communis, EEF33296.1]	binding
	target 5' C U A A 3'<br>CAUG CAAAU CCCUCUGUU<br>GUAU GUUUA GGGAGAUAA<br>miRNA 3' CU U G 5'	-25.1	54	FF536711	similar to gamma-glutamyl hydrolase precursor [Ricinus communis, EEF37850.1]	catalytic activity
	target 5' G A A A 3'<br>GGCAUAG GAA UCCCUCUGU<br>CUGUAUU UUU GGGGAGAUA<br>miRNA 3' G A A 5'	-26	1	DB935104	similar to phospholipase A21 [Ricinus communis, EEF38745.1]	binding
**EACMV-UG-mir-1-8**	target 5' U G 3'<br>UGGGG A GACGGAUUUCUUCUG<br>ACCCU U UUGUUUAGGGGAGAU<br>miRNA 3' G A A 5'	-25.1	327	DB945313	similar to conserved hypothetical protein [Ricinus communis, EEF45059.1]	
**EACMV-UG-mir-1-9**	target 5' U G U G 3'<br>GGACGUAA GA UCCCCUCU<br>CCUGUAUU UU AGGGGAGA<br>miRNA 3' G U 5'	-32.7	360	DB924479	similar to glyceraldehyde 3-phosphate dehydrogenase [Ricinus communis, EEF49293.1]	binding

	target 5' U C U 3'<br>GAUG AACAAAUCCUCUCU<br>CUGU UUGUUUAGGGGAGA<br>miRNA 3' C A 5'	-26.7	637	CK652148	similar to elongation factor 1-alpha [Ricinus communis, EEF23174.1]	binding
	target 5' U C 3'<br>GACGUGGCAGAUCUCCUUU<br>CUGUAUUGUUUAGGGGAGA<br>miRNA 3' C 5'	-30.7	567	FF535038	similar to DNA binding protein [Ricinus communis, EEF43744.1]	binding
	target 5' A C 3'<br>ACAUAACAAGUCCUCUU<br>UGUAUUGUUUAGGGGAG<br>miRNA 3' CC A 5'	-28.3	496	DB940432	similar 40S ribosomal protein S3a [Ricinus communis, EEF45248.1]	structural molecule activity
	target 5' U A 3'<br>GGGCA GACAGGUCCCUU<br>CCUGU UUGUUUAGGGGA<br>miRNA 3' A GA 5'	-27.1	203	DB941027	similar to proteasome maturation protein [Ricinus communis, EEF36388.1]	
	target 5' U A U 3'<br>ACA AACGAAUCCUCUCU<br>UGU UUGUUUAGGGGAGA<br>miRNA 3' CC A 5'	-26.7	282	DB954927	similar to sugar transporter [Ricinus communis, EEF52582.1]	transporter activity
	target 5' U C C 3'<br>GGCA GAUAAGUCUCCUCU<br>CUGU UUGUUUAGGGGAGA<br>miRNA 3' C A 5'	-27.8	305	DB920165	similar to glutathione s-transferase [Ricinus communis, EEF29553.1]	catalytic activity
	target 5' C A 3'<br>GAUG GACAAAUUCCCUC<br>CUGU UUGUUUAGGGGAG<br>miRNA 3' C A A 5'	-25.6	284	DB925456	similar to polyneuridine-aldehyde esterase precursor [Ricinus communis, EEF40028.1)	catalytic activity
**EACMV-UG-mir-1-10**	target 5' A C A 3'<br>ACCUGGGAUGU ACAAAUUC	-32.2	164	BI325178	similar to zinc finger protein [Ricinus communis, EEF40424.1]	binding

	UGGACCCUGUA UGUUUAGG<br>miRNA 3' AA U G 5'					
	target 5' C U G A 3'<br>UUACUUG GGC UGA CAGAUCCC<br>AAUGGAC CUG AUU GUUUAGGG<br>miRNA 3' C U 5'	-27.7	104	DR085496	similar to chitinase [Ricinus communis, EEF43061.1]	binding
	target 5' A U 3'<br>ACUUGGG CA GACAGGUCCC<br>UGGACCC GU UUGUUUAGGG<br>miRNA 3' AA U A 5'	-31.4	199	DB941027	similar to proteasome maturation protein [Ricinus communis, EEF36388.1]	
	target 5' A G 3'<br>GCUUGGGAU U GCAAAUCCU<br>UGGACCCUG A UGUUUAGGG<br>miRNA 3' AA U U 5'	-28.7	326	FF535269	similar to kinesin light chain [Ricinus communis, EEF43396.1]	
	target 5' C C U A 3'<br>UACUU G A CAUGGCAAAUCCC<br>AUGGA C U GUAUUGUUUAGGG<br>miRNA 3' A C C 5'	-25.9	92	DB932241	similar to sucrose synthase [Ricinus communis, EEF39300.1]	catalytic activity
	target 5' A A U A 3'<br>UGCUUG GGGU UGACAAAUCC<br>AUGGAC CCUG AUUGUUUAGG<br>miRNA 3' A U G 5'	-27.6	547	DB932203	similar to ankyrin repeat domain protein [Ricinus communis, EEF47974.1]	binding
**EACMV-UG-mir-1-11**	target 5' U U A 3'<br>UUGCCUGGGG C UGGCAAAU<br>AAUGGACCCU G AUUGUUUA<br>miRNA 3' U 5'	-26.9	376	CK642833	similar to cyanohydrin UDP-glucosyltransferase UGT85K5 [Manihot esculenta, AEO45782.1]	catalytic activity
	target 5' C A A A 3'<br>GCC UGGGA AUGGCAAAU<br>UGG ACCCU UAUUGUUUA<br>miRNA 3' AA G 5'	-25.3	116	DB925098	similar to chlorophyll A/B binding protein [Ricinus communis, EEF36594.1]	

**EACMV-UG-mir-1-12***	target 5' C U C A 3'<br>UUU UCGCU CGCCUCAAGUAA<br>GAG AGUGA GCGGAGUUCGUU<br>miRNA 3' UU U C 5'	-32.1	176	FF536419	similar to defective in cullin neddylation protein [Ricinus communis, EEF35070.1]	binding
	target 5' G C 3'<br>UUUAUCAUUGCGCUUUAAGC<br>GAGUAGUGACGCGGAGUUCG<br>miRNA 3' UU UU 5'	-34.8	249	GR422139	similar to dual specificity phosphatase Cdc25 [Ricinus communis, EEF45101.1]	catalytic activity
	target 5' U C U 3'<br>GCUCAUCACUGU CUUUGAGUAG<br>UGAGUAGUGACG GGAGUUCGUU<br>miRNA 3' U C 5'	-34.6	461	DB954094	similar to receptor serine/threonine kinase [Ricinus communis, EEF48962.1]	binding
**EACMV-UG-mir-1-13***	target 5' U C G 3'<br>GCUCAUCACUGU CUUUGAGUA<br>UGAGUAGUGACG GGAGUUCGU<br>miRNA 3' CU C 5'	-34.2	461	DB954094	similar to receptor serine/threonine kinase [Ricinus communis, EEF48962.1]	binding
	target 5' G G G U 3'<br>GAA UUUGUCA UGCGUCUCAGGC<br>CUU GAGUAGU ACGCGGAGUUCG<br>miRNA 3' G U 5'	-34.6	21	FF381798	similar to regulatory-associated protein of tor 1-like [Glycine max, XP_003533671.1]	
	target 5' U U C 3'<br>GGCUUA C CUGC GUUUCAGGC<br>UUGAGU G GACG CGGAGUUCG<br>miRNA 3' C A U U 5'	-26.8	535	DB940712	similar to rnf5 [Ricinus communis, EEF52524.1]	catalytic activity
**EACMV-UG-mir-1-14***	target 5' G G G U 3'<br>GGAA UUUGUCA UGCGUCUCAGGC<br>CCUU GAGUAGU ACGCGGAGUUCG<br>miRNA 3' G 5'	-37.9	20	FF381798	similar to regulatory-associated protein of tor 1-like [Glycine max, XP_003533671.1]	

	target 5' C U G 3'<br>GAGUUCAUUAUUGC CCUUGGG<br>CUUGAGUAGUGACG GGAGUUC<br>miRNA 3' C C G 5'	-28.9	347	DV446218	similar to NAD dependent epimerase/dehydratase [Ricinus communis, EEF51074.1]	binding
	target 5' A U G 3'<br>AGCUCAUUAUUGC CCUUGGG<br>UUGAGUAGUGACG GGAGUUC<br>miRNA 3' CC C G 5'	-31.1	367	DB944961	similar to NAD dependent epimerase/dehydratase [Ricinus communis, EEF51074.1]	binding
	target 5' U U U U 3'<br>GGAUUUGUCGCUG C CUUUGAGU<br>CUUGAGUAGUGAC G GGAGUUCG<br>miRNA 3' C C 5'	-27.4	210	DB922734	similar to vacuolar protein sorting protein [Ricinus communis, EEF42794.1]	catalytic activity
	target 5' U G G A 3'<br>GAG U GUUACU GUGCCUCGGG<br>CUU A UAGUGA CGCGGAGUUC<br>miRNA 3' C G G G 5'	-28.1	498	DB940358	similar to heat shock 70 kDa protein [Ricinus communis, EEF34206.1]	binding
**EACMV-UG-mir-1-15***	target 5' U C 3'<br>UUAUCAUUGCGCUUUAAGC<br>AGUAGUGACGCGGAGUUCG<br>miRNA 3' G 5'	-34.4	250	GR422139	similar to dual specificity phosphatase Cdc25 [Ricinus communis, EEF45101.1]	catalytic activity
	target 5' G C A 3'<br>CUCAUCACUGU CUUUGAGU<br>GAGUAGUGACG GGAGUUCG<br>miRNA 3' C 5'	-30.5	462	DB954094	similar to receptor serine/threonine kinase [Ricinus communis, EEF48962.1]	binding
	target 5' A G U G 3'<br>CUUAUCACU G UGCUUCAAG<br>GAGUAGUGA C GCGGAGUUC<br>miRNA 3' G 5'	-28.3	512	FF534681	similar to serine-threonine protein kinase, plant-type [Ricinus communis, EEF45192.1]	binding

	target 5' G A A A 3'<br>CAUCA U UGUGCCUUGGGU<br>GUAGU G ACGCGGAGUUCG<br>miRNA 3' GA 5'	-25	190	FF535400	similar to gamma-interferon-inducible lysosomal thiol reductase precursor [Ricinus communis, EEF34301.1]	catalytic activity
	target 5' U U A 3'<br>UUGUUG CUGUGCCUCAA<br>AGUAGU GACGCGGAGUU<br>miRNA 3' G CG 5'	259	-26.3	DB921043	similar to chloroplast ferritin 2 [Jatropha curcas, ACV50433.1]	binding
**EACMV-UG-mir-1-16***	target 5' U G G C 3'<br>GGGAA UUUGUCA UGCGUCUCAGG<br>CCCUU GAGUAGU ACGCGGAGUUC<br>miRNA 3' G 5'	-37	19	FF381798	similar to regulatory-associated protein of tor 1-like [Glycine max, XP_003533671.1]	
	target 5' U U U A 3'<br>GGG ACUUGUUAUU UGCUUCAA<br>CCC UGAGUAGUGA GCGGAGUU<br>miRNA 3' U C C 5'	-28.7	378	DB929051	similar to potassium transporter [Ricinus communis, EEF44904.1]	transporter activity
	target 5' U U G A G 3'<br>GG GGAUUUG UGC GUGUUUCAAG<br>CC CUUGAGU GUG CGCGGAGUUC<br>miRNA 3' A A 5'	-25	45	DB929495	similar to s-receptor kinase [Ricinus communis, EEF33110.1]	binding
**EACMV-UG-mir-1-17***	target 5' U U A A 3'<br>GG GGAGCU GUUACUGUGUCU<br>CC CCUUGA UAGUGACGCGGA<br>miRNA 3' GU G G 5'	-34.4	330	FF381021	similar to ATP-dependent Clp protease proteolytic subunit [Ricinus communis, EEF34901.1]	binding
**EACMV-UG-mir-1-18***	target 5' U U A A 3'<br>GG GGAGCU GUUACUGUGUCU<br>CC CCUUGA UAGUGACGCGGA<br>miRNA 3' U G 5'	-34	330	FF381021	similar to ATP-dependent Clp protease proteolytic subunit [Ricinus communis, EEF34901.1]	binding

	target 5' G G U G 3'<br>GGGGGA GCUCAUUAUUGC UCU<br>UCCCCU UGAGUAGUGACG GGA<br>miRNA 3' C 5'	-34.8	387	DB938664	similar to protein phosphatase [Ricinus communis, EEF49929.1]	catalytic activity
**EACMV-UG-mir-1-19***	target 5' A A U C 3'<br>UCG AC GGGGAGCUCGUCAUU<br>AGC UG CCCCUUGAGUAGUGA<br>miRNA 3' AG G U 5'	-36.6	205	FF380533	similar to hydrolyzing O-glycosyl compounds [Ricinus communis, EEF30440.1]	binding
	target 5' G A A G 3'<br>CUCGCGUGG G AACUUGUCGCU<br>GAGCGUGUC C UUGAGUAGUGA<br>miRNA 3' A C C 5'	-28.7	222	DV453504	similar to pumilio [Ricinus communis, EEF38125.]	binding
	target 5' G U G U C 3'<br>UCUU CA AGG GAAUUCGUUGCU<br>AGAG GU UCC CUUGAGUAGUGA<br>miRNA 3' C G C 5'	-28.6	441	FF380021	similar to ubiquinone biosynthesis protein COQ9, mitochondrial precursor, [Ricinus communis, EEF46249.1]	binding
	target 5' G A G 3'<br>CUU UACAG GGGAGCUCAUUA<br>GAG GUGUC CCCUUGAGUAGU<br>miRNA 3' A C GA 5'	-31.4	479	DB935812	similar to phenylalanine ammonia-lyase 2 [Manihot esculenta]	catalytic activity
	target 5' U A A 3'<br>UCU GCA AGG GAAUUCGUUGC<br>AGA CGU UCC CUUGAGUAGUG<br>miRNA 3' G G C A 5'	-26.5	279	DB935731	similar to WD-repeat protein [Ricinus communis, EEF50650.1]	
	target 5' A G 3'<br>UCUCG GUAGGGGAACUCAUCA<br>AGAGC UGUCCCCUUGAGUAGU<br>miRNA 3' G GA 5'	-37.7	453	DB944314	similar to serine/threonine-protein kinase PBS1 [Ricinus communis, EEF52379.1]	binding
**EACMV-UG-mir-2-1**	target 5' A A C 3'	-32.6	180	DV446663	similar to ubiquitin-conjugating enzyme E2 [Ricinus communis, EEF51304.1]	binding

	GUAUGCC UGGGCUAAAUGCU<br>UAUAUGG ACUCGAUUUACGA<br>miRNA 3' C 5'					
	target 5' U U C A 3'<br>UAU CCUG GCU GGGUGCUG<br>AUA GGAC CGA UUUACGAC<br>miRNA 3' U U U 5'	-25	404	FF535175	similar to dihydrolipoamide acetyltransferase component of pyruvate dehydrogenase [Ricinus communis, EEF41039.1]	catalytic activity
	target 5' C G U 3'<br>UGUAUU GAGCUGAAUGCU<br>AUAUGG CUCGAUUUACGA<br>miRNA 3' U A C 5'	-27.5	324	DB927266	similar to replication factor C / DNA polymerase III gamma-tau subunit [Ricinus communis, EEF51126.1]	binding
**EACMV-UG-mir-2-2**	target 5' U U U G 3'<br>GCUU AGCUAAAUGCU GCCUU<br>UGGA UCGAUUUACGA CGGGA<br>miRNA 3' C C 5'	-32.1	454	FF379719	similar to protein with unknown function [Ricinus communis, EEF41267.1]	
	target 5' A U A 3'<br>UUGAGUUAAAU CUGGUUC<br>GACUCGAUUUA GACCGGG<br>miRNA 3' UG C A 5'	-28.4	471	DB923111	similar to ent-kaurene synthase B, chloroplast precursor [Ricinus communis, EEF28689.1]	catalytic activity
	target 5' C U A G 3'<br>ACCUGAGCU GA GCUGGUCC<br>UGGACUCGA UU CGACCGGG<br>miRNA 3' U A A 5'	-38.6	456	DB940414	similar to hypothetical protein RCOM_0911910 [Ricinus communis, EEF45287.1]	
	target 5' G U G 3'<br>UUUG GCU GAUGCUGGCCUU<br>GGAC CGA UUACGACCGGGA<br>miRNA 3' U U U 5'	-32.9	42	FF380276	similar to disease resistance protein RPM1 [Ricinus communis, EEF34466.1]	binding

	target 5' U A G 3'<br>UCUG GC UGGAUGUUGGCUUU<br>GGAC CG AUUUACGACCGGGA<br>miRNA 3' U U 5'	-29.5	163	FF380919	similar to probable peptide/nitrate transporter At1g59740-like [Vitis vinifera, XP_002268504.1]	transporter activity
	target 5' A A A 3'<br>GC UG GCUA AUGCUGGCCCU<br>UG AC CGAU UACGACCGGGA<br>miRNA 3' G U U 5'	-34.1	83	FF380201	similar to peptidyl-prolyl cis-trans isomerase [Ricinus communis, EEF47141.1]	catalytic activity
	target 5' G G 3'<br>CUGAGCU GAUGUUGGCCU<br>GACUCGA UUACGACCGGG<br>miRNA 3' UG U A 5'	-35.7	703	FF380166	similar to chalcone synthase [Ricinus communis, EEF44265.1]	catalytic activity
**EACMV-UG-mir-2-3***	target 5' U A C C 3'<br>UUAUGAAGCA A UUGGCUCU<br>AAUGCUUCGU U AAUCGAGG<br>miRNA 3' U C A 5'	-27.4	292	DR088252	similar to glucose-1-phosphate adenylyltransferase [Ricinus communis, EEF49428.1]	catalytic activity
**EACMV-UG-mir-2-4***	target 5' A A G 3'<br>AGC CUUGA U AGACGAGGCA<br>UCG GAGCU A UCUGCUUCGU<br>miRNA 3' A A 5'	-27.8	292	FF535731	similar to protein phosphatase 2c [Ricinus communis, EEF44394.1]	binding
	target 5' U G A 3'<br>G CU GAUUUGGACGAAGCG<br>C GA CUAAAUCUGCUUCGU<br>miRNA 3' U G G 5'	-29.7	315	DB935062	similar to r3h domain containing protein [Ricinus communis, EEF37398.1]	binding
**EACMV-UG-mir-2-5***	target 5' U U U U 3'<br>GG CUUU AUUUAGGCGAAGU<br>UC GGAG UAAAUCUGCUUCG<br>miRNA 3' C C 5'	-25	374	FF535553	similar to nucleic acid binding protein [Ricinus communis, EEF42371.1]	binding

	target 5' A A 3'<br>GGCUUUGA UUAGAUGAGG<br>UCGGAGCU AAUCUGCUUC<br>miRNA 3' C A G 5'	-26.9	303	FF536499	similar to mannosyl-oligosaccharide alpha-1,2-mannosidase [Ricinus communis, EEF34217.1]	binding
	target 5' U G G C 3'<br>GGCU CGGU UUGGAUGGGGU<br>UCGG GCUA AAUCUGCUUCG<br>miRNA 3' C A 5'	-25.1	168	DB924801	similar to cadmium-induced protein AS8 [Ricinus communis, EEF42469.1]	
**EACMV-UG-mir-2-6***	target 5' C U C 3'<br>AG GCCUUGGUUUGGAUGA<br>UC CGGAGCUAAAUCUGCU<br>miRNA 3' CU U U 5'	-30.8	398	DB930831	similar to hypothetical protein ZEAMMB73_258850 [Zea mays, AFW77571.1]	
	target 5' U G U 3'<br>GGAGGGUCU GGUUUGGGUGGA<br>CUUCUCGGA CUAAAUCUGCUU<br>miRNA 3' G 5'	-28.4	381	DV443635	similar to proteinase inhibitor [Ricinus communis, EEF41423.1]	enzyme regulator activity
	target 5' C U U 3'<br>AAG GCUU CGAUUUAGGCGG<br>UUC CGGA GCUAAAUCUGCU<br>miRNA 3' C U U 5'	-27.4	292	DV456795	similar to GTP-binding protein SAR1A [Medicago truncatula, AES65040.1]	binding
	target 5' G A U 3'<br>GGAG GCUUUGGUU UAGAUGGA<br>CUUC CGGAGCUAA AUCUGCUU<br>miRNA 3' U 5'	-26.1	192	DB922500	similar to cdk10/11 [Ricinus communis, EEF51826.1]	binding
	target 5' U U 3'<br>AGGG UUUGGUUUGGGCGA<br>UCUC GAGCUAAAUCUGCU<br>miRNA 3' CU G U 5'	-25.4	188	FF534916	similar to serine-type endopeptidase inhibitor [Ricinus communis, EEF47109.1]	enzyme regulator activity
**EACMV-UG-mir-2-7***	target 5' C U A A 3'	-27.8	404	CK641772	similar to grave disease carrier protein [Ricinus communis]	

	AUGA G AGAUUUGGCUUCGA<br>UGCU C UCUAAAUCGAGGCU<br>miRNA 3' U G 5'					
	target 5' A C G 3'<br>GAGGCGGAUU AGCUUUGA<br>CUUCGUCUAA UCGAGGCU<br>miRNA 3' UG A 5'	-30.6	482	DB933402	similar to DNA binding protein [Ricinus communis, EEF41947.1]	binding
	target 5' U U G G 3'<br>UGA AG GGAUUUGGCUCUGG<br>GCU UC UCUAAAUCGAGGCU<br>miRNA 3' U G 5'	-29	257	DB936972	similar to pectin acetylesterase [Ricinus communis, EEF42516.1]	catalytic activity
	target 5' A U 3'<br>AUG AGUGGGUUUGGCUUCGA<br>UGC UCGUCUAAAUCGAGGCU<br>miRNA 3' U 5'	-29.6	679	FF534378	similar to protein pob [Ricinus communis, EEF41790.1]	
**EACMV-UG-mir-2-8***	target 5' U A 3'<br>UGGGCGAAGUGUUGAUG<br>AUCUGCUUCGUAAUUGC<br>miRNA 3' G 5'	-26	155	FF379863	similar to peroxiredoxins, prx-1, prx-2, prx-3 [Ricinus communis, EEF32207.1]	catalytic activity
	target 5' A U A 3'<br>GGAC GAAGUAUUGAUGU<br>UCUG CUUCGUAAUUGCG<br>miRNA 3' A 5'	-25.5	376	DB951176	similar to phospholipid-transporting atpase [Ricinus communis, EEF47941.1]	binding

Table S7 Predicted putative targets of miRs/miRs* from ACMV and EACMV-UG in cassava ESTs using psRNATarget

miR/miR*	Expectation Score	Target start	Target end	Genbank accession	Target description	Target function
**ACMV-mir-1-1**	4	33	53	DB944076	similar to pyruvate kinase, cytosolic isozyme-like [Vitis vinifera, XP_002283911.1]	binding
	4.5	294	317	DV441932	similar to ring-h2 finger protein ATL3J [Ricinus communis, EEF46180.1]	binding
	4.5	340	363	DB951428	similar to aspartate aminotransferase [Ricinus communis, EEF46904.1]	binding
	4.5	297	319	FG804714	similar to kinase [Ricinus communis, EEF36760.1]	binding
	5	117	137	DV452869	similar to 50S ribosomal protein L36, [Ricinus communis, EEF37944.1]	structural molecule activity
**ACMV-mir-1-2**	3.5	147	166	CK641549	similar to ATP binding protein [Ricinus communis, EEF51206.1]	binding
	4	79	98	FF380185	similar to chaperone protein dnaJ [Ricinus communis, EEF52952.1]	binding
	4	24	43	DB953110	similar to similar to pyruvate kinase, cytosolic isozyme-like [Vitis vinifera, XP_002283911.1]	binding
	4.5	599	618	DB949511	similar to pyridoxamine 5-phosphate oxidase [Ricinus communis, EEF48587.1]	binding
	4.5	181	200	DB947066	similar to ATP-dependent clp protease [Ricinus communis, EEF51704.1]	binding
	4.5	298	317	DV441932	similar to ring-h2 finger protein ATL3J [Ricinus communis, EEF46180.1]	binding
	5	453	472	DB949248	similar to glyceraldehyde 3-phosphate dehydrogenase A subunit [Arabidopsis thaliana, BAD93961.1]	binding
**ACMV-mir-1-3**	3.5	56	75	DB942812	similar to pyruvate kinase, cytosolic isozyme-like [Vitis vinifera, XP_002283911.1]	binding
	4.5	458	478	DV448856	similar to ankyrin-repeat containing protein [Ricinus communis, EEF49533.1]	binding
	4.5	294	315	DV441932	similar to ring-h2 finger protein ATL3J [Ricinus communis, EEF46180.1]	binding
	4.5	340	361	DB951428	similar to aspartate aminotransferase [Ricinus communis, EEF46904.1]	binding
	4.5	330	350	DB941875	similar to ribonuclease t2 [Ricinus communis, EEF37789.1]	binding
	4.5	132	152	DB953264	similar to uridylate kinase [Ricinus communis, EEF35901.1]	binding
	5	76	97	DB928368	similar to eukaryotic translation initiation factor 3 subunit [Ricinus communis, EEF46838.1]	binding
**ACMV-mir-1-4**	3	457	476	DV448856	similar to ankyrin-repeat containing protein [Ricinus communis, EEF49533.1]	binding
	3.5	106	125	CK643583	similar to protein dimerization [Ricinus communis]	binding
	4	386	407	DB940463	similar to s-locus-specific glycoprotein S6 precursor [Ricinus communis, EEF37743.1]	binding
	4	292	313	DV441932	similar to ring-h2 finger protein ATL3J [Ricinus communis, EEF46180.1]	binding
	4.5	501	522	DB923039	similar to nucleoside transporter [Ricinus communis, EEF36986.1]	transporter activity
	4.5	133	154	DB953072	similar to lipoamide acyltransferase component of branched-chain alpha-keto acid dehydrogenase [Ricinus communis, EEF45565.1]	binding
	5	251	272	DB949051	similar to asymmetric leaves1 and rough sheath [Ricinus communis, EEF31393.1]	binding

	5	472	493	FF382006	similar to big map kinase/bmk [Ricinus communis, EEF33894.1]	binding
	5	407	428	DB944787	similar to zinc finger protein [Ricinus communis, EEF34907.1]	binding
**ACMV-mir-1-5**	3.5	453	475	DV448856	similar to ankyrin-repeat containing protein [Ricinus communis, EEF49533.1]	binding
	3.5	386	406	DB940463	similar to s-locus-specific glycoprotein S6 precursor [Ricinus communis, EEF37743.1]	binding
	4	251	271	DB949051	similar to asymmetric leaves1 and rough sheath [Ricinus communis, EEF31393.1]	binding
	4.5	405	427	DB944787	similar to zinc finger protein [Ricinus communis, EEF34907.1]	binding
	4.5	500	521	DB923039	similar to nucleoside transporter [Ricinus communis, EEF36986.1]	transporter activity
	5	160	182	CK651722	similar to protein YME1 [Ricinus communis, EEF45768.1]	binding
	5	290	312	DV441932	similar to ring-h2 finger protein ATL3J [Ricinus communis, EEF46180.1]	binding
	5	470	492	FF382006	similar to big map kinase/bmk [Ricinus communis, EEF33894.1]	binding
	5	771	792	FF535270	similar to vesicle-associated membrane protein [Ricinus communis, EEF50326.1]	structural molecule activity
	5	225	246	DV450585	similar to KDEL motif-containing protein 1 precursor [Ricinus communis, EEF50504.1]	
**ACMV-mir-1-6**	3	143	162	DB930211	similar to n-acetyltransferase [Ricinus communis, EEF29170.1]	catalytic activity
	3.5	452	472	DV448856	similar to ankyrin-repeat containing protein [Ricinus communis, EEF49533.1]	binding
	3.5	264	283	DV448292	similar to zinc finger protein [Ricinus communis, EEF37443.1]	binding
	3.5	238	261	DV449595	similar to ferritin [Ricinus communis, EEF35690.1]	binding
	4.5	336	357	DV443989	similar to translation initiation factor if-3 [Ricinus communis, EEF29148.1]	binding
	5	514	536	DB921840	similar to nucleoside transporter [Ricinus communis, EEF37723.1]	transporter activity
	5	95	116	FF535177	similar to 26S protease regulatory subunit [Ricinus communis, EEF36160.1]	binding
**ACMV-mir-1-7**	3.5	211	232	DB940121	similar to methylglutaconyl-CoA hydratase, mitochondrial precursor [Ricinus communis, EEF30028.1]	binding
	3.5	549	568	FF536747	similar to U1 small nuclear ribonucleoprotein C [Ricinus communis, EEF41660.1]	binding
	4	25	44	DB921240	similar to calcineurin B subunit [Ricinus communis, EEF44849.1]	binding
	4.5	87	107	DB932028	similar to transcription factor MYC2 [Ricinus communis, EEF42418.1]	binding
	4.5	209	229	DB947562	similar to ankyrin repeat-containing protein [Ricinus communis, EEF31090.1]	binding
	5	469	489	DB951704	similar to serine/threonine protein kinase [Ricinus communis, EEF30552.1]	binding
**ACMV-mir-1-8**	3.5	212	232	DB940121	similar to methylglutaconyl-CoA hydratase, mitochondrial precursor [Ricinus communis, EEF30028.1]	binding
	3.5	549	568	FF536747	similar to U1 small nuclear ribonucleoprotein C [Ricinus communis, EEF41660.1]	binding
	4	25	44	DB921240	similar to calcineurin B subunit [Ricinus communis, EEF44849.1]	binding
	4.5	288	307	DB951056	similar to cysteine protease [Ricinus communis, EEF49466.1]	catalytic activity

	4.5	40	59	DB943889	similar to short-chain dehydrogenase [Ricinus communis, EEF51954.1]	binding
	5	685	704	DV443195	similar to ubiquitin-conjugating enzyme h [Ricinus communis, EEF51217.1]	binding
**ACMV-mir-1-9***	3.5	307	326	FF535752	similar to histone h2a [Ricinus communis, EEF44725.1]	binding
	4	321	340	DV441173	similar to 60S ribosomal protein L3 [Ricinus communis, EEF35945.1]	structural molecule activity
**ACMV-mir-1-11***	5	94	117	DV451954	similar to transcriptional corepressor SEUSS [Ricinus communis, EEF42030.1]	binding
**ACMV-mir-1-12***	4.5	59	79	DR084471	similar to glyceraldehyde 3-phosphate dehydrogenase [Ricinus communis, EEF51837.1]	binding
	4.5	189	211	DV450499	similar to hippocampus abundant transcript 1 protein [Ricinus communis, EEF37396.1	transporter activity
	4.5	94	116	DV451954	similar to transcriptional corepressor SEUSS [Ricinus communis, EEF42030.1]	binding
	4.5	181	201	DB929926	similar to coatomer delta subunit [Ricinus communis, EEF35793.1]	structural molecule activity
	5	127	149	DB945207	similar to protein binding protein [Ricinus communis, EEF44516.1]	
**ACMV-mir-1-13***	3.5	58	77	DR084471	similar to glyceraldehyde-3-phosphate dehydrogenase [Ricinus communis, EEF51837.1]	binding
	3.5	170	191	DV446448	similar to sedoheptulose-1,7-bisphosphatase, chloroplast [Ricinus communis, EEF31985.1]	binding
	4	171	190	DB943003	similar to hydrolase [Ricinus communis, EEF33634.1]	binding
	5	445	468	CK648767	similar to tetrahydrofolylpolyglutamate synthase [Ricinus communis, EEF48993.1]	binding
**ACMV-mir-1-14***	2.5	6	25	DV458694	similar to phosphoribosylamine-glycine ligase [Ricinus communis, EEF35937.1]	binding
	4	28	47	FF381465	similar to 60S ribosomal protein L7 [Ricinus communis, EEF38248.1]	structural molecule activity
	4	217	237	DV451804	similar to mitotic checkpoint protein bub3 [Ricinus communis, EEF34902.1]	
	4	8	27	DV442119	similar to DELLA protein GAIP-B [Ricinus communis, EEF37235.1]	binding
	4.5	473	492	DB922131	similar to protein PNS1 [Ricinus communis, EEF33259.1]	
	4.5	549	568	DV447543	similar to r2r3-myb transcription factor [Ricinus communis, EEF27899.1]	binding
	4.5	586	605	DB927893	similar to ankyrin repeat-containing protein [Arabidopsis thaliana, AED91210.1]	binding
	5	105	124	FF379815	similar to actin-depolymerizing factor 6 [Jatropha curcas, ADB85088.1]	
**ACMV-mir-2-1**	2	16	35	DB942360	similar to ribosomal protein S28 [Ricinus communis, XP_002531592.1]	structural molecule activity
	2.5	49	68	DB948720	similar to auxin-induced in root cultures protein 12 precursor [Ricinus communis, EEF30377.1]	
	3	483	502	DR084721	similar to hexokinase [Ricinus communis, EEF48175.1]	binding
	3	22	41	CK643553	similar to serine/threonine protein phosphatase [Ricinus communis]	binding

	3	196	215	FG806669	similar to phenylalanine ammonia-lyase 3 [Manihot esculenta, AF383150_1]	catalytic activity
	3.5	385	404	DV445976	similar to 5-formyltetrahydrofolate cyclo-ligase [Ricinus communis, EEF52087.1]	binding
	3.5	24	43	DV456975	similar to E3 ubiquitin-protein ligase UPL5-like [Glycine max, XP_003528704.1]	catalytic activity
	4	639	658	DV452906	similar to acyltransferase [Ricinus communis, EEF30758.1]	catalytic activity
	4.5	511	530	DV442563	similar to sulfite reductase [Ricinus communis, EEF50910.1]	electron carrier activity
	4.5	281	300	DV448413	similar to sec15 [Ricinus communis, EEF39290.1]	
	5	533	552	DB935433	similar to aspartic proteinase precursor [Ricinus communis, EEF32480.1]	catalytic activity
**ACMV-mir-2-2**	2.5	17	36	DB937669	similar to predicted protein [Populus trichocarpa, EEE72000.1]	
	3	482	501	DR084721	similar to hexokinase [Ricinus communis, EEF48175.1]	binding
	3	50	70	DV454666	similar to ribosomal protein S28 [Ricinus communis, XP_002531592.1]	structural molecule activity
	3.5	530	551	DB935433	similar to aspartic proteinase precursor [Ricinus communis, EEF32480.1]	catalytic activity
	3.5	507	529	DV442563	similar to sulfite reductase [Ricinus communis, EEF50910.1]	electron carrier activity
	3.5	23	42	DV456975	similar to E3 ubiquitin-protein ligase UPL5-like [Glycine max, XP_003528704.1]	catalytic activity
	3.5	324	343	DV455353	similar to carbonic anhydrase [Ricinus communis, EEF37015.1]	binding
	4	402	423	DR085317	similar to alternative oxidase 4, chloroplast precursor [Ricinus communis, EEF43798.1]	binding
	4	660	682	DV450054	similar to metal transporter [Ricinus communis, EEF42120.1]	transporter activity
	4	383	403	DV445976	similar to 5-formyltetrahydrofolate cyclo-ligase [Ricinus communis, EEF52087.1]	binding
	4	46	67	DB928052	similar to pentatricopeptide repeat-containing protein [Ricinus communis, EEF50485.1]	binding
	4.5	15	36	CK644658	similar to calmodulin binding protein [Ricinus communis]	binding
	4.5	419	440	DV454879	similar to poly-A binding protein [Ricinus communis, EEF30719.1]	binding
**ACMV-mir-2-3**	2	663	686	DV446975	similar to hypothetical protein RCOM_1573710 [Ricinus communis, EEF48719.1]	
	2.5	529	550	DB935433	similar to aspartic proteinase precursor [Ricinus communis, EEF32480.1]	catalytic activity
	3	383	402	DV445976	similar to 5-formyltetrahydrofolate cyclo-ligase [Ricinus communis, EEF52087.1]	binding
	3.5	402	422	DR085317	similar to alternative oxidase 4, chloroplast precursor [Ricinus communis, EEF43798.1]	binding
	3.5	505	528	DV442563	similar to sulfite reductase [Ricinus communis, EEF50910.1]	electron carrier activity
	3.5	12	35	CK644658	similar to calmodulin binding protein [Ricinus communis]	binding
	4	470	491	FF379968	similar to vesicle transport v-snare protein vti1a [Ricinus communis, EEF32373.1]	
	4.5	67	89	GR421415	similar to 60S ribosomal protein L3 [Ricinus communis, EEF38042.1]	structural molecule activity
	5	216	239	DB934108	similar to receptor protein kinase [Ricinus communis, EEF34847.1]	binding

**ACMV-mir-2-4**	2	525	548	DB935433	similar to aspartic proteinase precursor [Ricinus communis, EEF32480.1]	catalytic activity
	3	274	296	DV448413	similar to sec15 [Ricinus communis, EEF39290.1]	
	4	21	42	DB925685	similar to heparanase-2 [Ricinus communis, EEF47802.1]	catalytic activity
	4	143	166	BI325097	similar to chaperone protein dnaJ [Ricinus communis, ABH06547.1]	binding
	4	156	179	DB952735	similar to chaperone protein dnaJ, putative [Ricinus communis, ABH06547.1]	binding
	4	370	391	DV449788	similar to fructokinase [Ricinus communis, EEF29025.1]	catalytic activity
	4	216	237	DB934108	similar to receptor protein kinase [Ricinus communis, EEF34847.1]	binding
	4.5	401	424	DB948734	similar to porphobilinogen deaminase [Ricinus communis, EEF42025.1]	binding
**ACMV-mir-2-5**	2.5	21	41	DB925685	similar to heparanase-2 [Ricinus communis, EEF47802.1]	catalytic activity
	2.5	525	547	DB935433	similar to aspartic proteinase precursor [Ricinus communis, EEF32480.1]	catalytic activity
	3	274	295	DV448413	similar to sec15 [Ricinus communis, EEF39290.1]	
	3	216	236	DB934108	similar to receptor protein kinase [Ricinus communis, EEF34847.1]	binding
	3.5	401	423	DB948734	similar to porphobilinogen deaminase [Ricinus communis, EEF42025.1]	binding
	4	98	119	FF379904	similar to translation initiation factor 2b, delta subunit [Ricinus communis, EEF50656.1]	binding
	4.5	370	390	DV449788	similar to fructokinase [Ricinus communis, EEF29025.1]	catalytic activity
	5	249	271	DB930142	similar to potassium transporter [Ricinus communis, EEF49310.1]	transporter activity
**ACMV-mir-2-6**	2.5	20	39	DB925685	similar to heparanase-2 [Ricinus communis, EEF47802.1]	catalytic activity
	3	365	388	DV449788	similar to fructokinase [Ricinus communis, EEF29025.1]	catalytic activity
	3.5	82	105	DB936077	similar to 60S ribosomal protein L38 [Ricinus communis, EEF47361.1]	structural molecule activity
	4.5	603	626	DV451262	similar to fructose-1,6-bisphosphatase, cytosolic [Ricinus communis, EEF29949.1]	binding
**ACMV-mir-2-7**	3	82	104	DB936077	similar to 60S ribosomal protein L38 [Ricinus communis, EEF47361.1]	structural molecule activity
	3.5	393	416	DR085317	similar to alternative oxidase 4, chloroplast precursor [Ricinus communis, EEF43798.1]	binding
	3.5	19	38	DB925685	similar to heparanase-2 [Ricinus communis, EEF47802.1]	catalytic activity
	5	627	649	FF381034	similar to clathrin coat associated protein ap-50 [Ricinus communis, EEF42435.1]	
	5	545	565	DB927054	similar to nucellin [Ricinus communis, EEF39461.1]	catalytic activity
	5	602	625	DV451262	similar to fructose-1,6-bisphosphatase, cytosolic [Ricinus communis, EEF29949.1]	binding
**ACMV-mir-2-8***	3	220	240	DV450633	similar to beta-adaptin-like protein A [Glycine max, XP_003522688.1]	transporter activity
	3.5	56	76	DB945014	similar to kbp-type peptidyl-prolyl cis-trans isomerase 2, chloroplast, [Ricinus communis, EEF32649.1]	binding
	4	393	415	FF380166	similar to chalcone synthase [Ricinus communis, EEF44265.1]	catalytic activity
	4	478	500	DV455841	similar to aminomethyltransferase, Ricinus communis, EEF43843.1]	catalytic activity
	4	186	207	FG805474	similar to ring-h2 zinc finger protein [Manihot esculenta, AEQ20638.1]	binding

	4.5	155	176	DB946837	similar to purine permease [Ricinus communis, EEF52048.1]	transporter activity
	4.5	127	149	CK647992	similar to solute carrier family 35 member F5-like isoform 1 [Vitis vinifera, XP_002285575]	
	4.5	23	44	DB947282	similar to ring finger protein [Ricinus communis, EEF46787.1]	binding
	4.5	126	148	CK646013	similar to solute carrier family 35 member F5-like isoform 1 [Vitis vinifera, XP_002285575]	
	5	212	234	DR085969	similar to optic atrophy 3 protein [Ricinus communis, EEF39804.1]	
**ACMV-mir-3-1**	3	247	266	DV457211	similar to tRNA-pseudouridine synthase [Ricinus communis, EEF40978.1]	binding
	4	460	480	DB949701	similar to transmembrane protein 14 [Ricinus communis, EEF46177.1]	
	4	105	125	DR084409	similar to phosphatidylinositol 4-kinase type 2-beta [Medicago truncatula, AES93933.1]	catalytic activity
	4	46	66	DR084671	similar to NADH dehydrogenase subunit [Medicago truncatula, AES58603.1]	binding
	4	249	269	CK650220	similar to ATP-binding cassette transporter [Ricinus communis, EEF28273.1]	binding
	5	200	220	DB928794	similar to zinc finger CCCH domain-containing protein [Medicago truncatula, AET03818.1]	binding
	5	369	389	DB952007	similar to protein enhancer of rudimentary [Ricinus communis, EEF46862.1]	
	5	563	583	DB935443	similar to aspartic proteinase precursor [Ricinus communis, EEF32480.1]	catalytic activity
**ACMV-mir-3-2**	2	48	68	DB935122	similar to calcineurin B [Ricinus communis, EEF45699.1]	binding
	3	119	138	DB921275	similar to ATP-binding cassette transporter [Ricinus communis, EEF38310.1]	binding
	3.5	443	464	DV458768	similar to similar to transmembrane protein 14 [Ricinus communis, EEF39398.1]	
	4	298	319	DB944450	similar to ATP-dependent clp protease [Ricinus communis, EEF51704.1]	binding
	4	349	370	DB952898	similar to methionine-tRNA synthetase [Ricinus communis, EEF44189.1]	binding
	4	132	153	CK901238	similar to flavonoid 3',5'-hydroxylase [Citrus clementina, AEC50089.1]	electron carrier activity
	4.5	365	386	DV441052	similar to serine/threonine-protein kinase PBS1[Ricinus communis, EEF28053.1]	binding
	4.5	422	443	CK644012	similar to auxin-responsive protein IAA6 [Ricinus communis]	nucleic acid binding transcription factor activity
**ACMV-mir-3-3**	2	48	67	DB935122	similar to calcineurin B [Ricinus communis, EEF45699.1]	binding
	3	298	318	DB944450	similar to ATP-dependent clp protease [Ricinus communis, EEF51704.1]	binding
	3	294	314	DB950650	similar to methionine-tRNA synthetase [Ricinus communis, EEF44189.1]	binding
	3.5	132	152	CK901238	similar to flavonoid 3',5'-hydroxylase [Citrus clementina]	electron carrier activity
	4	115	137	DB921275	similar to ATP-binding cassette transporter [Ricinus communis, EEF38310.1]	binding
	4	152	175	DB947196	similar to auxin response factor [Ricinus communis, EEF28340.1]	binding
	4.5	180	203	DV446211	similar to metalloendoproteinase 1 precursor [Ricinus communis, EEF28446.1]	binding
**ACMV-mir-3-4***	1.5	109	128	CK643811	similar to phenylalanyl-tRNA synthetase [Ricinus communis, EEF33425.1]	binding

	3	329	351	CK645990	similar to lysM domain GPI-anchored protein 2 [Ricinus communis, EEF36585.1]	binding
	3	235	257	DB954572	similar to SWIB/MDM2 domain-containing protein [Arabidopsis thaliana, AAF03473.1]	binding
	3.5	454	476	FF380092	similar to 60S ribosomal protein L13aA [Hevea brasiliensis, ADR71263.1]	catalytic activity
	4	169	190	DV449505	similar to lipoxygenase [Ricinus communis, EEF49219.1]	binding
**ACMV-mir-3-5***	2	36	58	DV458538	similar to cold-inducible RNA-binding protein [Ricinus communis, EEF38570.1]	binding
	2.5	45	68	DV449925	similar to actin depolymerizing factor 4 [Hevea brasiliensis, ADV04049.1]	
	3	118	140	FF534285	similar to transferring glycosyl groups [Ricinus communis, EEF41222.1]	catalytic activity
	3	42	63	DV455184	similar to DAG protein, chloroplast precursor [Ricinus communis, EEF43977.1]	
	3	726	749	DV445323	similar to arginine/serine-rich-splicing factor [Ricinus communis, EEF51221.1]	binding
	3	76	99	DB920502	similar to protein binding protein [Ricinus communis, EEF45740.1]	
	4	3	25	FF535121	similar to allene oxide cyclase 4, chloroplast [Ricinus communis, EEF31990.1]	catalytic activity
	4	137	160	DB948980	similar to ATP synthase [Ricinus communis, EEF30158.1]	binding
	4.5	311	334	DB954030	similar to receptor serine-threonine protein kinase [Ricinus communis, EEF43165.1]	binding
	4.5	22	45	DB925338	similar to dead box ATP-dependent RNA helicase [Ricinus communis, EEF41044.1]	binding
**ACMV-mir-3-6***	2	58	78	DV446437	similar to DAG protein, chloroplast precursor [Ricinus communis, EEF43977.1]	
	2	49	70	DV443339	similar to cold-inducible RNA-binding protein [Ricinus communis, EEF38570.1]	binding
	2.5	118	139	FF534285	similar to transferring glycosyl groups [Ricinus communis, EEF41222.1]	catalytic activity
	2.5	45	67	DV449925	similar to actin depolymerizing factor 4 [Hevea brasiliensis, ADV04049.1]	
	2.5	216	239	CK652354	similar to transcription factor RF2a [Ricinus communis, EEF32889.1]	nucleic acid binding transcription factor activity
	3	124	147	DB937863	similar to alcohol dehydrogenase [Ricinus communis, EEF28016.1]	binding
	3.5	311	333	DB954030	similar to receptor serine-threonine protein kinase [Ricinus communis, EEF43165.1]	binding
	3.5	428	451	DB950434	similar to heat shock protein [Ricinus communis, EEF43398.1]	binding
	3.5	3	24	FF535121	similar to allene oxide cyclase 4, chloroplast precursor [Ricinus communis, EEF31990.1]	catalytic activity
	3.5	136	159	DB948980	similar to ATP synthase [Ricinus communis, EEF30158.1]	binding
**ACMV-mir-3-7***	2	118	138	FF534285	similar to transferring glycosyl groups [Ricinus communis, EEF41222.1]	catalytic activity
	2.5	3	23	FF535121	similar to allene oxide cyclase 4, chloroplast precursor [Ricinus communis, EEF31990.1]	catalytic activity
	3	427	450	DB950434	similar to heat shock protein [Ricinus communis, EEF43398.1]	binding
	3	22	43	DB925338	similar to dead box ATP-dependent RNA helicase [Ricinus communis, EEF41044.1]	binding

	3.5	134	157	DB926004	similar to pentatricopeptide repeat-containing protein [Ricinus communis, EEF28596.1]	binding
	3.5	263	284	DB922198	similar to amidophosphoribosyltransferase [Ricinus communis, EEF49515.1]	binding
	3.5	311	332	DB954030	similar to receptor serine-threonine protein kinase [Ricinus communis, EEF43165.1]	binding
	3.5	240	261	DB925153	similar to amidophosphoribosyltransferase [Ricinus communis, EEF49515.1]	binding
	5	237	260	DB954572	similar to SWIB/MDM2 domain-containing protein [Arabidopsis thaliana, AAF03473.1]	binding
**ACMV-mir-3-8***	2	118	137	FF534285	similar to transferring glycosyl groups [Ricinus communis, EEF41222.1]	catalytic activity
	2.5	401	421	DB948922	similar to protein with unknown function [Ricinus communis, EEF33873.1]	
	3.5	246	268	DV448023	similar to carbonic anhydrase [Ricinus communis] >gb|EEF37661.1	binding
	4	109	130	CK643811	similar to phenylalanyl-tRNA synthetase, chloroplastic/mitochondrial [Vitis vinifera]	binding
	4	427	449	DB950434	similar to heat shock protein [Ricinus communis, EEF43398.1]	binding
	4	215	238	DB942816	similar to calmodulin [Zea mays, ACG25844.1]	binding
	4.5	237	259	DB954572	similar to SWIB/MDM2 domain-containing protein [Arabidopsis thaliana, AAF03473.1]	binding
	4.5	192	215	DV447511	similar to 60S ribosomal protein L13aA [Hevea brasiliensis, ADR71263.1]	structural molecule activity
	5	169	192	DV449505	similar to lipoxygenase [Ricinus communis, EEF49219.1]	binding
**ACMV-mir-3-9***	2.5	108	127	CK643811	similar to phenylalanyl-tRNA synthetase, chloroplastic/mitochondrial [Vitis vinifera]	binding
	3	392	415	DB925921	similar to pentatricopeptide repeat-containing protein [Ricinus communis, EEF28596.1]	binding
	3	234	256	DB954572	similar to SWIB/MDM2 domain-containing protein [Arabidopsis thaliana, AAF03473.1]	binding
	4.5	428	451	FF535568	similar to 60S ribosomal protein L30 [Ricinus communis, EEF33410.1]	structural molecule activity
**ACMV-mir-3-11***	1.5	109	128	CK643811	similar to phenylalanyl-tRNA synthetase, chloroplastic/mitochondrial [Vitis vinifera]	binding
	3	234	257	DB954572	similar to SWIB/MDM2 domain-containing protein [Arabidopsis thaliana, AAF03473.1]	binding
	3.5	191	213	DV447511	similar to 60S ribosomal protein L13aA [Hevea brasiliensis, ADR71263.1]	structural molecule activity
	4	219	242	DB947102	similar to eukaryotic translation initiation factor 3 subunit [Ricinus communis, EEF46210.1]	binding
	4	169	190	DV449505	similar to lipoxygenase [Ricinus communis, EEF49219.1]	binding
**ACMV-mir-3-12***	2.5	108	127	CK643811	similar to phenylalanyl-tRNA synthetase, chloroplastic/mitochondrial [Vitis vinifera]	binding
	2.5	93	112	CK652796	similar to phenylalanyl-tRNA synthetase [Ricinus communis, EEF33425.1]	binding
	3	392	415	DB925921	similar to pentatricopeptide repeat-containing protein [Ricinus communis, EEF28596.1]	binding
	3	234	256	DB954572	similar to SWIB/MDM2 domain-containing protein [Arabidopsis thaliana, AAF03473.1]	binding

	4.5	428	451	FF535568	similar to 60S ribosomal protein L30 [Ricinus communis, EEF33410.1]	structural molecule activity
**ACMV-mir-3-13***	4	372	397	DV448789	similar to 60S ribosomal protein L30 [Ricinus communis, EEF33410.1]	structural molecule activity
	4.5	370	397	DV443985	similar to calmodulin binding protein [Ricinus communis, EEF39751.1]	binding
	5	39	66	DB929559	similar to sodium/hydrogen exchanger [Ricinus communis, EEF49734.1]	transporter activity
	5	284	311	DB925922	similar to malate dehydrogenase [Ricinus communis, EEF40237.1]	binding
	5	424	449	DB950660	similar to gamma-tocopherol methyltransferase [Hevea brasiliensis, BAH10645.1]	catalytic activity
	5	386	413	DB946667	similar to 30S ribosomal protein S8 [Ricinus communis, EEF33949.1]	structural molecule activity
**ACMV-mir-3-14***	2.5	460	479	CK645625	similar to zeamatin precursor [Ricinus communis, EEF28230.1]	
	3	1	20	DV444886	similar to glycosyltransferase CAZy family GT8 [Medicago truncatula, AES92758.1]	catalytic activity
	3.5	96	115	DB932480	similar to TMV resistance protein N [Ricinus communis, EEF38940.1]	binding
	4	106	125	CK643811	similar to phenylalanyl-tRNA synthetase [Ricinus communis, EEF33425.1]	binding
	4	367	387	DB948502	similar to DNA binding protein [Ricinus communis, EEF49890.1]	binding
**ACMV-mir-3-15***	2.5	372	394	DV448789	similar to 60S ribosomal protein L30 [Ricinus communis, EEF33410.1]	structural molecule activity
	3.5	387	410	DB946667	similar to 30S ribosomal protein S8 [Ricinus communis, EEF33949.1]	structural molecule activity
	3.5	40	63	DB929559	similar to sodium/hydrogen exchanger [Ricinus communis, EEF49734.1]	transporter activity
	3.5	416	438	DB951116	similar to short chain alcohol dehydrogenase [Ricinus communis, EEF32225.1]	binding
	4	123	146	FF381792	similar to 50S ribosomal protein L21[Ricinus communis, EEF3555]	structural molecule activity
	4	414	437	DB952421	similar to photosystem II reaction center protein M [Medicago truncatula, AES87815.1]	
	4	478	500	|DB949316	similar to gamma-tocopherol methyltransferase [Hevea brasiliensis, BAH10645.1]	catalytic activity
	4.5	257	280	DB924110	similar to catalytic [Ricinus communis, EEF51611.1]	binding
**ACMV-mir-3-16***	2.5	372	393	DV448789	similar to 60S ribosomal protein L30 [Ricinus communis, EEF33410.1]	structural molecule activity
	2.5	322	345	DB922103	similar to short-chain dehydrogenase [Ricinus communis, EEF51832.1]	binding
	3	122	145	FF381792	similar to 50S ribosomal protein L21[Ricinus communis, EEF3555]	structural molecule activity
	3.5	386	409	DB946667	similar to 30S ribosomal protein S8 [Ricinus communis, EEF33949.1]	structural molecule activity
	4	256	279	DB924110	similar to catalytic [Ricinus communis, EEF51611.1]	binding
	4.5	451	474	DB927765	similar to breast carcinoma amplified sequence [Ricinus communis, EEF49767.1]	
	5	304	327	DB954943	similar to stearoyl-acyl-carrier protein desaturase [Manihot esculenta]	binding
	5	240	263	DB950710	similar to transparent testa 12 protein [Ricinus communis, EEF30163.1]	transporter activity
	5	7	30	DB926288	similar to basic 7S globulin 2 precursor small subunit [Ricinus communis, EEF45325.1]	nutrient reservoir activity

**ACMV-mir-3-17***	2.5	118	141	DB921109	similar to 50S ribosomal protein L21 [Ricinus communis, EEF3555]	structural molecule activity
	3	321	344	DB922103	similar to short-chain dehydrogenase [Ricinus communis, EEF51832.1]	binding
	3.5	385	408	DB946667	similar to 30S ribosomal protein S8 [Ricinus communis, EEF33949.1]	structural molecule activity
	3.5	255	278	DB924110	similar to catalytic [Ricinus communis, EEF51611.1]	binding
	3.5	38	61	DB929559	similar to sodium/hydrogen exchanger [Ricinus communis, EEF49734.1]	transporter activity
	4	450	473	DB927765	similar to breast carcinoma amplified sequence [Ricinus communis, EEF49767.1]	
	4	474	497	DB950915	similar to uncharacterized protein Glycine max, XP_003556451.1]	
	4	172	195	DB944492	similar to DNA binding protein [Ricinus communis, EEF44141.1]	binding
	4.5	239	262	DB950710	similar to transparent testa 12 protein [Ricinus communis, EEF30163.1]	transporter activity
	4.5	552	575	DB928865	similar to electron transporter [Ricinus communis, EEF49572.1]	transporter activity
	5	297	320	DB929517	similar to ERD1 protein, chloroplast [Ricinus communis, EEF51657.1]	binding
	5	347	370	DB940623	similar to 60S ribosomal protein L22 [Ricinus communis, EEF39275.1]	structural molecule activity
**ACMV-mir-3-18***	2	118	139	DB921109	similar to 50S ribosomal protein L21 [Ricinus communis, EEF3555]	structural molecule activity
	3	450	471	DB927765	similar to breast carcinoma amplified sequence [Ricinus communis, EEF49767.1]	
	3.5	140	162	DB928298	similar to disulfide oxidoreductase [Ricinus communis, EEF32882.1]	binding
	3.5	243	265	DB933884	similar to diphosphomevalonate decarboxylase [Ricinus communis, EEF41203.1]	binding
	3.5	425	447	FF534999	similar to 2-deoxyglucose-6-phosphate phosphatase [Ricinus communis, EEF49975.1]	binding
	3.5	171	193	DB944492	similar to DNA binding protein [Ricinus communis, EEF44141.1]	binding
	3.5	141	163	DB932235	similar to polyadenylation factor subunit [Ricinus communis, EEF37441.1]	binding
	4	346	368	DB940623	similar to 60S ribosomal protein L22 [Ricinus communis, EEF39275.1]	binding
	4	338	359	CK641680	similar to short-chain dehydrogenase [Ricinus communis]	binding
	4.5	83	105	CK650969	similar to heat shock protein binding protein [Ricinus communis, EEF47017.1]	binding
	4.5	291	313	DB926022	similar to omega-3 fatty acid desaturase, chloroplast [Ricinus communis, P48619.1]	binding
**ACMV-mir-3-19***	2.5	552	571	DB928865	similar to electron transporter [Ricinus communis, EEF49572.1]	electron carrier activity
	3	346	366	DB940623	similar to 60S ribosomal protein L22 [Ricinus communis, EEF39275.1]	structural molecule activity
	3	336	357	CK641680	similar to short-chain dehydrogenase [Ricinus communis]	binding
	3.5	371	392	DB949918	similar to uncharacterized protein LOC100789970 [Glycine max, XP_003517375.1]	
	3.5	285	305	FG807027	similar to 2-deoxyglucose-6-phosphate phosphatase [Ricinus communis, EEF49975.1]	binding
	3.5	88	108	CK649175	similar to indole-3-acetic acid-induced protein ARG2 [Ricinus communis, EEF39286.1]	
	4	292	313	DB922202	similar to omega-3 fatty acid desaturase, chloroplast precursor [Ricinus communis, EEF34057.1]	catalytic activity
	4	379	399	DV445050	similar to nucleic acid binding protein [Ricinus communis, EEF35632.1]	binding

	4	483	503	DB934327	similar to kinase [Ricinus communis, EEF37837.1]	binding
**ACMV-mir-4-1**	4	294	317	DB929628	similar to actin related protein [Populus trichocarpa, EEE96159.1]	
	5	84	107	DB942321	similar to methionine sulfoxide reductase [Ricinus communis, EEF51626.1]	catalytic activity
	5	129	153	FG805909	similar to big map kinase/bmk [Ricinus communis, EEF36363.1]	binding
	5	148	169	DV457980	similar to signal transducer [Ricinus communis, EEF43930.1]	molecular transducer activity
	5	336	358	DB923899	similar to mannose-1-phosphate guanyltransferase [Ricinus communis, EEF44959.1]	catalytic activity
	5	538	560	FF535040	similar to cw7 protein [Ricinus communis, EEF52246.1]	
**ACMV-mir-4-2**	3.5	291	314	DB929628	similar to actin related protein [Populus trichocarpa, EEE96159.1]	
	4	172	195	DB928320	similar to R2R3 MYB transcription factor [Jatropha curcas, JF795918]	binding
	4.5	162	184	DV444240	similar to protein transporter [Ricinus communis, EEF28561.1]	
	5	462	486	DB944600	similar to glutamine synthetase I [Medicago truncatula, CAB63844.1]	binding
	5	467	489	DB953621	similar to map3k delta-1 protein kinase [Ricinus communis, EEF31491.1]	binding
	5	292	314	FF379768	similar to carboxy-lyase [Ricinus communis, EEF42942.1]	catalytic activity
**ACMV-mir-4-3**	3	171	194	DB928320	similar to R2R3 MYB transcription factor [Jatropha curcas, JF795918]	binding
	3.5	291	313	DB929628	similar to actin related protein [Populus trichocarpa, EEE96159.1]	
	4.5	467	488	DB953621	similar to map3k delta-1 protein kinase [Ricinus communis, EEF31491.1]	binding
	4.5	292	313	FF379768	similar to carboxy-lyase [Ricinus communis, EEF42942.1]	catalytic activity
	5	461	485	DB944600	similar to glutamine synthetase I [Medicago truncatula, CAB63844.1]	binding
	5	714	737	DV458407	similar to heme-binding protein [Ricinus communis, EEF51963.1]	electron carrier activity
**ACMV-mir-4-4**	3.5	461	483	DB944600	similar to glutamine synthetase I [Medicago truncatula, CAB63844.1]	binding
	4	582	604	DV455176	similar to receptor serine-threonine protein kinase [Ricinus communis, EEF37167.1]	binding
	4.5	252	273	DB944352	similar to ATP binding protein [Ricinus communis, EEF40790.1]	binding
**ACMV-mir-4-5**	3	30	49	DV451964	similar to 60S ribosomal protein L44 [Ricinus communis, EEF46746.1]	structural molecule activity
**ACMV-mir-4-6***	4.5	377	397	DV448964	similar to cytochrome P450 [Manihot esculenta, AAV97888.1]	electron carrier activity
	5	8	30	DR086636	heat shock factor protein HSF24-like [Vitis vinifera, CBI21429.3]	nucleic acid binding transcription factor activity
**ACMV-mir-4-7***	3	528	547	DB942747	similar to conserved hypothetical protein [Ricinus communis, EEF28583.1]	
	3.5	188	207	DB933048	similar to conserved hypothetical protein [Ricinus communis, EEF52310.1]	
	4	552	571	DV457422	similar to 60S ribosomal protein L21[Ricinus communis, EEF31216.1]	structural molecule activity
	4	280	299	CK648412	similar to transcription factor [Ricinus communis, EEF35860.1]	binding

**ACMV-mir-5-1**	1.5	113	132	DR086591	similar to translationally controlled tumor protein [Hevea brasiliensis, AFM77714.1]	binding
	1.5	511	530	CK647841	similar to casein kinase [Ricinus communis, EEF48084.1]	binding
**ACMV-mir-5-2**	1.5	113	132	DR086591	similar to translationally controlled tumor protein [Hevea brasiliensis, AFM77714.1]	binding
	1.5	511	530	CK647841	similar to casein kinase [Ricinus communis, EEF48084.1]	binding
**ACMV-mir-5-3**	2.5	112	131	DR086591	similar to translationally controlled tumor protein [Hevea brasiliensis, AFM77714.1]	binding
	4	115	138	DV445508	similar to RNA and export factor binding protein [Ricinus communis, EEF31023.1]	binding
**ACMV-mir-5-4**	3.5	2	25	DR088266	similar to protein binding protein [Ricinus communis, EEF35243.1]	
	3.5	382	405	DB934040	similar to FGGY carbohydrate kinase domain-containing protein [Vitis vinifera, CBI17791.3]	catalytic activity
	3.5	128	147	DR083744	similar to translationally controlled tumor protein [Hevea brasiliensis, AFM77714.1]	binding
	4.5	114	137	DV445508	similar to RNA and export factor binding protein [Ricinus communis, EEF31023.1]	binding
	4.5	254	276	DB924672	similar to WD-repeat protein [Ricinus communis, EEF44865.1]	
	5	70	92	DR086242	similar to thioredoxin h-type [Ricinus communis, EEF42213.1]	electron carrier activity
**ACMV-mir-5-5**	2.5	423	444	DB922799	similar to phopholipase d alpha [Ricinus communis, EEF44789.1]	binding
	3	545	565	DV454057	similar to vacuolar cation/proton exchanger 1a [Ricinus communis, EEF34279.1]	transporter activity
	3	113	134	DV445508	similar to RNA and export factor binding protein [Ricinus communis, EEF31023.1]	binding
	3	354	374	DB938778	similar to xyloglucan endotransglucosylase/hydrolase protein 22 [Ricinus communis, EEF29701.1]	catalytic activity
	3	70	89	DR086242	similar to thioredoxin h-type [Ricinus communis, EEF42213.1]	electron carrier activity
	3.5	294	315	CK647078	similar to AP2/ERF domain-containing transcription factor [Populus trichocarpa, EEE71425.1]	nucleic acid binding<br>transcription factor activity
	4	421	442	DB934347	similar to SIT4 phosphatase-associated family protein [Arabidopsis lyrata subsp. Lyrata, EFH65936.1]	
**ACMV-mir-5-6**	2.5	422	444	DB922799	similar to phopholipase d alpha [Ricinus communis, EEF44789.1]	binding
	3	545	565	DV454057	similar to vacuolar cation/proton exchanger 1a, [Ricinus communis, EEF34279.1]	transporter activity
	3	113	134	DV445508	similar to RNA and export factor binding protein [Ricinus communis, EEF31023.1]	binding
	3	354	374	DB938778	similar to xyloglucan endotransglucosylase/hydrolase protein 22 [Ricinus communis, EEF29701.1]	catalytic activity
	3	70	89	DR086242	similar to thioredoxin h-type [Ricinus communis, EEF42213.1]	electron carrier activity
	3.5	293	315	CK646147	similar to AP2/ERF domain-containing transcription factor [Populus trichocarpa, EEE71425.1]	nucleic acid binding<br>transcription factor activity

	3.5	220	243	CK644558	similar to nucleotide sugar epimerase [Cucumis sativus]	catalytic activity
	3.5	48	71	CK644942	similar to dtdp-glucose 4-6-dehydratase [Ricinus communis, EEF33919.1]	catalytic activity
	4	420	442	DB934347	similar to SIT4 phosphatase-associated family protein [Arabidopsis lyrata subsp. Lyrata, EFH65936.1]	
**ACMV-mir-5-7***	4	39	62	CK641566	similar to 14-3-3 protein [Ricinus communis, EEF48511.1]	binding
	4.5	430	453	CK643355	similar to pyruvate decarboxylase [Ricinus communis]	binding
	4.5	587	609	FF381959	similar to zinc finger protein [Ricinus communis, EEF40425.1]	binding
	5	458	481	DB942972	similar to similar to ribosomal protein L15 [Ricinus communis, EEF51579.1]	structural molecule activity
	5	199	223	DB930084	similar to pentatricopeptide repeat-containing protein [Ricinus communis, EEF46437.1]	binding
**ACMV-mir-5-8***	3	106	125	DB939328	similar to conserved hypothetical protein [Ricinus communis, EEF46231.1]	
	3.5	435	456	DV454886	similar to xyloglucan endotransglucosylase/hydrolase protein 22 precursor [Ricinus communis, EEF36165.1]	catalytic activity
	3.5	492	513	DV451674	similar to 14-3-3 protein [Ricinus communis, EEF48511.1]	binding
	3.5	280	302	DB954659	similar to groes chaperonin [Ricinus communis, EEF36369.1]	binding
	4	455	477	DV446664	similar to 60S ribosomal protein L13aA [Hevea brasiliensis, ADR71263.1]	structural molecule activity
	4.5	514	535	CK643668	similar to pyrophosphate-energized vacuolar membrane proton pump [Ricinus communis, EEF31615.1]	catalytic activity
	4.5	449	470	DT883569	similar to xyloglucan endotransglucosylase/hydrolase protein 22 precursor, [Ricinus communis, XP_002526224.1]	catalytic activity
	5	194	215	DB935464	similar to calcium-activated outward-rectifying potassium channel [Ricinus communis, EEF47173.1]	binding
	5	140	161	DB938331	similar to oxygen-evolving enhancer protein 2, chloroplast precursor, [Ricinus communis,EEF40070.1]	binding
**ACMV-mir-5-9***	2.5	194	213	DB935464	similar to calcium-activated outward-rectifying potassium channel [Ricinus communis, EEF47173.1]	binding
	3	454	475	DV446664	similar to 60S ribosomal protein L13aA [Hevea brasiliensis, ADR71263.1]	structural molecule activity
	3	379	400	DB950958	similar to U2 snrnp auxiliary factor, small subunit [Ricinus communis, EEF33789.1]	binding
	4	101	123	FG805214	similar to ATP binding protein [Ricinus communis, EEF37841.1]	binding
	4	245	267	DB940960	similar to fructose-bisphosphate aldolase [Ricinus communis, EEF49496.1]	catalytic activity
	5	227	250	CK652240	similar to receptor protein kinase [Ricinus communis, EEF50538.1]	binding
	5	142	164	DB920171	similar to serine hydroxymethyltransferase [Ricinus communis, EEF39657.1]	catalytic activity
**ACMV-mir-5-10***	3	191	212	DB935464	similar to calcium-activated outward-rectifying potassium channel [Ricinus communis, EEF47173.1]	binding
	3	454	474	DV446664	similar to 60S ribosomal protein L13aA [Hevea brasiliensis, ADR71263.1]	structural molecule activity
	4	227	249	CK652240	similar to receptor protein kinase [Ricinus communis, EEF50538.1]	binding
	4.5	126	147	DV448457	similar to 2-oxoisovalerate dehydrogenase [Ricinus communis, EEF46052.1]	catalytic activity
	5	222	244	DB922396	similar to delta12-fatty acid desaturase [Jatropha curcas, ABA41034.1]	catalytic activity

	5	222	244	DB934052	similar to omega-6 fatty acid desaturase [Hevea brasiliensis]	catalytic activity
	5	188	210	DV453091	similar to mitochondrial acidic protein MAM33, mitochondrial precursor [Ricinus communis, EEF32518.1]	
	5	287	309	DV453887	similar to aldo/keto reductase AKR [Manihot esculenta, AAX84672.1]	catalytic activity
	5	483	505	DB955293	similar to polyadenylate-binding protein [Ricinus communis, EEF43658.1]	binding
**ACMV-mir-5-11***	3	107	129	DB921202	similar to reticulon-3 [Ricinus communis, EEF43605.1]	
	3	388	407	DB940119	similar to serine/threonine-protein kinase PBS1 [Ricinus communis, EEF43245.1]	binding
	3.5	313	335	DB944934	similar to photosystem I P700 apoprotein A1 [Oryza australiensis, ADD62970.1]	binding
	4	173	195	DB940672	similar to carbonic anhydrase [Ricinus communis, EEF37661.1]	binding
	5	111	131	DB937239	similar to zinc finger CCCH domain-containing protein 6 [Vitis vinifera, XP_002277913.1]	binding
**ACMV-mir-5-12***	2	105	128	DB921202	similar to reticulon-3 [Ricinus communis, EEF43605.1]	
	3.5	7	30	DB929039	similar to hydroxycinnamoyl-Coenzyme A shikimate/quinate hydroxycinnamoyltransferase [Vitis vinifera]	catalytic activity
	4.5	462	484	DB944406	similar to 40S ribosomal protein S15 [Ricinus communis, EEF45178.1]	structural molecule activity
	4.5	180	203	DB922561	similar to RNA binding protein [Ricinus communis, EEF29218.1]	binding
	4.5	55	78	DB954098	similar to succinyl-CoA synthetase beta chain [Ricinus communis, EEF32531.1]	binding
**ACMV-mir-5-13***	1.5	104	127	DB921202	similar to reticulon-3 [Ricinus communis, EEF43605.1]	
	4	6	29	DB929039	similar to hydroxycinnamoyl-Coenzyme A shikimate/quinate hydroxycinnamoyltransferase [Vitis vinifera], predicted	catalytic activity
	4	208	231	DV458190	similar to RNA binding protein [Ricinus communis, EEF29218.1]	binding
	4	462	483	DB944406	similar to 40S ribosomal protein S15 [Ricinus communis, EEF45178.1]	structural molecule activity
	4.5	18	41	DB926794	similar to sulfate transporter 3.1-like [Vitis vinifera]	transporter activity
	4.5	282	304	DB922855	similar to pentatricopeptide repeat-containing protein [Ricinus communis, EEF35728.1]	binding
	4.5	139	162	GH612006	similar to calmodulin binding protein [Ricinus communis, EEF48083.1]	binding
	5	216	239	DB946443	similar to serine/threonine protein kinase [Ricinus communis, EEF38017.1]	binding
	5	488	511	DB946291	similar to signal recognition particle 14 kDa protein [Ricinus communis, EEF31797.1]	binding
**ACMV-mir-5-14***	1	104	126	DB921202	similar to reticulon-3 [Ricinus communis, EEF43605.1]	
	4	6	28	DB929039	similar to hydroxycinnamoyl-coenzyme A shikimate/quinate hydroxycinnamoyltransferase [Vitis vinifera]	catalytic activity
	4	260	282	DB928965	similar to RNA binding protein [Ricinus communis, EEF29218.1]	binding
	4	18	40	DB926794	similar to sulfate transporter 3.1-like [Vitis vinifera]	transporter activity
	4	282	303	DB922855	similar to pentatricopeptide repeat-containing protein [Ricinus communis, EEF35728.1]	binding
	4.5	218	240	DB949658	similar to serine/threonine protein kinase [Ricinus communis, EEF38017.1]	binding

	5	19	41	DB923718	similar to scarecrow-like protein 3-like [Vitis vinifera, CAN60488.1]	nucleic acid binding transcription factor activity
	5	7	29	DB924182	similar to disulfide interchange protein dsbD precursor [Ricinus communis, EEF31339.1]	catalytic activity
**ACMV-mir-5-15***	1	104	126	DB921202	similar to reticulon-3 [Ricinus communis, EEF43605.1]	
	4	207	230	DV458190	similar to RNA binding protein [Ricinus communis, EEF29218.1]	binding
	4	720	743	FF536237	similar to asparagine synthetase [Ricinus communis, EEF31122.1]	binding
	4	6	28	DB929039	similar to hydroxycinnamoyl-Coenzyme A shikimate/quinate hydroxycinnamoyltransferase [Vitis vinifera]	catalytic activity
	4	18	40	DB926794	similar to sulfate transporter 3.1-like [Vitis vinifera]	transporter activity
	4.5	217	240	DB949658	similar to serine/threonine protein kinase [Ricinus communis, EEF38017.1]	binding
	4.5	487	510	DB946291	similar to signal recognition particle 14 kDa protein [Ricinus communis, EEF31797.1]	binding
	4.5	476	499	DV456500	similar to 14-3-3 protein [Ricinus communis, EEF48599.1]	binding
	5	18	41	DB923718	similar to scarecrow-like protein 3-like [Vitis vinifera, CAN60488.1]	nucleic acid binding transcription factor activity
	5	6	29	DB924182	similar to disulfide interchange protein dsbD precursor [Ricinus communis, EEF31339.1]	catalytic activity
**ACMV-mir-6-1**	2.5	470	489	DB941578	similar to conserved hypothetical protein [Ricinus communis, EEF28668.1]	
	3	41	61	FG805430	similar to heat shock protein hsp82 [Oryza sativa, CAA78738.1]	binding
	3.5	144	163	DB946929	similar to glycerate dehydrogenase [Ricinus communis, EEF46448.1]	binding
	4	107	127	DV454101	similar to leucine-rich repeat containing protein [Ricinus communis, EEF48942.1]	binding
	4	132	152	CK652363	similar to mitogen activated protein kinase kinase, mapkk2 [Ricinus communis, EEF48158.1]	binding
	4	215	235	DB937274	similar to vesicle-associated membrane protein[Ricinus communis, EEF30603.1]	structural molecule activity
	4.5	268	288	FF536554	similar to serine-threonine protein kinase, plant-type [Ricinus communis, EEF38944.1]	binding
	4.5	200	220	FF380232	similar to rer1 protein [Ricinus communis, EEF49424.1]	
	4.5	260	280	DB922715	similar to ADP-ribosylation factor GTPase-activating protein AGD14-like [Glycine max], predicted	enzyme regulator activity
	4.5	256	276	DB938113	similar to HIV-1 rev binding protein, hrbl [Ricinus communis, EEF31425.1]	enzyme regulator activity
**ACMV-mir-6-2**	4	249	271	FF380695	similar to 40S ribosomal protein S9 [Ricinus communis, EEF52159.1]	structural molecule activity
	4	130	151	CK652363	similar to mitogen activated protein kinase kinase, mapkk2 [Ricinus communis, EEF48158.1]	binding
	4	212	234	DB937274	similar to vesicle-associated membrane protein [Ricinus communis, EEF30603.1]	structural molecule activity
	4.5	196	219	FF380232	similar to rer1 protein [Ricinus communis, EEF49424.1]	
	4.5	266	287	FF536554	similar to serine-threonine protein kinase, plant-type [Ricinus communis, EEF38944.1]	binding

	4.5	260	282	DV447145	similar to DNA binding protein [Ricinus communis, EEF52387.1]	binding
	5	159	182	DV444080	similar to ubiquitin-activating enzyme E1b, [Ricinus communis, EEF49457.1]	binding
	5	316	339	DV442611	similar to thylakoid lumenal 16.5 kDa protein, chloroplast precursor [Ricinus communis, EEF35210.1]	
**ACMV-mir-6-3**	3	61	82	FF380931	similar to beta-1,3-glucuronyltransferase [Ricinus communis, EEF52188.1]	catalytic activity
	3	119	138	DV441358	similar to rer1 protein [Ricinus communis, EEF40831.1]	
	3	315	336	DV442611	similar to thylakoid lumenal 16.5 kDa protein, chloroplast precursor [Ricinus communis, EEF35210.1]	
	3.5	158	179	DV444080	similar to ubiquitin-activating enzyme E1b, [Ricinus communis, EEF49457.1]	binding
	3.5	20	42	DV440887	similar to nutrient reservoir [Ricinus communis, EEF31271.1]	nutrient reservoir activity
	3.5	246	265	DV446004	similar to protein binding protein [Ricinus communis, EEF34752.1]	
	4	335	358	DV454973	similar to nonspecific lipid-transfer protein precursor [Ricinus communis, EEF33672.1]	binding
	4.5	56	78	DV442154	similar to exportin-7 [Ricinus communis, EEF36832.1]	transporter activity
	4.5	145	168	FG804994	similar to ATP-binding cassette transporter [Ricinus communis, EEF35082.1]	binding
**ACMV-mir-6-4**	3.5	242	261	DV445947	similar to ribosome biogenesis protein tsr1 [Ricinus communis, EEF52849.1]	binding
	4.5	59	79	FF535000	similar to phytanoyl-CoA dioxygenase domain containing [Ricinus communis, EEF36371.1]	catalytic activity
	5	309	331	DB947351	similar to serine-threonine protein kinase, plant-type [Ricinus communis, EEF49172.1]	binding
	5	196	215	DB928369	similar to protein binding protein [Ricinus communis, EEF31511.1]	
	5	637	657	FF536517	similar to ecotropic viral integration site [Ricinus communis, EEF36381.1]	enzyme regulator activity
**ACMV-mir-6-5**	3.5	194	214	DB948863	similar to acetylglucosaminyltransferase [Ricinus communis, EEF44495.1]	catalytic activity
	3.5	230	252	DB930065	similar to dead box ATP-dependent RNA helicase [Ricinus communis, EEF33161.1]	binding
	3.5	15	37	DV458200	similar to ATP-binding cassette transporter [Ricinus communis, EEF49231.1]	binding
	4.5	544	567	DB951256	similar to acetylglucosaminyltransferase [Ricinus communis, EEF35190.1]	catalytic activity
	5	224	247	DB941011	similar to serine carboxypeptidase [Ricinus communis, EEF32785.1]	catalytic activity
	5	398	421	FF535408	similar to pentatricopeptide repeat-containing protein [Ricinus communis, EEF29801.1]	binding
	5	167	188	DR085866	similar to 30S ribosomal protein S5 [Ricinus communis, EEF50844.1]	structural molecule activity
**ACMV-mir-6-6**	4	219	240	DV447596	similar conserved hypothetical protein [Ricinus communis, EEF42682.1]	
	4.5	226	249	CK650688	similar to casein kinase [Ricinus communis, EEF41046.1]	binding
**ACMV-mir-6-7***	2.5	454	473	DB951887	similar to nucleic acid binding protein [Ricinus communis, EEF48069.1]	binding
	3	255	274	DB953977	similar to ATP-dependent clp protease [Ricinus communis, EEF51704.1]	binding

	4	15	34	DB935590	similar to voltage-dependent anion-selective channel [Ricinus communis, EEF45012.1]	transporter activity
	4	263	282	DV445604	similar to ubiquitin-protein ligase[Ricinus communis, EEF36752.1]	catalytic activity
	4	105	124	DV450629	similar to homeobox protein [Ricinus communis, EEF42201.1]	binding
	4	344	363	DB922543	similar to proteasome maturation protein [Ricinus communis, EEF36388.1]	
	4	14	33	DB948941	similar to queuine tRNA-ribosyltransferase [Ricinus communis, EEF35976.1]	binding
	4.5	45	64	DV445687	similar to short-chain dehydrogenase [Ricinus communis, EEF48252.1]	binding
**ACMV-mir-6-8***	2.5	250	271	DB953977	similar to ATP-dependent clp protease [Ricinus communis, EEF51704.1]	binding
	3	42	61	DV445687	similar to short-chain dehydrogenase [Ricinus communis, EEF48252.1]	binding
	4	64	87	DV448290	similar to glycine-rich RNA-binding protein [Ricinus communis,EEF29005.1]	binding
	5	304	324	DR086922	similar to tubulin beta chain [Ricinus communis, EEF39168.1]	structural molecule activity
**ACMV-mir-6-9***	3	131	154	DV445431	similar to zinc metalloprotease [Ricinus communis, EEF43712.1]	catalytic activity
	3.5	498	517	DB951580	similar to pectin acetylesterase [Ricinus communis, EEF42516.1]	catalytic activity
	4.5	20	43	DV450217	similar to protein yippee-like At5g53940-like [Glycine max, XP_003543228.1]	binding
	4.5	442	464	DB951887	similar to nucleic acid binding protein [Ricinus communis, EEF48069.1]	binding
**ACMV-mir-6-10***	3.5	661	681	DV443352	similar to chaperonin containing t-complex protein 1, alpha subunit, tcpa, [Ricinus communis, EEF37754.1]	binding
	4	67	88	DV447941	similar to protein yippee-like At5g53940-like [Glycine max, XP_003543228.1]	binding
	4.5	280	302	DB933827	similar to FK506-binding protein [Ricinus communis, EEF43127.1]	catalytic activity
	5	115	138	DB935466	similar to calreticulin [Ricinus communis, P93508.1]	binding
	5	271	294	DB954431	similar to catalytic [Ricinus communis, EEF48203.1]	binding
**ACMV-mir-6-11***	3.5	661	680	DV443352	similar to chaperonin containing t-complex protein 1, alpha subunit, tcpa, [Ricinus communis, EEF37754.1]	binding
	4	116	137	DB938103	similar to calreticulin [Ricinus communis, P93508.1]	binding
	4	67	87	DV447941	similar to protein yippee-like At5g53940-like [Glycine max, XP_003543228.1]	binding
	4.5	273	294	DB931218	similar to serine/threonine-protein kinase PBS1 [Ricinus communis, EEF34595.1]	binding
	5	266	285	DB948416	similar to regulatory protein NPR1 [Ricinus communis, EEF48081.1]	
**ACMV-mir-6-13***	3	297	316	DB929462	similar to 2,3-bisphosphoglycerate-independent phosphoglycerate mutase [Ricinus communis, EEF42299.1]	binding
	4	247	267	CK643935	similar to coatomer beta subunit [Ricinus communis]	structural molecule activity
	4.5	201	222	CK646843	similar to chitinase [Ricinus communis, EEF46716.1]	binding
**ACMV-mir-6-14***	2.5	246	265	CK643935	similar to coatomer beta subunit [Ricinus communis]	structural molecule activity

	3.5	295	314	DB929462	similar to 2,3-bisphosphoglycerate-independent phosphoglycerate mutase [Ricinus communis, EEF42299.1]	binding
	4	401	420	FF534371	similar to glutathione s-transferase [Ricinus communis, EEF29553.1]	catalytic activity
	4.5	36	56	DV454879	similar to poly-A binding protein [Ricinus communis, EEF30719.1]	binding
	5	238	258	DB937598	similar to DNA-directed RNA polymerase subunit [Ricinus communis, EEF46877.1]	binding
**ACMV-mir-6-15***	2.5	234	256	DB937598	similar to DNA-directed RNA polymerase subunit [Ricinus communis, EEF46877.1]	binding
	3.5	74	97	DB938479	similar to poly-A binding protein [Ricinus communis, EEF30719.1]	binding
	4.5	398	421	FF381012	similar to protein phosphatase 2a, regulatory subunit [Ricinus communis, EEF42877.1]	enzyme regulator activity
	4.5	307	330	DV455349	similar to chloroplast photosystem II 10 kDa polypeptide [Jatropha curcas, ADB93062.1]	
**ACMV-mir-6-16***	2.5	31	52	DV454879	similar to poly-A binding protein [Ricinus communis, EEF30719.1]	binding
	3	398	419	FF381012	similar to protein phosphatase 2a, regulatory subunit [Ricinus communis, EEF42877.1]	enzyme regulator activity
	4.5	476	500	DB929843	similar to heterogeneous nuclear ribonucleoprotein [Ricinus communis, EEF48904.1]	binding
	4.5	628	649	FF380371	similar to glucose-1-phosphate adenylyltransferase [Ricinus communis, EEF45359.1]	catalytic activity
	5	600	622	DV458828	similar to protein phosphatase 2c [Ricinus communis, EEF42042.1]	binding
**ACMV-mir-6-17***	3.5	628	648	FF380371	similar to glucose-1-phosphate adenylyltransferase [Ricinus communis, EEF45359.1]	catalytic activity
	3.5	398	418	FF381012	similar to protein phosphatase 2a, regulatory subunit [Ricinus communis, EEF42877.1]	enzyme regulator activity
	3.5	74	94	DB938479	similar to poly-A binding protein [Ricinus communis, EEF30719.1]	binding
	3.5	478	499	DB929843	similar to heterogeneous nuclear ribonucleoprotein [Ricinus communis, EEF48904.1]	binding
	5	56	75	FF536776	similar to ubiquitin extension protein [Jatropha curcas, ACU81074.1]	structural molecule activity
**ACMV-mir-7-1**	3	548	567	DB937072	similar to catalytic [Ricinus communis, EEF34353.1]	binding
	4	342	363	DV456944	similar to auxin-induced protein 5NG4 [Ricinus communis, EEF48375.1]	
	4	80	101	DV448004	similar to protein binding protein [Ricinus communis, EEF43079.1]	
	4.5	456	477	DR084750	similar to tubulin beta chain [Ricinus communis, EEF39167.1]	structural molecule activity
	4.5	1	22	DB925029	similar to DNA binding protein [Ricinus communis, EEF50395.1]	binding
**ACMV-mir-7-2***	3	307	326	DB954930	similar to casein kinase [Ricinus communis, EEF45884.1]	binding
	3.5	139	161	DB953761	similar to mak [Ricinus communis, EEF42474.1]	binding
	4	32	51	CK648460	similar to speckle-type POZ protein [Ricinus communis, EEF45411.1]	
	4	345	367	FG805232	similar to NADH-cytochrome B5 reductase [Ricinus communis, EEF34822.1]	catalytic activity
	4.5	305	327	BM259935	similar to CDK [Ricinus communis, EEF52923.1]	binding

	5	229	251	CK640931	similar to chalcone synthase [Populus trichocarpa]	catalytic activity
	5	407	429	FF536260	similar to 40S ribosomal protein S23 [Ricinus communis]	structural molecule activity
**ACMV-mir-7-3***	3.5	30	49	CK648460	similar to speckle-type POZ protein [Ricinus communis, EEF45411.1]	
	3.5	344	364	DB937516	similar to zinc finger protein, [Ricinus communis, EEF42871.1]	binding
	3.5	229	249	CK640931	similar to chalcone synthase [Populus trichocarpa]	catalytic activity
	3.5	139	159	DB953761	similar to mak [Ricinus communis, EEF42474.1]	binding
	3.5	345	365	FG805232	similar to NADH-cytochrome B5 reductase [Ricinus communis, EEF34822.1]	catalytic activity
	4	386	406	CK644983	similar to Beta-1,3-galactosyltransferase sqv-2 [Ricinus communis]	catalytic activity
	4	697	717	FF380976	similar to monoglyceride lipase-like [Glycine max, XP_003522717.1]	catalytic activity
	4	303	322	DB941671	similar to S-adenosylmethionine-dependent methyltransferase [Ricinus communis, EEF39951.1]	catalytic activity
	4	482	502	DB938141	similar to hydrolase, hydrolyzing O-glycosyl compounds [Ricinus communis, EEF30440.1]	binding
	4.5	328	348	DV441169	similar to argininosuccinate synthase [Ricinus communis, EEF41199.1]	binding
	4.5	395	415	CK644588	similar to structural molecule [Ricinus communis]	structural molecule activity
	4.5	59	79	DB926338	similar to calmodulin-binding heat-shock protein [Ricinus communis, EEF30464.1]	catalytic activity
**EACMV-UG-mir-1-1**	2.5	329	349	DV453191	similar to density-regulated protein [Ricinus communis, EEF48071.1]	binding
	3	104	127	DB951239	similar to phenazine biosynthesis protein [Ricinus communis, EEF41730.1]	catalytic activity
	3	507	526	DB926096	similar to glutathione S-transferase GST 23 [Glycine max, AAG34813.1]	catalytic activity
	3.5	270	293	FF535557	similar to sentrin/sumo-specific protease [Ricinus communis, EEF45541.1]	catalytic activity
	4	174	197	CK642230	similar to sucrose synthase [Manihot esculenta, ABD96570.1]	catalytic activity
	4	122	144	DV451570	similar to AMP dependent CoA ligase [Ricinus communis, EEF49284.1]	catalytic activity
	4	379	401	DB946651	similar to radical sam protein [Ricinus communis, EEF40626.1]	catalytic activity
	4	207	229	FF381020	similar to photosystem II reaction center protein K [Medicago truncatula, AES77051.1]	
	4	407	429	CK642739	similar to 4-Coumarate:CoA ligase [Populus trichocarpa]	catalytic activity
	4.5	339	362	DV449149	similar to 2-deoxyglucose-6-phosphate phosphatase [Ricinus communis, EEF49975.1]	binding
	5	99	122	CK644358	similar to amino acid transporter [Ricinus communis]	transporter activity
	5	191	214	DV444267	similar to peptidyl-prolyl cis-trans isomerase [Ricinus communis, EEF28526.1]	catalytic activity
**EACMV-UG-mir-1-2**	3.5	73	95	CK644982	similar to oxysterol-binding protein-related protein 3C [Vitis vinifera]	binding
	3.5	379	399	DB946651	similar to radical sam protein [Ricinus communis, EEF40626.1]	catalytic activity
	4	269	291	FF535557	similar to sentrin/sumo-specific protease [Ricinus communis, EEF45541.1]	catalytic activity
	4	190	212	DV444267	similar to peptidyl-prolyl cis-trans isomerase [Ricinus communis, EEF28526.1]	catalytic activity
	4.5	17	40	DV442611	similar to thylakoid lumenal 16.5 kDa protein, chloroplast precursor [Ricinus communis, EEF35210.1]	

	5	337	360	DV449149	similar to similar to 2-deoxyglucose-6-phosphate phosphatase [Ricinus communis, EEF49975.1]	binding
**EACMV-UG-mir-1-3**	3.5	73	95	CK644982	similar to oxysterol-binding protein-related protein 3C [Vitis vinifer]	binding
	3.5	379	399	DB946651	similar to radical sam protein [Ricinus communis, EEF40626.1]	catalytic activity
	4	269	291	FF535557	similar to sentrin/sumo-specific protease [Ricinus communis, EEF45541.1]	catalytic activity
	4	190	212	DV444267	similar to peptidyl-prolyl cis-trans isomerase [Ricinus communis, EEF28526.1]	catalytic activity
	4.5	18	40	DV442611	similar to thylakoid lumenal 16.5 kDa protein, chloroplast precursor [Ricinus communis, EEF35210.1]	
	4.5	814	836	FF536597	similar to Rab1 [Hevea brasiliensis, ADL59582.1]	binding
	5	338	360	DV449149	similar to 2-deoxyglucose-6-phosphate phosphatase [Ricinus communis, EEF49975.1]	binding
**EACMV-UG-mir-1-4**	3	18	39	DV442611	similar to thylakoid lumenal 16.5 kDa protein, chloroplast precursor [Ricinus communis, EEF35210.1]	
	3	379	398	DB946651	similar to radical sam protein [Ricinus communis, EEF40626.1]	catalytic activity
	4	73	94	CK644982	similar to oxysterol-binding protein-related protein 3C [Vitis vinifer]	binding
	4	98	119	CK644358	similar to amino acid transporter [Ricinus communis]	transporter activity
	4	269	290	FF535557	similar to sentrin/sumo-specific protease [Ricinus communis, EEF45541.1]	catalytic activity
	4	5	24	FF380439	similar to multidrug resistance pump [Ricinus communis, EEF43879.1]	transporter activity
	4.5	17	38	BM260106	similar to 60S ribosomal protein L23a [Ricinus communis, EEF31274.1]	structural molecule activity
	4.5	190	211	DV444267	similar to peptidyl-prolyl cis-trans isomerase [Ricinus communis, EEF28526.1]	catalytic activity
	4.5	78	99	DB927321	similar to catalytic [Ricinus communis, B9SQI7.1]	binding
	4.5	461	480	FF380571	similar to ATP binding protein [Ricinus communis, EEF52386.1]	binding
	5	404	425	DB941217	similar to coproporphyrinogen III oxidase [Ricinus communis, EEF43783.1]	catalytic activity
	5	327	348	DB927600	similar to stearoyl-acyl-carrier protein desaturase [Ricinus communis, ACG59949.1]	binding
**EACMV-UG-mir-1-5**	3	16	38	DV442611	similar to thylakoid lumenal 16.5 kDa protein, chloroplast precursor [Ricinus communis, EEF35210.1]	
	3	71	93	CK644982	similar to oxysterol-binding protein-related protein 3C [Vitis vinifer]	binding
	3	98	118	CK644358	similar to amino acid transporter [Ricinus communis]	transporter activity
	3.5	17	37	BM260106	50S ribosomal protein L29, chloroplast precursor, putative [Ricinus communis]	structural molecule activity
	3.5	78	98	DB927321	similar to catalytic [Ricinus communis, B9SQI7.1]	binding
	4	397	419	DB951269	similar to coproporphyrinogen III oxidase [Ricinus communis, EEF43783.1]	catalytic activity
	5	87	109	DB931450	similar to RNA-binding protein [Ricinus communis, EEF42723.1]	binding
	5	718	740	FF536261	similar to nuclear acid binding protein [Ricinus communis, EEF43886.1]	binding
**EACMV-UG-mir-1-6**	2.5	71	92	CK644982	similar to oxysterol-binding protein-related protein 3C [Vitis vinifer]	binding
	3	16	37	DV442611	similar to thylakoid lumenal 16.5 kDa protein, chloroplast precursor, [Ricinus communis, EEF35210.1]	
	4	87	108	DB931450	similar to RNA-binding protein [Ricinus communis, EEF42723.1]	binding

**EACMV-UG-mir-1-7**	3	16	36	DV442611	similar to thylakoid lumenal 16.5 kDa protein, chloroplast precursor [Ricinus communis, EEF35210.1]	
	4	347	366	DB946990	similar to lupus la ribonucleoprotein, [Ricinus communis, EEF33296.1]	binding
	4	666	686	DV445085	similar to carbonyl reductase [Ricinus communis, EEF50490.1]	catalytic activity
	4	587	608	DV447321	similar to r2r3-myb transcription factor [Ricinus communis, EEF31285.1]	binding
	4.5	50	71	DB929615	similar to microsomal signal peptidase 25 kD subunit [Ricinus communis, EF35484.1	catalytic activity
	5	614	635	FF381760	similar to transferase, transferring glycosyl groups [Ricinus communis, EEF41222.1]	catalytic activity
**EACMV-UG-mir-1-8**	3	410	431	DV441041	similar to carbonyl reductase [Ricinus communis, EEF50490.1]	catalytic activity
	3.5	346	365	DB946990	similar to lupus la ribonucleoprotein [Ricinus communis, EEF33296.1]	binding
	3.5	357	379	DV453453	similar to r2r3-myb transcription factor [Ricinus communis, EEF31285.1]	binding
	3.5	718	737	FF536261	similar to nuclear acid binding protein [Ricinus communis, EEF43886.1]	binding
	4.5	535	555	DR087887	similar to RNA polymerase-associated protein LEO1 [Ricinus communis, EEF47773.1]	binding
	5	9	31	FG806619	similar to NADH-ubiquinone oxidoreductase 1, chain [Ricinus communis, EEF50873.1]	binding
	5	1	23	DB932495	similar to catalytic [Ricinus communis, B9SQI7.1]	binding
**EACMV-UG-mir-1-9**	3	328	347	DB928490	similar to glyceraldehyde 3-phosphate dehydrogenase [Ricinus communis, EEF49293.1]	binding
	3.5	534	553	DR087887	similar to RNA polymerase-associated protein LEO1 [Ricinus communis, EEF47773.1]	binding
	3.5	637	656	CK652148	similar to elongation factor 1-alpha [Ricinus communis, EEF44205.1]	binding
	4	567	586	FF535038	similar to DNA binding protein [Ricinus communis, EEF43744.1]	binding
	4	177	196	CK648753	similar to nucleic acid binding protein [Ricinus communis, EEF35929.1]	binding
	4	318	337	DB922088	similar to ribulose bisphosphate carboxylase/oxygenase activase 1, chloroplast precursor [Ricinus communis, EEF38130.1]	binding
	4	10	29	FG806619	similar to NADH-ubiquinone oxidoreductase 1, chain [Ricinus communis, EEF50873.1]	binding
	4.5	344	363	DB946990	similar to lupus la ribonucleoprotein, [Ricinus communis, EEF33296.1]	binding
	4.5	495	514	DB940432	similar to 40S ribosomal protein S3a [Ricinus communis, EEF45248.1]	structural molecule activity
**EACMV-UG-mir-1-10**	3.5	165	185	BI325178	similar to zinc finger protein [Ricinus communis, EEF40424.1]	binding
	3.5	325	346	CK644202	similar to ubiquitin-protein ligase[Ricinus communis]	catalytic activity
	4	209	231	FG805332	similar to heterogeneous nuclear ribonucleoprotein A1 [Ricinus communis, EEF40297.1]	binding
	5	105	127	DR085496	similar to chitinase [Ricinus communis, EEF46716.1]	binding
**EACMV-UG-mir-1-11**	3	209	228	FG805332	similar to heterogeneous nuclear ribonucleoprotein A1 [Ricinus communis, EEF40297.1]	binding
	3.5	79	98	CK649514	similar to cyanohydrin UDP-glucosyltransferase UGT85K4 [Manihot esculenta, AEO45781.1]	catalytic activity

	3.5	127	146	DV454128	similar to UDP-glucuronosyltransferase [Ricinus communis, EEF29506.1]	catalytic activity
	4	645	664	FF536729	similar to protein MSP1[Ricinus communis, EEF30282.1]	binding
	4	519	538	DB929628	similar to actin related protein [Populus trichocarpa, EEE96159.1]	
	4.5	163	182	BI325178	similar to zinc finger protein [Ricinus communis,EEF40424.1]	binding
**EACMV-UG-mir-1-12***	3.5	249	271	GR422139	similar to dual specificity phosphatase Cdc25 [Ricinus communis, EEF45101.1]	binding
	3.5	146	167	DV451780	similar to defective in cullin neddylation protein [Ricinus communis, EEF35070.1]	binding
	4	462	484	DB954094	similar to receptor serine/threonine kinase [Ricinus communis, EEF48962.1]	binding
	4	429	452	DB948966	similar to aspartyl-tRNA synthetase [Ricinus communis, EEF48129.1]	binding
	4.5	226	249	DB939746	similar to 40S ribosomal protein S26 [Ricinus communis, EEF41563.1	structural molecule activity
**EACMV-UG-mir-1-13***	3	249	270	GR422139	similar to dual specificity phosphatase Cdc25 [Ricinus communis, EEF45101.1]	catalytic activity
	3.5	462	483	DB954094	similar to receptor serine/threonine kinase [Ricinus communis, EEF48962.1]	binding
	4	24	46	FF381798	similar to regulatory-associated protein of tor 1-like [Glycine max, XP_003533671.1]	
	4.5	76	99	CK642844	similar to protein fluG [Ricinus communis, EEF42861.1]	binding
**EACMV-UG-mir-1-14***	2.5	249	269	GR422139	similar to dual specificity phosphatase Cdc25 [Ricinus communis, EEF45101.1]	catalytic activity
	3.5	22	45	FF381798	similar to regulatory-associated protein of tor 1-like [Glycine max, XP_003533671.1]	
	5	348	370	DV446218	similar to NAD dependent epimerase/dehydratase [Ricinus communis, EEF51074.1]	binding
	5	165	188	DB923899	similar to mannose-1-phosphate guanyltransferase [Ricinus communis, EEF44959.1]	catalytic activity
**EACMV-UG-mir-1-15***	2.5	250	269	GR422139	similar to dual specificity phosphatase Cdc25 [Ricinus communis, EEF45101.1]	catalytic activity
	3.5	463	482	DB954094	similar to receptor serine/threonine kinase [Ricinus communis, EEF48962.1]	binding
	4	363	382	FF380750	similar to ccaat-binding transcription factor [Ricinus communis, EEF45343.1]	binding
**EACMV-UG-mir-1-16***	3	249	268	GR422139	similar to dual specificity phosphatase Cdc25 [Ricinus communis, EEF45101.1]	catalytic activity
	4	21	44	FF381798	similar to regulatory-associated protein of tor 1-like [Glycine max, XP_003533671.1]	
	4	267	290	BI325147	similar to 40S ribosomal protein S26 [Ricinus communis, EEF42086.1]	structural molecule activity
	4	368	388	DB944961	similar to NAD dependent epimerase/dehydratase [Ricinus communis, EEF51074.1]	binding
	4	9	30	FG807188	similar to cytoplasmic dynein light chain [Ricinus communis, EEF45562.1]	catalytic activity
	4.5	132	155	CK645440	similar to chromo domain protein [Ricinus communis]	binding
	5	165	187	DB923899	similar to mannose-1-phosphate guanyltransferase [Ricinus communis, EEF44959.1]	catalytic activity
	5	379	402	DB929051	similar to potassium transporter [Ricinus communis, EEF44904.1]	transporter activity

	5	24	48	FG805555	similar to indole-3-acetic acid-induced protein ARG2 [Ricinus communis, EEF39286.1]	
**EACMV-UG-mir-1-17***	4	103	122	CK644092	similar to cytochrome P450 [Citrus sinensis]	electron carrier activity
	4.5	364	385	DB922409	similar to superoxide dismutase [fe] [Ricinus communis, EEF41460.1]	binding
	4.5	18	41	FF381798	similar to regulatory-associated protein of tor 1-like [Glycine max, XP_003533671.1]	
	4.5	330	353	FF381021	similar to ATP-dependent Clp protease proteolytic subunit [Ricinus communis, EEF34901.1]	binding
	4.5	320	340	CK644406	similar to blue copper protein [Ricinus communis]	binding
	5	95	117	FF535511	similar to bromodomain-containing protein [Ricinus communis, EEF37258.1]	catalytic activity
**EACMV-UG-mir-1-18***	3	219	238	DB939560	similar to 40S ribosomal protein S26 [Ricinus communis, EEF41563.1]	structural molecule activity
	3.5	320	339	CK644406	similar to blue copper protein precursor [Ricinus communis]	binding
	4	364	384	DB922409	similar to superoxide dismutase [fe] [Ricinus communis, EEF41460.1]	binding
	4	132	151	CK645440	similar to chromo domain protein [Ricinus communis]	binding
	4.5	95	116	FF535511	similar to bromodomain-containing protein [Ricinus communis, EEF37258.1]	catalytic activity
	4.5	276	297	DB952327	similar to ankyrin repeat domain protein [Ricinus communis, EEF47974.1]	binding
	4.5	331	352	FF381021	similar to ATP-dependent Clp protease proteolytic subunit [Ricinus communis, EEF34901.1]	binding
	4.5	163	182	DV448840	similar to mitogen-activated protein kinase kinase [Ricinus communis, EEF39361.1]	binding
	5	213	233	FF380533	similar to hydrolase, hydrolyzing O-glycosyl compounds [Ricinus communis, EEF30440.1]	binding
	5	126	147	CK652150	similar to protein phosphatase [Ricinus communis, EEF49929.1]	catalytic activity
**EACMV-UG-mir-1-19***	3.5	206	227	FF380533	similar to hydrolyzing O-glycosyl compounds [Ricinus communis, EEF30440.1]	binding
	4.5	6	28	DV450868	similar to 40S ribosomal protein S24B [Hevea brasiliensis, ADR71268.1]	structural molecule activity
	5	223	245	DV453504	similar to pumilio [Ricinus communis, EEF38125.]	binding
	5	442	465	FF380021	similar to ubiquinone biosynthesis protein COQ9, mitochondrial precursor, [Ricinus communis, EEF46249.1]	binding
	5	210	231	DR085969	similar to optic atrophy 3 protein [Ricinus communis, EEF39804.1]	
**EACMV-UG-mir-2-1**	3.5	223	242	DB947470	similar to ATP synthase delta chain, chloroplastic [Vitis vinifera, XP_002274963.1]	binding
	3.5	219	239	DV452317	similar to 60S ribosomal protein L6 [Ricinus communis, EEF37645.1]	structural molecule activity
	4	145	165	DB940253	similar to ammonium transporter [Ricinus communis, EEF31872.1]	transporter activity
	4	244	264	CK652637	similar to RNase l inhibitor [Ricinus communis, EEF34119.1]	binding
	4	181	202	DV446663	similar to ubiquitin-conjugating enzyme E2 [Ricinus communis, EEF51304.1]	binding
	4.5	500	520	DB934312	similar to sucrose synthase [Ricinus communis, EEF39300.1]	catalytic activity

	5	106	126	DB948021	similar to ubiquitin-protein ligase [Ricinus communis, EEF40124.1]	catalytic activity
**EACMV-UG-mir-2-2**	3.5	455	476	FF379719	similar to protein with unknown function [Ricinus communis, EEF41267.1]	
	4	2	23	DV441023	similar to endosomal P24A protein precursor [Ricinus communis, EEF34973.1]	
	4	472	491	DB923111	similar to ent-kaurene synthase B, chloroplast precursor [Ricinus communis, EEF28689.1]	catalytic activity
	4.5	471	491	DB950113	similar to lupus la ribonucleoprotein [Ricinus communis, EEF40478.1]	binding
	4.5	187	208	DV444614	similar to methyltransferase [Ricinus communis, EEF33544.1]	catalytic activity
	4.5	232	253	DB941252	similar to ran GTPase binding protein [Ricinus communis, EEF48024.1]	binding
**EACMV-UG-mir-2-3***	3	11	30	CK646416	similar to phospholipid-sterol O-acyltransferase-like [Glycine max, XP_003553502]	catalytic activity
	3	289	310	DV457430	similar to endonuclease [Ricinus communis, EEF36937.1]	binding
	3.5	293	313	DR088252	similar to glucose-1-phosphate adenylyltransferase [Ricinus communis, EEF49428.1]	catalytic activity
	4	372	393	FF380284	similar to ubiquitin fusion degradaton protein [Ricinus communis, EEF49506.1]	binding
	5	375	396	DV443492	similar to cullin [Medicago truncatula, AES64163.1]	
	5	251	272	DB922691	similar to pentatricopeptide repeat-containing protein [Ricinus communis, EEF45402.1]	binding
**EACMV-UG-mir-2-4***	3.5	686	705	FF381511	similar to conserved hypothetical proteins [Ricinus communis, EEF47036.1, EEF28577.1]	
	4	155	174	DV441296	similar to dual specificity phosphatase Cdc25 [Ricinus communis, EEF45101.1]	catalytic activity
	4.5	125	145	DV452169	similar to protein phosphatase 2c [Ricinus communis, EEF52889.1]	binding
**EACMV-UG-mir-2-5***	4	116	136	DB922626	similar to predicted protein [Populus trichocarpa, ABK95421.1]	
	4.5	375	395	FF535553	similar to nucleic acid binding protein [Ricinus communis, EEF42371.1]	binding
	5	519	538	DB921122	similar to pentatricopeptide repeat-containing protein [Ricinus communis, EEF48438.1]	binding
**EACMV-UG-mir-2-6***	3.5	669	688	DV447588	similar to conserved hypothetical protein [Ricinus communis, EEF42715.1]	
	4.5	382	403	DV443635	similar to proteinase inhibitor [Ricinus communis, EEF41423.1]	enzyme regulator activity
	4.5	293	313	DV456795	similar to GTP-binding protein sar1[Ricinus communis, EEF47281.1]	binding
	4.5	143	164	FG805453	similar to cold-inducible RNA-binding protein [Ricinus communis, EEF38570.1]	binding
**EACMV-UG-mir-2-7***	3.5	12	32	CK646416	similar to phospholipid-sterol O-acyltransferase-like [Glycine max, XP_003553502]]	catalytic activity
	3.5	405	425	CK641772	similar to grave disease carrier protein [Ricinus communis, EEF46089.1]	
	4	481	501	DB933402	similar to DNA binding protein [Ricinus communis, EEF41947.1]	binding
	4	48	68	DB936739	similar to uncharacterized protein LOC100254082 [Vitis vinifera, CAN73522.1]	
	4.5	330	350	DV453019	similar to nucleolar phosphoprotein [Ricinus communis, EEF32038.1]	binding

4.5	408	428	CK643376	similar to 4-hydroxy-3-methylbut-2-en-1-yl diphosphate synthase [Ricinus communis]	catalytic activity
4.5	667	687	FF380075	similar to FK506 binding protein [Ricinus communis, EEF29893.1]	catalytic activity
5	117	137	DV447561	similar to GATA transcription factor [Ricinus communis, EEF39774.1]	nucleic acid binding<br>transcription factor activity
5	17	37	DV441108	similar to ethylene-responsive transcription factor [Ricinus communis, EEF31169.1]	nucleic acid binding<br>transcription factor activity
5	292	312	DV457430	similar to endonuclease [Ricinus communis, EEF36937.1]	binding
5	326	346	DV453960	similar to heat shock factor protein [Ricinus communis, EEF32881.1]	nucleic acid binding<br>transcription factor activity

Table S8 Plant miRs/miRs* from the miRBase sequence DataBase, release 18, , with putative targets in DNA-A of ACMV [GenBank: JN053423, JN053421] and EACMV-UG [GenBank: JN053454, JN053447] using RNAhybrid

Virus	miRNA	ΔG (kcal/mol)	Genomic position	Target-miR hybrid	Assigned ORF	Conservation
**ACMV**	miR156e*	-25.9	1696	target 5' G U A A A 3'<br>GAU GCAGAGG AG UAGUGGG<br>CUA UGUCUCU UC GUCACUC<br>miRNA 3' C C G 5'	AC1	conserved
	miR159c	-26.1	2590	target 5' G A U A U 3'<br>AGGAGUUCUC UUU G CCAAG<br>UCCUCGAGGG AAG U GGUUC<br>miRNA 3' U A 5'	AC1	conserved
	miR160a	-30.2	871	target 5' A A U 3'<br>UGGCAUG A GGAGC AGGCG<br>ACCGUAU U CCUCG UCCGU<br>miRNA 3' G C G 5'	AV1	conserved
	miR164a	-26.5	1642	target 5' A U 3'<br>GCGC U UCUUGCUUUUCC<br>CGUG A GGGACGAAGAGG<br>miRNA 3' A C C U 5'	AC1	conserved
	miR164c	-26.5	1642	target 5' A U 3'<br>GCGC U UCUUGCUUUUCC<br>CGUG A GGGACGAAGAGG<br>miRNA 3' G C C U 5'	AC1	conserved
	miR164d	-26.9	1641	target 5' U U 3'<br>AGCGC U UCUUGCUUUUCC<br>UCGUG A GGGACGAAGAGG<br>miRNA 3' C C U 5'	AC1	conserved
	miR166k*	-26.5	1834	target 5' C U A 3'<br>CCUUG A CCAGGCAGCAAU<br>GGGGC U GGUCUGUUGUUA<br>miRNA 3' C GG 5'	AC1	conserved

miR169aa	-26.4	2556	target 5' G G A A G 3'<br>UGA A AG CAUUCUUGGCUU<br>GCU U UC GUAAGAACCGAG<br>miRNA 3' G G A 5'	AC1 and AC4	conserved
miR169*	-27.9	2557	target 5' U A A U 3'<br>GAG AAG CAUUCUUGGCU<br>CUC UUC GUAGGAACCGA<br>miRNA 3' G G A U 5'	AC1 and AC4	conserved
miR319c	-26.7	2589	target 5' C A U A G 3'<br>GAGGAGUUCUC UUU G CCAA<br>UUCCUCGAGGG AAG C GGUU<br>miRNA 3' U A 5'	AC1	conserved
miR397a	-28.3	1094	target 5' A A 3'<br>GUCAAC UUGCACUCAAU<br>UAGUUG GACGUGAGUUA<br>miRNA 3' G C CU 5'	AC3	conserved
miR397b	-28.3	1094	target 5' A A 3'<br>GUCAAC UUGCACUCAAU<br>UAGUUG GACGUGAGUUA<br>miRNA 3' G C CC 5'	AC3	conserved
miR446	-28.8	1741	target 5' G U A 3'<br>GUUUCCCGUA UUCGUGUUGG<br>UAAAGGGUAU AAGUAUAACU<br>miRNA 3' GG AC 5'	AC1	nonconserved
miR841c	-27.2	1272	target 5' C A A 3'<br>UCGGUU UC AG GGCUCGUA<br>AGUCAA AG UC CCGAGCAU<br>miRNA 3' A U A 5'	AC3	nonconserved
miR854a	-27.6	1645	target 5' G G U U 3'<br>CUUCUU CUU UCCUCGUC<br>GGAGGA GGA AGGAGUAG<br>miRNA 3' GA G U 5'	AC1	nonconserved
miR948	-25.3	1645	target 5' G A U 3'	AC1	nonconserved

			UG GGUUCC C CAGUCUGA<br>GC CUAGGG G GUCGGACU<br>miRNA 3' G U U 5'		
miR1107	-26.3	2337	target 5' G G A 3'<br>UUGGG UGGAACUGGUGCU<br>GACUU ACCUUGGUCGUGG<br>miRNA 3' UG A 5'	AC1 and AC4	nonconserved
miR1117	-25.3	2336	target 5' U G G C 3'<br>GUU G G UG GAACUGGUGCUA<br>CAA C C GU CUUGGCCAUGAU<br>miRNA 3' C G A G G 5'	AC1 and AC4	nonconserved
miR1864	-25.5	910	target 5' G A G U U 3'<br>GUUG AUCAUCACGU AC UAUAA<br>UAAC UGGUAGUGCA UG AUGUU<br>miRNA 3' UG A 5'	AV1	nonconserved
miR2094-3p	-27.5	2347	target 5' A G 3'<br>AC UGG UGCUACAGCUU<br>UG ACC ACGGUGUCGAG<br>miRNA 3' GC C U AC 5'	AC1 and AC4	nonconserved
miR2640a	-27.5	242	target 5' U G C A U 3'<br>UGG CCA G UCUGGUGAGGGA<br>AUC GGU C AGGCCGUUCCUU<br>miRNA 3' C A G 5'	AV2	nonconserved
miR2668	-27	2324	target 5' U A A U A 3'<br>GGC CCUUGA UGU GGGGUGGA<br>CUG GGGAUU ACG UCCUACUU<br>miRNA 3' A U 5'	AC1 and AC4	nonconserved
miR2911	-28.3	1644	target 5' C U A 3'<br>UUCU GCUU UUCCUCGGCU<br>AGGG CGGG AGGGGGCCGG<br>miRNA 3' U C 5'	AC1	nonconserved
miR3632*	-27.3	1751	target 5' A G A G C 3'	AC1	nonconserved

				UUUC UGUUGG CU CCAGUCC<br>AAAG GUAGCC GG GGUUAGG<br>miRNA 3' GG G 5'		
**EACMV-UG**	miR156e*	-25.9	1729	target 5' G U A A A 3'<br>GAU GCAGAGG AG UAGUGGG<br>CUA UGUCUCU UC GUCACUC<br>miRNA 3' C C G 5'	AC1	conserved
	miR156g	-25.8	2558	target 5' G G U 3'<br>G CCUCU UCUUUUGUUAA<br>C GGAGA AGAAGACAGUU<br>miRNA 3' CA G U 5'	AC1	conserved
	miR160f*	-26	1036	target 5' A C A U G U 3'<br>CAUGCCU G A CCU UGUAUGC<br>GUACGGA C U GGA GCGUGCG<br>miRNA 3' C G 5'	AV1	conserved
	miR166h*	-25.6	1869	target 5' G U A G 3'<br>UUG ACC GGCGUCGUUU<br>AGC UGG CUGCAGUAAG<br>miRNA 3' CA C C G 5'	AC1	conserved
	miR171a	-26.7	1366	target 5' U A U U 3'<br>GUGAUG UG CGUGGUUCA<br>CACUAU AC GCGCCGAGU<br>miRNA 3' A C U 5'	AC2 and AC3	conserved
	miR171b	-25.5	1364	target 5' A A U U 3'<br>GUGUGAUG UG CGUGGUUCA<br>UACACUAU AC GUGCCGAGU<br>miRNA 3' A C 5'	AC2 and AC3	conserved
	miR171d	-30.3	1364	target 5' A A U U 3'<br>GUGUGAUG UG CGUGGUUCA<br>UACACUAU AC GCGCCGAGU<br>miRNA 3' A C 5'	AC2 and AC3	conserved
	miR171f	-27.9	1366	target 5' U A U U 3'<br>GUGAUG UG CGUGGUUCA	AC2 and AC3	conserved

			`           CACUAU AC GCGCCGAGU`<br>`miRNA  3' U      A  C         U 5'`		
miR399c*	-25.1	379	`target 5' G              U A    C 3'`<br>`           GUG CGGAGGAGGC G ACUU`<br>`           CAC GUUUCCUCUG C UGGG`<br>`miRNA  3' A   G            A      5'`	AV2	conserved
miR472*	-25.2	1957	`target 5' U          G         C 3'`<br>`           GUUUUGCC G UUCGGCUAU`<br>`           UAAAACGG U AAGCUGGUA`<br>`miRNA  3' C        A G           5'`	AC1	nonconserved
miR478a	-25.2	143	`target 5' U  G   C       U   G       A 3'`<br>`           UC UUG GAAGUAG AGA CGCGUCA`<br>`           AG GAU UUUUAUC UCU GUGCAGU`<br>`miRNA  3'    G           U             5'`	IR	nonconserved
miR482	-25.7	2684	`target 5'  C   C               C 3'`<br>`            AAU GGUGGAA UGGGGGG`<br>`            UUA CCGCCUU ACCCUUC`<br>`miRNA  3' CC   C       A       U 5'`	IR	conserved
miR859	-26.3	1412	`target 5'  G        A          A 3'`<br>`            UGAUUUCG AAUAGAGGGG`<br>`            ACUGAAGU UUGUCUCUCU`<br>`miRNA  3' AA        G            5'`	AC2 and AC3	nonconserved
miR868*	-26.8	1723	`target 5'  G     G  U   A       A 3'`<br>`            GUCCU GA UGC GAGGAAG`<br>`            UAGGA UU ACG CUCUUUC`<br>`miRNA  3' UG     G  C   G         5'`	AC1	nonconserved
miR1082b	-32.5	619	`target 5' G   G                 A 3'`<br>`           CGU GGCC G GGGCUGACAC`<br>`           GCG CCGG C UCCGGUUGUG`<br>`miRNA  3'     G    U G            5'`	AV1	nonconserved
miR1111	-28.1	1391	`target 5' C                     C 3'`<br>`           CUGG CCU CUU GUCGUGGU`<br>`           GACC GGA GAA CAGUACCG`	AC2 and AC3	nonconserved

			miRNA 3' A C U 5'		
miR1118	-25	2477	target 5' A U 3'<br>CC CCA UCC UGAUGUAGU<br>GG GGU AGG AUUACAUCA<br>miRNA 3' AG A A U C 5'	AC1 and AC4	nonconserved
miR1311	-30.5	2110	target 5' U C A 3'<br>GGCGGAGC UGG AAGAUUCU<br>CCGCCUUG ACC UUUUGAGA<br>miRNA 3' G CU 5'	AC1	nonconserved
miR1446a	-26.7	847	target 5' U U U U 3'<br>UUGAGGGA AGG UUCAGG<br>AACUCCCU UCU AAGUCU<br>miRNA 3' C C U 5'	AV1	nonconserved
miR1510b	-25.4	2628	target 5' C U 3'<br>GGUGGAAUGGG G GGCAAUA<br>CCACCUUAUCC U UUGUUGU<br>miRNA 3' A U 5'	AC1	nonconserved
miR1520j	-26.2	1370	target 5' A U C 3'<br>UGA UGUCGUG G UCAUGUUC<br>ACU ACAGUAC C AGUGCAAG<br>miRNA 3' A A A AA 5'	AC2 and AC3	nonconserved
miR2104	-27.9	1383	target 5' U C U G G 3'<br>CAUGUUC C G CCUCUUGUCGU<br>GUGCGAG G U GGGGAGCGGCG<br>miRNA 3' C A 5'	AC2 and AC3	nonconserved
miR2119	-25.5	1791	target 5' A G C G G 3'<br>UU CUUUGC A UCCCUUUGG<br>AA GGGAUG U AGGGAAACU<br>miRNA 3' G U G 5'	AC1	nonconserved
miR2119	-26.2	65	target 5' G C G U 3'<br>UCCCUGCG ACUU UUUUGG<br>GGGGAUGU UGAG GAAACU<br>miRNA 3' AA G 5'	IR	nonconserved

miR2927	-30.4	173	target 5' U A A U 3'<br>GUGGG UCCAUUGGUGA CGAU<br>UACCC AGGUAGCUGCU GCUG<br>miRNA 3' G G U 5'	AV2	nonconserved
miR3633b	-27.7	63	target 5' U U G A 3'<br>GGUCCC GC CACUUGUUUU<br>CUAGGG CG GUGGGUAAGG<br>miRNA 3' AU U 5'	IR	nonconserved
miR4390	-29.4	2515	target 5' A A U A 3'<br>AUUUGAUA UCG CGGGUACGA<br>UGGGCUAU GGC GCUCAUGCU<br>miRNA 3' UA G U 5'	AC1	nonconserved
miR4409	-31.6	1940	target 5' C G A U 3'<br>CAGU AACGAGCCCAC UUGUU<br>GUCA UUGUUUGGGUG AACAA<br>miRNA 3' G U 5'	AC1	nonconserved

Table S9 Plant miRs/miRs* from the miRBase sequence DataBase, release 18, with putative targets in DNA-A of ACMV [GeneBank: JN053423, JN053421] and EACMV-UG [GeneBank: JN053454, JN053447] using psRNATarget

Virus	miRNA	Expectation Score	Target start	Target end	Assigned ORF	Conservation
**ACMV**	miR159a	5	2242	2261	AC1 and AC4	conserved
	miR159b	5	2242	2261	AC1 and AC4	conserved
	miR170	4.5	425	443	AV1	conserved
	miR395b	5	1786	1806	AC1	conserved
	miR397b	4.5	1095	1113	AC2 and AC3	conserved
	miR841	4.5	272	290	AV2	nonconserved
	miR868	4.5	1687	1708	AC1	nonconserved
	miR1446a	4	813	832	AV1	nonconserved
	miR1887	4.5	800	819	AV1	nonconserved
	miR2630a	4.5	668	688	AV1	nonconserved
	miR2669	5	341	362	AV2	nonconserved
	miR2905	5	979	999	AV1	nonconserved
	miR3513-5p	5	1051	1070	AV1	nonconserved
	miR4243	5	2008	2027	AC1	nonconserved
	miR4246	5	1065	1084	AC3	nonconserved
**EACMV-UG**	miR156c	5	2558	2576	AC1	conserved
	miR171a	5	1366	1386	AC2 and AC3	conserved
	miR171b	5	1367	1386	AC2 and AC3	conserved
	miR171d	5	1367	1386	AC2 and AC3	conserved
	miR171f	4.5	1367	1386	AC2 and AC3	conserved
	miR397a	5	2242	2262	AC1	conserved
	miR472	5	1960	1978	AC1	nonconserved
	miR477g	4.5	839	858	AV1	conserved
	miR771	4.5	628	647	AV1	nonconserved
	miR857	4.5	2744	2768	IR	nonconserved
	miR859	4.5	1412	1431	AC2 and AC3	nonconserved
	miR1084	4.5	2123	2142	AC1	nonconserved
	miR1446a	4	848	867	AV1	nonconserved
	miR1887	4.5	835	854	AV1	nonconserved
	miR2588a	5	1580	1600	AC1 and AC2	nonconserved
	miR2588b	5	1580	1600	AC1 and AC2	nonconserved
	miR2668	5	2362	2383	AC4	nonconserved
	miR2669	5	379	400	AV1	nonconserved
	miR3513-5p	5	1086	1105	AV1	nonconserved
	miR4232	4.5	2501	2521	IR	nonconserved
	miR4238	5	308	329	AV2	nonconserved

miR4379	5	206	226	AV2	nonconserved
miR4390	4	2516	2537	AC1	nonconserved
miR4399	4.5	2173	2194	AC1	nonconserved
miR4412	4.5	1843	1862	AC1	nonconserved

Table S10 Predicted putative target location of plant miRs/miRs* in *Jatropha* ESTs using psRNATarget

miRNA	Expectation Score	Target start	Target end	Genbank accession	Target desccription	Target function
miR156c	3	384	403	GT972922	similar to conserved hypothetical protein [Ricinus communis, EEF46527.1]	
miR156c	3.5	282	301	GW616458	similar to E3 ubiquitin-protein ligase RHF1A [Vitis vinifera, CBI26202.3]	catalytic activity
miR156e*	3.5	372	393	FM891233	similar to chloroplast acyl-ACP thioesterase [Jatropha curcas, ACT09366]	catalytic activity
miR159a	2.5	452	471	GT975153	similar to copper-zinc superoxide dismutase [Ricinus communis, EEF38668.1]	binding
miR159b	2.5	318	338	GT978051	similar to copper-zinc superoxide dismutase [Ricinus communis, EEF38668.1]	binding
miR159c	2	318	338	GT978051	similar to copper-zinc superoxide dismutase [Ricinus communis, EEF38668.1]	binding
miR160a	3.5	279	298	FM889693	similar to gag-pol polyprotein [Medicago truncatula, ACL97387.1]	binding
miR160a	4	148	167	GR209297	similar to phosphatidylinositol-glycan biosynthesis protein [Medicago truncatula, AET03225.1]	catalytic activity
miR160f*	4	496	516	GW611252	similar to cyclic nucleotide-gated ion channel [Ricinus communis, EEF36596.1]	ion channel activity
miR164a	1.5	427	446	GT978826	similar to NAC domain containing protein [Ricinus communis, EEF47842.1]	binding
miR164c	1.5	426	446	GT978826	similar to NAC domain containing protein [Ricinus communis, EEF47842.1]	binding
miR164d	1.5	427	446	GT978826	similar to NAC domain containing protein [Ricinus communis, EEF47842.1]	binding
miR166h*	4	343	363	GW876848	similar to aldose 1-epimerase [Ricinus communis, EEF40837.1]	binding
miR166h*	4	362	382	GT971031	similar to AGT3 (alanine-glyoxylate aminotransferase) [Ricinus communis, EEF31063.1]	catalytic activity
miR166k*	4	13	34	GT975642	similar to conserved hypothetical protein [Ricinus communis, EEF34426.1]	
miR169aa	2.5	407	427	GT972308	similar to conserved hypothetical protein [Ricinus communis, EEF46406.1]	
miR169aa	3.5	46	66	GT970459	similar to predicted protein [Populus trichocarpa, EEF10343.1]	
miR169*	2.5	407	427	GT976844	similar to conserved hypothetical protein [Ricinus communis, EEF36406.1]	
miR170	4	68	87	GT981637	similar to ASP1 (aspartate aminotransferase)[Ricinus communis, EEF32176.1]	catalytic activity
miR170	4	220	239	GT973501	similar to ribulose-5-phosphate-3-epimerase [Ricinus communis,EEF47836.1]	catalytic activity
miR171a	4	420	439	GW618999	similar to anther-specific protein LAT52 precursor [Ricinus communis, EEF38957.1]	
miR171b	4	17	36	GT970431	similar to hAT dimerisation domain [Oryza sativa , ABF97129.1]	binding
miR171b	4	399	418	GW875726	similar to shikimate dehydrogenase [Ricinus communis, EEF41656.1]	binding
miR171d	4	444	463	GW880593	similar to Beta tubulin [Ricinus communis, EEF39168.1]	structural molecule activity
miR171f	3.5	68	87	GT981637	similar to ASP (aspartate aminotransferase) [Ricinus communis, EEF32176.1]	catalytic activity
miR319c	3.5	318	337	GT978051	similar to copper-zinc superoxide dismutase [Ricinus communis, EEF38668.1]	binding
miR319c	3.5	368	388	GW875627	similar to cytochrome P450 Ricinus communis, EEF52507.1]	electron carrier activity
miR395b	3	506	526	GW879884	similar to sulfate adenylyltransferase [Ricinus communis, EEF45221.1]	catalytic activity
miR395b	3.5	245	264	GW875884	similar to zinc finger protein [Ricinus communis, EEF52740.1]	binding
miR397a	3.5	420	440	FM892216	similar to breast carcinoma amplified sequence [Ricinus communis, EEF32832.1]	
miR397a	3.5	347	367	GT978274	similar to triacylglycerol lipase [Ricinus communis, AAY17358.1]	catalytic activity
miR397b	3.5	419	439	GT977255	similar to conserved hypothetical protein [Ricinus communis, EEF42790.1]	
miR399c*	3.5	12	31	GT972088	similar to cysteine synthase [Ricinus communis, EEF45296.1]	catalytic activity
miR446	3	493	515	GT981181	similar to iron-sulfur cluster scaffold protein [Hevea brasiliensis, ADM67612.1]	structural molecule activity
miR472	3.5	103	122	GT977925	similar to isocitrate lyase [Ricinus communis, EEF32648.1]	catalytic activity
miR472a	2.5	172	191	GT978276	similar to s-locus-specific glycoprotein S6 precursor [Ricinus communis, EEF37743.1]	binding
miR472a	3.5	29	49	GT982525	similar to isoamyl acetate-hydrolyzing esterase [Ricinus communis, EEF28734.1]	catalytic activity

miR476a	2.5	462	481	GW614542	similar to pantoate-beta-alanine ligase [Ricinus communis, EEF41264.1]	binding
miR476a	3	525	545	GT975028	similar to pyrophosphate-dependent 6-phosphofructose-1-kinase [Ricinus communis, EEF52925.1]	catalytic activity
miR477g	3	462	481	FM894847	similar to starch branching enzyme II [Ricinus communis, EEF52859.1]	catalytic activity
miR477g	3.5	234	253	GT978997	similar to paramyosin[Ricinus communis, EEF47616.1]	catalytic activity
miR478a	4	511	534	GT981288	similar to NADH-ubiquinone oxidoreductase 24 kDa subunit [Ricinus communis, EEF41394.1]	binding
miR482	3	120	139	GW879579	similar to two-component response regulator ARR9 [Ricinus communis, EEF48328.1]	molecular transducer activity
miR841	3.5	183	203	GW880076	similar to histone h2a [Ricinus communis, EEF49869.1]	
miR854a	2	29	48	GT980392	similar to CSY (citrate synthase) [Ricinus communis, EEF29688.1]	catalytic activity
miR854a	2.5	147	169	GT970035	similar to KRP4 (KIP-related protein 4) [Populus trichocarpa, EEE84983.1]	enzyme regulator activity
miR857	2.5	618	638	GW615139	similar to protein transporter [Ricinus communis, EEF42056.1]	
miR859	3	519	539	GW875646	similar to conserved hypothetical protein [Ricinus communis, EEF41557.1]	
miR859	4	515	535	GT981267	similar to glutathione S-transferase [Ricinus communis, EEF51173.1]	catalytic activity
miR868	2.5	402	422	GW876988	similar to RNA binding protein [Ricinus communis, EEF41662.1]	binding
miR868	3	118	137	GT972521	similar to casein kinase [Ricinus communis, EEF30734.1]	binding
miR948	4	170	189	GW618863	similar to peptide transporter [Ricinus communis, EEF28493.1]	transporter activity
miR948	4	94	113	FM896813	similar to 40S ribosomal protein S5A [Hevea brasiliensis, ADR71286.1]	structural molecule activity
miR1107	3	531	551	GT979546	similar to trihelix factor GT-2-like [Glycine max, XP_003548983.1]	binding
miR1107	3	236	255	GW876853	similar to ATP binding protein [Ricinus communis, EEF37432.1]	binding
miR1082b	3.5	608	627	GT982339	similar to myb family transcription factor [Glycine max, XP_003524515.1]	binding
miR1082b	4	572	592	GT982572	similar to protein-tyrosine phosphatase mitochondrial 1, mitochondrial precursor [Ricinus communis, EEF48147.1]	catalytic activity
miR1084	3.5	312	331	GT975969	similar to phosphoric diester hydrolase [Ricinus communis, EEF38025.1]	catalytic activity
miR1111	2.5	26	46	GT977080	similar to sugar transporter [Ricinus communis, EEF45377.1]	transporter activity
miR1111	3.5	123	143	GW875366	similar to nodulation receptor kinase precursor [Ricinus communis, EEF33010.1]	binding
miR1117	3.5	40	60	GT969582	similar to predicted protein [Populus trichocarpa, EEE83498.1]	
miR1118	4	220	242	GW874906	Jatropha curcas flower and seed Jatropha curcas	
miR1311	2.5	68	85	GW880684	similar to mta/sah nucleosidase [Ricinus communis, EEF42368.1]	catalytic activity
miR1311	3.5	456	475	GW875363	similar to dead box ATP-dependent RNA helicase	binding
miR1446a	2.5	366	385	GT978057	similar to DNA-directed RNA polymerase II [Ricinus communis, EEF41911.1]	binding
miR1446a	3.5	222	241	GT969555	similar to gag-Pol polyprotein [Vitis vinifera, AAO26684.1]	binding
miR1510b	3	235	254	GW877068	similar to elongation factor tu [Ricinus communis, EEF37652.1]	binding
miR1510b	3.5	146	165	GT979652	similar to ethylene-responsive transcription factor [Ricinus communis, EEF29138.1]	nucleic acid binding transcription factor activity
miR1864	3.5	183	204	GT973964	similar to hypothetical protein VITISV_001215 [Vitis vinifera, CAN60374.1]	
miR1887	2.5	545	565	GW614952	similar to beta-glucosidase [Ricinus communis, EEF29253.1]	binding
miR2094-3p	4	364	383	GT975154	similar to ATP synthase beta chain 2, mitochondrial [Ricinus communis, EEF30158.1]	binding
miR2104	3	100	121	GT974599	similar to catalytic [Ricinus communis, EEF47629.1]	catalytic activity
miR2119	3.5	517	536	GW878245	similar to homeobox protein [Ricinus communis, EEF42201.1]	nucleic acid binding transcription factor activity
miR2588a	4	214	233	GT980589	similar to dead box ATP-dependent RNA helicase [Ricinus communis, EEF26699.1]	binding
miR2588b	4	214	233	GT980589	similar to dead box ATP-dependent RNA helicase [Ricinus communis, EEF26699.1]	binding

miR2630a	2.5	97	116	GW879963	similar to WRKY transcription factor 39 [Ricinus communis, EEF41412.1]	binding
miR2630a	3	447	466	GT981566	similar to cysteine proteinase [Ricinus communis, EEF51879.1]	catalytic activity
miR2640a	3	170	189	GT980056	similar to conserved hypothetical protein [Ricinus communis, EEF49901.1]	
miR2640a	3.5	342	361	GT975118	similar to hypothetical protein VITISV_010143 [Vitis vinifera, CAN71595.1]	
miR2668	3.5	291	311	FM891085	similar to elongation factor 1-alpha [Ricinus communis, EEF34338.1]	binding
miR2669	3	519	538	GT973172	similar to 26S proteasome regulatory complex [Ricinus communis, EEF52627.1]	enzyme regulator activity
miR2669	3.5	517	537	GW878759	similar to ribosomal pseudouridine synthase [Ricinus communis, EEF51731.1]	binding
miR2905	3.5	511	530	GW617764	similar to microsomal signal peptidase 23 kD subunit [Ricinus communis, EEF49804.1]	catalytic activity
miR2905	4	676	697	GT977219	similar to tubulin alpha chain [Ricinus communis, EEF52197.]	binding
miR2911	3.5	207	226	GW877131	similar to 30S Ribosomal protein S20 [Ricinus communis, EEF30134.1]	structural molecule activity
miR2911	3	239	258	GT978779	similar to transferase, transferring glycosyl groups [Ricinus communis, EEF34254.1]	catalytic activity
miR2927	4	338	358	GT970187	similar to protein binding protein [Ricinus communis, EEF37746.1]	
miR3513-5p	3	363	382	GW878452	similar to purine transporter [Ricinus communis, EEF28388.1]	transporter activity
miR3632	3	118	137	GO247104	similar to ADP-ribosylation factor [Ricinus communis, EEF40773.1]	binding
miR3633b	3	520	539	GT972658	similar to LysM domain GPI-anchored protein 1 precursor, putative [Ricinus communis, EEF48379.1]	binding
miR4232	3.5	203	225	GT228737	similar to conserved hypothetical protein [Ricinus communis, EEF44307.1]	
miR4238	2	464	483	GT978314	similar to phosphoethanolamine/phosphocholine phosphatase [Ricinus communis, EEF34991.1]	catalytic activity
miR4238	3	46	65	FM896524	similar to 40S ribosomal protein S7 [Ricinus communis, EEF30900.1]	structural molecule activity
miR4239	3	300	320	GW612689	similar to acetyl co-enzyme A carboxylase biotin carboxylase subunit [Ricinus communis, EEF42607.1]	binding
miR4246	3	137	157	GT977929	similar to glycosyl transferase [Ricinus communis, EEF34070.1]	catalytic activity
miR4379	2	256	279	FM888970	similar to predicted protein [Populus trichocarpa, EEE72171.1]	
miR4379	3	72	92	GW879132	similar to peptidyl-tRNA hydrolase [Ricinus communis, EEF45636.1] |	catalytic activity
miR4390	4	530	551	FM894923	similar to photosystem I P700 chlorophyll a apoprotein A2 [Oryza sativa Japonica Group, BAG87218.1]	binding
miR4399	3.5	565	586	GW618386	similar to 40S ribosomal protein S13 [Ricinus communis, EEF50364.1]	structural molecule activity
miR4409	3.5	336	357	GT982448	similar to cak1 [Ricinus communis, EEF48229.1]	binding
miR4412	4	36	55	FM894220	similar to hydrolase, hydrolyzing O-glycosyl compounds [Ricinus communis, EEF47769.1]	binding

Table S11 Predicted putative target location of plant miRs/miRs* in cassava ESTs using psRNATarget

miRNA	Expectation Score	Target start	Target end	Genbank accession	Target desccription	Molecular function
miR156c	2.5	142	161	DV456109	similar to conserved hypothetical protein [Ricinus communis, EEF47445.1]	
miR156e*	3.5	256	275	DB945167	similar to uv excision repair protein rad23 [Ricinus communis, EEF33228.1]	binding
miR156g	0.5	448	467	DV455740	similar to squamosa promoter-binding protein [Ricinus communis, EEF50837.1]	binding
miR159a	3	242	261	DV445931	similar to calcineurin B subunit [Ricinus communis, EEF44849.1]	binding
miR159a	3.5	104	124	DV453037	similar to cysteine protease inhibitor [Manihot esculenta, AAF72202.1]	catalytic activity
miR159b	2	137	157	DB942210	similar to flavonol synthase/flavanone 3-hydroxylase [Ricinus communis, EEF28715.1]	catalytic activity
miR159b	2	32	52	DB948907	similar to oxidoreductase [Ricinus communis, EEF31612.1]	catalytic activity
miR160f	4	335	355	DB943991	similar to dimethylaniline monooxygenase [Ricinus communis, EEF35124.1]	binding
miR164a	3.5	605	624	FF536019	similar to glutathione s-transferase [Ricinus communis, EEF29553.1]	catalytic activity
miR164c	2.5	460	480	FF536822	similar to NAC domain-containing protein 21/22 [Ricinus communis, EEF47842.1]	binding
miR164d	3.5	368	388	DR085222	similar to WRKY transcription factor [Ricinus communis, EEF43114.1]	nucleic acid binding transcription factor activity
miR166h*	4	250	270	DV457472	similar to GATA-4/5/6 transcription factor, partial [Populus balsamifera, ACZ67163.1]	nucleic acid binding transcription factor activity
miR166k*	3	588	607	BM259772	similar to huntingtin interacting protein [Ricinus communis, EEF46647.1]	catalytic activity
miR166k*	4	389	408	DV458662	similar to ATP binding protein [Ricinus communis, EEF39485.1]	binding
miR169aa	2	318	337	DV443290	similar to nuclear transcription factor Y subunit A-1 [Ricinus communis, EEF48902.1]	binding
miR169*	2.5	534	554	DV445967	similar to nuclear transcription factor Y subunit A-1 [Ricinus communis, EEF48902.1]	binding
miR169*	3	219	239	DV454576	similar to transcription factor [Ricinus communis, EEF38283.1]	binding
miR170	4	171	190	DB944656	similar to plastid 1-acylglycerol-phosphate acyltransferase [Jatropha curcas, AEZ56251.1]	catalytic activity
miR171a	3.5	442	461	DV442482	similar to conserved hypothetical protein [Ricinus communis, EEF40419.1]	
miR171b	3.5	8	28	DV442624	similar to Beta-galactosidase [Medicago truncatula, AES67469.1]	binding
miR171b	3.5	474	493	DB942692	similar to 50S ribosomal protein L21 [Ricinus communis, EEF35552.1]	structural molecule activity
miR171d	3.5	588	607	DV442297	similar to cysteine protease [Ricinus communis,EEF46275.1]	catalytic activity
miR171f	3.5	322	342	FG804848	similar to chloroplast inner envelope protein [Ricinus communis, EEF41373.1]	catalytic activity
miR171f	4.5	173	194	DV457745	similar to vacuolar ATP synthase proteolipid subunit 1, 2, 3 [Ricinus communis, XP_002531515.1]	transporter activity
miR319c	2.5	352	370	DV442935	similar to conserved hypothetical protein [Ricinus communis, EEF51355.1]	
miR395b	3.5	77	97	DV456132	similar to conserved hypothetical protein [Ricinus communis, EEF30907.1]	
miR397a	1.5	352	371	DR087678	similar to laccase [Ricinus communis, EEF41505.1]	binding

miR397a	3.5	347	367	DV445382	similar to triose phosphate/phosphate translocator, chloroplast precursor [Ricinus communis, EEF50815.1]	transporter activity
miR397b	3	212	232	DB941259	similar to protein binding protein [Ricinus communis, EEF34752.1]	
miR397b	3	352	371	DR087678	similar to laccase [Ricinus communis, EEF41505.1]	binding
miR399c	2.5	69	89	DB924911	similar to thioredoxin m(mitochondrial)-type [Ricinus communis, EEF42031.1]	catalytic activity
miR446	2	645	666	DV449294	similar to glycerol kinase [Ricinus communis, EEF44845.1]	catalytic activity
miR472	2	271	290	DB947587	similar to alcohol dehydrogenase [Ricinus communis, EEF43600.1]	binding
miR472	3.5	126	146	DV442917	similar to conserved hypothetical protein [Ricinus communis, EEF31937.1]	
miR472a	3	355	375	DV452251	similar to glycosyltransferase QUASIMODO1 [Ricinus communis, EEF29635.1]	catalytic activity
miR472a	3.5	89	108	DR086205	similar to histone-lysine n-methyltransferase, suvh [Ricinus communis, EEF36840.1]	binding
miR477g	3	62	81	DV451042	similar to ubiquitin-conjugating enzyme [Ricinus communis, EEF36411.1]	binding
miR477g	3	311	330	DB940824	similar to lrr receptor-linked protein kinase [Ricinus communis, EEF44487.1]	binding
miR478a	2.5	30	53	FG806373	similar to cullin [Ricinus communis, EEF39908.1]	
miR478a	3.5	221	244	DV441681	similar to ubiquitin carboxyl-terminal hydrolase [Ricinus communis, EEF36623.1]	binding
miR482	3	168	187	DV442993	similar to ATP binding protein [Ricinus communis, EEF36976.1]	binding
miR482	3	264	283	DB933070	similar to arsenical pump-driving atpase [Ricinus communis, EEF31448.1]	binding
miR841	3.5	556	575	DV446422	similar to proteasome maturation protein [Ricinus communis, EEF36388.1]	
miR854a	2	160	180	DB936379	similar to conserved hypothetical protein [Ricinus communis, EEF29835.1]	
miR857	2.5	111	131	DB946073	similar to s-adenosylmethionine-dependent methyltransferase [Ricinus communis, EEF48491.1]	catalytic activity
miR859	3	276	296	DR086701	similar to conserved hypothetical protein [Ricinus communis, EEF36909.1]	
miR859	4	206	226	DV444254	similar to two-component system sensor histidine kinase/response regulator [Ricinus communis, EEF29785.1]	binding
miR868	3	191	212	CK640893	similar to oligopeptidase A [Ricinus communis, EEF35149.1]	binding
miR868	3	519	538	DR086088	similar to pentatricopeptide repeat-containing protein [Ricinus communis, EEF36748.1]	binding
miR948	3.5	516	535	DB945134	similar to DNA binding protein [Ricinus communis, EEF50512.1]	binding
miR1082b	3.5	49	69	FG805052	similar to 40S ribosomal protein S25-1 [Ricinus communis, EEF49429.1]	structural molecule activity
miR1082b	4	519	538	DV456554	similar to quinone oxidoreductase [Ricinus communis, EEF27237]	binding
miR1107	2.5	481	500	DB922869	similar to conserved hypothetical protein [Ricinus communis, EEF41125.1]	
miR1111	3.5	464	483	DB939768	similar to calcium binding protein/cast [Ricinus communis, EEF42084.1]	binding
miR1117	4.5	531	554	DB949524	similar to predicted protein [Ricinus communis, EEF45451.1]	
miR1118	1.5	147	167	DB937387	similar to eukaryotic translation initiation factor 2c [Ricinus communis]	binding
miR1311	2.5	1	20	CK647703	similar to chaperone protein dnaJ [Ricinus communis, EEF33453.1]	binding
miR1311	3	591	610	DB934800	similar to insulin-degrading enzyme [Ricinus communis, EEF30306.1]	binding
miR1446a	3.5	332	351	CK641626	similar to ubiquitin-like protein [Medicago truncatula, AES75920.1]	
miR1446a	3.5	113	132	CK641790	similar to polyubiquitin [Arabidopsis thaliana, AAC39466.1]	

miR1510b	3	125	144	BM260297	similar to ATP synthase D chain, mitochondrial [Ricinus communis, EEF32668.1]	catalytic activity
miR1520j	3	110	129	FG804802	similar to sodium-bile acid cotransporter[Ricinus communis, EEF51029.1]	transporter activity
miR1864	3.5	173	193	DV441336	similar to phosphoglycerate kinase [Ricinus communis, EEF48756.1]	catalytic activity
miR1887	2.5	319	341	DV441606	similar to pyruvate kinase [Ricinus communis, EEF36945.1]	binding
miR1887	3	366	386	DV458163	similar to sterol carrier [Ricinus communis, EEF52404.1]	binding
miR2094-3p	3.5	466	485	DB952064	similar to ubiquitin-conjugating enzyme E2 G [Ricinus communis, EEF33916.1]	binding
miR2104	2.5	428	447	CK643733	similar to double-stranded RNA binding protein [Ricinus communis, EEF32455.1]	binding
miR2119	2.5	454	474	DB946234	similar to protein kinase APK1A, chloroplast precursor [Ricinus communis, EEF42172.1]	binding
miR2588a	3.5	609	629	DV458160	similar to conserved hypothetical protein [Ricinus communis, EEF32195.1]	
miR2630a	3	42	62	DB950634	similar to alpha-1,4-glucan-protein synthase [UDP-forming] [Ricinus communis, EEF50063.1]	catalytic activity
miR2640a	3.5	397	416	DV443241	similar to aquaporin PIP1.3 [Ricinus communis, EEF51202.1]	transporter activity
miR2668	3	571	591	DV449893	similar to conserved hypothetical protein [Ricinus communis, EEF39535.1]	
miR2669	3.5	269	289	DB946376	similar to zinc ion binding protein [Ricinus communis, EEF52519.1]	binding
miR2905	3	274	293	FG806910	similar to GTP binding protein [Arabidopsis thaliana, AAM60858.1]	binding
miR2911	3	278	297	BI325193	similar to 40S ribosomal protein S27 [Ricinus communis, XP_002519401.1]	structural molecule activity
miR2927	3	411	431	DV446904	similar to tetracycline transporter [Ricinus communis, EEF41099.1]	
miR2927	4.5	569	590	DV451567	similar to poly(p)/ATP NAD kinase [Ricinus communis, EEF42098.1]	binding
miR3513-5p	3	655	675	DV445298	similar to cysteine synthase [Ricinus communis, EEF49709.1]	catalytic activity
miR3513-5p	3	25	44	DB943267	similar to ATP-dependent phosphoenolpyruvate carboxykinase [Medicago sativa, AF212109_1]	catalytic activity
miR3632*	3.5	408	429	DB946640	similar to late embryogenesis abundant protein Lea14-A [Ricinus communis, EEF29030.1]	
miR3632*	3.5	244	265	DB941218	similar to bHLH-like DNA binding protein [Vitis vinifera, ACC68685.1]	binding
miR3633b	2.5	203	222	DB938906	similar to metal ion binding protein [Ricinus communis, EEF37798.1]	catalytic activity
miR4232	3	18	38	DB951935	similar to protein phosphatase 2c [Ricinus communis, EEF46865.1]	binding
miR4238	1.5	115	136	DV457195	similar to stromal cell-derived factor 2 precursor [Ricinus communis, EEF46887.1]	catalytic activity
miR4239	4	113	133	DV449569	similar to DNA repair protein reca [Ricinus communis, EEF32430.1]	binding
miR4243	3	306	326	FF379689	similar to ribosomal protein S12 [Jatropha curcas, ACN72715.1]	structural molecule activity
miR4246	2.5	711	732	DV448805	similar to sedoheptulose-1,7-bisphosphatase, chloroplast [Ricinus communis, EEF31985.1]	catalytic activity
miR4379	2.5	212	235	DV454857	similar to predicted protein [Populus trichocarpa, EEE72171.1]	
miR4390	3.5	91	114	DV446169	similar to conserved hypothetical protein [Ricinus communis, EEF42790.1]	
miR4399	3	331	350	DV441093	similar to LOB domain-containing protein [Ricinus communis, EEF37083.1]	
miR4399	3	393	413	FF380985	similar to 50S ribosomal protein L32 [Hevea brasiliensis, ADR71288.1]	structural molecule activity

miR4409	2	384	404	DR084552	similar to conserved hypothetical protein [Ricinus communis, EEF47036.1]

Table S12 Detection of ACMV and EACMV-UG virus miRNAs from cassava and *Jatropha* samples. (+) amplied, (-) not amplified miRNAs

	Cassava				*Jatropha*	
Virus miRNA	S4C4	S2C6	S4C6	B2C15	K5J5	S4J12
ACMV-mir-1-1	-	+	+	-	-	-
ACMV-mir-1-3	-	-	-	-	-	-
ACMV-mir-1-4	-	+	+	-	-	-
ACMV-mir-1-5	-	+	+	-	-	-
ACMV-mir-1-6	-	-	+	-	-	-
ACMV-mir-1-8	-	-	-	-	-	-
ACMV-mir-2-1	-	-	-	-	-	-
ACMV-mir-2-3	-	-	-	-	-	-
ACMV-mir-2-4	-	-	-	-	-	-
ACMV-mir-2-7	-	-	-	-	-	-
ACMV-mir-3-1	-	-	-	-	-	-
ACMV-mir-3-2	-	+	+	-	-	-
ACMV-mir-3-3	-	-	-	-	-	-
ACMV-mir-4-1	-	+	+	-	-	-
ACMV-mir-4-2	-	+	+	-	-	-
ACMV-mir-4-3	-	-	-	-	-	-
ACMV-mir-4-4	-	+	+	-	-	-
ACMV-mir-4-5	-	-	-	-	-	-
ACMV-mir-5-1	-	+	+	-	-	-
ACMV-mir-5-3	-	+	+	-	-	-
ACMV-mir-5-4	-	+	-	-	-	-
ACMV-mir-5-5	-	+	-	-	-	-
ACMV-mir-6-1	-	-	-	-	-	-
ACMV-mir-6-3	-	-	-	-	-	-
ACMV-mir-6-4	-	-	-	-	-	-
ACMV-mir-6-6	-	-	-	-	-	-
ACMV-mir-7-1	-	-	-	-	-	-
EACMV-UG-mir-1-1	-	-	+	-	-	-
EACMV-UG-mir-1-2	-	-	+	-	-	-
EACMV-UG-mir-1-4	-	+	+	-	-	-
EACMV-UG-mir-1-5	-	-	+	-	-	-
EACMV-UG-mir-1-6	-	+	+	-	-	-
EACMV-UG-mir-1-7	-	+	+	-	-	-
EACMV-UG-mir-1-8	-	-	+	-	-	-
EACMV-UG-mir-1-9	-	-	+	-	-	-
EACMV-UG-mir-1-10	-	-	+	-	-	-
EACMV-UG-mir-1-11	-	-	+	-	-	-
EACMV-UG-mir-2-1	-	-	+	-	-	-
EACMV-UG-mir-2-2	-	+	+	-	-	-

Table S13 Detection of plant miRNAs in cassava and *Jatropha* samples. (+) amplied, (-) not amplified miRNAs

	Cassava				*Jatropha*	
Plant miRNA	S4C4	S2C6	S4C6	B2C15	K5J5	S4J12
miR156c	+	+	+	+	+	+
miR159b	+	+	+	+	+	+
miR160a	+	+	+	+	+	+
miR164c	+	+	+	+	+	+
miR169aa	-	-	-	-	-	-
miR170	+	+	+	+	+	+
miR171b	+	+	+	+	+	+
miR395b	+	+	+	+	+	+
miR397a	+	+	+	+	+	+
miR446	-	-	-	-	-	-
miR477g	-	-	-	-	-	-
miR472	+	+	+	+	+	+
miR478a	-	-	-	-	-	-
miR482	-	-	-	-	-	-
miR771	-	-	-	-	-	-
miR841	-	-	-	-	-	-
miR854a	+	+	+	+	+	+
miR857	-	-	-	-	-	-
miR859	-	-	-	-	-	-
miR868	-	-	-	-	-	-
miR948	-	-	-	-	-	-
miR1082b	+	+	+	+	+	+
miR1084	-	-	-	-	-	-
miR 1107	-	-	-	-	-	-
miR 1111	-	-	-	-	-	-
miR 1117	-	-	-	-	-	-
miR 1118	-	-	-	-	-	-
miR 1311	-	-	-	-	-	-
miR 1446a	-	-	-	-	-	-
miR 1510b	-	-	-	-	-	-
miR 1520j	-	-	-	-	-	-
miR 1864	-	-	-	-	-	-
miR 1887	-	-	-	-	-	-
miR 2094-3p	-	-	-	-	-	-
miR 2104	-	-	-	-	-	-
miR 2119	-	-	-	-	-	-
